# Unscrambling butterfly oogenesis

**DOI:** 10.1186/1471-2164-14-283

**Published:** 2013-04-26

**Authors:** Jean-Michel Carter, Simon C Baker, Ryan Pink, David RF Carter, Aiden Collins, Jeremie Tomlin, Melanie Gibbs, Casper J Breuker

**Affiliations:** 1 Evolutionary Developmental Biology Research Group, Faculty of Health and Life Sciences, Department of Biological and Medical Sciences, Oxford Brookes University, Gipsy Lane, Headington, Oxford, OX3 0BP, UK; 2 Bioline Reagents Ltd, 16 The Edge Business Centre, Humber Road, London, NW2 6EW, UK; 3 Non-coding RNA Research Group, Faculty of Health and Life Sciences, Department of Biological and Medical Sciences, Oxford Brookes University, Gipsy Lane, Headington, Oxford, OX3 0BP, UK; 4 NERC Centre for Ecology and Hydrology, Maclean Building, Benson Lane, Crowmarsh Gifford, Wallingford, OX10 8BB, UK

**Keywords:** Oogenesis, *Pararge aegeria*, Lepidoptera, *Bombyx mori*, *Drosophila melanogaster*, Transcriptome, Eco-evo-devo, Reproductive physiology, Maternal effects, Early embryogenesis

## Abstract

**Background:**

Butterflies are popular model organisms to study physiological mechanisms underlying variability in oogenesis and egg provisioning in response to environmental conditions. Nothing is known, however, about; the developmental mechanisms governing butterfly oogenesis, how polarity in the oocyte is established, or which particular maternal effect genes regulate early embryogenesis. To gain insights into these developmental mechanisms and to identify the conserved and divergent aspects of butterfly oogenesis, we analysed a *de novo* ovarian transcriptome of the Speckled Wood butterfly *Pararge aegeria* (L.), and compared the results with known model organisms such as *Drosophila melanogaster* and *Bombyx mori*.

**Results:**

A total of 17306 contigs were annotated, with 30% possibly novel or highly divergent sequences observed. *Pararge aegeria* females expressed 74.5% of the genes that are known to be essential for *D. melanogaster* oogenesis. We discuss the genes involved in all aspects of oogenesis, including vitellogenesis and choriogenesis, plus those implicated in hormonal control of oogenesis and transgenerational hormonal effects in great detail. Compared to other insects, a number of significant differences were observed in; the genes involved in stem cell maintenance and differentiation in the germarium, establishment of oocyte polarity, and in several aspects of maternal regulation of zygotic development.

**Conclusions:**

This study provides valuable resources to investigate a number of divergent aspects of butterfly oogenesis requiring further research. In order to fully unscramble butterfly oogenesis, we also now also have the resources to investigate expression patterns of oogenesis genes under a range of environmental conditions, and to establish their function.

## Background

Successful development relies heavily on parental contribution over and above the direct effect of maternal and paternal genes. For example, maternal effect genes, which have been particularly well studied in *Drosophila melanogaster,* are involved in setting up; 1) the location of the germ plasm and subsequent germ cell line development in the offspring [1-3] and, 2) a basic framework of positional information, which is interpreted by the embryo’s own genetic program [4,5]. Furthermore, insect embryos rely on nutrients for growth derived from the mother in the form of yolk deposited in the egg [6-9]. The investigation of insect egg production (i.e. oogenesis) is thus not only crucial in understanding reproductive, and consequently fitness variation [10-12], it is also a popular model system for studying epigenetic programming [13,14], the apoptotic pathway [15,16], stem cell behaviour [17-20], cell cycle regulation [21,22] and developmental patterning mechanisms in general [4,5,23-25].

Research into the physiological mechanisms underlying insect oogenesis and egg provisioning has a rich history [26], particularly in moths and butterflies (Lepidoptera) [7,8,27,28]. However, to date sufficiently detailed developmental genetic data to allow us to comprehensively understand the gene regulatory mechanisms underlying oogenesis and maternal effect gene expression controlling early embryogenesis only really exist for the model organism *D. melanogaster*[3-5,15,21]. Developmental genetic studies focussing on species other than *D. melanogaster* provide us with the opportunity to investigate how the Gene Regulatory Networks (GRNs) underlying insect oogenesis might have evolved [3-5,23].

Maternal effects can have consequences that extend well beyond embryonic or juvenile development, affecting offspring fertility and longevity [28,29]. The exact nature of the maternal effects and thus the contribution of a female to the phenotype (and fitness) of her offspring are not static, however, but to a large extent depend on her own internal state, resource availability [12,30] and in general the environmental conditions she experienced during her life (both biotic and abiotic) [31-34]. As such maternal effects constitute a form of non-genetic transmission of environmental conditions across generations. This means that elements of the regulatory states from the oogenesis GRN of a mother can be passed on to the next generation. There is thus a developmental framework in place with mothers having the possibility to influence the fecundity and survival of their offspring in response to their own environment, thereby providing an alternative system of inheritance with profound consequences for phenotypic evolution [32,35-38]. However, much of life history theory has been developed without regard to the actual developmental genetic basis of the variation in the traits being investigated, such as reproductive output and maternal effects [39-41]. What has been lacking is a powerful model system to study the developmental genetics of insect reproduction in an evolutionary ecological context [42]. Lepidoptera are ideal candidates to undertake such ecological evolutionary developmental (eco-evo-devo) studies given the vast amount of physiological data on oogenesis [8], as well as very detailed information, for butterflies in particular, on reproductive variability in relation to environmental variability [10,11,43-46].

Recently, valuable functional genomic tools have been developed for butterflies [47]; for example, for *Melitaea cinxia* to study life history variation [48], *Bicyclus anynana* to study wing colour patterning [49], the monarch butterfly *Danaus plexippus* to study long-distance migration [50], *Heliconius* species to study mimicry [51] and for both *Erynnis propertius* and *Papilio zelicaon* to study variability among populations in response to environmental heterogeneity and climate change [52]. The information that has been missing so far in butterflies is a detailed description of the ovarian transcriptome, including maternal regulation of patterning the embryo along its axes and mRNA contributed maternally to eggs. In fact, in Lepidoptera, there is a distinct lack of such developmental studies; only in the silkmoth *Bombyx mori* have a number of recent studies on candidate genes in maternal regulation of early embryogenesis (e.g. establishing positional information) been undertaken [53,54].

**Figure 1:**
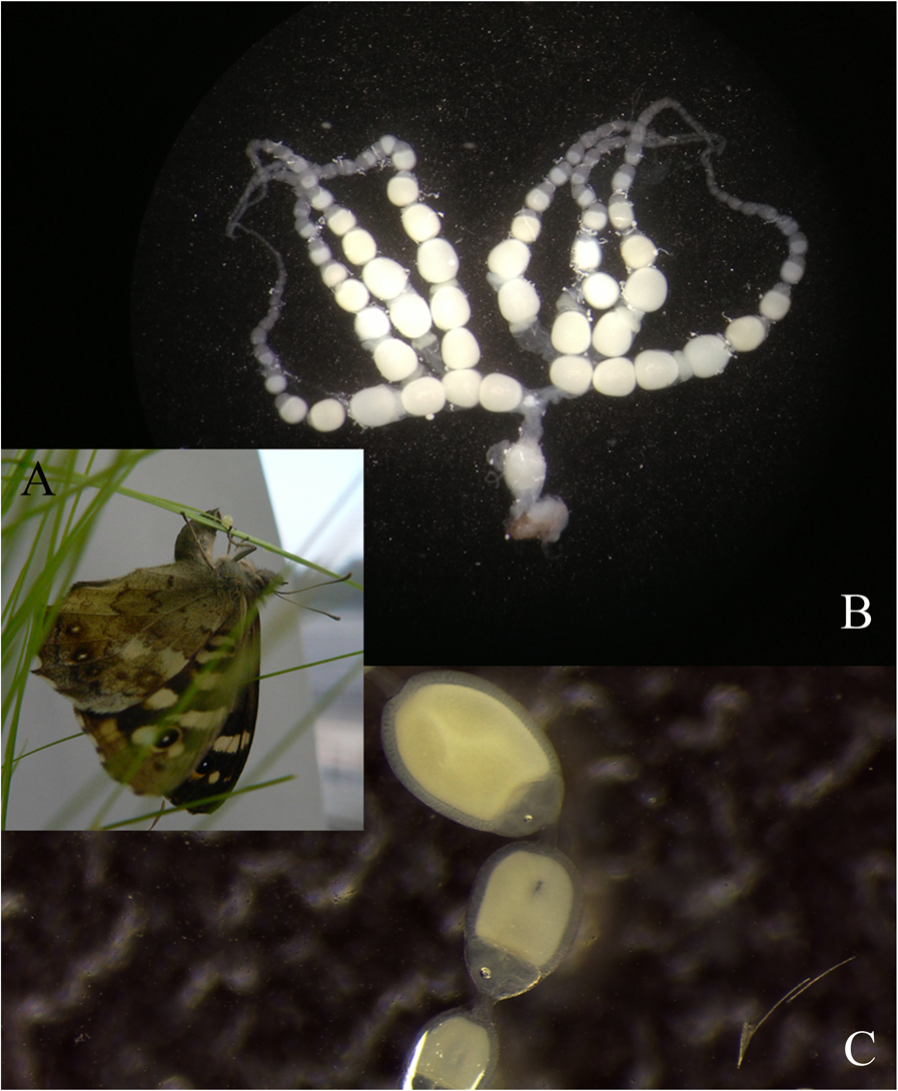
**Overview ovarian morphology of the Speckled Wood butterfly *Pararge aegeria.*** (**A**) Female *P. aegeria* laying an egg. (**B**) Complete meroistic *P. aegeria* ovary, consisting of a total of 8 ovarioles. Two times 4 ovarioles are attached to each other in the germarium region. Ovary in photo is still attached to the oviduct and part of the ovipositor. Only the ovaries were used for sequencing in this study. (**C**) Detail of previtellogenic eggs, with nurse and follicle cells visible.

The Speckled Wood butterfly *Pararge aegeria* (L.), a temperate zone species, is a popular model species for evolutionary ecology studies, for example on plasticity in female reproduction [10,11,55-57]. Female *P. aegeria* mate soon after emergence and usually mate only once [58]. At eclosion they have no or just a few [56] mature oocytes and if mated on the day of emergence, usually they start ovipositing 48 hrs later on the third day of their life [10,11]. In female *P. aegeria* resources for reproduction are, to a significant degree, obtained during the larval stage and there is little opportunity to obtain more nitrogenous resources for reproduction through adult feeding [59] or nuptial gifts. Like many other butterflies [8], *P. aegeria* has meroistic ovaries with 8 ovarioles. Each ovariole consists of a germarium (i.e. stem cell region), previtellogenic primary oocytes, vitellogenic eggs and mature chorionated eggs [8] (Figure 1). A total of seven nurse cells transfer maternal proteins, and mRNA of maternal effect genes into developing oocytes, whilst the somatic follicle cells surrounding the oocyte are involved in choriogenesis and vitellogenesis, as well as oocyte patterning [8].

In this paper, we present a comprehensive study of the genes expressed during oogenesis for the butterfly *P. aegeria*, using *de novo* transcriptome sequencing and qPCR. Given the wealth of data on reproductive physiology in Lepidoptera, the genes implicated in hormonal control of reproduction will be investigated in particular detail in this study. Furthermore, as a first step in determining the conserved and divergent elements of the butterfly oogenesis GRN (including maternal regulation of zygotic gene expression and embryonic patterning), we investigated which of the genes known to play an essential role in *D. melanogaster* or *B. mori* oogenesis were also transcribed by *P. aegeria*.

Although the number of ovarioles differs among *D. melanogaster*, *P. aegeria* and *B. mori*, these species have similar organisation of their meroistic ovaries, making for an ideal comparison. Furthermore, within Lepidoptera, the silkmoth *B. mori* and butterflies (including *P. aegeria*) belong to the more derived division Ditrysia within the infraorder Heteroneura and thus are likely to share developmental characteristics [60,61]. Many aspects of maternal regulation of early *D. melanogaster* embryogenesis can be explained by the fact that it is a long germ band insect [5]. Within the order of Lepidoptera there is a transition from a short germ in the more ancestral species to something more similar to long germ in the more derived species, such as those belonging to Ditrysia [60]. This fact, again, makes for an interesting comparison between the three species.

We describe particular features of the *P. aegeria* ovarian transcriptome that were revealed during assembly and annotation, including orthologs of genes involved in several major conserved signaling pathways, maternal regulation of early embryogenesis, vitellogenesis and choriogenesis. We observed that *P. aegeria* differed most significantly from *D. melanogaster* (and many other insect species) in terms of stem cell maintenance in the germarium, EGF signalling in establishing oocyte polarity along anterior-posterior (AP) and dorsal-ventral (DV), and the signalling mechanisms used at the termini of the oocyte. Furthermore, we observed a high proportion of apparently unique sequences in the transcriptome, and we discuss how future exploration of the function and expression patterns of these unique sequences will undoubtedly provide valuable insights into the evolution of insect oogenesis.

## Results

The main aim of this study was to identify the genes expressed in the ovaries involved in oocyte formation, establishing oocyte polarities and the RNA transcripts transferred into the eggs by the mother, which either regulate early embryogenesis or are needed during early embryogenesis. *Drosophila melanogaster* is arguably the best studied insect species in terms of ovarian gene expression and maternal effect gene function. Additional file 1 contains an extensively referenced list of the key essential oogenesis genes. FlyBase [62] and SilkBase [63] were used as a starting point to conduct the comprehensive literature search. The vast majority of papers thus mainly concern the model species *D. melanogaster* and *B. mori.* Furthermore, for *D. melanogaster* genes, a high-throughput developmental time series database was consulted for FPKM (Fragments Per Kilobase of exon per Million of fragments mapped) -based gene expression levels [64] (see also Methods), as well as an *in-situ* database for maternal transcript contribution to the oocyte [65]. The oogenesis genes discussed in this paper have been classified into functional groupings and were identified predominantly from *D. melanogaster* studies (and to a lesser extent *B. mori* studies). Studies on *D. melanogaster* oogenesis are too numerous to list exhaustively, but key relevant papers (and references therein) have been cited to enable the reader to explore the role of each particular gene during oogenesis further. It should of course be noted that quite a number of genes are expressed in different functional contexts during oogenesis, such as genes encoding the components of various signalling pathways or a gene such as *cornichon*, which is involved in setting up both AP and DV axis polarity as well as oocyte nucleus localisation in *D. melanogaster*[66]. Such genes only occur once in Additional file 1 and the tables presented in this paper, but the references to and discussion of such genes will highlight their pleiotropic functions.

### Annotation and verification of expression by means of qPCR

**Figure 2:**
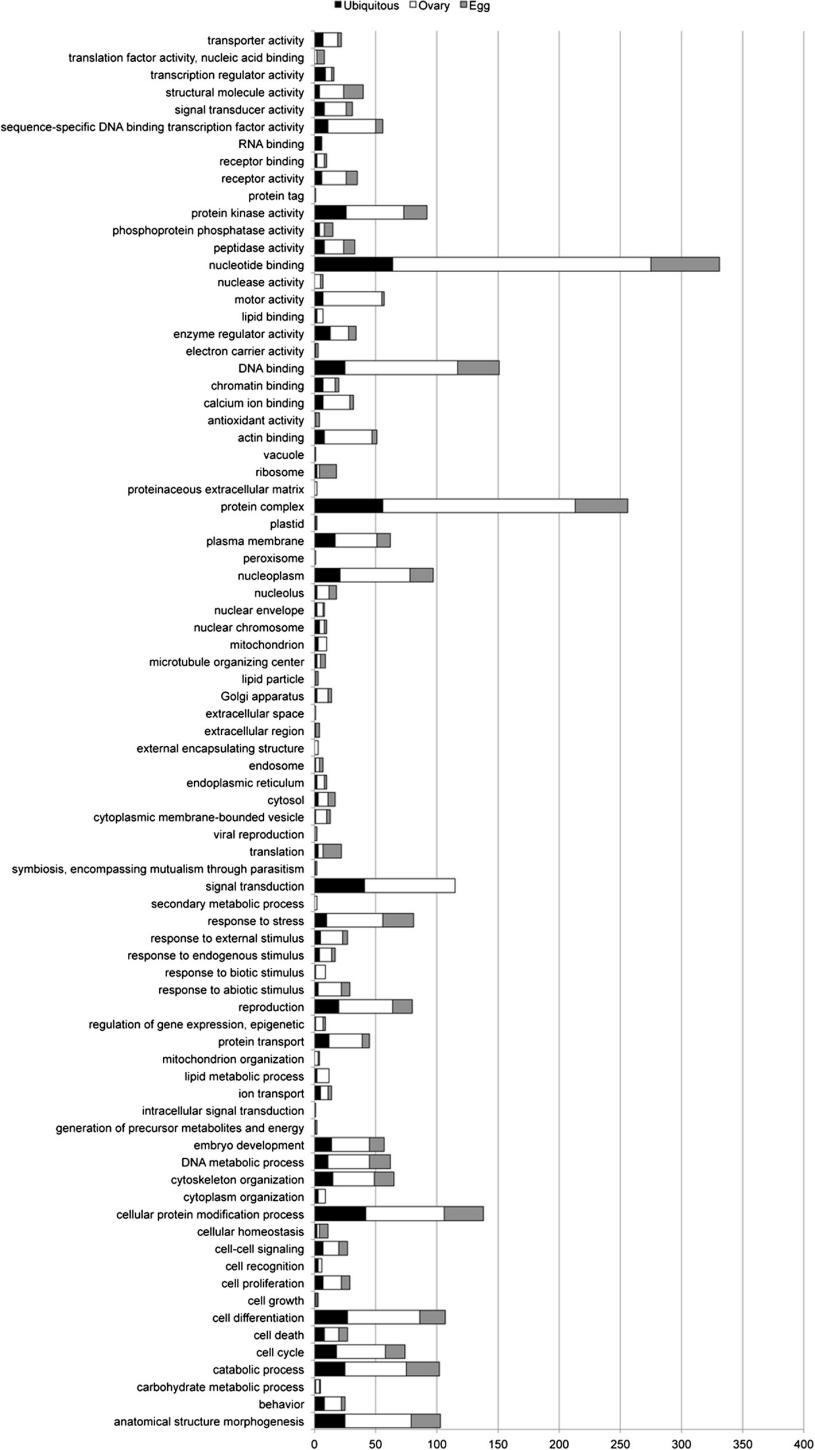
**Gene Ontology manually annotated genes.** The presence or absence of orthologs of essential oogenesis genes listed in Additional file 1 has been manually verified. The Gene Ontologies (GO) of genes that were present were determined by BLAST2GO and GO terms were subsequently condensed using the generic GO Slim subset. The histogram details the number of *Pararge aegeria* manually verified contigs (note, as has been observed for many *de novo* assemblies, for some genes multiple contigs were present in the transcriptome) for each GO term. FPKM estimates were used to compare the levels of transcripts found in the ovaries and as maternal transcripts in the egg. Using a Log2 fold change threshold of 1, genes were classified in the histogram as present in similar amounts in the egg and ovarian transcriptome (labelled *Ubiquitous*), used predominantly during oogenesis to make an egg, but not or hardly used as a maternal transcript (labelled *Ovary*), or highly concentrated in the egg as maternal transcripts (labelled *Egg*).

**Figure 3:**
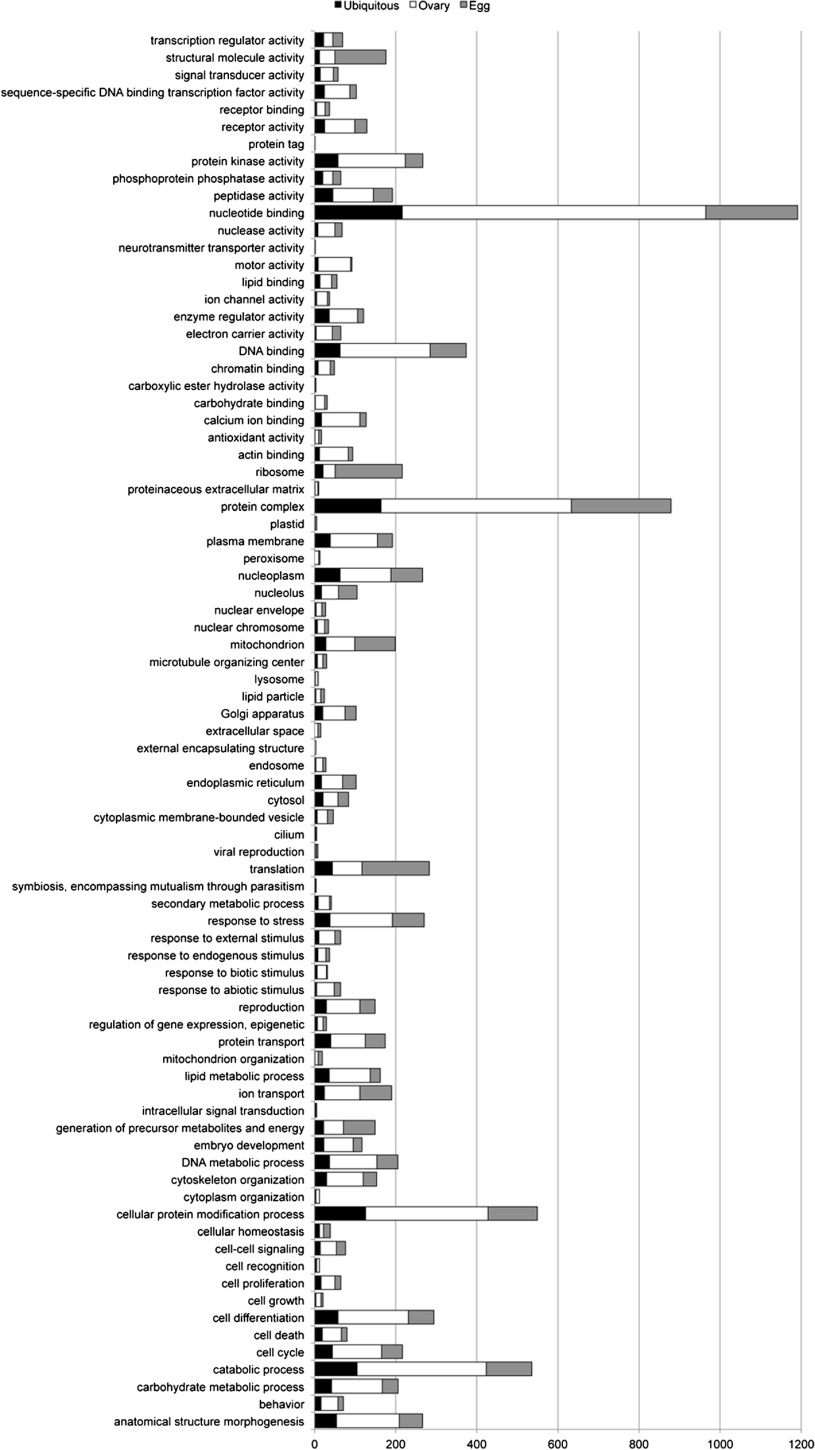
**Gene Ontology total transcriptome.** The Gene Ontologies (GO) of succesfully annotated genes in the total transcriptome were determined by BLAST2GO and GO terms were subsequently condensed using the generic GO Slim subset. The histogram details the number of *Pararge aegeria* contigs (note, for some genes multiple contigs were present in the transcriptome) for each GO term. FPKM estimates were used to compare the levels of transcripts found in the ovaries and as maternal transcripts in the egg. Using a Log2 fold change threshold of 1, genes were classified in the histogram as present in similar amounts in the egg and ovarian transcriptome (labelled *Ubiquitous*), used predominantly during oogenesis to make an egg, but not or hardly used as a maternal transcript (labelled *Ovary*), or highly concentrated in the egg as maternal transcripts (labelled *Egg*).

*Pararge aegeria* egg and ovary RNA was sequenced using Illumina short read RNA-Seq technology. Of the 25266 contigs, 17306 contigs were of sufficient quality and length to be annotated (both automated and manually) with 30%, possibly novel or highly divergent, remaining uncharacterised (Table 1; Additional file 2; see Methods). The presence or absence of *P. aegeria* orthologs in the transcriptome data of 1035 essential oogenesis genes listed in Additional file 1 was verified manually; 833 were found, which is 80.5%. A total of 994 genes out of the 1035 had been identified in *D. melanogaster* studies. *Pararge aegeria* expressed 741 of these, which is 74.5%. A further 56 genes were found to be expressed for which functionality during oogenesis can be inferred, but which have not been verified experimentally. Specific genes will be discussed elsewhere in this paper. A large number of these genes are not only transcribed during oogenesis to produce an oocyte, but maternal transcripts were also found to be present in the oocyte itself (Additional file 2; Figure 2). Exceptions include genes encoding chorion proteins as well as yolk and associated proteins. Large amounts of transcripts of these genes are found in the ovaries only (Additional file 2; Table 2). A number of contigs appeared to have relatively high transcript abundance (measured by means of FPKM values; see Methods) in the oocytes compared to the ovaries, suggesting that these transcripts are important as maternal effect transcripts incorporated into the oocytes in relatively large concentrations (Table 2 and Figure 2). An example of this is the gene encoding a signal transducing adaptor molecule (STAM; Table 2 and Additional file 2), which in *D. melanogaster* is expressed throughout oogenesis [67], but of which transcripts are detected in very high levels in early embryogenesis [68]. On the basis of the GO terms, the 838 gene orthologs appear to be representative of the annotated genes in the transcriptome as a whole (Figures 2 and 3).

**Table 1:** Transcript abundance

**Ovary/Egg LOG2 fold change**	**Egg/Ovary LOG2 fold change**	**FPKM - value**
spherulin-2A	signal transducing adapter molecule 1	ribosomal protein LP2
PACG20471	nucleolar GTP-binding protein 2	40S ribosomal protein S6
chorion class A precursor family 5	ubiquitin-conjugating enzyme E2 S	ribosomal protein L39
Bmtitin1	SLIT-ROBO Rho GTPase-activating protein	cytochrome oxidase subunit 3
Egg protein 80	mo-molybdopterin cofactor sulfurase	Bmtitin1
Vitellogenin	poly U binding factor 68kD	ribosomal protein L32
chorion class A precursor family 3	NADH dehydrogenase subunit 6	40S ribosomal protein S28
chorion class A precursor family 4	PACG6651	ubiquitin
PACG21670	chromatin regulatory protein sir2	Ferritin 2 – light chain homolog
chorion class C precursor family 2	PACG13792	BmBR-C gene for Broad-Complex isoform Z2
putative uncharacterized protein DDB	DNA repair protein complementing XP-A cells homolog	polyubiquitin
PACG20450	disulfide oxidoreductase	ribosomal protein L27
PACG21661	PACG710	60S ribosomal protein L28
PACG24051	similar to phosphinothricin acetyltransferase gene	PACG20761
chorion class B precursor family 1	PACG5386	60S ribosomal protein L18
chorion protein-like	abhydrolase domain-containing protein 1	translationally controlled tumor protein
endonuclease-reverse transcriptase	RAD51C protein	ribosomal protein S3A
spec2	PACG18339	60S ribosomal protein L38
PACG19208	PACG19350	ribosomal protein L7A
PACG20509	SLIT-ROBO Rho GTPase-activating protein 1-like	heat shock protein cognate 3

Transcript abundance (on the basis of FPKM values) in the *Pararge aegeria* ovarian/oocyte transcriptome (see also Additional file 2). The first column is a measure of which transcripts were most abundant in the ovaries compared to those present as maternal transcripts in the oocytes. These are genes highly expressed in the ovaries, but with few to no maternal transcripts in the oocyte. The genes listed in column 2 have relatively high FPKM values in the oocytes compared to the ovaries, indicating large concentrations of transcripts (see also Additional file 2). The third column lists the genes most transcribed during *P. aegeria* oogenesis. Columns list the gene top 20, from high to low.

**Table 2:** Sequencing and annotation summary

**Location/Feature**	**Contigs annotated**	**Manually curated**	**Av. Contig (bp)**	**Av. CDS (bp)**	**Av. 5' UTR (bp)**	**Av. 3'UTR (bp)**
**Genomic**	**16919**	**1564**	**625.99**	**459.89**	**69.61**	**75.17**
Complete CDS	4530	473	1022.96	667.12	142.07	210.79
*Homology*	*3055*	*466*	*1196.75*	*855.06*	*124.15*	*214.53*
*Novel*	*1475*	*7*	*663.02*	*277.87*	*179.18*	*203.02*
Partial CDS	11842	992	485.34	393.59	45.11	26.77
*Homology*	*8054*	*975*	*521.96*	*454.21*	*51.65*	*12.67*
*Novel*	*3788*	*17*	*407.48*	*264.69*	*31.20*	*56.73*
Partial mRNA	547	99	383.36	179.24	0.00	0.00
**Mitochondrion**	**387**	**11**	**728.64**	**563.20**	**83.18**	**75.32**
Complete CDS	177	7	996.59	719.80	115.86	157.94
Partial CDS	201	3	510.06	443.30	58.13	5.95
Partial mRNA	9	1	340.67	161.22	0.00	0.00
**Grand Total**	**17306**	**1575**	**628.28**	**462.20**	**69.91**	**75.18**

A total of 17306 sequences have been submitted. The sequences are classified below according to their location (i.e. nuclear or mitochondrial genome), completeness and annotation status (i.e. whether orthoogous sequences could be found in other Metazoa or not). Characteristics of the contigs are listed, such as average size of the contig, coding sequence and the 3’ and 5’ UTRs (all in base-pairs, bp).

For of a subset of 17 genes, sampled across the functional groups identified in Additional file 1, the expression in the ovarioles and the presence of transcripts in the oocyte were confirmed further by means of RT-qPCR. These genes were: *argonaute 2* (*AGO2*), *caudal* (*cad*), *decapentaplegic* (*dpp*), *egalitarian* (*egl*), *exuperantia* (*exu*), *Fragile X mental retardation 1* (*Fmr1*), *nanos-like* (*nos-like*), *nanos-M* (*nos-M*), *nanos-O* (*nos-O*), *ornithine decarboxylase antizyme* (*Oda*), *anterior open* (*aop*), *par-1*, *piwi*, *chorion b-ZIP transcription factor* (*CbZ*), *staufen* (*stau*), *vitellogenin receptor yolkless* (*yl; VgR*) and *vitellogenin* (*Vtg*/*Vg*). Two further genes, which have not been explicitly studied in the context of oogenesis (references in Additional file 1), were investigated: *embryonic lethal abnormal vision* (*elav*) and *minibrain* (*mnb*). Furthermore, 3 housekeeping genes were selected to be used as reference genes: *RNA polymerase II 215 KD subunit* (*RPII215*), *TATA binding protein* (*Tbp*) and *zwischenferment* (*zw, G6PDH*) (Additional file 3).

**Figure 4:**
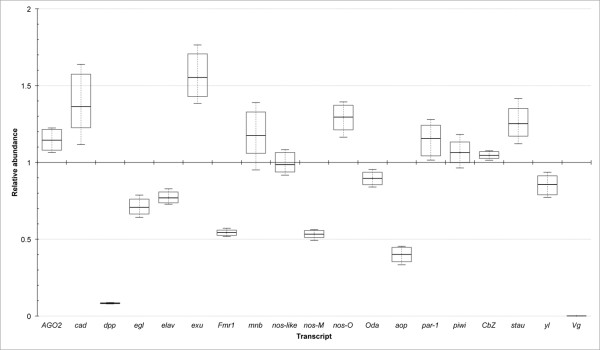
**qPCR results.** Normalised relative abundance of transcripts for 19 genes of interest. Data above the midline (the median gene expression level set at 1) indicate a relatively high number of transcripts in the oocyte compared with the ovary. Boxes represent the interquartile range. Whiskers represent the minimum and maximum observations. Note *Vtg*/*Vg* transcripts were not found in the oocyte*.*

The qPCR results were used to confirm the presence of expression as well as the levels of expression (as indicated by means of FPKM values) in the transcriptome dataset (Figure 4; Additional files 4, 5, and 6). Transcripts of vitellogenin were not transferred into the oocytes and very few *dpp* transcripts were transferred into the egg (Figure 4). All of the other oogenesis genes investigated by means of qPCR were included as maternal effect gene transcripts in the oocytes (see also Additional file 2). Specific qPCR results will be discussed in the remainder of the paper.

## Discussion

### Germ-line and ovarian stem cells

In *D. melanogaster* three major signalling pathways play a significant role in cystoblast differentiation, and the maintenance and division of germ-line and ovarian stem cells; TGF-beta, Wnt and hedgehog signalling [69-71]. Components of all three signalling pathways have been identified for *P. aegeria* (Table 3 and Additional file 1). However, it is not clear, to what extent these signalling pathways are essential in the Lepidopteran germarium, as they were not identified as such in *B. mori* using SAGE analyses [72]. Rather than signalling, for example, a previously unidentified non-coding RNA appears to regulate cystoblast differentiation in *B. mori*[72].

**Table 3:** Maintenance and division of germ-line and ovarian somatic stem cells


*armadillo* (*arm)*	Y	*shutdown* (*shu)*	Y
*axin; axis inhibition protein* (*axn)*	Y	*FK506-binding protein* (*FKBP59)*	Y
*dishevelled* (*dsh)*	Y	*vasa; vasa-like gene (vasa* homolog in Lepidoptera*)* (*vas; vlg)*	Y
*shaggy; gsk-3* (*sgg; Zw3)*	Y	*outstretched* (*upd; sisc)*	N
*sugarless; UDP glucose6 dehydrogenase* (*sgl; UDPGDH)*	Y	*bag of marbles* (*bam*	N
*legless* (*lgs; BCL9)*	Y	*mei-p26* (*mei-p26)*	N
*pygopus* (*pygo; gam)*	Y	*brain tumor* (*brat)*	Y
*wingless* (*wg)*	Y?	*benign gonial cell neoplasm* (*bgcn)*	N
*wntless; evenness interrupted* (*wls; Evi)*	Y	*within bgcn* (*wibg; pym)*	Y
*hedgehog* (*hh)*	Y	*decapentaplegic* (*dpp)*	Y
*shifted; wnt inhibitory factor 1 precursor* (*shf; wif1)*	Y	*kekkon5* (*kek5 )*	N
*costa* (*cos2)*	N	*Mothers against dpp* (*Mad)*	Y
*skinny hedgehog; hedgehog acyltransferase;* CG32281 (*ski)*	Y	*Smad on X* (*Smad2; Smox)*	Y
*roadkill;* similar to *speckle-type POZ protein* (*rdx)*	Y	*saxophone* (type I Dpp receptor) (*sax)*	N
*patched* (*ptc)*	N	*thickveins* (type I Dpp receptor) (*tkv)*	Y
*smoothened* (*smo)*	Y	*punt* (type II Dpp receptor) (*pnt)*	N
*cubitus interruptus* (*ci)*	Y	*medea* (*med; SMAD4)*	N
*engrailed* (*en)*	N	*Daughters against dpp* (*Dad)*	N
*pangolin* (*pan; Tcf/LEF)*	Y	*glass bottom boat* (*gbb)*	Y
*wnt oncogene analog 4* (*wnt4)*	N	*dullard* (*dd)*	Y
*dicer-1* (*dcr-1)*	Y	*quo vadis; schnurri* (*quo; shn)*	N
*loquacious* (*loqs)*	Y	*lethal with a checkpoint kinase* (*smurf; lack)*	Y
*mir-184* (*mir-184)*	N	*supernumerary limbs* (*slimb)*	Y
*effete* (*eff; UbcD1)*	Y	*starry night; flamingo* (*stan; fmi)*	N
*fs(1)Yb* (*Yb)*	N	*roughened;* similar to *ras-related protein rap-1a; enhancer of faf;* similar to *Bombyx mori ras3* (*r; rap1; dras3)*	Y
*fused;* similar to *serine/threonine kinase 36* (*fu)*	Y	*ras-associated protein 2-like; ras-related protein 2 (rap2l)*	Y
*Suppressor of fused* (*Su(fu))*	Y	*fruitless isoform a* (*fru)*	Y
*bicaudal* (*bic)*	Y	*fruitless isoform k* (*fru)*	Y
*otefin* (*ote)*	N	*fruitless* (*fru)*	Y
*piwi* (*piwi)*	Y	*sex-lethal* (*sxl)*	N
*pelota* (*pelo)*	Y	*pre-mRNA-splicing regulator wtap;* similar to *female lethal d;* CG6315 (*fl(2)d )*	N
*pumillio* (*pum)*	Y	*maleless;* ATP-*dependent RNA helicase a-like* (*mle; dhx9; nap)*	Y
*penguin* (*pen)*	Y	*lamin c* (*lamc)*	Y
*sans fille; U1 small nuclear ribonucleoprotein A; fs(1)1621* (*snf)*	Y	*clift; eyes absent* (*cli; eya)*	Y
*bric a brac* (*bab)*	N	*slowmo* (*slmo)*	Y

Genes identified mainly from the *Drosophila melanogaster* literature as functioning early for the maintenance and division of germ-line and ovarian somatic stem cells. Presence (Y), possible presence (Y?) or absence (N) of orthologous transcripts in the *Pararge aegeria* transcriptome is indicated.

The TGF-beta ligands *glass bottom boat* (*gbb*) and *dpp* were expressed in *P. aegeria* ovarioles (qPCR results; Table 3). The type I TGF-beta receptors used were *thickveins* (*tkv)* and an activin type 1 receptor similar to *baboon* (*ATR1*) (Additional files 1 and 2), the latter of which is present in the *D. melanogaster* oocyte as a maternal transcript necessary for early embryogenesis [73]. No evidence, however, could be found for an ortholog of activin type I receptor *saxophone* (*sax*) (Table 3)*.* No ortholog of the activin type II receptor *punt* (*pnt*) was found, although PACG16964 was found to be a type II BMP receptor (Additional file 2). The *P. aegeria* transcriptome contained orthologs of two SMAD family genes; *Mothers against dpp* (*Mad*) and *Smad on X* (*Smox*), but not of *medea* nor of the anti-SMAD *Daughters against decapentaplegic* (*Dad*), which have been shown to be of importance in *D. melanogaster* germline stemcell maintenance [71]. Furthermore, the negative regulator of Dpp signalling *dullard* (*dd*) was found to be expressed in *P. aegeria* ovaries. In *D. melanogaster* this gene plays a role in wing vein formation [74], and although it has been found to be maternally deposited [65], its role in oogenesis has not been verified. Another negative regulator of Dpp signalling, *brinker* (*brk*), which plays a role in eggshell patterning in *D. melanogaster*[75,76], was also expressed by *P. aegeria*. In *D. melanogaster, bag of marbles* (*bam*) interacts with Dpp signalling to regulate stem cell maintenance and differentiation in the germarium [77]. However, *bam* is a *Drosophila* unique gene and is not found in *P. aegeria*.

During oogenesis *P. aegeria* females express two Wnt receptors, which show orthology to *frizzled-2* and *frizzled-7* (Table 4 and Additional file 1). Furthermore, they express the Wnt receptor *l(2)43Ea* (*boca)*, which plays a role in *D. melanogaster* vitellogenesis [78], as well as *dishevelled* (*dsh*), which is part of the Wnt receptor complex (Table 3 and Additional files 1 and 2). Other components of the Wnt pathway expressed include *armadillo* (*arm*), *pangolin* (*Tcf/LEF*), *groucho* (*gro*), *axin* (*axn*), *sugarless* (*sgl*), *legless* (*lgs*), *pygopus* (*pygo*) and s*haggy* (*sgg*; *Zw3*), as well as *wntless* (*wls*)(Table 3 and Additional file 2; references in Additional file 1). Maternal transcripts of each of these genes were found in the oocyte (Table 3; Additional files 1 and 2), with the exception of *sgl*. Asymmetric localisation of maternal *axn* RNA has been shown to be involved in AP formation in *Tribolium castaneum*[79]. Rather interestingly, the ligand *wingless* (*wg*) was not found in the assembled transcriptome (Table 3 and Additional file 2). However, 201 ovary and 100 oocyte raw RNA-seq reads mapped against the complete *wg* CDS from our unpublished *P. aegeria* genome (approximately between 3.2× and 6.5× coverage, displaying a discontinuous transcript with a number of gaps not covered by reads; Additional file 7). In *D. melanogaster*, transcripts of *wg* are not found in the oocyte [65] and although Wnt signaling has been established as present during oogenesis [69], expression levels of *wg* are extremely low [64], making it hard to detect the transcripts. It is clear that in *P. aegeria* there is strong maternal contribution to zygotic Wnt signaling (Additional file 2), but whether Wnt signaling plays a role during oogenesis needs to be further investigated.

**Table 4:** Cytoskeleton and actomyosin contractile ring assembly


*abnormal spindle (a microtubule-associated protein)* (*asp)*	N	*dedicator of cytokinesis 6,7;* similar to CG11376 (*dock6; dock7)*	Y
*javelin-like (microtubule-associated protein);* similar to CG3563 (*jvl)*	Y	*myoblast city; dedicator of cytokinesis 1* (*mbc; dock180)*	Y
*mini spindles (microtubule-associated protein;* belongs to *xmap215/tog family of genes)* (*msps; xmap215)*	Y	*spaghetti squash; myosin light polypeptide 9; myosin regulatory light chain 9* (*sqh; mrlc)*	Y
*a-kinase anchor protein 200* (*akap200)*	N	*nonmuscle myosin essential light chain; myosin II essential light chain* (*mlc-c)*	Y
*capulet; act up, bcDNA:ld24380,* CG5061 (*capt)*	N	*myosin regulatory light chain interacting protein* (*mylip)*	Y
*cdc42* (*cdc42)*	Y	*genghis kahn; cdc42 binding protein kinase alpha or beta* (*gek; cdc42bpb)*	Y
*Bombyx mori cdc42 small effector 2-like protein (*LOC692865*)* (*cdc42-sep2; spec2)*	Y	*jaguar/myosin VI* (*jar; mhc95f; myo6)*	Y
*p21/cdc42/rac1 activated kinase* (*pak)*	Y	*myosin heavy chain (*similar to CG17927*)* (*mhc)*	Y
*rac1; ras-related c3 botulinum toxin substrate 1* (*rac1)*	Y	*myosin heavy chain 2; zipper* (*zip; mhc2)*	Y
*specifically Rac1 associated protein; Fmr1-interacting protein* (*sra-1; cyfip)*	Y	*myosin light chain kinase; bent; titin-like* (*bt)*	Y
*engulfment and cell motility protein; ced-12 homolog* (*ced-12; elmo)*	Y	*myosin 1 light chain; myosin alkali light chain 1* (*mlc)*	Y
*centrosomin* (*cnn)*	Y	*myosin 1; myosin 61f* (*myo1b)*	Y
*aurora-a* (*aur)*	Y	*dilute class unconventional myosin; myosin V; myosin-Va* (*myoV; myo-Va; didum)*	Y
*chickadee (*homolog of *profilin)* (*chic)*	Y	*unconventional myosin class XV* (*myo10a)*	Y
*citron; sticky* (*sti; dck)*	N	*myosin heavy chain like* (*mhcl)*	Y
*focal adhesion kinase-like; fak56(D)* (*fak56D)*	Y	CG17293*; WD40 protein type* (*wdr82)*	Y
*diaphanous* (*dia)*	Y	*washout* (*wash; p63; p65)*	N
*frizzled; frizzled-7-like* (*fz7-l)*	Y	*james bond* (*bond)*	N
*frizzled; frizzled-2-like* (*fz2-l)*	Y	*kette; hem-protein;* similar to *membrane-associated protein hem (dhem-2);* similar to *membrane-associated protein gex-3* (*hem; kte; nap1; dhem2)*	Y
*chromosome bows; mast; orbit; clasp* (*chb)*	N	*short stop; kakapo;* similar to *bullous pemphigoid antigen 1 (Homo sapiens); microtubule-actin cross linking factor 1* (*shot)*	Y
*shotgun; E-Cadherin* (*shg; E-Cad)*	Y	*vacuolar protein sorting 35* (*vps35)*	Y
*mushroom body defect* (*mud)*	N	*rotund; racGTPase-activating protein; roughened eye* (*rn; roe; rnracgap)*	Y
*dishevelled associated activator of morphogenesis-1* (*daam-1)*	Y	*twinstar; actin-depolymerizing factor 1 cofilin* (*tsr)*	Y
*karst (*also known as *betaheavy spectrin)* (*kst)*	Y	*slingshot* (*mkp; ssh)*	Y
*flightless I* (*fliI)*	Y	*subito; double or nothing; Bombyx mori kinesin-like protein c* (*sub)*	Y
*klarsicht* (*klar; marb)*	Y	*IplI-aurora-like kinase; aurora b (kinase)* (*aurb)*	Y
*muscle-specific protein 300* (*msp-300)*	Y	*tumbleweed; racGAP50c;* similar to *racGTPase-activating protein* (*tum; racGAP)*	Y
*lissencephaly-1* (*lis-1)*	Y	*arp2; actin-related protein 14d* (*arp2; arp14d)*	Y
*cortactin(−like)* (*cortactin)*	Y	*arp3; actin-related protein 66b* (*arp3; arp66b)*	Y
*src oncogene at 42a* (*src42a)*	Y	*suppressor of profilin 2 (*also known as *arpc1)* (*sop2; arpc1; arc41)*	Y
*src oncogene 1* (*src64b)*	Y	*arp2/3 complex subunit p34; arpc2* (*arpc2; arc-p34)*	Y
*α actinin* (*actn)*	Y	*arp2/3 complex 21kD subunit p21; arpc3b* (*arpc3; arpc3b)*	Y
*ovarian tumor; fs(1)m101; fs(1)231* (*otu*	N	*arp2/3 complex subunit p20; arpc4* (*arpc4; arc-p20)*	Y
*Guanyl cyclase at 32e* (*Gyc32e)*	N	*arp2/3 complex 16kD subunit p16; arpc5* (*arpc5; p16-arc)*	Y
*Guanylyl cyclase at 76c; receptor-type Guanylate cyclase* (*Gyc76c)*	Y	*kinesin associated protein 3* (*kap3; kap)*	Y
*stand still* (*stil)*	N	*kinesin-like protein at 68d; kinesin II; kinesin-2* (*klp5; klp68d )*	Y
*hold up* (*hup)*	N	*kinesin-like protein at 64d; kinesin family member 3a* (*klp64d; kif3a)*	Y
*dicephalic* (*dic)*	N	*pericentrin-like protein (cp309)* (*cp309)*	N
*kelch* (*kel)*	Y	*rho-type Guanine exchange factor; pak-interacting exchange factor;* AGAP007877 (*rtgef; dpix)*	Y
similar to *kelch domain containing 4* (*klhdcp)*	Y	*SCAR; actin binding protein; (*in vertebrates*) wiskott-aldrich syndrome protein family member 2; wasp family protein member 2* (*SCAR; wave)*	Y
*cullin 3* (*cul3)*	Y	*quail; villin* (*qua)*	Y

Genes identified mainly from the *Drosophila melanogaster* literature as important in cytoskeleton and actomyosin contractile ring assembly. Presence (Y) or absence (N) of orthologous transcripts in the *Pararge aegeria* transcriptome is indicated.

No ortholog of *Drosophila wnt4* (a vertebrate *wnt9* ortholog) was found (Table 3), which in *D. melanogaster* is involved in regulating cell movement during ovarian morphogenesis [80]. Finally, transcripts of an ortholog of *shifted* (*shf*) were present both in the ovary and oocyte in *P. aegeria* (Table 3 and Additional file 2). This gene encodes an EGF-like protein acting as a Wnt inhibitory factor 1, which in *D. melanogaster* stabilises hedgehog signalling and transcripts of which are deposited in the oocyte [81]. *Hedgehog* (*hh*) itself, as well as components of the pathway including s*moothened* (*smo*), *fused* (*fu*), *Suppressor of fused* (*Sufu*), and *cubitus interruptus* (*ci*) were all found to be expressed and maternal transcripts of all were present in the oocyte (Table 3; Additional files 1 and 2). Both *costa* (*cos2*) and the receptor *patched* (*ptc*) were not expressed during oogenesis by *P. aegeria* (Table 3; Additional file 1). Although Ptc protein has been detected in the *D. melanogaster* germarium [70], detecting *ptc* transcripts may prove more difficult because *ptc* appears to be transcribed in very low amounts [64], and it is possible that this is why *ptc* transcripts were also not found in *P. aegeria*. As has been observed for Wnt signalling, there is a maternal contribution to zygotic Hh signalling, but presently it is not clear whether this signalling pathway plays a significant role during *P. aegeria* oogenesis.

### Cytoskeleton and actomyosin contractile ring assembly

Orthologs of the vast majority of genes that have been described as affecting the cytoskeleton and actomyosin contractile ring during *D. melanogaster* oogenesis were expressed in *P. aegeria* (Table 4). One of the genes not found is *ovarian tumor* (*otu*), which plays a crucial role during *D. melanogaster* oogenesis. Otu is involved in cytoskeletal formation, cyst formation in germ-line cells, nurse cell chromosome dispersion and *gurken* (*grk*) mRNA localisation [82]. For 14 genes no *P. aegeria* orthologs could be found in the dataset (Table 4). For a number of these, this is not surprising, as in general it has proven to be difficult to find orthologs outside the genus *Drosophila*; for example *dicephalic* (*dic*), *mushroom body defect* (*mud*), *hold up* (*hup*) and *stand still* (*still*)(references in Additional file 1).

*Pararge aegeria* females were found to express *E-Cadherin* (Table 4). E-Cadherin-dependent adhesion underlies the positioning of the oocyte at the posterior of the cyst, which in turn plays a role in establishing the AP polarity in *D. melanogaster* during very early oogenesis [83].

### Oocyte determination (including fusome formation) and formation of the anterior-posterior polarity during the early stages of oogenesis

Three genes have been described in the literature as important in *D. melanogaster* follicle ring canal formation; *visgun* (*vsg*), *nasrat* (*fs(1)N*) and *scraps* (*scra*)[84,85]. Only *fs(1)N* was not transcribed by *P. aegeria* females (Additional file 1). Fusomes, regions of spectrin-rich cytoplasm, are essential in *D. melanogaster* to establish a system of directional transport between cystocytes underpinning oocyte determination and subsequent oocyte polarity [86]. The majority of genes that are expressed early in *D. melanogaster* oogenesis regulating the formation of the fusome (e.g. *alpha* and *beta spectrin* and *hu-li tai shao*) were also transcribed by *P. aegeria*, as well as the genes involved in establishing initial AP polarity, including *par-1* and *egalitarian* (*egl*) (Figure 4 qPCR results and Table 5; references in Additional file 1). Par-1 in particular is essential in *D. melanogaster* for both oocyte determination and for establishing AP polarity through its effects on the organisation of the microtubule cytoskeleton in conjunction with a number of other proteins [87]. Among the proteins with which Par-1 interacts in establishing AP polarity are Bazooka (Baz/Par3), Bicaudal D (BicD), Lkb1/Par4, Egl, 14-3-3epsilon, and Dynein proteins (references in Additional file 1). The genes encoding these proteins were all expressed by *P. aegeria* (Table 5). Transcripts of both *par-1* and *egl* were also present in the oocyte (Figure 4 qPCR results and Additional file 2).

**Table 5:** Oocyte determination, fusome and AP polarity


*transitional endoplasmic reticulum ATPase; ter94* (*ter94)*	Y	*atypical protein kinase c;* CG10261 (*apkc)*	N
*capping protein alpha* (*cpa)*	Y	*typical protein kinase c* (*pkc)*	Y
*leonardo* (*14-3-3zeta; leo)*	Y	*protein kinase c inhibitor;* similar to CG2862 (*pkc inhibitor)*	Y
*bazooka* (*baz; par3)*	Y	*rab-protein 6; small (monomeric) GTPase* (*rab6)*	Y
*bicaudal C* (*bicC)*	Y	*rhino* (*rhi)*	N
*bicaudal D* (*bicD)*	Y	*ß1 tubulin 1* (*tub1)*	Y
*bicaudal D-related* (CG32137)	Y	*ß1 tubulin 2* (*tub2)*	Y
*glued; dynactin* (*gl)*	Y	*β-tubulin at 60d* (*tub3; betatub60d)*	Y
*egalitarian; 3'-5' exonuclease domain-like-containing protein* (*egl)*	Y	*β-tubulin at 56d* (*betatub56d)*	Y
*stonewall; fs(3)02024* (*stwl)*	N	homologous to *Drosophila γ-tubulin at 37c; gamma tubulin (*in general*)* (*gammatub37c; gamma tub 1)*	Y
*egghead; zeste-white 4; beta-1,4-mannosyltransferase* (*egh; zw4; bre3)*	Y	*gamma-tubulin complex component 3; lethal (1) discs degenerate 4* (*tubgcp3; gcp3; dgrip91)*	Y
*4ehp* (*4ehp)*	N	*gamma-tubulin complex component 2; gamma-tubulin ring protein 84 (Drosophila)* (*tubgcp2; gcp2; dgrip84)*	Y
*pipsqueak (BTB/POZ containing gene)* (*psq)*	N	*alpha tubulin tua1;* similar to *Drosophila alpha-tubulin at 84b* (*atub; tua1)*	Y
*BTB/POZ domain containing gene* (*BTB-POZ)*	Y	*alpha tubulin tua2;* similar to *Drosophila alpha-tubulin at 84b* (*atub; tua2)*	Y
*BTB domain containing protein 2* (*BTBd2)*	Y	*deadlock* (*del)*	N
*spindle c* (*spnc)*	N	*mo25; calcium-binding protein 39* (*mo25)*	Y
*coracle; band 4.1-like protein* (*cora)*	Y	*14-3-3ϵ* (*14-3-3epsilon)*	Y
*alpha spectrin* (*alpha-spec)*	Y	*par-1; map/microtubule affinity-regulating kinase* (*par-1)*	Y
*beta spectrin* (*beta-spec)*	Y	*serine/threonine kinase lkb1; partitioning defective 4* (*lkb1; par4; stk11)*	Y
*hu-li tai shao* (*hts)*	Y	*partitioning defective 6* (*par-6)*	N
*ankyrin;* similar to *ankyrin 2,3/unc44;* AGAP002272-PA (*ank)*	Y	*combgap* (*cg; mig)*	Y
*neuroglian* (*ceb; nrg)*	Y	*dynein heavy chain 64C; cytoplasmic dynein heavy chain* (*dhc64c; dhc)*	Y
*inscuteable* (*insc)*	N	*cut up* (*ddlc-1; cdlc1; dynein light chain)*	Y
*sec61 alpha* (*sec61 alpha)*	Y	*kinesin heavy chain* (*khc)*	Y
*sec61 gamma* (*sec61 gamma)*	Y	*kinesin light chain* (*klc)*	Y
*sec63* (*sec63)*	Y	*rhomboid-2; stem cell tumor; brother of rhomboid* (*stet; rho-2)*	N
*tropomodulin* (*tmod)*	Y	*ensconsin* (*ens)*	Y
*p38 MAPK* (*p38MAPK)*	Y	*helicase at 25e;* ATP-*dependent RNA helicase; ddx39 (*in vertebrates*)* (*hel25E; ddx39)*	Y
*protein kinase a; cAMP-dependent protein kinase 1; dc0, pka* (*pka-c1)*	Y	*licorne;* similar to *dual specificity mitogen-activated protein kinase kinase 3;* similar to *dual specificity mitogen-activated protein kinase kinase (*in *Nasonia); dual specificity mitogen-activated protein kinase kinase 6 (*mainly in vertebrates*)* (*lic; MAPKK; mek3)*	Y
*cAMP-dependent protein kinase r1* (*pka-r1)*	Y	*protein tyrosine phosphatase 10D* (*ptp10D)*	Y
*cAMP-dependent protein kinase r2* (*pka-r2)*	Y	*protein tyrosine phosphatase 4E;* similar to *protein tyrosine phosphatase 10D* (*ptp4E)*	Y

Genes identified mainly from the *Drosophila melanogaster* literature acting early in the egg for oocyte determination (including fusome formation) and formation of the anterior-posterior (AP) axis. Presence (Y) or absence (N) of orthologous transcripts in the *Pararge aegeria* transcriptome is indicated.

Soon after the posterior localisation of the oocyte in the *D. melanogaster* cyst, EGF signalling takes place in the posterior between the oocyte (Grk ligand) and the overlying follicle cells (Torpedo receptor) [88,89], further consolidating AP polarity. Orthologs of the fast-evolving *grk* are difficult to find outside the genus *Drosophila*[24]. Two genes encoding EGF ligands and likely to be paralogs of *grk*, *spitz* (*spi*) and *keren* (*krn*), are involved in the regulation of border cell migration in *D. melanogaster*[90]. A single *spi*/*krn*-like EGF ligand has been found in the genomes of *N. vitripennis* and *T. castaneum*, and has been argued to be functionally similar to *grk* in DV patterning in these species [24]. *Pararge aegeria* females expressed an ortholog of this single *spi*/*krn*-like EGF ligand, with the sequence displaying significant similarity to *Harpegnathos saltator spi* (Additional file 2; Table 6). Large amounts of these transcripts were detected in the *P. aegeria* oocyte (Additional file 2), suggesting a significant role for its use during early embryogenesis as observed in *D. melanogaster*[65]. Given the expression of a *spi*/*krn* in *P. aegeria* and the significance of EGF signalling in insect oogenesis in general, and establishing oocyte polarity in particular [24], it is very surprising that only weak evidence was found for expression of *egfr*, the gene encoding the EGF receptor, in *P. aegeria* ovaries (Table 6). None of the contigs in our *de novo* assembly could be clearly identified as an *egfr* transcript. However, 780 raw RNA-seq reads did map against the complete *efgr* CDS from our unpublished *P. aegeria* genome (approximately 7.1× coverage, displaying a discontinuous transcript with a number of gaps not covered by reads; Additional file 7). Intriguingly, all of the raw reads that mapped successfully came from the ovariole transcriptome, not the oocyte transcriptome, consistent with the importance of EGF signalling during oogenesis itself. Transcript levels of *egfr* are low to moderate in *D. melanogaster* ovaries [64], and thus there is always the possibility, as was suggested for the absence of *ptc* transcripts in our study, that *P. aegeria egfr* transcript levels were not high enough to be accurately detected. However, it is intriguing that as for a number of other components of the EGF pathway involved in DV patterning in *D. melanogaster*, *P. aegeria* also did not transcribe, for example, *rho* during oogenesis (Table 6). Spatial restriction dorsally of *rhomboid* (*rho*), encoding a ligand-processing protease in the EGFR pathway, is necessary in *D. melanogaster* both for DV axis formation as well as for correct patterning of the eggshell [89] (further references in Additional file 1). Although further study is required, at present it thus seems that EGF signalling either does not play a significant role in *P. aegeria* during oogenesis or a highly divergent one. This will be discussed further in the next section.

**Table 6:** Follicle cell gene expression and border cell migration


*capping protein beta* (*cpb)*	Y	*innexin 3* (*inx3)*	Y
*hepatocyte growth factor regulated tyrosine kinase substrate* (*hrs)*	Y	*zero population growth* (*inx4; zpg)*	Y
*Calpain-B* (*CalpB)*	N	*crumbs* (*crb)*	Y
*big brain* (*bib)*	N	*stardust;* weakly similar to *maguk p55 subfamily member 5* (*sdt; std)*	Y
*brainiac* (*brn)*	Y	*quit* (*qui)*	N
*mastermind* (*mam)*	N	*dual-specificity a-kinase anchor protein spoonbill;* CG3249*;* homologous to *akap149* (*spoon; yu)*	N
*neuralized* (*neur)*	Y	*lethal (2) giant larvae* (*lgl)*	Y
*derailed* (*drl; lio)*	N	*myosin light chain 2;* similar to *Bombyx mori myosin regulatory light chain 2* (*mlc-2)*	Y
*delta* (*dl)*	Y	*deep orange; Vacuolar sorting protein 18* (*dor; Vps18)*	Y
*notch; abruptex (ax), split (spl)* (*N)*	Y	*Vacuolar protein sorting 9; sprint; rab GDP/GTP exchange factor (gef)* (*Vps9; spri)*	Y
*presenilin* (*psn)*	Y	*twinfilin* (*twf)*	Y
*nicastrin* (*nct)*	Y	*toucan* (*toc)*	Y
*gamma-secretase subunit aph-1; anterior pharynx defective 1; presenilin-stabilization factor* (*aph1)*	Y	*abrupt* (*ab)*	N
*presenilin enhancer* (*pen-2)*	Y	*taiman/ p160 coactivator fisc* (*DAIB1; tai)*	Y
*strawberry notch* (*sno)*	Y	*puckered; hearty;* similar to *dual specificity phosphatase 10* (*puc; hrt)*	N
*notchless* (*nle)*	Y	*misshapen; traf2 and nck interacting kinase;* homolog of *serine/threonine-protein kinase mig-15 (c. elegans)* (*msn; tnik)*	Y
*cut;* similar to *CCAAT displacement protein;* similar to *homeobox protein cut* (*ct; cux)*	N	*fusilli; e(cacte10)7* (*fus)*	Y
*fringe* (*fng)*	Y	*dribble; krr1 small subunit processome component homolog* (*dbe)*	Y
*bunched; shortsighted* (*bun)*	Y	*kuzbanian;* similar to *disintegrin and metalloproteinase domain-containing protein 10* (*kuz)*	Y
*dodo;* similar to *Bombyx mori rotamase pin1* (*dod)*	Y	*tie; tie-like receptor tyrosine kinase* (*tie)*	N
*Broad-Complex core protein isoform 6* (*br; Br-C)*	Y	*fk506-binding protein (fkbp13)* (*fkbp13)*	Y
*zinc finger and BTB domain-containing protein* weak homology to *Broad-Complex core protein isoforms 1, 2, 3, 4, 5* (*br; Br-C)*	Y	*m6; myelin protolipid* (*m6)*	Y
*daughterless* (*da)*	Y	*tanc2-like rolling pebbles; antisocial* (*ants; rols)*	Y
*ets at 97D; tiny eggs* (*ets97D; tny)*	N	*amphiphysin; bridging integrator* (*damph)*	Y
*pointed;* similar to *protein c-ets1* (*pnt; D-ets-1)*	N	*fasciclin II* (*fas2)*	N
*dystroglycan* (*dg)*	Y	*semaphorin; fasciclin-IV* (*fas4; sema-1a)*	Y
*discs lost; tight junction pdz protein patj* (*dlt)*	Y	*kayak* (*kay; fos)*	Y
*filamin; cheerio* (*fln; cher)*	Y	*src homology 2, ankyrin repeat, tyrosine kinase* (*shark)*	Y
*jitterbug; filamin-related* (*jbug)*	Y	*bullwinkle* (*bwk)*	N
*leukocyte-antigen-related-like; tyrosine-protein phosphatase lar* (*lar)*	N	*basket; jun amino terminal kinase (djnk); c-jun nh2-terminal kinase* (*bsk)*	Y
*discs large* (*dlg1)*	Y	*Cad74A* (*Cad74A)*	N
*scribble(d)* (*scrib)*	Y	*locomotion defects; regulator of g protein signaling (rgs)* (*loco)*	Y
*singed* (*sn)*	Y	*blistered; serum response factor; pruned* (*bs; serf)*	N
*slow border cells;* homologous to *Bombyx C/EBP* (*slbo; bmC/EBP)*	Y	*calmodulin-binding protein related to a rab3 gdp/gtp exchange protein;* weakly similar to *denn domain-containing protein 4c* (*crag)*	Y
*midline fasciclin* (*mfas)*	N	*G protein-coupled receptor kinase 1;* similar to *beta-adrenergic receptor kinase 2* (*Gprk1)*	Y
*brinker* (*brk)*	Y	*G protein-coupled receptor kinase 2;* similar to *beta-adrenergic receptor kinase 1* (*Gprk2)*	Y
*egf-r; torpedo; der* (*egfr; der)*	Y?	*rutabaga;* similar to *ca(2+)/calmodulin-responsive adenylate cyclase;* similar to *adenylate cyclase 1* (*rut)*	Y
*rhomboid-1; rhomboid; veinlet* (*rho)*	N	*dunce; cAMP-specific 3',5'-cyclic phosphodiesterase* (*dnc)*	Y
*spitz* (*spi); spitz/keren*-like	Y	*jun related antigen* (*jra)*	Y
ovarian serine protease encoding *nudel* (*ndl)*	Y	*myocardin-related transcription factor* (*mrtf)*	Y
*kekkon-1* (*kek1)*	N	similar to *rolling stone* (*rost)*	Y
*vein (*similar to a vertebrate *neuregulin)* (*vn)*	N	*jing* (*jing)*	N
*argos* (*aos)*	Y	*yan; anterior open;* similar to *ets DNA-binding protein pokkuri* (*aop)*	Y
*18 wheeler* (*18w)*	Y	*adherens junction protein p120; armadillo repeat protein; catenin delta;* CG17484 (*p120ctn)*	Y
*hopscotch* (*hop; jak)*	N	*G protein sα 60a; G protein alpha s subunit GS1 (Bombyx mori)* (*G-salpha60a)*	N
*star; asteroid* (*S)*	N	*protein tyrosine phosphatase 99a* (*ptp99a)*	N
*ran-binding protein m (ranbpm )*	Y	*diacyl glycerol kinase ϵ* (*dgkϵ)*	N
*PDGF- and VEGF-receptor related* (*PVR)*	Y	*ovary protein-29kD* (*op29)*	N
*innexin 2* (*inx2)*	Y		

Genes identified mainly from the *Drosophila melanogaster* literature acting in follicle cells early and late and promoting their motility such as border cell migration (and in *Drosophila* important for choriogenesis and dorsal appendage formation). Presence (Y), possible presence (Y?) or absence (N) of orthologous transcripts in the *Pararge aegeria* transcriptome is indicated.

### Genes acting early in the ovariole to establish dorsal-ventral polarity and genes promoting follicle cell motility such as border cell migration

Quite a number of genes involved in establishing DV polarity in the oocyte are also important for choriogenesis and dorsal appendage formation in *D. melanogaster* (references in Additional file 1). Apart from aforementioned *grk, pipe* was also not expressed by *P. aegeria*. Pipe plays an essential role in establishing DV polarity in *D. melanogaster* oocytes, with its expression being confined to ventral follicle cells as a result of localised EGF signalling [91]. Recently, however, it has been proposed that *pipe* is not necessary in a number of insect species studied [4] and even in *D. melanogaster* there appears to be a second mechanism in establishing DV [92] that may involve delayed induction by graded maternal Dpp signalling in the perivitelline space [93]. Whatever the mechanism employed by Lepidoptera, it is clear from *B. mori* research that the factors determining DV polarity are associated with the egg cortex [94].

Despite significant differences found in expression patterns of genes involved in EGF signalling in a number of insects, this pathway has been argued to be the ancient mechanism for establishing DV polarity in insect eggs [4]. Transcription factors that have been discussed as mediators of EGF signalling include *pointed* (*pnt*), *aop* and *capicua* (*cic*) [91]. Only the latter two were expressed by *P. aegeria* and present as maternal transcripts, but whether they play a role in establishing DV polarity remains to be investigated (Tables 6 and 7, and Additional file 2; qPCR results). The ETS transcription factor Aop also plays a role in border cell migration and does not receive input exclusively from EGF, but from a number of signalling pathways including Notch [95]. All components of the Notch signalling pathway were expressed in the ovarioles, with only *Notch* (*N*) itself not being present as maternal transcripts in the oocyte (Table 6 and Additional file 2). Maternal N transcripts are also not found in *D. melanogaster*.

**Table 7:** Dorsal ventral polarity


*cappuccino; formin 1/2* (*capu)*	Y	*maelstrom* (*mael)*	Y
*spire* (*spir)*	Y	*pipe (*encoding a sulfotransferase*)* (*pip)*	N
*cornichon* (*cni)*	Y	*okra (a spindle gene); rad54; rad54-like* (*okr; rad54)*	Y
*fs(1)k10* (*fs(1)k10)*	N	*spindle B* (*spnB)*	N
*sec61 beta* (*sec61 beta)*	Y	*spindle D* (*spnD)*	N
*mirror; iroquois-class homeodomain protein irx* (*mirr)*	N	*orb; oo18 RNA-binding protein* (*orb)*	N
*groucho; Enhancer of split m9/10* (*gro; E(spl)m9/10)*	Y	*heterogeneous nuclear RNA-binding protein 40; squid* (*sqd; hrp40)*	Y
*capicua* (*cic)*	Y	*heterogeneous nuclear ribonucleoprotein at 27c;* similar to *Bombyx mori hnrnpa/b-like 28* (*hrp48; hrb27c; hnrnpa/b-like 28)*	Y
*gurken* (*grk)*	N	*heterogeneous nuclear ribonucleoprotein at 87f;* similar to *Bombyx mori heterogeneous nuclear ribonucleoprotein a1* (*hrp36; p11)*	Y
*trailer hitch* (*tral)*	N	*transportin; importin 3, karyopherin beta 2b* (*impβ2)*	Y

Genes identified mainly from the *Drosophila melanogaster* literature acting early in the egg to establish dorsal-ventral (DV) polarity. Presence (Y) or absence (N) of orthologous transcripts in the *Pararge aegeria* transcriptome is indicated.

The Notch pathway interacts with the EGF pathway in establishing oocyte polarity in *D. melanogaster*, in particular through its effects on follicle cell differentiation at both termini of the oocyte [96]. As has been established in this study, there is only weak evidence at present for the use of the EGF pathway during *P. aegeria* oogenesis, and it is striking that the iroquois-class homeodomain protein Mirror is not expressed by *P. aegeria* (Table 7). This protein appears essential in *D. melanogaster* in integrating EGF and Notch signalling in follicle differentiation and thus establishing AP and DV polarity [97]. Apart from the EGF pathway, Notch interacts with a number of other proteins in patterning the follicle cells surrounding the oocyte, including Toucan and Daughterless (references in Additional file 1). These were expressed by *P. aegeria* (Table 6), suggesting that the Notch pathway is essential for correct patterning of the follicle cells and possibly oocyte polarity, but in *P. aegeria* it may not require an interaction with the EGF pathway. Further studies are required to establish whether butterflies have dispensed with EGF signalling and localised *pipe* expression in establishing oocyte polarity and instead rely on, for example, the Notch and Dpp pathway.

### Anterior and posterior system genes

The Lepidopteran *Bombyx mori* displays features of both short and long germ band type insects, in which *orthodenticle* (*otd*) and *cad* maternal mRNA are localised to establish the embryonic AP-axis [53]. Both were expressed during *P. aegeria* oogenesis (Table 8) and indeed were present as mRNA in the oocytes (Additional file 2; Figure 4 qPCR results for *cad*). *Bicoid* (*bcd*) is *Drosophila*-specific and although no ortholog was found to be expressed, the genes that are involved in *bcd* localisation were, including *exu* and *stau*, but not *swallow* (*swa*) (Table 8; Figure 4 qPCR results). As observed in *D. melanogaster,* transcripts for both *exu* and *stau* were also present in significant amounts in *P. aegeria* oocytes (Figure 4 qPCR results; Additional file 2) [65]. The use of *bcd* in translational repression of *cad* is unique to *Drosophila*. It is very likely that the ancestral mechanism for translational repression of *cad* is by means of the KH-domain containing protein encoded for by *mex-3*[98]. *Pararge aegeria* females expressed an ortholog of *mex-3* (Table 8). Furthermore, in *D. melanogaster, bcd* interacts with genes such as *bicoid interacting protein 3* (*bin3*), *eIF4E*, *larp1*, *polyA binding protein* (*pAbp*) and *AGO2* in order to repress *cad* translation [99]. All of these were found to be expressed in *P. aegeria*, and similarly to *D. melanogaster*[64,65], present as maternal transcripts in the oocytes (Tables 8 and 9, and Additional file 2; Figure 4 qPCR results for *AGO2*).

**Table 8:** Maternal specification of embryonic anterior-posterior axis


*bicoid* (*bcd)*	N	*bicoid-interacting protein 3* (*bin3)*	Y
*orthodenticle; Drosophila ocelliless* (*oc; otd)*	Y	*larp1* (*larp1)*	Y
*exuperantia* (*exu)*	Y	*Eukaryotic initiation factor 4E;* similar to *Bombyx mori Eukaryotic initiation factor 4E-2* (*eIF4E)*	Y
*swallow; fs(1)1502* (*swa)*	N	*argonaute 2* (*AGO2)*	Y
*maternal expression at 31B* (*me31B)*	Y	*caudal* (*cad)*	Y
*staufen* (*stau)*	Y	*hunchback* (*hb)*	N
*muscle excess 3 (mex-3)*	Y		

Genes in anterior-posterior axis specification, identified from a wide variety of insects. Presence (Y) or absence (N) of orthologous transcripts in the *Pararge aegeria* transcriptome is indicated.

**Table 9:** Maternal specification of embryonic posterior


*apontic* (*apt)*	N	*mago nashi* (*mago)*	Y
*nanos; nanos-like (*LOC100125608*)* (*nos-like)*	Y	*tsunagi/y14* (*tsu/y14)*	Y
*nanos-M* (*nos-M)*	Y	*ranshi;* similar to *zinc finger protein 195;* CG9793 (*ranshi)*	Y
*nanos-P* (*nos-P)*	N	*glorund* (*glo; p67)*	N
*nanos-O* (*nos-O)*	Y	*smaug* (*smg)*	Y
*shavenbaby; ovo* (*ovo)*	Y	*twin; CCR4 (*part of CCR4-Not complex*)* (*twin; CCR4)*	N
*armitage* (*armi)*	Y	*not1 (*part of CCR4-Not complex*)* (*Not1)*	Y
*arrest (*also known as *bruno)* (*aret/bru)*	Y	*not2 (*part of CCR4-Not complex*); Regena* (*Not2; Rga)*	Y
*lasp* (*lasp)*	Y	*not3 (*part of CCR4-Not complex*); l(2)nc136* (*Not3)*	Y
*oskar (osk)*	N	*chromatin assembly factor 1 (*part of CCR4-Not complex*);* similar to CG4236 (*caf1)*	Y
*poly(a)-binding protein* (*pAbp)*	Y	*Pop2;* similar to CG5684*; CCR4-Not transcription complex subunit 7* (*Pop2)*	Y
*Eukaryotic translation initiation factor 4AIII* (*eIF4AIII)*	Y	*hiiragi (Poly A Polymerase)* (*hrg; PAP)*	Y
*barentsz; eIF4aIII binding protein; weak localizer* (*wkl; btz)*	Y	*rabenosyn-5; rabenosyn* (*rbsn-5)*	Y
*syntaxin 1a* (*syx1a)*	Y	*ypsilon schachtel (Bombyx mori Y-box protein)* (*yps; ybp)*	Y
*moesin-like; dmoesin (ezrin, radixin, moesin gene)* (*moe; ERM1)*	Y	*ubiquitin specific protease 9; fat facets* (*faf)*	Y
*Eukaryotic translation initiation factor 4e transporter* similar to *cup* (*cup; fs(2)cup; fs(1)cup)*	Y	*hephaestus; polypyrimidine tract-binding protein; heterogeneous nuclear ribonucleoprotein I* (*heph; ptb; hnrnp I)*	Y
*Eukaryotic translation initiation factor 2α* (*eIF2alpha)*	Y	*synaptotagmin* (*syt 1; syt)*	Y
*miranda* (*mira)*	N	*synaptotagmin;* similar to *Drosophila melanogaster extended synaptotagmin 2* (*esyt2)*	Y

Genes identified mainly from the *Drosophila melanogaster* literature involved in posterior pole specification. Presence (Y) or absence (N) of orthologous transcripts in the *Pararge aegeria* transcriptome is indicated.

*Drosophila melanogaster* includes maternal *hunchback* (*hb*) transcripts into the egg, the protein of which will form an AP gradient during early embryogenesis and cooperate with Bcd to specify the anterior of the embryo, whilst being repressed at the posterior by Nos [100]. Although there is variation between insect species as to whether maternal *hb* RNA or protein is transferred to the egg, as well as in the significance of the maternal contribution to the Hb gradient for AP patterning, the transcription of *hb* during oogenesis appears conserved [5,101]. For example, although only zygotic Hb is necessary for AP patterning in the grasshopper *Schistocerca americana* embryo, maternal *hb* transcripts appear to be involved in distinguishing embryonic from extra-embryonic cells along the AP axis, whilst in *D. melanogaster* maternal and zygotic Hb are redundant for AP patterning of the embryo [101]. In *B. mori*, the *hb* transcripts detected appear to be transcribed by the zygote, not the mother [53,101]. *Pararge aegeria* also did not express *hb* during oogenesis (Table 8), suggesting that Lepidoptera, or at least Ditrysia, may have dispensed with a maternal contribution to the Hb gradient in the embryo.

*Nanos* is involved in both the differentiation of the germ plasm and posterior patterning in *D. melanogaster*[102], although these two functions can be mechanistically uncoupled [103]. Lepidopteran primordial germ cells (PGCs) develop in a midventral position and in the germ disk after blastoderm formation, not posteriorly before the blastoderm is formed as in *D. melanogaster*[54]. It is therefore unlikely in Lepidoptera that the genes involved in setting up the embryonic posterior will interact with and be dependent on the genes involved in the localisation of germline determinants, as shown to occur in *D. melanogaster*[54,60]. *Bombyx mori* contains a number of *nos* paralogs (*nos-M, -O, -P* and *–like (also called –N)*), which indeed appear to have divided up these functions [54]. Although it has been argued that *B. mori* does not have a germ plasm, the location of maternal *B. mori nos-O* transcripts in the embryo seems to correspond with where the PGCs will form [54]. These *nos* paralogs, with the exception of *nos-P*, are expressed during oogenesis in both *B. mori* and *P. aegeria,* with maternal transcripts detectable in *P. aegeria* eggs (Figure 4 qPCR results; Additional file 2 and Table 9) [53]. *Nanos-P* is primarily zygotically expressed during embryogenesis in *B. mori* and may be implicated in stabilising the embryonic AP-axis [53]. The *nos* paralogs have also been found in the monarch butterfly (*D. plexippus*) genome [50] and phylogenetic analysis of *nos* sequences shows *nos-P* to be quite different from the other paralogs (Additional file 8), suggesting it may have a different functional role.

Translational repression of *D. melanogaster nos* RNA is accomplished during oogenesis by proteins encoded by *glorund* (glo) and in the early embryo by *smaug* (*smg*) [104]. Transcripts of both are found in *D. melanogaster* oocytes [65]. A *P. aegeria* ortholog of *smg* was found, which was present as RNA in the oocyte, but not of *glo* (Table 9 and Additional file 2). Furthermore, Smg protein bound to the *nos* 3’ UTR recruits the deadenylation complex CCR4-NOT in *D. melanogaster*[105]. Rapid deadenylation leads to decay of *nos* RNA, which is essential in establishing the AP gradient of *nos* RNA [105]. Although it has been argued above that Lepidoptera in all likelihood do not use *nos* paralogs during oogenesis in establishing the posterior, *P. aegeria* does express all the genes that encode proteins that form this complex, despite the absence of an obvious ortholog for *twin/CCR4* (Table 9). In *D. melanogaster* it is the germ plasm protein Oskar (Osk) that prevents rapid deadenylation at the posterior pole, establishing *nos* as a posterior defining gene [105]. Ditrysia appear not to possess an *osk* ortholog [3], which could be another reason why the identified *nos* paralogs may not being involved in AP axis formation during oogenesis. Indeed, *P. aegeria* also does not possess an ortholog of *osk* (Table 9; unpublished *P. aegeria* genome).

### Germ plasm, polar granules, nuage and p-bodies

Although a germ plasm type structure has been identified cytologically in the moth *Pectinophora gossypiella*[2], it is not clear whether Lepidoptera possess a proper germ plasm as they lack *osk*, which has been argued to have been co-opted as the essential gene in germ plasm formation in holometabolous insects [1,3]. *Pararge aegeria* may not possess an *osk* ortholog, but it does express two genes, which in *D. melanogaster* silence *osk* translationally during oogenesis; *bruno*[106] and *cup*[107] (Table 9 and Additional file 1). It should be noted, however, that these genes are expressed in a number of functional contexts during oogenesis in *D. melanogaster* (e.g. cell cycle regulation; references in Additional file 1). As part of the germ plasm, Oskar induces polar (or germ) granule formation and in doing so interacts with a number of genes that characterise these polar granules, in particular *tudor* (*tud*), *vasa* (*vas*) and *valois* (*vls*) [3,103]. Only *valois* (*vls*) could not be found in the *P. aegeria* transcriptome (Tables 9 and 10).

**Table 10:** Ovarian nuage and piRNA pathway


*capsuléen; Arginine n-methyltransferase 5* (*csul; prmt5)*	Y	*tejas;* similar to *tudor domain containing 5* (*tej; TDRD5)*	Y
*valois* (*vls)*	N	*vreteno;* similar to CG4771 (*vret)*	N
*aubergine (related to eIF2c; a piwi protein)* (*aub)*	Y	similar to *tudor domain containing* CG9925 and CG9684 (*TDRD1)*	Y
ATP-*dependent helicase; cap; belle* (*cap; bel)*	Y	similar to CG8920*;* similar to *tudor domain containing 7* (*TDRD7)*	Y
*cutoff* (*cuff)*	N	*homeless; fs(3); spindle E;* similar to *tudor domain containing 9* (*hls; spnE; TDRD9)*	Y
*squash* (*squ)*	N	CG14303*;* similar to *tudor domain containing 4* (*TDRD4)*	N
*piwi-like protein; argonaute 3* (*AGO3; siwi)*	Y	*tudor-SN* (*tudor-SN)*	Y
*zucchini* (*zuc)*	N	*Brother of Yb;* CG11133 (*BoYb)*	N
*tudor;* similar to *tudor domain containing 6* (*tud)*	Y	*Sister of Yb;* CG31755 (*SoYb)*	N
*krimper* (*mtc; krimp)*	N		

Ovarian nuage and piRNA pathway genes identified mainly from the *Drosophila melanogaster* literature. Presence (Y) or absence (N) of orthologous transcripts in the *Pararge aegeria* transcriptome is indicated.

Both the ovarian nuage, an electron-dense perinuclear structure found predominantly in nurse cells [108], and polar granules are characterised by a number of the same genes, including *tud*, *vas* and *vls* (references in Additional file 1). The nuage appears not only to play a role in protecting germline cells against the expression of selfish genetic elements in the majority of animals, but also in establishing the polar granules in *D. melanogaster*[108,109]. It is therefore not surprising that PIWI proteins and their bound PIWI-interacting RNAs (piRNAs) have been identified as important for both nuage and polar granule formation [109,110]. Many of these genes encode TUDOR-domain containing proteins and seem to evolve rapidly making it difficult to find orthologs outside *Drosophila*; e.g. *vreteno* (*vret*), *Brother of Yb* (*BoYb*) and *Sister of Yb* (*SoYb*) [110]. Indeed, no orthologs of these genes could be found in the *P. aegeria* transcriptome (Table 10). Other genes encoding TUDOR-domain containing proteins seem more conserved, such as *TDRD1*, *tejas* (*TDRD5*), *TDRD7* and *spindle E/homeless* (*TDRD9*) [3,110] and these were expressed by *P. aegeria* (Table 10). What is interesting about *TDRD7* is that it shares the OST-HTH/LOTUS functional domain with *osk*[1,3]. It is likely that this domain is involved in RNA binding and thus for regulating mRNA translation and/or localisation in germ cell development [111].

There are three genes that encode PIWI proteins; *piwi*, *aubergine* (*aub*) and *argonaute 3* (AGO3) [112]. All three were expressed during oogenesis by *P. aegeria* (Figure 4 qPCR results; Tables 1 and 10). Piwi also plays an essential role in the *D. melanogaster* germarium and is thus involved in the establishment, maintainance and renewal of germline stem cells [113]. Furthermore, mutations in *D. melanogaster* piRNA (Piwi-interacting RNA) pathway genes often disrupt the axes of the developing oocyte, through their effects on the microtubule cytoskeleton; for example *maelstrom* (*mael*), *zucchini* (*zuc*) and *squash* (*squ*) affect DV polarity [114,115]. The latter two also interact with *aub* in *D. melanogaster* in silencing *osk* translation during oogenesis [115]. Similarly, the RNAi pathway gene *armitage* (*armi*) affects axis formation and is involved in *osk* translational silencing in *D. melanogaster*[107]. Neither *zuc* nor *squ* was found in the *P. aegeria* transcriptome, but *mael* and *armi* were (Tables 7 and 10).

Ovarian processing bodies (i.e. P-bodies) are aggregates of translationally inactive ribonucleoproteins (RNPs). In *D. melanogaster* these can be found in nurse cells, but also appear to be involved in compartmentalisation of mRNA decay and translation repression, for example of *osk*[116,117]. With the exception of *EDC4/Ge-1* and *pacman* (*pcm*), genes that encode the essential components of P-bodies were expressed in *P. aegeria* (described in the context of oogenesis or otherwise, Table 11 and references in Additional file 1). RNA of P-body components, for example *Dcp1*, are also transferred to oocytes during *D. melanogaster* oogenesis and are necessary for early embryogenesis [116]. This was also observed in *P. aegeria* (Additional file 2).

**Table 11:** Ovarian processing bodies


*Nonsense-mediated mRNA 3* (*Nmd3)*	Y	*telomerase-binding protein est1a;* similar to *smg6 homolog, nonsense mediated mRNA decay factor* (*smg6)*	Y
*regulator of nonsense transcripts 1; nonsense mRNA reducing factor 1; up-frameshift suppressor 1 homolog* (*rent1; norf1; Upf1)*	Y	*decapping protein 1* (*Dcp1)*	Y
similar to *Upf2 regulator of nonsense transcripts homolog* (*Upf2)*	Y	*decapping protein 2* (*Dcp2)*	Y
similar to *Bombyx mori Upf3 regulator of nonsense transcripts-like protein B* (*Upf3)*	Y	*pacman; 5'-3' exoribonuclease 1* (*XRN1; pcm)*	N
*no-on-and-no-off-transient C* (*smg1)*	Y	*EDC4; Ge-1* (*Ge-1)*	N
*smg5* (*smg5)*	Y		

Ovarian processing bodies genes identified mainly from the *Drosophila melanogaster* literature. Presence (Y) or absence (N) of orthologous transcripts in the *Pararge aegeria* transcriptome is indicated.

Once the germ plasm has been established at the posterior in *D. melanogaster*, a number of (late-acting) maternal-effect genes are essential in germline formation during early embryogenesis ([118]; further references in Additional file 1). *Pararge aegeria* females do express similar genes to the fruit fly, including genes associated traditionally with *D. melanogaster* pole plasm, such as *arrest/bruno* (*aret*) and *imp*[119]. However, there are some notable exceptions, the most significant of which are *germ cell-less* (*gcl*) and *polar granule component* (*pgc*) (Tables 12, and 13, and Additional file 1). These genes are essential in *D. melanogaster*, but there are no known *pgc* orthologs outside the genus *Drosophila*. Although orthologs can be found for *gcl* even in vertebrates, none can be found in genomic databases for the Lepidoptera, including the new data presented here. The gene *wunen* (*wun*) is involved in germ cell migration in *D. melanogaster* embryos (references in Additional file 1). *Pararge aegeria* females also include *wun* transcripts in the oocyte (Table 13 and Additional file 1).

**Table 12:** Germ plasm formation and germline viability


*rab-protein 11* (*rab11)*	Y	*germ cell-less* (*gcl)*	N
*rab-protein 5* (*rab5)*	Y	*stambha a;* CG8739*; protein efr3 homolog b; rolling blackout* (*cmp44e ; stma)*	Y
*skittles; pip5k (*type 1*)* (*pip5k)*	Y	*myoglianin* (*myo; myg )*	N
*rap1 GTPase activating protein* (*rapgap)*	Y	*mitochondrial small ribosomal RNA* (*mtsrRNA; 12s rRNA)*	N

Genes identified mainly from the *Drosophila melanogaster* literature involved in germ plasm (i.e. pole plasm in *D. melanogaster*) formation - Control of endocytosis in germline and germline viability. Presence (Y) or absence (N) of orthologous transcripts in the *Pararge aegeria* transcriptome is indicated.

**Table 13:** Maternal effect genes


*abstrakt* (*abs)*	Y	*jafrac1; thioredoxin peroxidase 1; thiol peroxiredoxin* (*jafrac1; dpx-4783)*	Y
*terribly reduced optic lobes; perlecan; zeste-white 1* (*trol; pcan; zw1)*	Y	*deadhead; thioredoxin* (*trx-1; trx)*	N
*TBC1 domain family member 1;* weakly similar to *Drosophila melanogaster pollux* (*plx)*	Y	*thioredoxin-like;* similar to *Bombyx mori thioredoxin (trxl)*	Y
*out at first* (*oaf)*	Y	*thioredoxin-2;* similar to *Bombyx mori thioredoxin-like* (*trx2)*	Y
*extra macrochaetae* (*emc)*	Y	*yema gene 2.8* (*yemg2.8)*	N
*wings up a; troponin 1* (*tn1; tpn1; wupa)*	Y	*yema gene 3.4* (*yemg3.4)*	N
*troponin c* (*tpnc; tnc47d)*	Y	*yema gene 3a* (*yemg3a)*	N
*troponin t; wings up b; upheld* (*tpnt; wupb)*	Y	*yema gene 3b* (*yemg3b)*	N
*tropomyosin 1 or 2* (*tm1; tm2)*	Y	*yema gene 3c* (*yemg3c)*	N
*alcohol dehydrogenase* (*adh)*	Y	*yema gene 4* (*yemg4)*	N
*polar granule component* (*pgc)*	N	*yema gene 9.5* (*yemg9.5)*	N
*type III alcohol dehydrogenase; iron-containing dehydrogenase* (*t3dh; adhfe1)*	Y	*yemanuclein α;* similar to *ubinuclein* (*yemalpha)*	Y
*plutonium* (*plu)*	N	*wings down; pourquoi-pas; serendipity-cognate* (*pqp; wdn; sry-h1)*	Y
*pan gu* (*png)*	N	*serendipity delta; serendipity δ* (*sry-delta)*	Y
*giant nuclei* (*gnu)*	N	*serendipity α* (*sry-alpha)*	Y
*germ cell guidance factor wunen; phosphatidate phosphatase* (*wun)*	Y	*heat shock RNA ω* (*hsr-omega)*	N
*receptor for activated protein kinase c rack 1* (*rack1)*	Y	*tiovivo; nebbish; kinesin-like protein at 38b* (*klp38b; tio; neb)*	N
*shuttle craft; transcriptional repressor nf-x1* (*stc)*	Y	*GTP-binding protein alpha-subunit; G protein α 73b* (*Galpha73b)*	N
*muscleblind* (*mbl)*	Y	*Guanine nucleotide-binding protein G(I) subunit* (*GalphaI)*	N
*grainyhead* (*NTF-1; grh)*	Y	*G protein β-subunit 13f; heterotrimeric guanine nucleotide-binding protein beta subunit (Bombyx mori)* (*Gbeta13f)*	Y
*dorsal (Drosophila); embryonic polarity protein dorsal (Bombyx* - 2 isoforms*)* (*dl)*	Y	*G protein γ 1;* CG8261 (*Ggamma1; bro4 )*	Y
*dorsal switch protein* (*dsp1; ssrp2)*	Y	*protein tyrosine phosphatase 69d* (*ptp69d)*	N
*tosca; exonuclease 1* (*tos)*	Y	similar to *serine/threonine kinase pelle;* homologous to *irak-4* (*pll)*	Y
*Darkener of apricot; dual specificity protein kinase clk2* (*Doa)*	Y	*gastrulation-defective* (*gd)*	Y
*clipper; cleavage and polyadenylation specific factor 4* (*clp; cpsf30)*	Y	*short gastrulation* (*sog )*	N
*vrille* (*vri; jf23)*	Y	*tube* (*tub)*	Y
*absent md neurons and olfactory sensilla* (*amos)*	N	similar to *Bombyx mori spätzle 1* (*spz)*	Y
*baboon; activin receptor type 1* (*ATR1)*	Y	*weckle* (*wek)*	N
*eyelid; osa* (*eld; osa)*	Y	*cactus* (*cact)*	Y
*gonadal* (*gdl)*	Y	*BzArgOEtase (Bombyx mori);* similar to *easter; clip-domain serine protease subfamily B* (*ea)*	Y
*éclair; transmembrane emp24 protein transport domain containing 9* (*eca)*	Y	similar to *snake (Drosophila melanogaster);* similar to *serine protease 21 (Manduca sexta); clip-domain serine protease subfamily c* (*snk)*	Y
*baiser; transmembrane trafficking protein* (*bai)*	Y	*toll* (*tl)*	N
*logjam* (*loj)*	Y	similar to *Bombyx mori calpain;* weakly similar to *Drosophila melanogaster Calpain-A* (*CalpA)*	Y
*bancal; (*similar to*) heterogeneous nuclear ribonucleoprotein K* (*hrb57A; q18)*	Y	similar to *brokenheart;* similar to *G protein oalpha 47A; Guanine nucleotide-binding protein G(o) subunit alpha; G protein alpha subunit go* (*G-olpha47A)*	Y
*maternal transcript 89BA* (*mat89BA)*	N	*concertina; Guanine nucleotide-binding protein subunit alpha-13* (*conc)*	N
*asunder; maternal transcript 89BB* (*mat89BB; asun)*	Y	*SNF1A/AMP-activated protein kinase - alpha subunit* (*SNF1-AMPK-alpha subunit)*	Y
*diadenosine tetraphosphatase;* similar to *bis(5-nucleosyl)-tetraphosphatase* (*datp)*	Y	*SNF1A/AMP-activated protein kinase - beta subunit* (*SNF1-AMPK-beta subunit)*	Y
*dopa decarboxylase; aromatic-l-amino-acid decarboxylase* (*ddc)*	Y	*SNF1A/AMP-activated protein kinase - gamma subunit* (*SNF1-AMPK-gamma subunit)*	Y
*hairless* (*h)*	N	*IGF-II mRNA-binding protein* (*imp; MRE11)*	Y
*suppressor of hairless; j kappa-recombination signal-binding protein* (*su(h))*	Y	similar to *G protein alpha q; G protein α49b* (*Gαq; Galpha49b)*	Y
*transcription termination factor lodestar; horka* (*horka; ids)*	Y	*map kinase activated protein-kinase-2* (*mk2; MAPK-ak2)*	Y
*raspberry; inosine monophosphate dehydrogenase* (*ras)*	Y	*ptb-associated splicing factor;* weakly similar to *Drosophila no on or off transient a* (*psf)*	Y
*misato* (*mst; lb20)*	Y	*palmitoyl-protein thioesterase 1* (*ppt1)*	Y
*peanut;* similar to *septin 7* (*pnut)*	Y	*abl tyrosine kinase* (*abl)*	Y
*septin 1; innocent bystander* (*sep-1; iby)*	Y	*Abelson interacting protein* (*Abi)*	Y
*septin 2* (*sep-2)*	Y	*wing blister;* homologous to *laminin alpha 2 (merosin)* (*wb)*	N
*septin and tuftelin interacting protein; elongator complex protein 2; septin interacting protein 1* (*stip)*	Y	*supervillin;* CG33232 (*svil)*	Y
*kurz;* similar to ATP-*dependent RNA helicase dhx37* (*kz)*	Y	*cyclope; cytochrome c oxidase subunit vic* (*cype)*	Y
*pebble* (*pbl)*	Y	*la autoantigen-like* (*la)*	Y
*numb* (*numb; nb)*	Y	*tramtrack* (*ttk; ttk69)*	Y
*catalase* (*cat)*	Y	*high mobility group protein b1; dorsal switch protein 1* (*HMGb1; dsp1; ssrp2)*	Y
*superoxide dismutase* (*sod1; csod; cu/znsod)*	Y	*zinc finger protein 43c* (*az2)*	N
*disc proliferation abnormal* (*mcm4; dpa)*	Y	*maverick* (*mav)*	N
*Fragile x mental retardation 1* (*Fmr1)*	Y	*shibire; dynamin* (*shi; dyn)*	Y
*female sterile (2) ketel; karyopherin beta 1; importin β* (*ketel; imp-beta)*	Y	*protein o-fucosyltransferase 1;* similar to *Bombyx mori fut12 gene* (*pofut1)*	Y
*karyopherin beta 3* (*karyβ3)*	Y	*protein o-fucosyltransferase 2;* similar to *Bombyx mori fut13 gene* (*pofut2)*	Y
*cas/cse1 segregation protein; export karyopherin cas/cse1p* (*cas)*	Y	similar to *bloated tubules; sodium/chloride dependent transporter* (*blot)*	Y
*importin alpha 1; karyopherin α1* (*imp alpha 1)*	Y	*gastrulation defective protein 1 homolog;* CG5543*;* similar to *WD repeat-containing 70 protein* (CG5543)	Y
*importin alpha 2; karyopherin α2; pendulin* (*imp alpha 2)*	Y	*high mobility group protein 20a* (*HMG20a)*	Y
*importin alpha 3; karyopherin α3* (*imp alpha 3)*	Y	*high mobility group box-containing protein 4; hmg-box protein hmg2l1* (*HMGx4)*	Y
*imaginal disc growth factor 1* (*idgf; idgf1)*	Y	*calcium atpase at 60a; sarcoplasmic/endoplasmic reticulum calcium atpase* (*serca; kum; dserca; cap60a)*	Y
*imaginal disc growth factor 2* (*idgf2)*	N	*dacapo* (*chakra; dap)*	N
*imaginal disc growth factor 3* (*idgf3)*	N	*liprin-α* (*liprin-a)*	N
*imaginal disc growth factor 4* (*idgf4)*	N	*mitochondrial acyl carrier protein 1; nadh-ubiquinone oxidoreductase acyl carrier protein* (*mtacp1)*	N
*kinesin-like protein at 61f; urchin; kinesin-like protein klp2 (in Bombyx mori)* (*klp61f; klp2)*	Y	*mitochondrial assembly regulatory factor; mitofusin* (*marf; mfn; mfn2)*	Y
*puromycin sensitive aminopeptidase* (*psa)*	Y	*ripped pocket; gonad-specific amiloride-sensitive sodium channel 1* (*rpk; gnac1)*	N
*cask ortholog; calmodulin-dependent kinase* (*caki; cmg; camguk)*	Y	*kurtz;* similar to *beta-arrestin 1* (*krz)*	Y
*signal transducing adaptor molecule* (*stam)*	Y	*ubiquitin carboxy-terminal hydrolase;* CG4265 (*uch)*	Y
*histone acetyltransferase kat2b; histone acetyltransferase pcaf; general control of amino acid synthesis protein 5-like 2* (*pcaf; gcn5)*	Y	*lark* (*lark)*	Y
*ada2b* (*ada2b)*	Y	*semaphorin-5c* (*sema-5c)*	N
*s-adenosyl-methyl transferase mraw;* CG14683 (mraw)	Y	*semaphorin 1b* (*sema-1b )*	N
*c-terminal binding protein; hairy-interacting protein;* similar to *2-hydroxyacid dehydrogenase* (*ctbp)*	Y	*selenophosphate synthetase 1; selenide, water dikinase* (*sps1 )*	Y
*reticulated* (*ret)*	N	*sodium/potassium exchanging and transporting ATPase subunit beta 1 nervana 1* (*nrv1)*	Y
*furin 1;* similar to *convertase subtilisin/kexin;* similar to *furin-like convetase* (*fur1 )*	N	*sodium/potassium exchanging and transporting ATPase subunit beta 2 nervana 2* (*nrv2)*	Y
*windbeutel; thioredoxin-like motif containing gene* (*wbl)*	Y		

Maternal transcripts; Maternal effect genes identified mainly from the *Drosophila melanogaster* literature encoding various types of proteins, including enzymes, needed for early embryogenesis and germ cell formation. Presence (Y) or absence (N) of orthologous transcripts in the *Pararge aegeria* transcriptome is indicated.

### Maternal transcripts involved in regulating early embryogenesis – dorsal-ventral patterning of the embryo and early neurogenesis

*Drosophila melanogaster* uses an elaborate network of genes to pattern the DV axis during embryogenesis on the basis of the oocyte polarity established during oogenesis (discussed in [89,120]; further references in Additional file 1). As discussed elsewhere in this paper, the two genes essential for establishing DV polarity in *D. melanogaster* oocytes, *grk* and *pipe* (the latter of which is repressed dorsally [120]), were absent from the *P. aegeria* transcriptome. The genes that are subsequently involved in establishing the ventral side of the *D. melanogaster* embryo are co-opted from the Toll innate immune defense pathway (including a serine protease cascade [121]). A similar cascade has been described in *T. castaneum*, but at present it is not known whether it is restricted to the ventral perivitelline space [4]. This protease cascade and associated (ventral) genes were also expressed in *P. aegeria*, but at present it is unclear in which functional context they are used. These genes include; *windbeutel* (*wind*), *nudel* (*ndl*), *gastrulation defective* (*gd*), *snake* (*snk*), *easter* (*ea*), *spn27A*, *spz*, *tube* (*tub*) and *pelle* (*pll*) (Tables 7 and 13; Additional files 1 and 2). No orthologs for the zinc-finger gene *weckle* (*wek*) have yet been found outside *Drosophila*, and *wek* was also not found in *P. aegeria* (Table 13). In *D. melanogaster*, Toll receptor protein accumulates during the embryonic syncytial stage prior to nuclear migration, and is activated ventrally as the result of a serine/protease cascade (references in Additional file 1). The Toll-like receptor expressed by *P. aegeria* during oogenesis was found to be an ortholog of *18 wheeler* (*18w*), rather than *toll* (*tl*) (Tables 6 and 13). In *D. melanogaster 18w* is involved in dorsal appendage formation and follicle cell migration [122], and DV patterning [89]. While *P. aegeria* eggs do not have dorsal appendages, *18w* may be involved in DV patterning. In *D. melanogaster 18w* expression in relation to eggshell patterning, and thus DV polarity, is dependent on input from Dpp and EGF signalling pathways [89]. As discussed elsewhere in the paper, there is not much evidence for EGF signalling in *P. aegeria* oogenesis, but there is for Dpp signalling (e.g. Figure 4 qPCR results). Furthermore, analyses of Toll receptors have shown that *B. mori tl* and *18w* sequences were more similar to each other, than to *D. melanogaster toll*[123]. It thus remains to be investigated exactly which functional role *18w* fulfils during oogenesis in Lepidoptera.

*Pararge aegeria* did express *cactus* (*cact*) and *dorsal* (*dl*) (Table 13). Dorsal protein is distributed evenly in a *D. melanogaster* embryo, but a gradient in the uptake of Dorsal protein into the nucleus (high on the ventral side) is essential for subsequent DV patterning in the *D. melanogaster* embryo. Dorsal protein activates some genes, whilst repressing others along the DV axis [120,124]. While there are some differences in detail, the gene regulatory network underlying embryonic DV patterning is largely conserved in all insects [4]. The Dorsal protein represses *dpp* ventrally and the protein encoded by *grainyhead* (*NTF-1*/*grh*) acts as co-repressor [124]. RNA of *grh* is deposited maternally into the oocyte to be translated and used ventrally during embryogenesis [124]. Repression of *dpp* by a Dorsal gradient does not, however, occur in *T. casteneum*[4]. A high concentration of Dpp will eventually be restricted to the dorsal side of the *D. melanogaster* embryo and its concentration is further restricted ventro-laterally by Short gastrulation (Sog), which in *D. melanogaster* may also be maternally provided [120]. Rather interestingly, this antagonistic interaction between Dpp and Sog may already be employed during oogenesis for the establishment of DV polarity in the oocyte [125]. The *vrille* (*vri*) gene encodes a Bzip transcription factor that interacts in *D. melanogaster* with Dpp signalling, acting as dominant maternal enhancers of embryonic DV patterning defects caused by *ea* and *dpp* mutations [126]. Two P24 proteins encoded by *eclair* (*eca*) and *baiser* (*bai*) are essential for the activity of maternal Tkv, a type I Dpp receptor [127]. *Pararge aegeria* females did transfer maternal transcripts of *grh*, *dpp, tkv, eca, bai* and *vri* into the oocyte, but did not express *sog* maternally (Figure 4 qPCR results; Tables 3 and 13; Additional files 1 and 2).

*Drosophila melanogaster* females express a group of genes called the *yema* genes (*yema 2.8*, *3.4*, *3a*, *3b*, *3c*, *4* and *9.5*) during oogenesis, with most of them displaying strict maternal expression. This may be of importance in the development of the central nervous system of the embryo [128]. However, the exact functional roles of the *yema* genes are not known and there are no orthologs outside *Drosophila*[128]. No orthologs were found for these genes in the *P. aegeria* transcriptome (Table 13 and Additional file 1). *Pararge aegeria* females did, however, express a number of other genes that are implicated in embryonic brain development or in general in the nervous system; e.g. *neuralized* (*neu*), *elav*, *brainiac* (*brn*), *Fmr1*, *brain tumor* (*brat*), *mnb*, and *terribly reduced optic lobes* (*trol*) (Tables 3, 6 and 13; Additional file 1). Of these, *mnb* and *elav* have not been explicitly studied in the context of oogenesis (references in Additional file 1). Although maternal transcripts of these genes may play a role in embryonic neural development in *D. melanogaster*, these genes appear to be important in establishing polarity of the oocyte and its differentiation during oogenesis (references in Additional file 1). The expressions of three of these were further investigated by means of qPCR: *elav*, *Fmr1* and the serine/protease encoding *mnb* (Figure 4 qPCR results). To date, of these three, only *Fmr1* has been described as present in *D. melanogaster* oocytes, but *elav*, *Fmr1* and *mnb* were all found in *P. aegeria* oocytes (Figure 4 qPCR results) [129]. Compared to the ovaries, the amount of *elav* and *Fmr1* transcripts in the oocytes was quite low (Figure 4 qPCR results; Additional file 2), suggesting they are important during oogenesis. Whether these genes play a role of significance in establishing oocyte polarity in *P. aegeria* needs to be investigated.

### Terminal genes

The Torso receptor tyrosine kinase (RTK) pathway has been implicated in a number of different processes during *D. melanogaster* oogenesis, including vitelline membrane (or envelope) biogenesis [130] and in particular terminal region specification [131]. The maternal-effect gene *torso* (*tor*) encodes a receptor whose ligand is most probably encoded for by *trunk* (*trk*). Furthermore, the protein encoded by *torsolike* (*tsl*) plays a role upstream of *trk* in activating the Tor receptor in a localised manner, and is thought to be essential for terminal specification [132]. Although both *tor* and *tsl* are involved in terminal specification in *T. castaneum*, different tissues are patterned and Torso signalling plays a role in defining the posterior growth zone during embryogenesis in this short germband insect [133]. Torso signalling is by no means the default mechanism for terminal specification, as the honey bee (*Apis mellifera*) has the gene *tsl*, but not *tor* and *trk* in its genome [134]. The honey bee seems to rely on other mechanisms for terminal specification [135]. *Pararge aegeria* does not express clear orthologs of either *tor* or *trk* during oogenesis, but does express *tsl* (Table 14). *Bombyx mori* does have a RTK in its genome (BGIBMGA003976), which shows similarity to *torso,* as well as to *tie-like* and *Cad96Ca*. *Pararge aegeria* did not express *tie-like* (Table 6), but did express *Cad96Ca* (PACG18092; Additional file 2). This transcript was not present in oocytes and was found only in the ovarioles (Additional file 2). Furthermore, a TBLASTN of the putative *B. mori tor* against the *P. aegeria* transcriptome showed that transcript PACG7078 (complete CDS; Additional file 2) was similar (E-value= 5.0 E^-50^), although it had greater similarity to the receptor tyrosine kinase *Fps85D* than to *tor.* This transcript is present in both *P. aegeria* oocytes and ovarioles, but its role in oogenesis has not been described in the literature. It is clear that *P. aegeria* uses RTK signalling during oogenesis and that the sequences of its ligands and receptors have diverged from those of other insects. However, at present it is unclear in which functional context RTK signalling takes place.

**Table 14:** Terminal specification


*corkscrew;* similar to *protein tyrosine phosphatase, non-receptor type 11* (*csw; ptpn11)*	Y	*raf; raf1; pole hole; raf kinase; effector of ras* (*raf; raf1; phl)*	Y
*dead ringer* (*dri)*	Y	*signal transducer and activator (stat)* (*stat; stat92e)*	Y
*torso* (*tor)*	N	*rolled; map kinase (MAPK)* (*rl; MAPK; erk)*	Y
*torsolike* (*tsl)*	Y	*downstream of raf1* (*dsor1)*	N
*trunk* (*trk)*	N	*hemipterous; mitogen-activated protein kinase kinase* (*hep; MAPKK; mkk7)*	Y
*female sterile (1) homeotic; fragile-chorion membrane protein* (*fs(1)h)*	Y	*growth arrest and DNA-damage inducible 45* (*gadd45)*	N
*ras1* (*ras1; ras85d)*	Y	*shc-adaptor protein; shc-transforming protein 1; src homology 2 domain containing;* CG3715 (*shc)*	N

Genes identified mainly from the *Drosophila melanogaster* literature involved in terminal specification. Presence (Y) or absence (N) of orthologous transcripts in the *Pararge aegeria* transcriptome is indicated.

### Chromatin regulation during oogenesis, DNA replication, general transcription and maternal regulation of zygotic transcription in general

In general, the genes that encode proteins involved in chromatin remodelling, DNA replication and transcription are highly conserved across insects and often across the Metazoa in general (references in Additional file 1). A large number of these genes have been studied specifically in the context of oogenesis in *D. melanogaster* (Table 15; references in Additional 1). *Pararge aegeria* was found to express orthologs of a number of these genes (Table 15 and Additional file 1). The genes not expressed by *P. aegeria* seem to either have no clear insect orthologs outside *Drosophila*, or no such orthologs have been reported in Lepidoptera, such as *B. mori*. Genes not expressed by *P. aegeria*, but for which Lepidopteran orthologs exist include *TATA box binding protein-related factor 2* (*Trf2*), *sex combs on midleg* (*scm*), and *Arginine methyltransferase 1* and *8* (*DART1* and *DART8*, Table 15 and Additional file 1). The gene *scm* is a member of the *polycomb* group (*PcG*) and similar to *D. melanogaster polyhomeotic* (*ph-p*) gene. Both play versatile and important roles in *D. melanogaster* oogenesis, particularly in ovarian follicle formation [136,137]. *Pararge aegeria* females did express and transfer orthologs of other PcG genes into the oocyte. These include the polycomb repressive complex 1 (PRC1) genes *sex combs extra* (*sce*), *polycomb* (*ph*), *posterior sex combs* (*psc*), the PRC2 genes *extra sex combs* (*esc*), *Enhancer of zeste* (*E(z)*) and the polycomb related genes *Enhancer of polycomb* (*E(ph)*) and *additional sex combs* (*asx*) (Table 15, Additional files 1 and 2; references therein). Recently these genes have also been identified in *B. mori* embryogenesis [138]. These genes encode proteins that regulate DNA and histone methylation patterns and general chromatin remodelling. However, they also appear to be important specifically during oogenesis and embryogenesis and may be implicated in transferring gene regulatory states from one generation to the next, being regarded as candidate genes in epigenetic processes [139], with possible involvement in transgenerational effects in relation to environmental heterogeneity.

**Table 15:** Regulation of transcription and chromatin structure


*DNA polymerase α 180KD; DNA polymerase alpha catalytic subunit* (*DNApol-α180)*	Y	homolog of *regulator of chromatin condensation 2;* similar to CG9135 (*rcc2)*	Y
*RNA polymerase II transcriptional coactivator single stranded-binding protein c31a* (*ssb-c31a)*	Y	*DNA polymerase interacting tpr containing protein* (*dpit47)*	Y
*polyadenylate-binding protein 2* (*rox2; pabp2)*	Y	*DNA polymerase α (180kD)* (*DNApol-α180; pola)*	Y
*high mobility group protein; structure specific recognition protein. fact complex subunit ssrp1* (*ssrp; ssrp1)*	Y	*DNA polymerase delta* (*DNApol-delta)*	Y
similar to *Drosophila melanogaster high mobility group protein d;* similar to *Bombyx mori high mobility group protein 1b* (*HMGd; HMG1b)*	Y	*DNA polymerase ϵ* (*DNApol-ϵ; pole)*	Y
*domina; jumeau* (*jumu/dom)*	Y	similar to *DNA polymerase ϵ subunit 2* (*DNApol-ϵ; pole2)*	Y
*modulo* (*mod)*	N	similar to *DNA polymerase ϵ subunit 3* (*DNApol-ϵ; pole3)*	Y
*lysine-specific histone demethylase 1; suppressor of variegation 3–3* (*suv3-3; su(var)3-3; lsd1)*	Y	*DNA polymerase eta* (*DNApol-eta; drad30a)*	Y
*histone methyltransferase 4–20; suppressor of variegation 4–20* (*suv4-20; su(var)4-20)*	Y	*DNA polymerase iota* (*drad30b; DNApol-iota)*	Y
*Drosophila melanogaster suppressor of variegation 3–9* (*suv3-9; su(var)3-9)*	Y	*DNA polymerase zeta;* similar to *mutagen-sensitive 205; rev3-like* (*DNApol-zeta; mus205)*	Y
*pitkin(dominant)* (*ptn(d))*	N	*replication protein a1* (*rpa1)*	Y
*Eukaryotic translation initiation factor 2 gamma subunit* (*eIF2g)*	Y	*replication protein a2* (*rpa2)*	Y
*suppressor of variegation 2–10; protein inhibitor of activated stat* (*su(var)2-10; pias; zimp; zimpb;)*	Y	*replication protein a3* (*rpa3)*	Y
*eggless* (*egg; SETDB1)*	Y	*replication factor c 38kD subunit* (*rfc38)*	Y
*histone h3k9 methyltransferase dg9A* (*g9A)*	N	*(Bombyx mori) replication factor c subunit 2; rfc40* (*rfc40; bm- rfc2)*	Y
*modifier of mdg4* (*mod(mdg4); e(var)3-93d)*	Y	*(Bombyx mori) replication factor c4;* CG8142 (*bm-rfc4)*	Y
*suppressor of hairy wing* (*su(hw))*	Y	*(Bombyx mori) replication factor c (activator 1) 5; Drosophila replication factor c subunit 3* (*rfc3)*	Y
*trithorax-like* (*trl; GAGA; gaf; e(var)3; e(var)62)*	N	*germ line transcription factor 1; replication factor 1* (*rfc1; gnf1)*	Y
*brahma; SWI/SNF-related matrix-associated actin-dependent regulator of chromatin subfamily A member; transcription activator brg1* (*smarca4; brm)*	Y	*recombination repair protein 1* (*rrp1)*	Y
*marcal1; SWI/SNF-related matrix-associated actin-dependent regulator of chromatin subfamily A member* (*marcal1; smarcal1)*	Y	*rev7* (*rev7)*	N
*snf5-related 1; SWI/SNF-related matrix-associated actin-dependent regulator of chromatin subfamily B member 1* (*snr1; bap45)*	Y	*trf4-1; sigma DNA polymerase* (*trf4-1)*	Y
*brg-1 associated factor; SWI/SNF-related matrix-associated actin-dependent regulator of chromatin subfamily d member 1; brahma associated protein 60kD* (*bap60)*	Y	*topoisomerase 1; topoisomerase i* (*top1)*	Y
*dalao; brahma-associated protein 111kD; SWI/SNF-related matrix-associated actin-dependent regulator of chromatin subfamily E* (*bap111; dalao)*	Y	*topoisomerase 2; topoisomerase II* (*top2; topII)*	Y
*moira* (*mor; bap155)*	Y	*topoisomerase 3 alpha; topoisomerase III aplha* (*topIII-alpha)*	Y
*imitation swi* (*dnurf; iswi; dchrac)*	Y	*topoisomerase 3 beta; topoisomerase III beta* (*topIII-beta)*	Y
*Brahma associated protein 170kD* (*bap170)*	Y	*minichromosome maintenance 3* (*mcm3)*	Y
*Brahma associated protein 55kD* (*bap55)*	Y	*minichromosome maintenance 5* (*mcm5)*	Y
helicase *domino* (*dom)*	Y	*minichromosome maintenance 6; fs(1)k1214* (*mcm6)*	Y
*etl1 homologue; SWI/SNF-related matrix-associated actin-dependent regulator of chromatin subfamily A containing dead/h box 1* (*etl1; smarcad)*	Y	*minichromosome maintenance 7* (*mcm7)*	Y
*Enhancer of zeste* (*E(z))*	Y	*minichromosome maintenance 8; recombination-defective* (*mcm8; rec)*	Y
*extra sex combs* (*esc)*	Y	*DNA methyltransferase 2* (*mt2)*	Y
*additional sex combs* (*asx)*	Y	*poly-(adp-ribose) polymerase* (*parp)*	Y
*sex comb on midleg* (*scm)*	N	*TATA box binding protein-related factor 2* (*Trf2; tlf)*	N
*multi sex combs* (*mxc)*	N	*TATA box binding protein* (*Tbp)*	Y
*polyhomeotic* (*ph-p)*	N	*tbp-associated factor 250kD* (*taf250; taf1)*	Y
*sex combs extra;* similar to *E3 ubiquitin-protein ligase ring1 (Bombyx mori)* (*sce; dring)*	Y	*trithorax-related* (*trr)*	Y
*polycomb* (*ph)*	Y	*supercoiling factor* (*scf; dcb-45)*	Y
*Enhancer of polycomb* (*E(pc))*	Y	*bx42; ski-interacting protein* (*skip)*	Y
*posterior sex combs* (*psc)*	Y	*boundary element-associated factor of 32KD* (*beaf32)*	N
*lethal (3) 73ah;* similar to *polycomb group ring finger protein 3* (*l(3)73ah)*	Y	*Histone h4* (*H4)*	Y
*activating transcription factor;* homologous to *Bombyx activating transcription factor of chaperone* (*atf-2)*	Y	*Histone h3.3* (*H3.3)*	Y
*cyclic-amp response element binding protein (1,2,3)*(*creb; dcreba)*	Y	*Histone h2a* (*H2a)*	Y
*creb binding protein;* similar to *nejire* (*crebbp(a))*	Y	*Histone h2a variant* (*H2a.v)*	Y
*retinoblastoma binding protein* (*rbp)*	Y	*mutagen-sensitive 308* (*PolQ; mus308 )*	Y
*retinoblastoma binding protein 2 (jumonji/arid domain containing); little imaginal discs* (*rbp2; lid)*	Y	*rpd3* (*hdac1; rpd3; hdac)*	Y
similar to *retinoblastoma binding protein 6* (*rbp6)*	Y	*mbd-like* (*mbd2/3; mbd-like)*	Y
*tousled-like kinase* (*tlk)*	Y	*mediator complex subunit 6* (*med6 )*	Y
*no child left behind;* similar to *wd repeat protein* (*nclb)*	Y	*mitochondrial single stranded DNA-binding protein* (*mtssb)*	Y
*Arginine methyltransferase 1; Arginine n-methyltransferase 1* (*DART1; prmt1)*	N	homolog of *recq* (*recq5)*	Y
*Arginine methyltransferase 2; Arginine n-methyltransferase 2* (*DART2; prmt2)*	N	*hen1* (*dmhen1; pimet)*	Y
*Arginine methyltransferase 3; Arginine n-methyltransferase 3* (*DART3; prmt3)*	Y	*Eukaryotic translation initiation factor 4G* (*eIF4G)*	Y
*Arginine methyltransferase 4; histone-Arginine methyltransferase carm 1* (*DART4; prmt4)*	Y	*Eukaryotic translation initiation factor 4A* (*eIF4A)*	Y
*Arginine methyltransferase 6; Arginine n-methyltransferase 6* (*DART6; prmt6)*	N	*Eukaryotic translation initiation factor 5* (*eIF5)*	Y
*Arginine methyltransferase 7; Arginine n-methyltransferase 7* (*DART7; prmt7)*	Y	*retrotransposon gypsy\envelope* (*gypsy\env)*	N
*Arginine methyltransferase 8; Arginine n-methyltransferase 8* (*DART8; prmt8)*	N	*jim* (*ovk; ovfc.k; jim)*	Y
*Arginine methyltransferase 9; Arginine n-methyltransferase 9* (*DART9; prmt9)*	N	*zelda; vielfaltig* (*vfl; zld)*	N
*absent, small, or homeotic discs 1* (*ash-1; ash; dash)*	Y	*Fcp1 RNA polymerase II CTD phosphatase*; CG12252 (*fcp1)*	Y
*bj1 protein;* homolog of *regulator of chromatin condensation 1* (*rangef; rcc1 )*	Y		

Genes identified mainly from the *Drosophila melanogaster* literature involved in regulation of chromatin structure during oogenesis, DNA replication, general transcription and maternal regulation of zygotic transcription in general. Presence (Y) or absence (N) of orthologous transcripts in the *Pararge aegeria* transcriptome is indicated.

### Genes influencing the cell cycle regulators of mitosis and meiosis

A large number of genes that regulate mitosis have been studied in a reproductive context in *D. melanogaster*. These genes are not only involved in stem cell maintenance and differentiation in the germarium, but also in relation to endocycling in nurse cells and selective amplication of genes (such as chorion genes) important in oocyte production (further references in Additional file 1). As before, the genes that were not expressed by *P. aegeria* in a mitotic context seemed either to have no clear insect orthologs outside *Drosophila*, or no such orthologs have been reported in Lepidoptera such as *B. mori* (Table 16). Among these are *dacapo* (*dap*), *matrimony* (*mtrm*), *microcephalin* (*MCPH1*) and *chiffon* (*chif*) (Additional file 1). The full list of genes in Table 16 contains a large number of cyclins, which regulate cyclin dependent kinases (CDKs). Orthologs of two common cyclins could not be found in the *P. aegeria* transcriptome: *cyclin E* and *J* (see the discussion on choriogenesis elsewhere in this paper).

**Table 16:** Cell cycle tregulation during mitosis and meiosis


*archipelago; WD repeat domain containing 7* (*ago)*	N	*myb transforming protein;* similar to CG6905 (*mybtp)*	Y
*dacapo* (*dap)*	N	*pitchoune* (*pit)*	Y
*coiled coil domain containing protein 25* (*ccdc25)*	Y	*rad51(−like); spindle A* (*rad51; spna)*	Y
*breast cancer 2, early onset homolog* (*brca2)*	Y	*tribbles* (*trbl)*	Y
*chiffon* (*chif)*	N	*fizzy; cdc20* (*fzy; cdc20)*	Y
*cyclin-dependent kinase 1; cell division cycle 2* (*cdk1; cdc2)*	Y	*meiotic 41 (*which is the *Drosophila atm/atr homolog)* (*mei-41; fs(1)m37)*	N
*cyclin-dependent kinase 2* (*cdk2)*	Y	*meiotic from via Salaria 332* (*mei-S332)*	N
*cyclin-dependent kinase 4* (*cdk4)*	Y	*mei-4 (*Forkhead domain containing*)* (*mei4)*	Y
*cyclin-dependent kinase 5* (*cdk5)*	Y	*mei-W68* (*mei-W68)*	N
*cyclin-dependent kinase 7* (*cdk7; mo15)*	Y	*cortex* (*cort)*	Y
*cyclin-dependent kinase 8* (*cdk8)*	Y	*grauzone* (*grau)*	N
*cyclin-dependent kinase 9* (*cdk9)*	Y	CG1647*; zinc-finger protein* (CG1647)	Y
*cyclin-dependent kinase 10 homolog; cdc2-related kinase* (*cdk10)*	Y	*btk family kinase at 29a* (*btk29a; tec29a)*	Y
*cyclin A* (*cycA)*	Y	*mutator 2* (*mu2)*	N
*cyclin B* (*cycB)*	Y	*myelin transcription factor 1* (*myt1)*	N
*cyclin B3; l(3)l6540* (*cycB3)*	Y	*orientation disrupter* (*ord)*	N
*cyclin C* (*cycC)*	Y	*mei-218* (*mei-218)*	N
*cyclin D* (*cycD)*	Y	*altered disjunction; mps1 (a kinetochore-associated protein kinase)* (*ald; mps1)*	N
*cyclin E* (*cycE)*	N	*no distributive disjunction* (*nod )*	N
*COP9 complex homolog subunit 5* (*csn5)*	Y	*sarah; nebula* (*sra; nla)*	Y
*COP9 complex subunit 3* (*csn3; dch3)*	Y	*calcineurin a* (*cana)*	Y
*COP9 complex subunit 4* (*csn4; dch4)*	Y	*calcineurin b* (*canb)*	Y
*COP9 complex subunit 6* (*csn6)*	Y	*mei-38* (*mei38)*	N
*COP9 complex subunit 7* (*csn7)*	Y	*ubiquitin conjugating enzyme E2 rad6* (*ubcd6; rad6)*	Y
*COP9 complex subunit 8* (*csn8)*	Y	*alpha-endosulfine* (*endos)*	Y
*cyclin H* (*cycH)*	Y	*early girl;* CG17033 (*elgi)*	Y
*cyclin J* (*cycJ)*	N	*encore* (*enc)*	N
*cyclin K* (*cycK)*	Y	*cullin 1* (*cul1; lin19)*	Y
*cyclin L1;* CG16903 (*cycL1)*	Y	*cullin 2* (*cul2)*	N
*cyclin T* (*cycT)*	Y	*cullin 4 (a and b)* (*cul4)*	Y
*cyclin fold protein; cyclin Y* (*cycfp; cycY)*	Y	*double parked* (*dup)*	Y
*cyclin M2* (*cycM2; cnnM2)*	Y	*cullin 5* (*cul5)*	Y
*cyclin-dependent kinase subunit 30a* (*cks30a)*	Y	*gustavus; Bombyx* sequence BHIBMGA008896-PA homologous to spry domain-containing socs box protein 4 (ssb4) (*gus; ssb4)*	Y
*cyclin-dependent kinase subunit 85a* (*cks85a)*	Y	*ubiquitin conjugating enzyme 2; l(2)k13206* (*ubcd2)*	Y
*diminutive; dmyc* (*dm)*	Y	*ubiquitin conjugating enzyme e2 d4* (*ubcd4)*	Y
*e2f1* (*e2f1)*	Y	*origin recognition complex subunit 1* (*ORC1)*	Y
*e2f5* (*e2f5)*	N	*origin recognition complex subunit 2; l(3)88ab* (*ORC2)*	Y
*dp; e2f dimerization partner 2* (*dp; tfdp2)*	Y	*origin recognition complex subunit 5; l(2)34df* (*ORC5)*	Y
*sin3a* (*sin3a)*	Y	*achintya* (*zaa)*	Y
*geminin* (*geminin)*	Y	*vismay* (*vis)*	N
*matrimony* (*mtrm; d52)*	N	*minichromosome maintenance 2 protein* (*mcm2)*	Y
*imaginal discs arrested* (*ida)*	N	*retinoblastoma-family protein 1* (*rbf1; rb1)*	N
*twine* (*twe)*	N	*grapes; serine/threonine-protein kinase chk1* (*chk1; lemp; grp)*	N
*string; cdc25 phosphatase* (*stg)*	N	*missing oocyte* (*mio)*	N
*microcephalin* (*MCPH1)*	N	*megator* (*mtor)*	Y
*inducer of meiosis 4; mta70 homologue* (*ime4)*	Y	*nucleoporin 44a;* similar to *sec13-like protein* (*seh1; nup44a)*	Y
*greatwall; mast-like* (*gwl)*	Y	*nucleoporin 154*; tulipano (*nup154; zk; nup32d; tlp)*	Y
*polo (kinase); l(3)01673* (*polo)*	Y	*kinesin-like protein ncd; non-claret disjunctional; claret segregational* (*ncd)*	Y
*loki; checkpoint kinase 2* (*lok; chk2)*	Y	*kinesin-13 motor; kinesin-like protein 10a; kinesin-like protein a (*in *Bombyx mori)*(*klp10a; klpa)*	Y
*always early;* a *lin9* homolog (*aly)*	Y	similar to *Bombyx mori kinesin-like protein b* (*klpb)*	Y
*pavarotti; kinesin family member 23* (*kif23; pav)*	Y	*crossover suppressor on 2 of Manheim* (*mei-910; c(2)M)*	N
*morula (anaphase-promoting complex subunit)* (*mr)*	Y	*crossover suppressor on 3 of Gowen* (*c(3)G)*	N
*proliferating cell nuclear antigen (mutagen-sensitive 209)* (*mus209; pcna)*	Y	*corona* (*cona)*	N
*mutagen-sensitive 304* (*atrip; mus304)*	N	*nipped-B* (*nipped-B)*	Y
*myb oncogene-like* (*myb)*	Y	*pch2* (*pch2)*	N
*the myb-muvb complex subunit lin-52* (*lin-52)*	Y	*Guanylate kinase-associated protein mars; hurp* (*hurp; dhrp/Gkap; mars)*	Y

Genes identified mainly from the *Drosophila melanogaster* literature that influence the cell cycle - regulators of mitosis (e.g. endocycling and selective amplification of chorion genes) and meiosis. Presence (Y) or absence (N) of orthologous transcripts in the *Pararge aegeria* transcriptome is indicated.

The cell cycle becomes arrested in meiotic prophase I in the majority of Metazoans oocytes. This is initiated during the first stages of oogenesis in region 2 of the *D. melanogaster* germarium [140]. The intriguing fact is that the gene *bruno* is not only essential in regulating the translation of a number of genes during oocyte differentiation, but it also appears to be involved in regulating the silencing of Cdk1 activity in order to achieve primary meiotic arrest [140]. It should be noted that oocyte AP and DV polarity is established during primary meiotic arrest and only once the oocyte is properly patterned by stage 14 is this arrest broken [140]. As indicated before, *bruno* was expressed by *P. aegeria* females (Table 9).

Meiosis during butterfly and moth oogenesis is characterised by the absence of crossing over and the formation of chiasmata [141,142]. Cytological studies have established that female Lepidoptera may form synaptonemal complexes (SC) in early meiotic prophase I, but no recombination nodules (RN) are formed subsequently. Instead, a structure called elimination chromatin is formed [143]. Usually chiasmata are formed from retained pieces of the SC in which a RN is, or has been, present [144]. The formation of the chiasmata takes place in the cell destined to become the oocyte in the *D. melanogaster* germarium [140]. Four genes appear essential in *D. melanogaster* for SC formation and thus possibly chiasmata formation: *crossover suppressor on 2 of Manheim* (*c(2)M*); *crossover suppressor on 3 of Gowen* (*c(3)G*); *corona* (*cona*) and *nipped-B* (references in Additional file 1). No genes specific for RN alone could be identified on FlyBase [62]. *Pararge aegeria* females only express *nipped-B* (Table 16 and Additional file 1), which is involved in a number of cellular processes in *D. melanogaster* including mitosis [145]. It is also the only one of the four SC genes for which orthologs outside *Drosophila* can be identified. Rather interestingly, a large proportion of the genes involved in *D. melanogaster* meiotic chromosome cohesion and segregation also appeared to be *Drosophila* or Diptera specific and were not identified in the *P. aegeria* transcriptome. These include *grauzone* (*grau*), *corona* (*cona*), *orientation disrupter* (*ord*) and *mei-S332* (Table 16; references in Additional file 1). A number of genes are, however, highly conserved and orthologs have been found in Lepidoptera as males do display crossing-over [141,142]. These include both *mei-W68* and *mei-218* but in particular includes the essential meiotic checkpoint gene *pch2* (references in Additional file 1). Female *P. aegeria* did not express any of these genes (Table 16 and Additional file 1). The *P. aegeria* oogenesis transcriptome described here is thus in accordance with the previous observations made during cytological studies on female Lepidoptera [141-143].

### Vitellogenesis and lipid storage

Not only is cell cycle regulation coordinated with oocyte differentiation in *D. melanogaster*[140], but also with resource provisioning of the oocyte [22]. The gene *greatwall* (*gwl*), for example, is both essential in *D. melanogaster* for maternal provisioning of the egg during vitellogenesis and to ensure secondary meiotic arrest by stage 14 of oogenesis in metaphase I [22]. It is a highly conserved gene in Metazoa and *P. aegeria* females did express this gene during oogenesis (Table 16 and Additional file 1). Furthermore, *gwl* (antagonistically) interacts with *polo kinase* (*polo*) in mitotic regulation particularly during early embryogenesis, and is maternally provided (references in Additional file 1). Transcripts of both were detected in *P. aegeria* oocytes (Table 16; Additional files 1 and 2).

Vitellogenesis during insect oogenesis is characterised by the accumulation in the developing oocytes of large lipid transfer proteins (LLTPs; i.e. yolk protein precursors), such as Vitellogenin (Vtg/Vg) and Apolipophorins (ApoLPs) [8,9]. Predominantly, LLTPs are produced in the fat bodies and secreted into the hemolymph [8,9], but not all yolk proteins are extraovarian [146]. Follicle cells not only allow extraovarian yolk protein to reach the oocytes, they also produce significant amounts of LLTPs themselves in a number of insect species, including *D. melanogaster*[146]. Vitellogenic behaviour of follicle cells is under hormonal control [146]. LLTPs are transported into the oocytes via clathrin-dependent endocytosis mediated by the receptors VgR (in *D. melanogaster* Yolkless, Yl) and LpR [9,147]. Nurse cells transport *yl/VgR* RNA into previtellogenic oocytes, thus preparing the oocyte for Vtg uptake [148]. *Pararge aegeria* females expressed not only *Vtg/Vg*, *apoLp-III*, *apoLp*, their receptors *yl*/*VgR* and *LpR*, but also the genes described in *D. melanogaster* vitellogenic endocytosis (references in Additional file 1). These genes include *clathrin heavy* and *light chain* (*chc* and *clc*), *sec5*, *sec6, garnet* (*G*) and *jagunal* (*jagn*) (Figure 4 qPCR results; Tables 2 and 17; further references in Additional file 1).

**Table 17:** Reproductive physiology and vitellogenesis


*apolipophorin-III* (*apoLp-III)*	Y	homologous to *Bombyx juvenile hormone epoxide hydrolase-like protein 3* (*jheh-lp3)*	Y
*apolipophorin precursor; Drosophila* CG11064 (*apoLp; apolp1/2)*	Y	homologous to *Bombyx juvenile hormone epoxide hydrolase-like protein 5* (*jheh-lp5)*	Y
*lipophorin receptor* (*Lpr1/2)*	Y	*juvenile hormone binding protein;* homologous to *Drosophila* CG1532 (*JHbp)*	Y
*arylphorin (subunit beta); sex-specific storage-protein 2* (*hex2; sp2)*	Y	*juvenile hormone binding protein (hemolymph)* (*hJHbp)*	Y
*vitellogenin* (protein cleaved into vitellin light chain (vl), vitellin light chain rare isoform, vitellin heavy chain rare isoform and vitellin heavy chain (vh)) (*Vg; Vtg)*	Y	*cytosolic juvenile hormone binding protein 36 KDa subunit* (*cJHbp)*	Y
*vitellogenin receptor; yolkless* (*yl; VgR)*	Y	*takeout* (*to)*	Y
*spherulin-2a (*similar to *Plodia interpunctella yp4)*(*yp4)*	Y	similar to *niemann-pick type c-2; ecdysteroid-regulated 16 kDa protein precursor* (*npc2a; esr16)*	Y
*chico* (*chico; IRS)*	Y	*ecdysone-induced protein 63e* (*Eip63E; cdc2-63E)*	N
*Bombyxin* genes(*bbxA1; bbxA3)*	Y	similar to *sgt1 protein homolog ecdysoneless* (*ecd)*	Y
*insulin-like receptor* (*InR)*	Y	*cytochrome p450 (E-class, group I) protein disembodied* (*dib; cyp302a1)*	N
*ribosomal protein l10a* (*rpl10ab )*	Y	*halfway; singed wings* (*hfw; swi)*	Y
*60s ribosomal protein l10; qm protein homolog* (*qm)*	Y	*clathrin light chain* (*chc)*	Y
*string of pearls; ribosomal protein s2* (*sop; rp2)*	Y	*clathrin heavy chain* (*clc)*	Y
*resistance to juvenile hormone; methoprene-tolerant* (*met)*	Y	*ced-6* (*ced-6)*	Y
*ultraspiracle; rxr type hormone receptor* (*usp; cf1)*	Y	*wnt receptor l(2)43Ea boca* (*boca)*	Y
*ecdysone receptor* (*EcR)*	Y	*jagunal* (*jagn)*	Y
*start1* (*start1)*	Y	*exocyst complex component sec5* (*sec5)*	Y
*defective in the avoidance of repellents dare; adrenodoxin reductase* (*dare)*	Y	*exocyst complex component sec6* (*sec6)*	Y
*ecdysone-induced protein 74* (*E74)*	N	*protein phosphatase 2a regulatory subunit b’; widerborst* (*wdb; PP2Ab’)*	Y
*ecdysone-induced protein 75b (75a,b,c and d)* (*E75)*	Y	*protein phosphatase 2a regulatory subunit b 55kDa; twins* (*PP2Ab55kDa)*	Y
homologous to *Bombyx mori c-cbl-associated protein (cap) transcript variant a* (*bmcap-a)*	Y	*protein phosphatase 2a regulatory subunit b gamma* (*PP2Agamma)*	Y
*follicle specific protein* (*fsp-I)*	N	*protein phosphatase 2a regulatory subunit a (65 kDa);* homologous to *Drosophila protein phosphatase 2a at 29b* (*PP2Aa)*	Y
similar to *Bombyx mori egg-specific protein (*LOC693022*)* (*ESP)*	N	*microtubule star; protein phosphatase 2a catalytic subunit c* (*mts; PP2Ac)*	Y
*calmodulin* (*cam)*	Y	*lipid storage droplet 1; perilipin 1* (*lsd1; plin-1; plin1)*	Y
*calmodulin-binding protein (striatin);* weak homology to CG7392 (*striatin)*	Y	*lipid storage droplet 2* (*lsd2)*	Y
*calmodulin dependent protein kinase* (*camk)*	Y	*lipase-1* (*lip-1)*	Y
*hormone receptor 3; Drosophila hormone receptor-like in 46* (*hr3; hr46)*	Y	*serine/threonine protein kinase akt* (*akt; akt1)*	Y
*hepatocyte nuclear factor 4 isoform a* (*hnf-4a)*	Y	*liquid facets-related* (*lqfr)*	Y
*hepatocyte nuclear factor 4 isoform b* (*hnf-4b)*	Y	*liquid facets* (*lqf)*	Y
*juvenile hormone esterase* (*jhe)*	N	*garnet* (*g)*	Y
*juvenile hormone esterase binding protein;* weak homology to *Drosophila* CG3776 (*JHEbp; DmP29)*	Y	*cationic amino acid transporter; slimfast* (*slif)*	Y
*juvenile hormone epoxide hydrolase* (*JHEH)*	Y	*ornithine decarboxylase* (*odc)*	Y
homologous to *Bombyx juvenile hormone epoxide hydrolase-like protein 1* (*jheh-lp1)*	Y	*ornithine decarboxylase antizyme; gutfeeling* (*guf; Oda; az)*	Y

Genes identified mainly from the *Drosophila melanogaster* and *Bombyx mori* literature involved in vitellogenesis, lipid storage, ovarian maturation and hormonal regulation of oogenesis. Presence (Y) or absence (N) of orthologous transcripts in the *Pararge aegeria* transcriptome is indicated.

The major yolk proteins, such as vitellogenins, share sequence similarities with lipases. Although not catalytically active, the vitellogenin region with sequence similarity to lipases is argued to be involved in steroid hormone binding, thus providing a possibility for a direct interaction with the hormones that regulate their production [149]. For example, maternal ecdysteroids are bound as ecdysteroid-phosphates to the Vtg cleaved product Vitellin (Vn) in yolk granules in *B. mori* and released as ecdysteroids during yolk uptake in the embryo as a result of dephosphorylation by ecdysteroid-phosphate phosphatase (EPPase)[150]. *Pararge aegeria* did express *EPPase* (Table 18). Furthermore, a significant component of yolk in a *B. mori* egg is the ovarian egg-specific protein ESP, a minor yolk protein [151]. The gene encoding ESP is intriguing, as convincing orthologs for minor yolk proteins outside the moths *Galleria mellonella* (yolk protein/yolk polypeptide 2) and *Samia cynthia* (ESP) had not been found [149]. More recently, however, a further two sequences with strong sequence similarity to *G. mellonella* yolk protein 2 have been discovered in *D. plexippus* and *Plodia interpunctella,* whilst *ESP* does show significant sequence similarity with genes encoding the KK-42 binding proteins in *Antheraea* moth species [152] (Additional file 9). Sharing the same ABhydrolase lipase region, The KK-42 binding proteins and the minor yolk proteins also show strong sequence similarity to lipases identified in species such as *D. melanogaster*, in particular *lipase-1 and 3* (*lip-1 and 3*) [149]. Lepidoptera may have evolved to use paralogs of these genes in yolk formation*.* Rather interestingly*,* although not functioning as a yolk protein*, lip-1,* but not *lip-3*, is expressed in vitellogenic follicles in *D. melanogaster*[149]. An orthologs of *lip-1*, and possibly *lip-3* (very short partial contig), was expressed by *P. aegeria*, whilst no clear ortholog of a minor yolk protein was found (Table 17; Additional files 2 and 9).

**Table 18:** Yolk consumption


*cathepsin l-like cysteine protease; Bombyx cysteine protease; cysteine proteinase-1* (*bcp; cl; cp1)*	Y	*vacuolar proton atpase; vacuolar h+ atpase subunit 100–2* (*vha100-2)*	Y
*cathepsin b; cathepsin b-like cysteine proteinase* (*catb)*	Y	*h+ transporting atpase v0 subunit d; vacuolar h+ atpase subunit ac39-1* (*vhaac39-1)*	Y
*cathepsin d; aspartic protease* (*catd)*	Y	*vacuolar atp synthase subunit d; vacuolar h+ atpase subunit 36–1* (*mvd; vha36-1)*	Y
*cathepsin f-like cysteine protease;* CG12163 (*catf)*	Y	CG7899*; acid phosphatase 1* (*acph-1; ap)*	N
*ecdysteroid-phosphate phosphatase* (*EPPase)*	Y	*primo-1; acid phosphatase isoenzyme* (*primo-1)*	Y
*vacuolar proton atpase; vacuolar h+ atpase subunit 100–1* (*mva; v100; vha100-1)*	Y		

Maternal effect genes, identified mainly from the *Drosophila melanogaster* and *Bombyx mori* literature, involved in facilitating yolk consumption by the developing embryos. Presence (Y) or absence (N) of orthologous transcripts in the *Pararge aegeria* transcriptome is indicated.

Among the most highly transcribed genes in *P. aegeria* ovarioles is an ortholog of the slime mold *Physarum polycephalum* gene *spherulin-2A.* No transcripts were found for this gene in eggs (Table 2 and Additional file 2). Lepidopteran orthologs of the protein encoded by this gene have been shown to function as a subunit Yp4 of follicular epithelium yolk protein produced by follicle cells [153].

Yolk is a food source for the developing embryo and a number of genes encoding Cathepsins and Vacuolar Proton ATP-ases are maternally expressed during oogenesis to facilitate yolk uptake in the embryos (references in Additional file 1). *Pararge aegeria* females were found to express all described yolk uptake genes, with the exception of the *acid phosphatase 1* gene (*acph-1*) (Table 18 and Additional file 1).

### Physiology of oogenesis

Reproductive output depends on female nutritional status which not only affects the rate and duration of oogenesis significantly, but also whether previtellogenic egg chambers will enter the vitellogenic stage or apoptose [154]. Two signalling systems are involved; insulin and hormone signalling [155]. In *D. melanogaster*, for example, absence of the insulin receptor substrate (IRS) Chico precludes vitellogenesis, whilst a sharp increase in 20-hydroxy-ecdysone (20E) relative to juvenile hormone (JH) results in apoptosis of the egg chamber before vitellogenesis is initiated or completed [16,155]. Although the two signalling systems operate simultaneously and interact, both have been shown to be able to independently terminate egg chamber progression before vitellogenesis takes place in *D. melanogaster*[155]. Furthermore, the Lepidoptera express a set of unique genes encoding insulin-like peptides, the Bombyxins (Bbx) [156]. The *bbx* genes are expressed predominantly in the brain, but some may also be expressed in ovaries [156]. Moths, in particular *B. mori*, possess a large number of *bbx*-like genes in their genome [156], but the genome of the butterfly *D. plexippus* appears to have only three such genes [50]. Orthologs of 2 of these 3 (*bbxA1-like* and *bbxA3-like*) were transcribed in *P. aegeria* ovarioles, whilst a third partial IRS transcript showed more sequence similarity to *chico* than to any *bbx-*like gene (Table 17 and Additional file 1). The *insulin-like receptor* (*InR*) was also expressed by *P. aegeria* during oogenesis (Table 17 and Additional file 1). Furthermore, *P. aegeria* expressed a large number of downstream target genes of insulin signalling including genes encoding the serine/threonine protein kinase Akt, the various protein phosphatase 2A subunits (PP2A, e.g. Widerborst) and the lipid storage droplet proteins 1 and 2 (Lsd1 and Lsd2). Please refer to Table 17 and references in Additional file 1 for additional details.

Apart from nutritional status, environmental factors such as temperature can affect hormone concentrations, providing a possibility for environmental control of reproductive output [7,26]. The interplay between 20E and JH is dynamic and complex, as both 20E and JH also play a role in regulating choriogenesis [157]. Both hormones have a range of pleiotropic effects during oogenesis and their exact developmental role is not only titre related, but also dependent on the dynamic spatio-temporal expression patterns of the receptors and modulators of hormone signalling [157].

There has been extensive investigation of JH signalling [7,26], but the signal transduction pathway, including the JH receptor, remains poorly understood [158-160]. The most likely candidate gene for the JH receptor proposed to date is the basic helix–loop–helix (bHLH)/Per-Arnt-Sim (PAS) domain gene *methoprene-tolerant* (*met*) [158-160]. It may form a homodimer, or possibly may form a JH-dependent transcriptionally active complex with another member of the bHLH-PAS family. The most likely candidate for the complex is the steroid co-activator NCoA-1/p160 FISC, encoded by the gene *taiman* (*tai*) in *D. melanogaster*[158,160]. The *tai* gene was originally discovered as a gene that was expressed in follicle cells in the functional context of border cell migration and was described as an ecdysone co-receptor (Table 6; references in Additional file 1). *Pararge aegeria* females expressed both *met* and *tai* (Tables 6 and 17 and S2; contigs for *tai* PACG7006 and PACG13674 in Additional file 2). An ortholog for *tai* (UNIPROT: G6DPV9) can also been found in the genome of *D. plexippus*[50].

Not much is known about which genes are transcriptionally regulated by the JH activated receptor complex [161]. The gene *kruppel-homolog 1* (*krh1*) has been described as a JH response gene, inhibiting 20E induced *broad* (*br*) expression in *D. melanogaster*, but not in the specific context of oogenesis [159]. Both *khr1* and *br* were expressed by *P. aegeria* females (Additional file 1). Furthermore, JH may either directly or indirectly upregulate *ornithine decarboxylase* (*odc*), which regulates polyamine biosynthesis and appears to be essential for vitellogenesis [162]. Both *odc* and its antagonist *gutfeeling* (*oda*), also a mitotic cell-cycle regulator, were expressed in *P. aegeria*. Maternal transcripts of *odc* and *oda* were found in eggs (Figure 4 qPCR results; Table 17, Additional files 1 and 2).

In order to regulate the precise amount of JH in both hemolymph and organs, two sets of enzymes are involved in JH degradation; the JH epoxide hydrolases (JHEHs) and the JH esterases (JHEs) [163]. JHEs function predominantly in the hemolymph and degradation is reversible, whilst JHEHs regulate the amount of JH in organs and degradation is irreversible [163]. Apart from JHEH, five recently discovered JHEH-like protein genes have been characterised in *B. mori*[163] and in addition to *JHEH*, *P. aegeria* expressed orthologs of three of these; *jheh-lp1*, *jheh-lp3* and *jheh-lp5* (Table 17 and Additional file 1). With the exception of *jheh-lp5*, moderate amounts of transcripts of JHEHs were found in the eggs (Additional file 2). The females did not express a clear ortholog of *jhe*, but did express an ortholog of a gene encoding an intracellular binding protein of JHE presumed to be involved in its transport (*JHEbp* or *DmP29*, *Drosophila mitochondrial protein 29*, Table 17). Significant amounts of maternal *JHEbp* transcripts were found in *P. aegeria* eggs (Additional file 2).

Juvenile hormone itself may be bound by JH binding proteins (JHbp) to enable immobilisation, regulate degradation or enable transport [28]. Four complete *JHbp* CDSs were identified in *P. aegeria* ovaries; *JHbp*, *cytosolic JHbp* (*cJHbp*), *hemolymph JHbp* (*hJHbp*) and a sequence showing strong orthology to *takeout* (*to*) identified in *D. melanogaster* as involved in JH binding (Table 17). Transcripts of both *cJHbp* and *to* were transferred to the eggs by *P. aegeria* (Additional file 2). Given that JH itself can be transferred maternally into eggs in Lepidoptera, it has been argued that JH binding proteins such as cJHbp will protect the developing embryo against the teratogenic effects of any excess JH transferred from the mother [28].

There is a significant amount of life-history variation among insects and consequently in the relative importance of 20E and JH on oogenesis [26], even within Lepidoptera [8]. Lepidoptera have been categorised into four (physiological) groups based on the hormones used to initiate vitellogenesis, choriogenesis and thus the timing of mature egg production [7]. Nymphalids*,* like *P. aegeria*, have been argued to best match the criteria for group 4 [7] where JH is the essential gonadotropic hormone. Juvenile hormone in this group is necessary for: a) synthesis of Vtg in the fat body and possibly the ovary (results supporting the latter in this study); b) inducing patency of ovarioles; c) uptake of Vtg by the oocyte (follicle cells deform to facilitate this uptake and this deformation is under JH control) and d) choriogenesis by the follicle cells. Whilst 20E modulates JH signalling in Nymphalids, it plays a more significant role in vitellogenesis and choriogenesis regulation in *B. mori* and *D. melanogaster*[7,146].

Ecdysone signalling, including its target genes, is in general better understood than JH signalling [164]. *Bombyx mori* appears to be capable of producing ecdysteroids in the ovaries [8], as does *D. melanogaster*[165]. *Drosophila melanogaster* expresses *start1* during oogenesis in significant amounts in nurse cells, most likely in response to ecdysone signalling. The cholesterol transporter Start1 may in turn facilitate ecdysteroid production from cholesterol-based precursors [165]. Another gene expressed in the nurse cells essential during *D. melanogaster* cholesterol conversion in the ovaries is *defective in the avoidance of repellents (dare*), which encodes an Adrenodoxin reductase [166]. Furthermore, in *D. melanogaster* the SGT1 protein homolog *ecdysoneless* (*ecd*) and *disembodied* (*dib*) have been described as essential for ecdysone, both for functionality and its production in the ovaries [165,167]. Maternal transcripts of *D. melanogaster start1* are hypothesised to be deposited into the egg to facilitate ecdysteroid signalling in the developing embryo [165]. Rather intriguingly *P. aegeria* females did not express *dib*, but did express *ecd, start1*, and *dare*. We observed the transfer of transcripts of all three genes into the oocytes (Table 17 and Additional file 2). Start1 has been implicated in ecdysteroid synthesis in the prothoracic gland in *B. mori*[168]. Further investigation is needed to determine whether ecdysteroids can be produced in *P. aegeria* ovaries and if the transfer of maternal *start1* and *dare* transcripts is involved in ecdysteroid signalling in early embryos. In common with the majority of insects [8,157], *P. aegeria* females did express *ecdysone receptor* (*EcR*) and its partner *ultraspiracle* (*usp*; labelled *chorion factor 1* (*cf1*) in *B. mori*) in the ovaries (Table 17). Although JH may be the gonadotropic hormone in *P. aegeria*, it is clear from the expression results presented here that 20E signalling does play a significant role in vitellogenesis and that there may be maternal regulation of ecdysteroid signalling in early embryos.

Among the so-called early genes in the hierarchy of genes up-regulated in response to activation of EcR in *B. mori* ovaries are the orphan nuclear receptor genes *hr3* and *E75(a,b, c* and *d*), the transcription factor gene *E74* and the *Broad-Complex gene Br-C*[151]. The genes encoding the two receptors Hepatocyte nuclear factor 4a and 4B (HNF4A and HNF4B) are up-regulated with a delay in *B. mori* and their expression increases during vitellogenesis [169]. With the exception of *E74,* all of these genes were expressed in *P. aegeria* (Tables 6, 17 and Additional file 1). In *B. mori* Hr3 regulates the expression of *ESP* during vitellogenesis, and it regulates the expression of *GATAbeta* (i.e. *transcription factor BCFI*) during choriogenesis [151]. As discussed before, *P. aegeria* females did not express *ESP*, but did express the related gene *lip-3* (Table 17). Furthermore, they also expressed *GATAbeta* (Table 19 and Additional file 1).

**Table 19:** Eggshell formation


weak homology to *Bombyx mori vitelline membrane associated protein p30* (*VMP30)*	Y	*chorion peroxidase; peroxinectin-related protein* (*pxt)*	Y
*Bombyx mori vitelline membrane protein 90* (*VMP90)*	N	*gataβ; transcription factor BCFI* (*GATAβ)*	Y
*vitelline membrane 32e* (*VM32e; VMP32e)*	N	*chorion transcription factor cf2* (*cf2)*	Y
*vitelline membrane 26a* (*VM26a)*	N	*chorion b-ZIP transcription factor* (*CbZ)*	Y
*vitelline membrane 26b* (*VM26b)*	N	*chorion protein 15 (Drosophila melanogaster);* CG6519 (*cp15; s15)*	N
*vitelline membrane 26ac* (*VM26Ac; tu-3)*	N	*chorion protein 16 (Drosophila melanogaster);* CG6533 (*cp16; s16)*	N
*vitelline membrane 34ca* (*VM34c)*	N	*chorion protein 18 (Drosophila melanogaster);* CG6517 (*cp18; s18)*	N
*femcoat* (*femcoat)*	N	*chorion protein 19 (Drosophila melanogaster);* CG6524 (*cp19; s19)*	N
*follicle cell protein 26Aa; palisade* (*psd; fcp26Aa; tu-1)*	N	*chorion protein 36 (Drosophila melanogaster);* CG1478 (*cp36; s36)*	N
*cad99c* (*cad99c; ca-10)*	Y	*chorion protein 38 (Drosophila melanogaster);* CG11213 (*cp38; s38)*	N
*crinkled; myosin-VIIa* (*ck; myoVIIa)*	Y	*chorion protein a at 7f (Drosophila melanogaster);* CG33962 (*cp7fa)*	N
*vitelline membrane like* (*vml)*	N	*chorion protein b at 7f (Drosophila melanogaster);* CG15350 (*cp7fb)*	N
*high mobility group protein a* (*HMGa)*	Y	*chorion protein c at 7f (Drosophila melanogaster);* CG15351 (*cp7fc)*	N
*egg protein 80* (*EP80)*	Y	*defective chorion 1* (*dec1)*	N
*follicle cell protein 3c* (*fcp3c)*	Y	Lepidopteran *chorion* genes (see Additional file 9)	Y

Genes identified mainly from the *Drosophila melanogaster* and *Bombyx mori* literature involved in eggshell formation; vitelline membrane formation and choriogenesis. Presence (Y) or absence (N) of orthologous transcripts in the *Pararge aegeria* transcriptome is indicated. See Additional file 9 for details on lepidopteran chorion genes.

### Vitelline membrane formation and choriogenesis

Vitellogenesis and choriogenesis are carefully coordinated, primarily by hormone signalling. The vitelline membrane (i.e. the inner eggshell layer) is formed halfway through vitellogenesis [170], for which RTK signalling is necessary as discussed elsewhere in this paper. The formation of the vitelline membrane is of significance in maternal regulation of embryonic AP and DV patterning, as some maternal factors become localised in the perivitelline space in *D. melanogaster* and interact with localised factors inside the oocyte [170]. This also appears to be the case in *B. mori*[94], although the genes involved remain uncharacterised. As discussed before, Ndl protein (also tellingly called ovarian serine protease in *B. mori*) is expressed in all follicle cells and is essential for DV patterning of the embryo in *D. melanogaster*[171]. Ndl is an unusual protein in that not only is its structure reminiscent of an extracellular matrix protein, but that it also has a catalytically active serine/protease domain [171]. As such, it is involved in both vitelline membrane formation as well as acting as the basis of the serine/protease cascade ventrally, essential for the maternally regulated DV patterning of the *D. melanogaster* embryo [170]. *Pararge aegeria* females expressed *ndl* and as in *D. melanogaster*, no transcripts were found in the oocyte (Table 6 and Additional file 2). It remains to be seen whether Ndl plays a similar dual role in *P. aegeria*.

Insect vitelline membrane protein (VMP) genes show tremendous sequence diversity. For example, no clear orthologs can be found for *D. melanogaster VMP* genes outside the genus *Drosophila*. The best-characterised *VMP* gene in Lepidoptera is *VMP30*[172], for which orthologs can be found in both moths and butterflies and which was also expressed in *P. aegeria* ovarioles. Once again, no transcripts were found in the oocyte (Table 19 and Additional file 2).

After the follicle cells have secreted proteins to form the vitelline membrane, endocycling takes place in *D. melanogaster* and clusters of chorion genes are selectively amplified or expressed at very high levels [170,173]. Perhaps rather surprisingly, *P. aegeria* did not express an ortholog of G1/S specific *cycE*, which in *D. melanogaster* is essential for chorion gene amplification and endocycling in general ([173]; Table 16; further references in Additional file 1). There is a possibility that Lepidoptera do not selectively amplify the chorion genes prior to the onset of choriogenesis, as no evidence was found for this in *B. mori*[174]. However, nurse cells do become polyploid during *B. mori* oogenesis [8]. *Pararge aegeria* females did express the G1/S specific genes *cycC* and *cycD*, as well as the S-phase regulators *E2f1* and *dp* (Table 16; further references in Additional file 1).

Choriogenesis as a whole is coordinated by genes such as *chorion peroxidase* (*pxt*) in *D. melanogaster*[170], which was also expressed by *P. aegeria* (Table 19). Furthermore, apart from aforementioned GATAbeta, a number of specific transcription factors are involved in the critical regulation of the spatio-temporal expression patterns of the various chorion genes in the later stages of oogenesis in Lepidoptera. All chorion genes in *B. mori* have multiple *cis*-regulatory binding sites for CCAAT/enhancer binding protein (C/EBP) transcription factors and their expression levels are C/EBP concentration dependent [175]. The *D. melanogaster* ortholog of C/EBP is *slbo*, which is also expressed in follicle cells though predominantly involved in border cell migration (references in Additional file 1). High mobility group protein A (HMGA) is essential for *B. mori* choriogenesis as it induces chorion gene promoter bending and recruits C/EBP and GATAbeta [176]. *Pararge aegeria* expressed *C/EBP* (i.e. *slbo*), its negative regulator *tribbles* (*trbl*) and *HMGa* (Tables 6, 16 and 19), but it is not known in which functional context *slbo* is used. Another transcription factor for which *cis*-regulatory binding sites have been identified for chorion genes, in both *D. melanogaster* and *B. mori*, is the C_2_H_2_ zinc finger protein Chorion factor 2 (Cf2) [177]. Furthermore, a chorion-specific b-ZIP transcription factor (CbZ) has been described in *B. mori*[175] and orthologs can be found in butterfly genomes, such as that of *D. plexippus*[50]. However, the exact function of CbZ during choriogenesis has not been characterised. Both *cf1* and *CbZ* were transcribed by *P. aegeria*, with transcripts of the latter rather intriguingly found to be present in the oocyte (Figure 4 qPCR results; Table 19).

Chorion protein (cp) genes evolve possibly even faster than vitelline membrane protein genes [178] and sequence similarity between *D. melanogaster cp* genes with those identified in Lepidoptera, including *P. aegeria*, is very low indeed (Table 19; further references in Additional file 1). The infraorder Heteroneura, to which *B. mori* and butterflies belong, possess unique helicoidal lamellar chorions, which may provide additional strength [61]. Furthermore, the two species for which chorion genes have been characterised and studied in some detail, *Lymantria dispar* and *B. mori*, have an extensively derived chorion in which the helicoidal lamellar framework is modified by expansion and densification [61]. Expression patterns of these chorion genes are also dynamically very complex. Gene families in Lepidoptera encoding the structural chorion proteins are characterised by numerous gene duplications, occasional subsequent gene loss, gene conversion, and in general rapid sequence divergence [61,179]. As a result, determining orthology between individual chorion genes of different species is very difficult and chorion protein phylogenetic trees are characterised by species-specific clusters (i.e. families) of genes [179]. Automatic annotation of butterfly chorion genes in the *D. plexippus* genome and from our *P. aegeria* ovarian transcriptome was performed on the basis of the most significant BLAST hit to available moth chorion gene sequences (Additional file 2 and Table 19). It is very doubtful, however, that true orthology has been uncovered in this way, as chorion genes within a species tend to be more similar to each other than to those found in other species. The phylogenetic tree of Lepidopteran chorion genes in Additional file 9 shows distinct clustering between moths and butterflies for each of the chorion gene families. *Pararge aegeria* chorion genes were highly transcribed during oogenesis (Table 2 and Additional file 1). As well as expressing these chorion gene families, *Bombyx mori* expresses a gene encoding protein 80 (BmEP80), which forms part of the eggshell and is produced by the follicle cells [180]. BmEP80 is also highly transcribed during *P. aegeria* oogenesis (Tables 2 and 19; Additional data file 1).

### Apoptosis and autophagy

Programmed cell death is an essential process during oogenesis in *D. melanogaster* and *B. mori*, with nurse and follicle cells undergoing apoptosis as oogenesis progresses, while complete egg chambers may apoptose in response to environmentally induced hormonal signals such as starvation [15,16,154,181]. Often, apoptosis and autophagy operate synergistally [181] and are to some extent integrated in *D. melanogaster* ovaries, where the effector caspase Dcp-1 and the inhibitor of apoptosis protein BIR-superfamily domain protein Bruce (also called survivin in *B. mori*) regulate both autophagy and starvation-induced cell death [182]. Recently, all apoptosis-related genes have been characterised in *B. mori*, and the results of the study by Zhang and co-workers showed that most of these genes are highly conserved [183]. Furthermore they demonstrated that a number of gene duplications have occurred in the Lepidoptera (e.g. genes ecoding BIR-superfamily domain proteins)[183]. Many of the known genes involved in autophagy and apoptosis have been studied in a reproductive context in *D. melanogaster* (references in Additional file 1) and the majority of these were expressed during oogenesis by *P. aegeria* (Table 20). In particular, *P. aegeria* expressed *buffy*, three orthologs of *bruce* (Additional file 2) and the Lepidopteran ortholog of *D. melanogaster dcp1*, *caspase-1* (Table 20).

**Table 20:** Growth regulation, apoptosis and autophagy


*p53* (*p53)*	Y	*quaking related 54b; sam50* (*qkr; sam50)*	Y
*p35* (*p35)*	N	*held out wings* (*how)*	Y
*death executioner Bcl-2 homologue* (*debcl)*	N	*spinster* (*spin)*	Y
homologous to *bruce and Bombyx bir-superfamily domain protein - survivin-1* (*bruce; survivin-1)*	Y	*death executioner caspase related to apopain/yama; decay; caspase 3* (*decay)*	N
*bir-superfamily domain protein - inhibitor of apoptosis 1; thread* (*iap1; th; diap1)*	Y	*death caspase 1* (*dcp-1)*	N
*bir-superfamily domain protein - inhibitor of apoptosis 2* (*iap2; diap2)*	Y	*death related ced-3/nedd2-like protein; dredd/dcp-2* (*dredd)*	Y
*ubiquitin conjugation enzyme E2; bendless* (*ubc13; ben)*	Y	*ice; drice; caspase-1 (*in *Bombyx mori)* (*ice)*	Y
*b-cell lymphoma protein 2 (bcl-2) protein - buffy* (*buffy)*	Y	*dronc; nedd2-like caspase* (*dronc; nc)*	Y
*autophagy-specific gene 1; serine/threonine-protein kinase unc-51* (*atg1)*	Y	*dynamin related protein 1* (*drp1)*	Y
*autophagy-specific gene 2* (*atg2)*	Y	similar to *optic atrophy 1-like* (*opa1-like)*	Y
*autophagy-specific gene 3* (*atg3; aut1)*	Y	*resistance to juvenile hormone; methoprene-tolerant* (*met)*	Y
*autophagy-specific gene 4* (*atg4)*	Y	*deterin* (*det )*	N
*autophagy-specific gene 5* (*atg5)*	Y	*tao-1* (*tao-1)*	Y
*autophagy-specific gene 6; beclin-1* (*atg6)*	Y	*melted* (*melt)*	N
*autophagy-specific gene 7* (*atg7)*	Y	*midway* (*mdy)*	N
*autophagy-specific gene 8* (*atg8)*	Y	*pita* (*pita)*	Y
*autophagy-specific gene 12* (*atg12)*	Y	*plenty of sh3s* (*posh)*	N
*autophagy-specific gene 13* (*atg13)*	N	*phosphoinositide-dependent kinase 1 dstpk61* (*dstpk61)*	Y
*phosphotidylinositol 3 kinase 59f* (*pi3k59f; vps34)*	Y	*dream* (*strica; dream)*	N
*cell death activator-b* (*cide-b)*	Y	*target of rapamycin* (*tor)*	Y
*cell cycle and apoptosis regulatory protein 1 (ccar1)*	Y	*thor* (*thor)*	N
*longitudinals-lacking* (*lola)*	Y	*death associated molecule related to mch2; daydream* (*damm)*	N
*translationally controlled tumour protein* (*tctp)*	Y	*ecdysone-induced protein 28/29kD; methionine-s-sulfoxide reductase* (*Eip28/29; Eip71CD)*	Y
*apoptosis linked protein 2* (*alg-2)*	Y	*modifier of rpr and grim, ubiquitously expressed;* weak homology to *ubiquitin-conjugating enzyme E2 D4* (*morgue)*	N

Genes identified mainly from the *Drosophila melanogaster* literature involved in regulation of growth during oogenesis (apoptosis, autophagy - response to starvation). Presence (Y) or absence (N) of orthologous transcripts in the *Pararge aegeria* transcriptome is indicated.

### General growth regulators (including the Hippo Pathway)

Hippo is a highly conserved serine-threonine kinase 3-like signalling protein (also called STE20). It is essential for regulating tissue size and growth [184]. Hippo signalling interacts with various other cellular processes in this functional context, including programmed cell death and cell cycling [184]. Hippo signalling is, however, required in a wide variety of developmental contexts, not just tissue growth [184]. In *D. melanogaster* oogenesis, for example, it is essential for establishing AP polarity in the oocyte as it regulates the expression of the downstream effector of Notch signalling, the gene *hindsight/pebbled* (*hnt*), which is required for posterior follicle cell maturation [184]. Orthologs of all the Hippo signalling related genes (i.e. Hippo signalling components, as well as up- and downstream factors) have been identified as being essential in *D. melanogaster* oogenesis (references in Additional file 1) and were transcribed by *P. aegeria*, with possibly two exceptions: *merlin* (*mer; ERM2*) and *mob as tumor suppressor* (*mats*, *mob1*) (Table 21). Merlin/ERM2 is a member of the band 4.1 protein superfamily and is characterised by a highly conserved FERM (Four.1 protein, Ezrin, Radixin, Moesin) domain involved in crosslinking the cell membrane and the actin cytoskeleton and so is thus important in localising proteins [184]. *Pararge aegeria* expressed a highly similar gene, *ERM1* (Table 9), which in *P. aegeria* shows a highly significant sequence similarity to *ERM2* (Table 9). In *D. melanogaster* ERM1 is important for Osk localisation [185], but clearly it cannot function in this way in *P. aegeria*, which lacks Osk. Likewise, *P. aegeria* appeared to express paralogs that are significantly similar to *mob1*; *mob2* and *mob4-like* (i.e. *preimplantation protein* in *B. mori*) (Table 21). The latter is most likely the Lepidopteran ortholog of *D. melanogaster mob1*.

**Table 21:** Growth regulation and Hippo pathway


*serine/threonine kinase 3-like (hippo; STE20)*(*hpo)*	Y	*expanded* (*ex)*	Y
*salvador* (*sav)*	Y	*merlin* (*mer; ERM2)*	N
*warts* (*wts)*	Y	*kibra;* CG33967 (*kibra)*	Y
*mob as tumor suppressor* (*mats; mob1)*	N	*yorkie; yap65-like protein* (*yki)*	Y
*mob-2* (*mob2)*	Y	*phosphatidylinositol 4-kinase alpha* (*PI4kIIIalpha)*	Y
*preimplantation protein; mps one binder kinase activator-like 4* (*mob4-like)*	Y	*bitesize; synaptotagmin-like* (*btsz)*	Y
*hindsight; pebbled* (*hnt)*	Y	*par-domain protein 1;* CG17888 (*pdp1)*	Y

Genes identified mainly from the *Drosophila melanogaster* literature involved in regulation of growth during oogenesis (including the Hippo pathway). Presence (Y) or absence (N) of orthologous transcripts in the *Pararge aegeria* transcriptome is indicated.

### Heat shock proteins and their control of protein abundance during oogenesis

Heat shock proteins (Hsps) provide a possible mechanism for environmental control of development in ovaries and as maternal effects. The transcription of genes encoding Hsps, or molecular chaperones in general, is not only regulated in response to various environmental factors (e.g. temperature), but is also essential during many developmental processes, including oogenesis. It is thought that Hsps are important for both developmental buffering and differentiation [72,186](further references in Additional file 1). The functional contexts in which Hsps operate are incredibly varied [186]. In *D. melanogaster*, for example, Hsp60C is essential in organising and maintaining cytoskeletal and cell adhesion components and thus for establishing AP and DV oocyte polarity [186], whilst Hsp70 affects border cell migration through its effects on the actin cytoskeleton [187]. A large number of genes encoding Hsps and related proteins have been described in a functional context during *D. melanogaster* oogenesis (references in Additional file 1) and orthologs of all of these were transcribed during *P. aegeria* ovarioles, often very abundantly (e.g. *heat shock protein cognate 3*, *hsc3*) (Tables 2 and 22; Additional file 2).

**Table 22:** Heat shock proteins


similar to *heat shock factor a2 (Bombyx mori)* (*hsf-2a)*	Y	*heat shock cognate protein 70; heat shock protein cognate 3* (*hsc70; hsc3; hsc70-3)*	Y
similar to *heat shock factor b (Bombyx mori)* (*hsfb)*	Y	*heat shock cognate protein 70cb* (*hsc70cb)*	Y
similar to *heat shock factor c (Bombyx mori)* (*hsfc)*	Y	*heat shock protein cognate 5* (*hsc5; hsp70-5)*	Y
*heat shock factor binding protein 1-like;* CG5446 (*hsfbp1; hsbpsb)*	Y	similar to *Bombyx mori heat shock protein 40 homolog DNAj-1* (*hsp40; DNAj)*	Y
*19.5 kDa heat shock protein (Bombyx mori)* (*19.5hsp)*	Y	*heat shock protein 60* (*hsp60)*	Y
*trap1 ; hsp90-like* (*trap1)*	Y	similar to *heat shock protein 68; heat shock protein 70-like* (*hsp70)*	Y
*(Bombyx mori) heat shock protein 1;* similar to *Drosophila lethal (2) essential for life* and *hsp27* (*hsp1)*	Y	*heat shock protein 83; heat shock protein 90* (*hsp90)*	Y
*(Bombyx mori small heat shock protein, shsp) - heat shock protein 19.9;* similar to *Drosophila lethal (2) essential for life* (*hsp19.9)*	Y	*endoplasmin; 94 kDa glucose-regulated protein;* similar to *Drosophila glycoprotein 93; heat shock protein 90 kDa beta member 1* (*gp93)*	Y
*(Bombyx mori small heat shock protein, shsp) - heat shock protein 20.1;* similar to *Drosophila lethal (2) essential for life* (*hsp20.1)*	Y	*hsc70/hsp90-organisng protein hop* (*hop)*	Y
*(Bombyx mori small heat shock protein, shsp) - heat shock protein 20.4;* similar to *Drosophila lethal (2) essential for life* (*hsp20.4)*	Y	CG11267*; heat shock 10kDa protein* (CG11267)	Y
*(Bombyx mori small heat shock protein, shsp) - heat shock protein 20.8;* similar to *Drosophila lethal (2) essential for life* (*hsp20.8)*	Y	CG1416*; activator of 90 kDa heat shock protein ATPase homolog; Bombyx mori bm44* (*bm44)*	Y
*(Bombyx mori small heat shock protein, shsp) - heat shock protein 23.7;* similar to *Drosophila lethal (2) essential for life* (*hsp23.7)*	Y	*RNA polymerase II 140kD subunit* (*rpII140)*	Y
*heat shock protein 21.4* (*hsp21.4)*	Y	*samui* (*samui)*	Y
*heat shock cognate protein 70–4; heat shock protein cognate 4* (*hsc70-4; hsc4)*	Y		

Genes encoding heat shock proteins (in ovaries and as maternal effects) and their control of protein abundance during oogenesis identified mainly from the *Drosophila melanogaster* literature. Presence (Y) or absence (N) of orthologous transcripts in the *Pararge aegeria* transcriptome is indicated.

### Ribosomal machinery needed for increased ovarian protein synthesis and early embryogenesis

Genes encoding ribosomal proteins, rRNA and other proteins involved in translation (e.g. RpA1) are among the most highly transcribed genes during Metazoan oogenesis, as large amounts of the translation machinery are needed both during oogenesis and by the developing embryo [188]. Just like Hsps, specific ribosomal proteins have been studied in a wide variety of functional contexts during *D. melanogaster* oogenesis and early embryogenesis (Tables 12 and 18; further references in Additional file 1). Ribosomal genes were also among the most highly transcribed in *P. aegeria* oogenesis (Table 2; Additional file 2).

### Immune defense and *Wolbachia* infection

Orthologs of the majority of the genes identified from the literature as being involved in immune response during oogenesis were also found to be expressed by *P. aegeria* and present as maternal transcripts in the oocytes (Table 23; Additional files 1 and 2). Apart from the aforementioned Toll innate immune defense pathway, which may have been co-opted for DV patterning of the embryo (Table 13), these include a large number of genes encoding Serpins (Table 23). *Drosophila melanogaster spn27A* (the ortholog of which is called *serpin-3* in *B. mori*), has been implicated in DV axis formation [120].

**Table 23:** Immune defense


*hemolin; p4* (*p4)*	Y	*MAPKK4* (*mkk4; MAPKK4)*	Y
*hemolin interacting protein; yippee* (*yip)*	Y	similar to *Bombyx mori clip domain serine protease 4;* similar to *manduca sexta hemolymph proteinase 17* (*bmclip4)*	Y
*yippee interacting protein 2* (*yip2)*	Y	similar to *Bombyx mori clip domain serine protease 11;* similar to *manduca sexta serine proteinase-like protein 1* (*bmclip11)*	Y
*cecropin A* (*cecA)*	Y	*transferrin* (*tf; tsf)*	Y
weak homology to *cecropin B* (*cecB)*	Y	*Ferritin 2 – light chain homolog* (*FER2-LCH)*	Y
homology to *Bombyx serpin-1* and *Drosophila spn4/42Da* (*srp1; spn4/42Da)*	Y	*Ferritin 1/3 – heavy chain homolog* (*FER1/3-HCH)*	Y
homology to *Bombyx serpin-2* and *Drosophila spn4/42Da* (*srp2; spn4/42Da)*	Y	*FK506-binding protein 2; FK506-binding protein 12 (in Bombyx mori)* (*FKBP12)*	Y
homology to *Bombyx serpin-3* and *Drosophila spn27A* (*srp3; spn27A)*	Y	*FK506-binding protein 1* (*FKBP39)*	Y
homology to *Bombyx serpin-4* and *Drosophila spn28D* (*srp4; spn28D)*	Y	weakly similar to *refractory to sigma p* (*ref(2)p)*	Y
homology to *Bombyx serpin-5* and *Drosophila spn77Ba* (*srp5; spn77Ba)*	Y	similar to *bmrelish1 and bmrelish2; nuclear factor nf-kappa-b p110 subunit isoform 1 or 2;* weakly similar to *Drosophila melanogaster relish* (*rel)*	Y
homology to *Bombyx serpin-6* and *Drosophila spn88Ea* (*srp6; spn88Ea)*	Y	*hemomucin* (*rrm5; hmu)*	Y
homology to *Bombyx serpin-10* and *Drosophila spn100a* (*srp10; spn100A)*	Y	*smt3 activating enzyme 2* (*sae2; sip2; uba2)*	Y
homology to *Bombyx serpin-11* and *Drosophila spn100A* (*srp11; spn100A)*	Y	*galactin; galactose specific c-type lectin* (*lectin-galc1)*	N
homology to *Bombyx serpin-13* and *Drosophila spn28d* (*srp13; spn28D)*	Y		

Genes identified mainly from the *Drosophila melanogaster* literature involved in immune defense during oogenesis. Presence (Y) or absence (N) of orthologous transcripts in the *Pararge aegeria* transcriptome is indicated.

The facultative reproductive parasite *Wolbachia* sp. is an endocytosymbiont in many arthropod species affecting oogenesis in a multitude of ways and the Bacterium is maternally transmitted [189-191]. In *D. mauritiana, Wolbachia* increases egg production by affecting the maintenance and division of germ-line stem cells [20], while in the wasp *Asobara tabida*, *Wolbachia* confers a reproductive advantage to the females by properly regulating apoptosis during oogenesis via its regulation of iron metabolism and *ferritin* expression [190,192]. However, in *D. melanogaster* highly infected females suffer from a range of oogenesis defects mediated via *grk* signalling [193]. *Pararge aegeria* females were also found to be infected with *Wolbachia,* but how this affects oogenesis in this species is at present not known. However, we did observe that the gene encoding an ortholog of the Ferritin 2 light chain protein (FER2-LCH) was amongst the most highly transcribed genes during *P. aegeria* oogenesis (Tables 2 and 23), but at present it is unknown whether this effect is due to *Wolbachia* or whether elevated expression levels are a normal part of female *P. aegeria* reproduction*.*

### Egg activation, ovulation, gene regulation in oviduct upon mating and maternal effect genes involved in fertilisation

As discussed elsewhere in this paper, after vitellogenesis both the *D. melanogaster* and the Lepidopteran oocyte are in a secondary meiotic arrest in metaphase I [60,194]. Unlike in Lepidoptera [60], egg activation in *D. melanogaster* is not triggered by the act of fertilisation, but due to the mechanical pressure experienced by the oocyte when moving from the ovary into the small and tight oviducts [194]. Egg activation involves eggshell modifications, resumption of meiosis, translation and subsequent degradation of maternal mRNAs, and cytoskeletal changes [194]. A small number of genes have been described as important in *D. melanogaster* in the latter stages of oogenesis in the general functional context of egg activation (references in Additional file 1). Orthologs for only around half of these were found in the *P. aegeria* transcriptome (Table 24), which may indicate observed differences in the mechanism of egg activation between the Lepidoptera and *D. melanogaster*. Among the genes found in the *P. aegeria* transcriptome is *wispy* (*fs(1)M19/wisp*) (Table 24). In *D. melanogaster* it is a maternal effect gene, encoding a GLD-2 family protein with polynucleotide adenylyltransferase activity and is essential for the oocyte-to-embryo transition [195]. The *D. melanogaster* Wisp protein is required for poly(A) tail elongation of *bcd*, *toll*, and *tor* transcripts upon egg activation. It is thus important for proper patterning of the embryo [195], but is also required to maintain a high level of active (phospho-) mitogen-activated protein kinases (MAPKs)[195]. *G*iven that *P. aegeria* females did not express *bcd* and *tor*, it remains to be investigated whether wisp is of any importance in patterning of the embryo.

**Table 24:** Egg activation


*cathepsin l-like cysteine protease; Bombyx cysteine protease; cysteine proteinase-1* (*bcp; cl; cp1)*	Y	*vacuolar proton atpase; vacuolar h+ atpase subunit 100–2* (*vha100-2)*	Y
*cathepsin b; cathepsin b-like cysteine proteinase* (*catb)*	Y	*h+ transporting atpase v0 subunit d; vacuolar h+ atpase subunit ac39-1* (*vhaac39-1)*	Y
*cathepsin d; aspartic protease* (*catd)*	Y	*vacuolar atp synthase subunit d; vacuolar h+ atpase subunit 36–1* (*mvd; vha36-1)*	Y
*cathepsin f-like cysteine protease;* CG12163 (*catf)*	Y	CG7899*; acid phosphatase 1* (*acph-1; ap)*	N
*ecdysteroid-phosphate phosphatase* (*EPPase)*	Y	*primo-1; acid phosphatase isoenzyme* (*primo-1)*	Y
*vacuolar proton atpase; vacuolar h+ atpase subunit 100–1* (*mva; v100; vha100-1)*	Y		

Genes identified mainly from the *Drosophila melanogaster* literature involved in egg activation, ovulation, gene regulation in oviduct upon mating and maternal effect genes involved in fertilisation. Presence (Y) or absence (N) of orthologous transcripts in the *Pararge aegeria* transcriptome is indicated.

## Conclusions

A large proportion of the genes currently described in the literature as being essential during insect oogenesis (in particular *D. melanogaster* oogenesis) were transcribed by *P. aegeria* and transcripts were transferred to the oocytes. As this was an ovarian transcriptome study, the precise functional context in which these genes were transcribed has not been identified. Differences in the functional context in which particular genes are expressed are to be expected compared to model organisms such as *D. melanogaster* and even *B. mori*. What is perhaps more revealing, however, is the absence of certain transcripts in the database, in particular where these transcripts concern paradigms of maternal regulation for various aspects of early insect embryogenesis [3-5,24]. *Pararge aegeria* differed most significantly from *D. melanogaster* (and quite a number of other insect species), both in terms of stem cell maintenance or differentiation in the germarium and in establishing (and maintaining) polarity along AP, DV and at the termini of the oocyte. In particular, although *Pararge aegeria* females expressed an ortholog of a *spi*/*krn*-like EGF ligand and possibly its receptor, many components of the EGF pathway involved in patterning of the axes in *D. melanogaster* embryos*,* as well as *pipe* and *mirror*, were not expressed. This may either suggest that there is not much evidence for a significant role of EGF signalling in establishing *P. aegeria* oocyte polarity, or that its functional role and genes involved is divergent from other insects. This requires further study, as well as the functional role and significance of Dpp and Notch signalling in this context.

Although the more derived species such as *B. mori* within the Ditrysia are argued to be long germ band-like [94], it is more appropriate to describe them as intermediate germ band [53,54], as they have a very unusual preblastoderm stage. Like *D. melanogaster*, cleavage in *B. mori* and the butterfly *Pieris rapae* is superficial but nuclear migration to the periphery of the oocyte and subsequent cellularisation occurs in an anterior to posterior gradient, after which they display long germ band characteristics [60]. It is very likely that this has a bearing on maternal effect gene expression regulating axes patterning after oocyte polarity has been established during the pre-vitellogenic stages in Ditrysia compared to *D. melanogaster,* and this could be reflected in the gene expression data presented in this study (e.g. the absence of maternal expression of *hb)*. Although progress has been made in investigating *B. mori* embryonic patterning [53,54], how polarity is established during oogenesis in Ditrysia and in the Lepidoptera as a whole is not known. This needs further investigation, and *P. aegeria* may prove an ideal model these future studies.

Unfortunately, maternal effect gene expression and regulation have received significantly less research attention in Lepidoptera compared to vitellogenesis, choriogenesis and reproductive physiology [8]. This is reflected in the discussion of the results in this paper. Although the latter aspects of oogenesis are well suited to studies of reproductive output under a variety of environmental conditions, many of the genes discussed in this study highlight the interconnectedness of all stages during oogenesis, for example eggshell production and oocyte polarity. Furthermore, key candidate genes that have the potential to play an important role in transgenerational maternal effects have been identified. Among these are genes encoding heat shock proteins and proteins involved in chromatin remodelling.

This study has taken a much-needed first step in determining the conserved and divergent elements of the butterfly oogenesis GRN (including maternal regulation of embryonic patterning) and establishes *P. aegeria* as an eco-evo-devo model system for the study of butterfly oogenesis. In order to fully unscramble butterfly oogenesis, an investigation of the spatio-temporal expression patterns of the genes discussed in this study, as well as establishment of their function, is required. Further studies are also required to establish the function and expression patterns of the uncharacterised contigs identified in this study, which make up 30% of the total contigs found, and are undoubtedly composed of genes that are of high importance in butterfly oogenesis.

## Methods

### Butterfly rearing and sample collection

As butterflies were used in this study, no ethical approval was required. Eggs were collected from a large outbred laboratory population of *P. aegeria* (kept at 300–400 individuals per generation). This population originated from a woodland population from the south of Belgium (St. Hubert; established from 50 eggs) and by the time of the experiment, the butterflies had been reared in the laboratory for 10 generations. Newly hatched larvae were placed on potted host plants (4 larvae per plant) of *Poa trivialis* L. with access to *ad libitum* food and were reared until eclosion in a climate room under a regime (24±0.3°C, LD 16:8) that promotes direct development (i.e. no diapause). On the day of eclosion (i.e. day −1, between 9 and 12 h) females from this laboratory stock placed individually in netted cages (0.5 m^3^) along with a potted *P. trivialis* plant for oviposition and an artificial flower containing a 10% honey solution [55]. Later the same day (between 13.00 and 16.00 h) a virgin male was introduced to the cage and the mating pair was left undisturbed for 24 h.

Eggs from 50 mated 4-day old females were collected within 20 minutes of being laid, which is well before the onset of cleavage and thus early embryogenesis in butterflies [60]. The eggs were placed immediately in 1ml TRI-Reagent (Sigma-Aldrich, Dorset, UK) and homogenised thoroughly. Furthermore, 2 mated females aged 4 days were sacrificed by severing the nerve cord, after which the abdomen was removed and the ovaries dissected out in ice-cold PBS (1×), with dissection taking no longer than 15 minutes to avoid RNA degradation. The ovaries were pooled and likewise homogenised immediately in 1ml TRI-Reagent.

### RNA extraction and quality control

The homogenate (both of eggs and ovarioles/ovary) was first centrifuged at 13000g for 10min primarily to remove the yolk, after which the supernatant was vortexed with 200μl of chloroform. Phases were separated at 13000g for 15min at room temperature. The aqueous phase was removed and precipitated in 0.5ml isopropanol [196]. The RNA samples were further purified using the RNeasy Mini Kit and re-eluted in 30μl nuclease-free water, following the manufacturer’s instructions (Qiagen, Hilden, Germany). Preliminary yield and quality for each RNA extraction were assayed using a Nanodrop, while RNA integrity was verified using the Agilent BioAnalyzer 2100 PicoRNA Chip (Agilent Technologies, Winnersh, UK) (Additional file 10).

### *De novo* transcriptome assembly

*Pararge aegeria* egg and ovary RNA was sequenced by Source BioScience (Nottingham, UK) using Illumina short read RNA-Seq technology. Both total RNA samples went through polyA selection, fragmentation and double stranded cDNA conversion to produce two separate libraries (300bp insert size) in accordance with the Illumina mRNA-seq library preparation protocol (Illumina, San Diego, USA). Sequencing was performed on the Illumina Genome Analyzer IIx platform with one flowcell lane allocated to each library. A total of 61,400,070 single-reads of 38 base pairs (bp) in length were obtained from the ovary and egg flowcell lanes (31,836,256 and 29,563,814 reads for ovary and egg samples respectively) which were pooled to produce a *de novo* assembly in CLC Genomics Workbench v4.0 (CLC bio, Aarhus, Denmark) using the default settings for short read data (automatic word and bubble size) [197]. The assembly generated 25266 contigs (Additional file 2) of an average length of 535bp (N50=671bp), 41.06% GC content and an estimated average coverage of 124× per nucleotide.

The RNA-seq data was analysed by FASTQC on the Galaxy platform [198,199]. Adaptor dimer or overruns in the reads (stretches of sequence matching the library preparation primers/adaptors) were trimmed from both egg and ovary data sets using CLC Genomics Workbench. Furthermore, the sequences were trimmed down to 25 bp from the 5’ end and sequencing artefacts discarded using the FASTX-Toolkit on Galaxy. Subsequently, the trimmed reads were mapped using default parameters against the *de novo* assembly using TopHat on the Galaxy server [200]. FPKM values were estimated from the TopHat output using Cufflinks [201] with quartile normalisation and multi read correct enabled. The estimates were limited to a reference general feature format file containing locations of the predicted coding regions from the automated annotation if available.

### Annotation

The 25,266 contigs generated by the *de novo* assembly (Additional file 2) were processed through a similarity-based annotation workflow. Open reading frames (ORF) over 200 bp were identified and extracted with the EMBOSS tool “getorf” in Galaxy. The GC content increased to 42.23% when limited to possible coding regions. The predicted ORF and contig sequences were then processed through different BLAST strategies to provide the most suitable annotation possible (Additional files 11 and 12). The alpha group compared the predicted ORF sequences against protein databases to identify complete or highly conserved transcripts. The beta group compared the full contigs against protein databases to identify incomplete or out of frame transcripts. Sequences not identified in the alpha and beta group were compared further against nucleic acid coding sequences (delta) and finally the whole nucleotide database (zeta). Each search strategy was attributed a different rank, ranging from A to I. Identity was inferred based on similarity to the top ranking hit. Similarity scores (SS) were assigned to each hit based on the bitscore (S’), number of positives in each alignment (P) and original contig length (L). Similarity score was calculated using the formula:

$$\mathrm{SS}=S^{\prime }\frac{P}{L}$$

Effectively this required hits with higher bitscores to also have good query coverage and positive matches. Any hit attaining an SS below 18 (lower SS threshold) was discarded from each rank, using the next best hit (which may be in a lower rank or group) (Additional file 11). Hits were sorted based on group, positives, rank and SS to determine the top hit that would be used to infer the nature of each sequence. Similarity scores also allowed an initial indication of possible homology; SS above the upper threshold (>/=40) were considered High, those above the lower SS threshold (>/=18) were considered Mild and any others were considered Low. Any hit with a bitscore below 40 was excluded from inferring any possible identity or homology (Additional files 12 and 13).

The output from the automated annotation was checked manually for any errors (Additional file 2). Furthermore, using FlyBase [62] and SilkBase [63] as a starting point, a comprehensive literature search was conducted to identify those genes that have been studied in the context of insect oogenesis and maternal regulation of early embryogenesis (1035 genes, of which 994 have been studied in *D. melanogaster*; fully referenced in Additional file 1). For a further 56 genes functionality during oogenesis can be inferred, but their expression during oogenesis has not always been verified experimentally. The presence or absence of orthologous *P. aegeria* transcripts in both the oocyte and the ovarioles was verified for each of the 1091 genes and these transcripts were further annotated manually (indicated as such in Additional file 2).

The final BLAST results (1 top hit per sequence) used for annotation, including those genes annotated manually, were used as input in the BLAST2GO software [202] and assigned with Gene Ontology (GO) terms where possible. To help provide an overview of the GO based on the BLAST results, the GO terms were condensed using the generic GO Slim subset.

### Transcript abundance and qPCR of genes involved in oogenesis and maternal regulation of early embryogenesis

For of a subset of 19 genes the expression in the ovarioles and the presence of transcripts in the oocyte were confirmed further by means of RT-qPCR (Additional file 3). For both ovary and oocyte, cDNA was generated from 500 – 1000 ng of RNA using the Verso RT Kit (Thermo Fisher, Surrey, UK). The reverse transcriptions were primed by a 3:1 mix of random hexamers:oligo-dT taking place in 20μl total volume reactions at 42**°**C for 30 min after an initial 5 min denaturation step at 70**°**C. Negative reverse transcription (NRT) controls were run in parallel without both Verso RT enzyme mix and primers. A final heat deactivation at 95**°**C for 2 min was also implemented to deactivate the RT enhancer. The resulting cDNA was stored at −20**°**C.

For the qPCR stage, suitable primer pairs were selected automatically using the online Primer3+ primer design service and tested *in-silico* via the Integrated DNA Technologies online structure prediction package (Oligo Analyzer). Only those primers exhibiting the best stability were selected. Each primer pair was tested on a 3-step 5-fold dilution series of the ovary cDNA in triplicate, which enabled the primer pair efficiencies to be determined using the CFX Manager software (Bio-Rad Laboratories, California, USA). Primers with adequate efficiency (>65%) were then used for investigating the transcript abundance in the egg and ovary cDNA (Additional file 3).

All qPCR runs were performed on the CFX96 Real-Time PCR Detection System (Bio-Rad) on white 96-well plates in ABsolute Blue qPCR SYBR Green Mastermix (Thermo Fisher, Surrey, UK) with the recommended amount of ROX reference dye (Additional file 14). Test samples were measured in triplicate, while no template controls (NTC) and NRTs were present in duplicate on each plate. The CFX96 data generated was recorded by the CFX manager program using automatic threshold determination. The quantification cycle (Cq) values are listed in Additional file 4.

Relative transcript abundance (i.e. ovary versus egg) was used to reveal whether any individual transcript was used as a maternal effect gene transcript or was merely necessary for oocyte production. Relative transcript abundance in the ovaries and eggs were obtained using the relative expression software tool REST v2.0.13.0 software package [203], which used the 3 available reference genes to normalise the measurements obtained from the egg and ovary derived cDNA (Additional file 5).

The number of reads mapping to a transcript of a particular gene in RNA-seq data was argued to be correlated linearly with the number of transcripts of that gene [204]. Rather than using read counts, it is considered to be more appropriate to use a corrected relative value, taking transcript length and total number of mapped reads into account [204]. Cufflinks generated such corrected values, the FPKM values, which can be used for the reliable determination of transcript abundance for each of the genes discussed in this study (Additional file 2). In fact, for the 22 genes in the *P. aegeria* transcriptome investigated by means of qPCR, transcript abundance calculated on the basis of Cq values by means of the methods described in [205] showed significant positive correlation with FPKM values in the combined oocyte and ovary transcriptome (Pearson regression, with null hypothesis that correlation is >0: t_41_ = 2.37, P = 0.011; Additional file 6).

### Annotated contigs and accession numbers of raw data

The sequence read data reported in this manuscript have been deposited in the NCBI Sequence Read Archive and are available under the accession numbers SRR771147 (ovarian reads) and SRR772253 (oocyte reads). Additional file 15 provides the fasta format sequences of the assembled contigs, including the suggested annotated names (top BLAST results as well as information on the manual annotation listed in Additional file 2). Additional file 2 provides information on the start and end of the coding regions in the contigs.

## Abbreviations

GRN: Gene Regulatory Network; eco-evo-devo: Ecological evolutionary development; AP: Anterior-posterior; DV: Dorso-ventral; RNA-seq: RNA-sequencing; RNP: Ribonucleoprotein; RTK: Receptor Tyrosine Kinase; CDK: Cyclin-dependent kinase; SC: Synaptonemal Complex; RN: Recombination Nodules; IRS: Insulin Receptor Substrate; 20E: 20-hydroxy-ecdysone; JH: Juvenile Hormone; FPKM: Fragments Per Kilobase of exon per Million of fragments mapped; ORF: Open Reading Frame; SS: Similarity Score; GO: Gene Ontology; RT-qPCR: Real-time reverse transcription quantitative polymerase chain reaction; NRT: Negative reverse transcription; NTC: No template control

## Competing interests

The authors declare that they have no competing interests.

## Authors’ contributions

JMC collected and analysed RT-qPCR data, designed the automatic annotation pipeline, performed bioinformatic analyses, and co-wrote the manuscript. SCB assisted in RT-qPCR study design and data collection. RP and DRFC prepared RNA samples for RNA-seq. AC performed phylogenetic analyses of *nanos*. JT assisted in manual annotation of the transcriptome. MG and CJB designed and supervised the study, performed the manual annotation of the transcriptome, and co-wrote the manuscript. All authors have provided comments on earlier drafts of the manuscript and approved the final version of the manuscript for publication.

## Supplementary Material

Additional file 1 **Oogenesis genes.** Contains a tabulated and fully referenced list of genes identified from the literature, which have been studied in the context of insect oogenesis and maternal regulation of early embryogenesis. The vast majority of papers concern the fruitfly *Drosophila melanogaster* and the silkmoth *Bombyx mori*. Many genes have multiple functions during oogenesis, but to avoid repetition, and keep the size of the Table manageable, each gene has been listed only once in the functional context for which it is probably best known. Referencing has been kept to a minimum, highlighting key papers and databases. Hyperlinks have been provided for almost all of the genes listed, which will provide full database information on their myriad functions and further references. Presence (Y) or absence (N) of orthologs in the *Pararge aegeria* combined oocyte and ovariole transcriptome are indicated. Click here for file

Additional file 2 **Annotation summary of the combined transcriptome of the *Pararge aegeria *ovarioles and oocytes.** Details the results of both automatic and manual annotation of 25266 contigs. Egg and ovary FPKM values are given for each contig. Each column contains a pop-up comment box with an explanation of the column contents. Click here for file

Additional file 3 **Overview of the primer pair properties and performance in qPCR conditions.** Gives an overview of the forward and reverse primers designed for qPCR of a set of 19 oogenesis and 3 housekeeping genes. Efficiency and R^2^ values are provided for each of the primers. Click here for file

Additional file 4 **Data generated by the CFX96 qPCR experiments.** Details the measurements from a total of 8 96-well white plates. Cq are given for each gene of interest or reference gene. Click here for file

Additional file 5 **Relative Abundance Data generated by REST.** Gives the results from using REST v2.0.13.0 to process Cq measurements and efficiencies in order to estimate relative transcript abundance, and thus compare relative transcript abundance between ovaries and eggs. Click here for file

Additional file 6 **Transcript abundance: Cq - FPKM correlation.** Provides the results of the correlation analyses between two measures of transcript abundance: Cq and FPKM-values. Click here for file

Additional file 7 **Mapping of raw RNA-seq reads against *egfr *and *wingless *coding sequences as predicted from the draft *Pararge aegeria *genome.** Provides the complete *egfr* and *wingless* (*wg*) CDS fasta information from our unpublished *P. aegeria* genome. Furthermore, raw RNA-seq reads were mapped against these sequences and coverage determined. Click here for file

Additional file 8 **Phylogenetic analysis of Nanos.** Provides a phylogenetic analysis of insect Nanos protein sequences. Click here for file

Additional file 9 **Phylogenetic analyses of both chorion and minor yolk proteins in Lepidoptera.** Provides the phylogenetic analyses of both chorion and minor yolk proteins in Lepidoptera. Click here for file

Additional file 10 **Oocyte and ovarian RNA quality.** Provides the Agilent BioAnalyzer Electropherograms detailing oocyte and ovarian RNA quality prior to cDNA synthesis. Click here for file

Additional file 11 **Filtering of BLAST hits in the automated annotation.** Provides a visualisation of the similarity score distribution and thresholds applied in the automated annotation of the *P. aegeria* transcriptome. Click here for file

Additional file 12 **Automated annotation based on different BLAST Strategies.** Provides a summary of the automated annotation method, detailing the different queries. Click here for file

Additional file 13 **Distribution of similarity classes across BLAST sources.** Provides details regarding the number of *Pararge aegeria* contigs in each of the similarity classes, according to the BLAST strategy used in the automated annotation. Click here for file

Additional file 14 **Thermocycler and qPCR reaction setup.** Provides details regarding the reaction conditions and thermocycler programming parameters for successful qPCR amplification for each qPCR measurement reported in this study. Click here for file

Additional file 15 **Combined annotated ovarian and oocyte transcriptome of *Pararge aegeria.*** Provides the fasta format sequences of the contigs, which in Additional file 2 had a YES in the SubmitFlag column (i.e. to be submitted to NCBI TSA). Suggested annotated names are given on the basis of the BLAST results listed in Additional file 2, and as described in the main text. The start and end of the open reading frames can be found in the final two columns of Additional file 2. Click here for file

## References

[B1] Ewen-Campen B Srouji JR Schwager EE Extavour CG *Oskar* predates the evolution of germ plasm in insects Curr Biol 2012 22 2278 2283 10.1016/j.cub.2012.10.019 23122849

[B2] Berg GJ Gassner G Fine structure of the blastoderm embryo of the pink bollworm, *Pectinophora Gossypiella* (saunders) (lepidoptera: Gelechiidae) Int J Insect Morphol Embryol 1978 7 81 105 10.1016/S0020-7322(78)80017-8

[B3] Lynch JA Ozuak O Khila A Abouheif E Desplan C Roth S The phylogenetic origin of *oskar* coincided with the origin of maternally provisioned germ plasm and pole cells at the base of the Holometabola PLoS Genet 2011 7 e1002029 10.1371/journal.pgen.1002029 21552321 PMC3084197

[B4] Lynch JA Roth S The evolution of dorsal–ventral patterning mechanisms in insects Genes Dev 2011 25 107 118 10.1101/gad.2010711 21245164 PMC3022256

[B5] Rosenberg MI Lynch JA Desplan C Heads and tails: evolution of antero-posterior patterning in insects Biochim Biophys Acta 2009 1789 333 342 10.1016/j.bbagrm.2008.09.007 18976722 PMC2700975

[B6] Ziegler R Van Antwerpen R Lipid uptake by insect oocytes Insect Biochem Mol Biol 2006 36 264 272 10.1016/j.ibmb.2006.01.014 16551540

[B7] Ramaswamy SB Shu SQ Park YI Zeng FR Dynamics of juvenile hormone-mediated gonadotropism in the Lepidoptera Arch Insect Biochem Physiol 1997 35 539 558 10.1002/(SICI)1520-6327(1997)35:4<539::AID-ARCH12>3.0.CO;2-B

[B8] Telfer WH Egg formation in Lepidoptera J Insect Sci 2009 9 1 21 20050770 10.1673/031.009.5001 PMC3011924

[B9] Tufail M Takeda M Insect vitellogenin/lipophorin receptors: Molecular structures, role in oogenesis, and regulatory mechanisms J Insect Physiol 2009 55 88 104 10.1016/j.jinsphys.2008.11.007 19071131

[B10] Gibbs M Van Dyck H Karlsson B Reproductive plasticity, ovarian dynamics and maternal effects in response to temperature and flight in *Pararge aegeria* J Insect Physiol 2010 56 1275 1283 10.1016/j.jinsphys.2010.04.009 20416319

[B11] Gibbs M Breuker CJ Van Dyck H Flight during oviposition reduces maternal egg provisioning and influences offspring development in *Pararge aegeria* (L.) Physiol Entomol 2010 35 29 39 10.1111/j.1365-3032.2009.00706.x

[B12] Rotem K Agrawal AA Kott L Parental effects in *Pieris rapae* in response to variation in food quality: adaptive plasticity across generations? Ecol Entomol 2003 28 211 218 10.1046/j.1365-2311.2003.00507.x

[B13] Skora AD Spradling AC Epigenetic stability increases extensively during Drosophila follicle stem cell differentiation Proc Natl Acad Sci 2010 107 7389 7394 10.1073/pnas.1003180107 20368445 PMC2867689

[B14] Li X Han Y Xi R Polycomb group genes *Psc* and *Su(z)2* restrict follicle stem cell self-renewal and extrusion by controlling canonical and noncanonical Wnt signaling Genes Dev 2011 24 933 20439432 10.1101/gad.1901510 PMC2861192

[B15] McCall K Eggs over easy: cell death in the *Drosophila* ovary Dev Biol 2004 274 3 14 10.1016/j.ydbio.2004.07.017 15355784

[B16] Terashima J Takaki K Sakurai S Bownes M Nutritional status affects 20-hydroxyecdysone concentration and progression of oogenesis in Drosophila melanogaster J Endocrinol 2005 187 69 79 10.1677/joe.1.06220 16214942

[B17] Xie T Spradling AC A niche maintaining germ line stem cells in the *Drosophila* ovary Science 2000 290 328 330 10.1126/science.290.5490.328 11030649

[B18] Dansereau DA Lasko P The development of germline stem cells in *Drosophila* Methods Mol Biol 2008 450 3 26 10.1007/978-1-60327-214-8_1 18370048 PMC2729445

[B19] Neumuller RA Betschinger J Fischer A Bushati N Poernbacher I Mechtler K Cohen SM Knoblich JA Mei-P26 regulates microRNAs and cell growth in the *Drosophila* ovarian stem cell lineage Nature 2008 454 241 245 10.1038/nature07014 18528333 PMC2988194

[B20] Fast EM Toomey ME Panaram K Desjardins D Kolaczyk ED Frydman HM *Wolbachia* enhance *Drosophila* stem cell proliferation and target the germline stem cell niche Science 2011 334 990 992 10.1126/science.1209609 22021671 PMC4030408

[B21] Bastock R St Johnston D *Drosophila* oogenesis Curr Biol 2008 18 R1082 R1087 10.1016/j.cub.2008.09.011 19081037

[B22] Archambault V Zhao X White-Cooper H Carpenter ATC Glover DM Mutations in *Drosophila Greatwall/Scant* reveal its roles in mitosis and meiosis and interdependence with polo kinase PLoS Genet 2007 3 e200 10.1371/journal.pgen.0030200 17997611 PMC2065886

[B23] Wilson MJ Abbott H Dearden PK The evolution of oocyte patterning in insects: multiple cell-signaling pathways are active during honeybee oogenesis and are likely to play a role in axis patterning Evol Dev 2011 13 127 137 10.1111/j.1525-142X.2011.00463.x 21410869

[B24] Lynch JA Peel AD Drechsler A Averof M Roth S EGF Signaling and the Origin of Axial Polarity among the Insects Curr Biol 2010 20 11 1042 1047 10.1016/j.cub.2010.04.023 20471269 PMC2902724

[B25] Roth S Lynch JA Symmetry Breaking During *Drosophila* Oogenesis Cold Spring Harb Perspect Biol 2009 1 2 a001891 10.1101/cshperspect.a001891 20066085 PMC2742093

[B26] Nijhout FH Insect hormones 1994 New Jersey: Princeton University Press

[B27] Riddiford LM Effects of juvenile hormone on the programming of postembryonic development in eggs of the silkworm, *Hyalophora cecropia* Dev Biol 1970 22 249 263 10.1016/0012-1606(70)90153-3 5424979

[B28] Orth AP Tauchman SJ Doll SC Goodman WG Embryonic expression of juvenile hormone binding protein and its relationship to the toxic effects of juvenile hormone in *Manduca sexta* Insect Biochem Mol Biol 2003 33 1275 1284 10.1016/j.ibmb.2003.06.002 14599499

[B29] Khila A Abouheif E Evaluating the role of reproductive constraints in ant social evolution Philos Trans R Soc Lond B Biol Sci 2010 365 617 630 10.1098/rstb.2009.0257 20083637 PMC2817144

[B30] Wheeler D The role of nourishment in oogenesis Annu Rev Entomol 1996 41 407 431 10.1146/annurev.en.41.010196.002203 15012335

[B31] Uller T Developmental plasticity and the evolution of parental effects Trends Ecol Evol 2008 23 432 438 10.1016/j.tree.2008.04.005 18586350

[B32] Khila A Abouheif E Reproductive constraint is a developmental mechanism that maintains social harmony in advanced ant societies Proc Natl Acad Sci 2008 105 17884 17889 10.1073/pnas.0807351105 19004767 PMC2584687

[B33] Rossiter MC Maternal effects generate variation in life history: consequences of egg weight plasticity in the Gypsy Moth Funct Ecol 1991 5 386 393 10.2307/2389810

[B34] Ginzburg LR Taneyhill DE Population cycles of forest Lepidoptera - A maternal effect hypothesis J Anim Ecol 1994 63 79 92 10.2307/5585

[B35] St Johnston D Nüsslein-Volhard C The origin of pattern and polarity in the *Drosophila* embryo Cell 1992 68 201 220 10.1016/0092-8674(92)90466-P 1733499

[B36] Munn K Steward R The anterior-posterior and dorsal-ventral axes have a common origin in *Drosophila melanogaster* Bioessays 1995 17 920 922 10.1002/bies.950171104 8526885

[B37] Christians E Davis AA Thomas SD Benjamin IJ Embryonic development - Maternal effect of Hsf1 on reproductive success Nature 2000 407 693 694 10.1038/35037669 11048707

[B38] Yatsu J Hayashi M Mukai M Arita K Shigenobu S Kobayashi S Maternal RNAs encoding transcription factors for germline-specific gene expression in *Drosophila* embryos Int J Dev Biol 2008 52 913 923 10.1387/ijdb.082576jy 18956321

[B39] Gilbert SF The morphogenesis of evolutionary developmental biology Int J Dev Biol 2003 47 467 477 14756322

[B40] Roff DA Life history evolution 2002 Sunderland, Mass: Sinauer

[B41] Johnson NA Porter AH Toward a new synthesis: population genetics and evolutionary developmental biology Genetica 2001 112–113 45 58 11838782

[B42] Jenner RA Wills MA The choice of model organisms in evo-devo Nat Rev Genet 2007 8 311 319 10.1038/nrg2062 17339879

[B43] Springer P Boggs CL Resource allocation to oocytes - heritable variation with altitude in *Colias philodice eriphyle* (Lepidoptera) Am Nat 1986 127 252 256 10.1086/284483

[B44] Gibbs M Van Dyck H Breuker CJ Development on drought-stressed host plants affects life history, flight morphology and reproductive output relative to landscape structure Evol Appl 2012 5 66 75 10.1111/j.1752-4571.2011.00209.x PMC3353328 25568030

[B45] Gibbs M Van Dyck H Reproductive plasticity, oviposition site selection, and maternal effects in fragmented landscapes Behav Ecol Sociobiol 2009 64 1 11 10.1007/s00265-009-0849-8

[B46] Jervis MA Boggs CL Ferns PN Egg maturation strategy and survival trade-offs in holometabolous insects: a comparative approach Biol J Linn Soc 2007 90 293 302 10.1111/j.1095-8312.2007.00721.x

[B47] Papanicolaou A Gebauer-Jung S Blaxter ML Owen McMillan W Jiggins CD ButterflyBase: a platform for lepidopteran genomics Nucleic Acids Res 2008 36 D582 D587 17933781 10.1093/nar/gkm853 PMC2238913

[B48] Wheat CW Fescemyer HW Kvist J Tas EVA Vera JC Frilander MJ Hanski I Marden JH Functional genomics of life history variation in a butterfly metapopulation Mol Ecol 2011 20 1813 1828 10.1111/j.1365-294X.2011.05062.x 21410806

[B49] Beldade P Rudd S Gruber JD Long AD A wing expressed sequence tag resource for *Bicyclus anynana* butterflies, an evo-devo model BMC Genomics 2006 7 130 10.1186/1471-2164-7-130 16737530 PMC1534037

[B50] Zhan S Merlin C Boore JL Reppert SM The monarch butterfly genome yields insights into long-distance migration Cell 2011 147 1171 1185 10.1016/j.cell.2011.09.052 22118469 PMC3225893

[B51] Consortium THG Butterfly genome reveals promiscuous exchange of mimicry adaptations among species Nature 2012 487 94 98 22722851 10.1038/nature11041 PMC3398145

[B52] O'Neil S Dzurisin J Carmichael R Lobo N Emrich S Hellmann J Population-level transcriptome sequencing of nonmodel organisms *Erynnis propertius* and *Papilio zelicaon* BMC Genomics 2010 11 310 10.1186/1471-2164-11-310 20478048 PMC2887415

[B53] Nakao H Anterior and posterior centers jointly regulate *Bombyx* embryo body segmentation Dev Biol 2012 371 293 301 10.1016/j.ydbio.2012.08.029 22975228

[B54] Nakao H Matsumoto T Oba Y Niimi T Yaginuma T Germ cell specification and early embryonic patterning in *Bombyx mori* as revealed by *nanos* orthologues Evol Dev 2008 10 546 554 10.1111/j.1525-142X.2008.00270.x 18803773

[B55] Gibbs M Breuker CJ Hesketh H Hails R Van Dyck H Maternal effects, flight versus fecundity trade-offs, and offspring immune defence in the Speckled Wood butterfly, *Pararge aegeria* BMC Evol Biol 2010 10 345 10.1186/1471-2148-10-345 21067561 PMC2993718

[B56] Karlsson B Variation in egg weight, oviposition rate and reproductive reserves with female age in a natural population of the speckled wood butterfly, *Pararge aegeria* Ecol Entomol 1987 12 473 476 10.1111/j.1365-2311.1987.tb01029.x

[B57] Berger D Olofsson M Friberg M Karlsson B Wiklund C Gotthard K Gilburn A Intraspecific variation in body size and the rate of reproduction in female insects - adaptive allometry or biophysical constraint? J Anim Ecol 2012 81 6 1244 1258 10.1111/j.1365-2656.2012.02010.x 22702372

[B58] Wickman PO Wiklund C Territorial defense and its seasonal decline in the Speckled Wood Butterfly (*Pararge aegeria)* Anim Behav 1983 31 1206 1216 10.1016/S0003-3472(83)80027-X

[B59] Karlsson B Feeding habits and change of body composition with age in three Nymphalid butterfly species Oikos 1994 69 224 230 10.2307/3546142

[B60] Kobayashi Y Tanaka M Ando H Kristensen NP Chapter 19: Embryology Lepidoptera, moths and butterflies: volume 2 - morphology, physiology and development 2003 Berlin: Walter de Gruyter 495 544

[B61] Regier JC Friedlander T Leclerc RF Mitter C Wiegmann BM Goldsmith MR, Wilkins AS Lepidopteran phylogeny and applications to comparative studies of development Molecular model systems in Lepidoptera 1995 Cambridge: Cambridge University Press 107 137

[B62] FlyBase http://www.flybase.org

[B63] SilkBase http://silkbase.ab.a.u-tokyo.ac.jp

[B64] Gelbart WM Emmert DB FlyBase high throughput expression pattern data Beta Version 2010 Flybase ID: FBrf0212041

[B65] Fisher B Weiszmann R Frise E Hammonds A Tomancak P Beaton A Berman B Quan E Shu S Lewis S Rubin G Barale C Laguertas E Quinn J Ghosh A Hartenstein V Ashburner M Celniker S BDGP insitu homepage 2012 http://flybase.org/reports/FBrf0219073.html

[B66] Roth S Neuman-Silberberg FS Barcelo G Schüpbach T Cornichon and the EGF receptor signaling process are necessary for both anterior-posterior and dorsal-ventral pattern formation in *Drosophila* Cell 1995 81 967 10.1016/0092-8674(95)90016-0 7540118

[B67] Galasso A Pane LS Russo M Grimaldi MR Verrotti AC Gigliotti S Graziani F *dSTAM* expression pattern during wild type and mutant egg chamber development in *D. melanogaster* Gene Expr Patterns 2007 7 730 737 10.1016/j.modgep.2007.06.004 17664083

[B68] Mesilaty-Gross S Reich A Motro B Wides R The *Drosophila STAM* gene homolog is in a tight gene cluster, and its expression correlates to that of the adjacent gene *ial* Gene 1999 231 173 186 10.1016/S0378-1119(99)00053-0 10231582

[B69] Song X Xie T *Wingless* signaling regulates the maintenance of ovarian somatic stem cells in *Drosophila* Development 2003 130 3259 3268 10.1242/dev.00524 12783796

[B70] Forbes AJ Spradling AC Ingham PW Lin H The role of segment polarity genes during early oogenesis in Drosophila Development 1996 122 3283 3294 8898240 10.1242/dev.122.10.3283

[B71] Xie T Spradling AC *Decapentaplegic* is essential for the maintenance and division of germline stem cells in the *Drosophila* ovary Cell 1998 94 251 260 10.1016/S0092-8674(00)81424-5 9695953

[B72] Funaguma S Hashimoto S Suzuki Y Omuro N Sugano S Mita K Katsuma S Shimada T SAGE analysis of early oogenesis in the silkworm, *Bombyx mori* Insect Biochem Mol Biol 2007 37 147 154 10.1016/j.ibmb.2006.11.001 17244543

[B73] Wrana JL Tran H Attisano L Arora K Childs SR Massague J O'Connor MB Two distinct transmembrane serine/threonine kinases from *Drosophila melanogaster* form an activin receptor complex Mol Cell Biol 1994 14 944 950 8289834 10.1128/mcb.14.2.944-950.1994 PMC358449

[B74] Liu Z Matsuoka S Enoki A Yamamoto T Furukawa K Yamasaki Y Nishida Y Sugiyama S Negative modulation of bone morphogenetic protein signaling by Dullard during wing vein formation in *Drosophila* Dev Growth Differ 2011 53 822 841 10.1111/j.1440-169X.2011.01289.x 21790556

[B75] Chen Y Schüpbach T The role of brinker in eggshell patterning Mech Dev 2006 123 395 406 10.1016/j.mod.2006.03.007 16707253

[B76] Shravage BV Altmann G Technau M Roth S The role of Dpp and its inhibitors during eggshell patterning in *Drosophila* Development 2007 134 2261 2271 10.1242/dev.02856 17507396

[B77] Casanueva MO Ferguson EL Germline stem cell number in the *Drosophila* ovary is regulated by redundant mechanisms that control Dpp signaling Development 2004 131 1881 1890 10.1242/dev.01076 15105369

[B78] Culi J Mann RS Boca, an endoplasmic reticulum protein required for wingless signaling and trafficking of LDL receptor family members in *Drosophila* Cell 2003 112 343 354 10.1016/S0092-8674(02)01279-5 12581524

[B79] Fu J Posnien N Bolognesi R Fischer TD Rayl P Oberhofer G Kitzmann P Brown SJ Bucher G Asymmetrically expressed axin required for anterior development in Tribolium Proc Natl Acad Sci 2012 109 7782 7786 10.1073/pnas.1116641109 22552230 PMC3356623

[B80] Cohen ED Mariol MC Wallace RMH Weyers J Kamberov YG Pradel J Wilder EL *DWnt4* regulates cell movement and focal adhesion kinase during *Drosophila* ovarian morphogenesis Dev Cell 2002 2 437 448 10.1016/S1534-5807(02)00142-9 11970894

[B81] Gorfinkiel N Sierra J Callejo A Ibanez C Guerrero I The *Drosophila* ortholog of the human Wnt inhibitor factor *Shifted* controls the diffusion of lipid-modified Hedgehog Dev Cell 2005 8 241 253 10.1016/j.devcel.2004.12.018 15691765

[B82] Goodrich JS Clouse KN Schüpbach T Hrb27C, Sqd and Otu cooperatively regulate *gurken* RNA localization and mediate nurse cell chromosome dispersion in *Drosophila* oogenesis Development 2004 131 1949 1958 10.1242/dev.01078 15056611

[B83] Gonzalez-Reyes A St Johnston D The *Drosophila* AP axis is polarised by the cadherin-mediated positioning of the oocyte Development 1998 125 3635 3644 9716529 10.1242/dev.125.18.3635

[B84] de Cuevas M Spradling AC Morphogenesis of the *Drosophila* fusome and its implications for oocyte specification Development 1998 125 2781 2789 9655801 10.1242/dev.125.15.2781

[B85] Airoldi SJ McLean PF Shimada Y Cooley L Intercellular protein movement in syncytial *Drosophila* follicle cells J Cell Sci 2011 124 4077 4086 10.1242/jcs.090456 22135360 PMC3244987

[B86] Lin H Spradling AC Fusome asymmetry and oocyte determination in *Drosophila* Dev Genet 1995 16 6 12 10.1002/dvg.1020160104 7758245

[B87] Cox DN Lu B Sun T-Q Williams LT Jan YN *Drosophila par-1* is required for oocyte differentiation and microtubule organization Curr Biol 2001 11 75 87 10.1016/S0960-9822(01)00027-6 11231123

[B88] Gonzalez-Reyes A Elliott H St Johnston D Polarization of both major body axes in *Drosophila* by gurken-torpedo signalling Nature 1995 375 654 658 10.1038/375654a0 7791898

[B89] Yakoby N Bristow CA Gong D Schafer X Lembong J Zartman JJ Halfon MS Schüpbach T Shvartsman SY A combinatorial code for pattern formation in *Drosophila* oogenesis Dev Cell 2008 15 725 737 10.1016/j.devcel.2008.09.008 19000837 PMC2822874

[B90] McDonald JA Pinheiro EM Kadlec L Schüpbach T Montell DJ Multiple EGFR ligands participate in guiding migrating border cells Dev Biol 2006 296 94 103 10.1016/j.ydbio.2006.04.438 16712835

[B91] Technau M Knispel M Roth S Molecular mechanisms of EGF signaling-dependent regulation of pipe, a gene crucial for dorsoventral axis formation in *Drosophila* Dev Genes Evol 2012 222 1 17 10.1007/s00427-011-0384-2 22198544 PMC3291829

[B92] Zhang Z Zhu X Stevens LM Stein D Distinct functional specificities are associated with protein isoforms encoded by the *Drosophila* dorsal-ventral patterning gene *pipe* Development 2009 136 2779 2789 10.1242/dev.034413 19633171 PMC2730406

[B93] Carneiro K Fontenele M Negreiros E Lopes E Bier E Araujo H Graded maternal short gastrulation protein contributes to embryonic dorsal–ventral patterning by delayed induction Dev Biol 2006 296 203 218 10.1016/j.ydbio.2006.04.453 16781701

[B94] Myohara M Fate mapping of the silkworm, *Bombyx mori*, using localized UV irradiation of the egg at fertilization Development 1994 120 2869 2877 7607077 10.1242/dev.120.10.2869

[B95] Schober M Rebay I Perrimon N Function of the ETS transcription factor Yan in border cell migration Development 2005 132 3493 3504 10.1242/dev.01911 16014514

[B96] Larkin MK Deng WM Holder K Tworoger M Clegg N Ruohola-Baker H Role of Notch pathway in terminal follicle cell differentiation during *Drosophila* oogenesis Dev Genes Evol 1999 209 301 311 10.1007/s004270050256 11252183

[B97] Zhao D Woolner S Bownes M The Mirror transcription factor links signalling pathways in *Drosophila* oogenesis Dev Genes Evol 2000 210 449 457 10.1007/s004270000081 11180850

[B98] Schoppmeier M Fischer S Schmitt-Engel C Loehr U Klingler M An ancient anterior patterning system promotes *caudal* repression and head formation in Ecdysozoa Curr Biol 2009 19 1811 1815 10.1016/j.cub.2009.09.026 19818622

[B99] Singh N Morlock H Hanes SD The Bin3 RNA methyltransferase is required for repression of *caudal* translation in the *Drosophila* embryo Dev Biol 2011 352 104 115 10.1016/j.ydbio.2011.01.017 21262214

[B100] Murata Y Wharton RP Binding of pumilio to maternal *hunchback* mRNA is required for posterior patterning in *Drosophila* embryos Cell 1995 80 747 756 10.1016/0092-8674(95)90353-4 7889568

[B101] Patel NH Hayward DC Lall S Pirkl NR DiPietro D Ball EE Grasshopper *hunchback* expression reveals conserved and novel aspects of axis formation and segmentation Development 2001 128 3459 3472 11566852 10.1242/dev.128.18.3459

[B102] Kobayashi S Yamada M Asaoka M Kitamura T Essential role of the posterior morphogen nanos for germline development in *Drosophila* Nature 1996 380 708 711 10.1038/380708a0 8614464

[B103] Anne J Mechler BM Valois, a component of the nuage and pole plasm, is involved in assembly of these structures, and binds to Tudor and the methyltransferase Capsuléen Development 2005 132 2167 2177 10.1242/dev.01809 15800004

[B104] Andrews S Snowflack DR Clark IE Gavis ER Multiple mechanisms collaborate to repress nanos translation in the *Drosophila* ovary and embryo RNA 2011 17 967 977 10.1261/rna.2478611 21460235 PMC3078745

[B105] Zaessinger S Busseau I Simonelig M Oskar allows *nanos* mRNA translation in *Drosophila* embryos by preventing its deadenylation by Smaug/CCR4 Development 2006 133 4573 4583 10.1242/dev.02649 17050620

[B106] Kim-Ha J Kerr K Macdonald PM Translational regulation of *oskar* mRNA by Bruno, an ovarian RNA-binding protein, is essential Cell 1995 81 403 412 10.1016/0092-8674(95)90393-3 7736592

[B107] Cook HA Koppetsch BS Wu J Theurkauf WE The *Drosophila* SDE3 homolog *armitage* is required for *oskar* mRNA silencing and embryonic axis specification Cell 2004 116 817 829 10.1016/S0092-8674(04)00250-8 15035984

[B108] Anne J Targeting and anchoring Tudor in the pole plasm of the *Drosophila* oocyte PLoS One 2010 5 e14362 10.1371/journal.pone.0014362 21179512 PMC3002268

[B109] Patil VS Kai T Repression of retroelements in *Drosophila* germline via piRNA pathway by the tudor domain protein tejas Curr Biol 2010 20 724 730 10.1016/j.cub.2010.02.046 20362446

[B110] Handler D Olivieri D Novatchkova M Gruber FS Meixner K Mechtler K Stark A Sachidanandam R Brennecke J A systematic analysis of *Drosophila* TUDOR domain-containing proteins identifies Vreteno and the Tdrd12 family as essential primary piRNA pathway factors EMBO J 2011 30 3977 3993 10.1038/emboj.2011.308 21863019 PMC3209783

[B111] Callebaut I Mornon J-P LOTUS, a new domain associated with small RNA pathways in the germline Bioinformatics 2010 26 1140 1144 10.1093/bioinformatics/btq122 20305267

[B112] Malone CD Brennecke J Dus M Stark A McCombie WR Sachidanandam R Hannon GJ Specialized piRNA pathways act in germline and somatic tissues of the *Drosophila* ovary Cell 2009 137 522 535 10.1016/j.cell.2009.03.040 19395010 PMC2882632

[B113] Cox DN Chao A Baker J Chang L Qiao D Lin H A novel class of evolutionarily conserved genes defined by *piwi* are essential for stem cell self-renewal Genes Dev 1998 12 3715 3727 10.1101/gad.12.23.3715 9851978 PMC317255

[B114] Sato K Nishida KM Shibuya A Siomi MC Siomi H Maelstrom coordinates microtubule organization during *Drosophila* oogenesis through interaction with components of the MTOC Genes Dev 2011 25 2361 2373 10.1101/gad.174110.111 22085963 PMC3222902

[B115] Pane A Wehr K Schüpbach T *Zucchini* and *squash* encode two putative nucleases required for rasiRNA production in the *Drosophila* germline Dev Cell 2007 12 851 862 10.1016/j.devcel.2007.03.022 17543859 PMC1945814

[B116] Lin MD Jiao X Grima D Newbury SF Kiledjian M Chou TB *Drosophila* processing bodies in oogenesis Dev Biol 2008 322 276 288 10.1016/j.ydbio.2008.07.033 18708044

[B117] Fan S-J Marchand V Ephrussi A *Drosophila* Ge-1 promotes P Body formation and *oskar* mRNA localization PLoS One 2011 6 e20612 10.1371/journal.pone.0020612 21655181 PMC3105097

[B118] Jongens TA Hay B Jan LY Jan YN The *germ cell-less* gene product: a posteriorly localized component necessary for germ cell development in *Drosophila* Cell 1992 70 569 584 10.1016/0092-8674(92)90427-E 1380406

[B119] Lecuyer E Yoshida H Parthasarathy N Alm C Babak T Cerovina T Hughes TR Tomancak P Krause HM Global analysis of mRNA localization reveals a prominent role in organizing cellular architecture and function Cell 2007 131 174 187 10.1016/j.cell.2007.08.003 17923096

[B120] Reeves GT Stathopoulos A Graded Dorsal and differential gene regulation in the *Drosophila* embryo Cold Spring Harb Perspect Biol 2009 1 4 a000836 10.1101/cshperspect.a000836 20066095 PMC2773625

[B121] Chen LY Wang JC Hyvert Y Lin HP Perrimon N Imler JL Hsu JC Weckle is a zinc finger adaptor of the Toll pathway in dorsoventral patterning of the *Drosophila* embryo Curr Biol 2006 16 1183 1193 10.1016/j.cub.2006.05.050 16782008

[B122] Kleve CD Siler DA Syed SK Eldon ED Expression of *18-wheeler* in the follicle cell epithelium affects cell migration and egg morphology in *Drosophila* Dev Dyn 2006 235 1953 1961 10.1002/dvdy.20820 16607637

[B123] Imamura M Yamakawa M Molecular cloning and expression of a *Toll* receptor gene homologue from the silkworm, *Bombyx mori* Biochim Biophys Acta 2002 1576 246 254 10.1016/S0167-4781(02)00336-6 12084571

[B124] Huang JD Dubnicoff T Liaw GJ Bai Y Valentine SA Shirokawa JM Lengyel JA Courey AJ Binding sites for transcription factor NTF-1/Elf-1 contribute to the ventral repression of *decapentaplegic* Genes Dev 1995 9 3177 3189 10.1101/gad.9.24.3177 8543160

[B125] Araujo H Bier E Sog and dpp exert opposing maternal functions to modify Toll signaling and pattern the dorsoventral axis of the *Drosophila* embryo Development 2000 127 3631 10903186 10.1242/dev.127.16.3631

[B126] George H Terracol R The *vrille* gene of *Drosophila* is a maternal enhancer of *decapentaplegic* and encodes a new member of the bZIP family of transcription factors Genetics 1997 146 1345 1363 9258679 10.1093/genetics/146.4.1345 PMC1208080

[B127] Bartoszewski S Luschnig S Desjeux I Grosshans J Nüsslein-Volhard C *Drosophila* p24 homologues *eclair* and *baiser* are necessary for the activity of the maternally expressed Tkv receptor during early embryogenesis Mech Dev 2004 121 1259 1273 10.1016/j.mod.2004.05.006 15327786

[B128] Ait-Ahmed O Thomas-Cavallin M Joblet C Capri M Expression in the central nervous system of a subset of the *yema* maternally acting genes during *Drosophila* embryogenesis. Post-embryonic expression extends to imaginal discs and spermatocytes Cell Diff Dev 1990 31 53 65 10.1016/0922-3371(90)90090-J 1699639

[B129] Zarnescu DC Jin P Betschinger J Nakamoto M Wang Y Dockendorff TC Feng Y Jongens TA Sisson JC Knoblich JA Fragile X protein functions with lgl and the par complex in flies and mice Dev Cell 2005 8 43 52 10.1016/j.devcel.2004.10.020 15621528

[B130] Ventura G Furriols M Martín N Barbosa V Casanova J *Closca,* a new gene required for both Torso RTK activation and vitelline membrane integrity. Germline proteins contribute to *Drosophila* eggshell composition Dev Biol 2010 344 224 232 10.1016/j.ydbio.2010.05.002 20457146

[B131] Klingler M Erdelyi M Szabad J Nüsslein-Volhard C Function of *torso* in determining the terminal anlagen of the *Drosophila* embryo Nature 1988 335 275 277 10.1038/335275a0 3412488

[B132] Savant-Bhonsale S Montell DJ *Torso-like* encodes the localized determinant of *Drosophila* terminal pattern formation Genes Dev 1993 7 2548 2555 10.1101/gad.7.12b.2548 8276237

[B133] Schoppmeier M Schroder R Maternal torso signaling controls body axis elongation in a short germ insect Curr Biol 2005 15 2131 2136 10.1016/j.cub.2005.10.036 16332539

[B134] Dearden PK Wilson MJ Sablan L Osborne PW Havler M McNaughton E Kimura K Milshina NV Hasselmann M Gempe T Patterns of conservation and change in honey bee developmental genes Genome Res 2006 16 1376 1384 10.1101/gr.5108606 17065607 PMC1626639

[B135] Wilson MJ Dearden PK Tailless patterning functions are conserved in the honeybee even in the absence of Torso signaling Dev Biol 2009 335 276 287 10.1016/j.ydbio.2009.09.002 19735651

[B136] Bornemann D Miller E Simon J The *Drosophila Polycomb* group gene *Sex comb on midleg (Scm)* encodes a zinc finger protein with similarity to polyhomeotic protein Development 1996 122 1621 1630 8625848 10.1242/dev.122.5.1621

[B137] Narbonne K Besse F Brissard-Zahraoui J Pret AM Busson D *Polyhomeotic* is required for somatic cell proliferation and differentiation during ovarian follicle formation in *Drosophila* Development 2004 131 1389 1400 10.1242/dev.01003 14993188

[B138] Li Z Tatsuke T Sakashita K Zhu L Xu J Mon H Lee JM Kusakabe T Identification and characterization of Polycomb group genes in the silkworm, *Bombyx mori* Mol Biol Rep 2012 39 5575 5588 10.1007/s11033-011-1362-5 22187347

[B139] Kiefer JC Epigenetics in development Dev Dyn 2007 236 1144 1156 10.1002/dvdy.21094 17304537

[B140] Sugimura I Lilly MA Bruno inhibits the expression of mitotic cyclins during the prophase I meiotic arrest of *Drosophila* oocytes Dev Cell 2006 10 127 135 10.1016/j.devcel.2005.10.018 16399084

[B141] Suomalainen E Cook LM Turner JRG Achiasmatic oogenesis in the Heliconiine butterflies Hereditas 1973 74 302 304

[B142] Rasmussen SW Raveh D Cowen J Lewis KR Meiosis in *Bombyx mori* females Philos Trans R Soc Lond B Biol Sci 1977 277 343 350 10.1098/rstb.1977.0022 16295

[B143] Rasmussen SW The transformation of the Synaptonemal Complex into the ‘elimination chromatin’ in *Bombyx mori* oocytes Chromosoma 1977 60 205 221 10.1007/BF00329771 870294

[B144] von Wettstein D The synaptonemal complex and genetic segregation Symp Soc Exp Biol 1984 38 195 231 6545723

[B145] Gause M Webber HA Misulovin Z Haller G Rollins RA Eissenberg JC Bickel SE Dorsett D Functional links between *Drosophila* Nipped-B and cohesin in somatic and meiotic cells Chromosoma 2008 117 51 66 10.1007/s00412-007-0125-5 17909832 PMC2258212

[B146] Carney GE Bender M The *Drosophila ecdysone receptor* (*EcR*) gene is required maternally for normal oogenesis Genetics 2000 154 1203 1211 10757764 10.1093/genetics/154.3.1203 PMC1461007

[B147] Sommer B Oprins A Rabouille C Munro S The exocyst component Sec5 is present on endocytic vesicles in the oocyte of *Drosophila melanogaster* J Cell Biol 2005 169 953 963 10.1083/jcb.200411053 15955846 PMC2171629

[B148] Schonbaum CP Perrino JJ Mahowald AP Regulation of the vitellogenin receptor during *Drosophila melanogaster* oogenesis Mol Biol Cell 2000 11 511 521 10679010 10.1091/mbc.11.2.511 PMC14789

[B149] Pistillo D Manzi A Tino A Boyl PP Graziani F Malva C The *Drosophila melanogaster* lipase homologs: a gene family with tissue and developmental specific expression J Mol Biol 1998 276 877 885 10.1006/jmbi.1997.1536 9566193

[B150] Yamada R Yamahama Y Sonobe H Release of ecdysteroid-phosphates from egg yolk granules and their dephosphorylation during early embryonic development in silkworm, *Bombyx mori* Zool Sci 2005 22 187 198 10.2108/zsj.22.187 15738639

[B151] Eystathioy T Swevers L Iatrou K The orphan nuclear receptor BmHR3A of *Bombyx mori*: hormonal control, ovarian expression and functional properties Mech Dev 2001 103 107 115 10.1016/S0925-4773(01)00335-5 11335116

[B152] Liu Y-Q Chen M-M Li Q Li Y-P Xu L Wang H Zhou Q-K Sima Y-H Wei Z-J Jiang D-F Characterization of a gene encoding KK-42-binding protein in *Antheraea pernyi* (Lepidoptera: Saturniidae) Ann Entomol Soc Am 2012 105 718 725 10.1603/AN12009

[B153] Perera OP Shirk PD cDNA of YP4, a follicular epithelium yolk protein subunit, in the moth, *Plodia interpunctella* Arch Insect Biochem Physiol 1999 40 157 164 10.1002/(SICI)1520-6327(1999)40:3<157::AID-ARCH5>3.0.CO;2-W 10207993

[B154] Terashima J Bownes M Translating available food into the number of eggs laid by *Drosophila melanogaster* Genetics 2004 167 1711 1719 10.1534/genetics.103.024323 15342510 PMC1470999

[B155] Richard DS Rybczynski R Wilson TG Wang Y Wayne ML Zhou Y Partridge L Harshman LG Insulin signaling is necessary for vitellogenesis in *Drosophila melanogaster* independent of the roles of juvenile hormone and ecdysteroids: female sterility of the *chico1* insulin signaling mutation is autonomous to the ovary J Insect Physiol 2005 51 455 464 10.1016/j.jinsphys.2004.12.013 15890189

[B156] Iwami M Tanaka A Hano N Sakurai S Bombyxin gene expression in tissues other than brain detected by reverse transcription-polymerase chain reaction (RT-PCR) and in situ hybridization Experientia 1996 52 882 887 10.1007/BF01938875 8841516

[B157] Swevers L Drevet JR Lunke MD Iatrou K The silkmoth homolog of the *Drosophila* ecdysone receptor (BI Isoform): Cloning and analysis of expression during follicular cell differentiation Insect Biochem Mol Biol 1995 25 857 866 10.1016/0965-1748(95)00024-P 7633470

[B158] Charles J-P Iwema T Epa VC Takaki K Rynes J Jindra M Ligand-binding properties of a juvenile hormone receptor, Methoprene-tolerant Proc Natl Acad Sci 2011 108 21128 21133 10.1073/pnas.1116123109 22167806 PMC3248530

[B159] Abdou MA He Q Wen D Zyaan O Wang J Xu J Baumann AA Joseph J Wilson TG Li S Wang J *Drosophila* Met and Gce are partially redundant in transducing juvenile hormone action Insect Biochem Mol Biol 2011 41 938 945 10.1016/j.ibmb.2011.09.003 21968404

[B160] Li M Mead EA Zhu J Heterodimer of two bHLH-PAS proteins mediates juvenile hormone-induced gene expression Proc Natl Acad Sci 2011 108 638 643 10.1073/pnas.1013914108 21187375 PMC3021087

[B161] Willis DK Wang J Lindholm JR Orth A Goodman WG Microarray analysis of juvenile hormone response in *Drosophila melanogaster* S2 cells J Insect Sci 2010 10 66 20672983 10.1673/031.010.6601 PMC3014815

[B162] Birnbaum MJ Gilbert LI Juvenile hormone stimulation of ornithine decarboxylase activity during vitellogenesis in *Drosophila melanogaster* J Comp Physiol B 1990 160 145 151 10.1007/BF00300946 2118148

[B163] Seino A Ogura T Tsubota T Shimomura M Nakakura T Tan A Mita K Shinoda T Nakagawa Y Shiotsuki T Characterization of juvenile hormone epoxide hydrolase and related genes in the larval development of the silkworm *Bombyx mori* Biosci Biotechnol Biochem 2010 74 1421 1429 10.1271/bbb.100104 20622465

[B164] Buszczak M Freeman MR Carlson JR Bender M Cooley L Segraves WA Ecdysone response genes govern egg chamber development during mid-oogenesis in *Drosophila* Development 1999 126 4581 4589 10498692 10.1242/dev.126.20.4581

[B165] Roth GE Gierl MS Vollborn L Meise M Lintermann R Korge G The *Drosophila* gene *Start1*: a putative cholesterol transporter and key regulator of ecdysteroid synthesis Proc Natl Acad Sci 2004 101 1601 1606 10.1073/pnas.0308212100 14745013 PMC341787

[B166] Freeman MR Dobritsa A Gaines P Segraves WA Carlson JR The *dare* gene: steroid hormone production, olfactory behavior, and neural degeneration in *Drosophila* Development 1999 126 4591 4602 10498693 10.1242/dev.126.20.4591

[B167] Gaziova I Bonnette PC Henrich VC Jindra M Cell-autonomous roles of the *ecdysoneless* gene in *Drosophila* development and oogenesis Development 2004 131 2715 2725 10.1242/dev.01143 15128659

[B168] Sakudoh T Tsuchida K Kataoka H BmStart1, a novel carotenoid-binding protein isoform from *Bombyx mori*, is orthologous to MLN64, a mammalian cholesterol transporter Biochem Biophys Res Commun 2005 336 1125 1135 10.1016/j.bbrc.2005.08.241 16169523

[B169] Swevers L Iatrou K The orphan receptor BmHNF-4 of the silkmoth *Bombyx mori*: ovarian and zygotic expression of two mRNA isoforms encoding polypeptides with different activating domains Mech Dev 1998 72 3 13 10.1016/S0925-4773(97)00180-9 9533948

[B170] Tootle TL Williams D Hubb A Frederick R Spradling A *Drosophila* eggshell production: identification of new genes and coordination by Pxt PLoS One 2011 6 e19943 10.1371/journal.pone.0019943 21637834 PMC3102670

[B171] Hong CC Hashimoto C An unusual mosaic protein with a protease domain, encoded by the *nudeI* gene, is involved in defining embryonic dorsoventral polarity in *Drosophila* Cell 1995 82 785 794 10.1016/0092-8674(95)90475-1 7671306

[B172] Kendirgi F Swevers L Iatrou K An ovarian follicular epithelium protein of the silkworm (*Bombyx mori*) that associates with the vitelline membrane and contributes to the structural integrity of the follicle FEBS Lett 2002 524 59 68 10.1016/S0014-5793(02)03003-X 12135742

[B173] Calvi BR Lilly MA Spradling AC Cell cycle control of chorion gene amplification Genes Dev 1998 12 734 744 10.1101/gad.12.5.734 9499407 PMC316579

[B174] Jones CW Kafatos FC Linkage and evolutionary diversification of developmentally regulated multigene families: tandem arrays of the 401/18 chorion gene pair in silkmoths Mol Cell Biol 1981 1 814 828 9279394 10.1128/mcb.1.9.814-828.1981 PMC369365

[B175] Sourmeli S Papantonis A Lecanidou R A novel role for the *Bombyx* Slbo homologue, BmC/EBP, in insect choriogenesis Biochem Biophys Res Commun 2005 337 713 719 10.1016/j.bbrc.2005.09.103 16202393

[B176] Papantonis A Van den Broeck J Lecanidou R Architectural factor HMGA induces promoter bending and recruits C/EBP and GATA during silkmoth chorion gene regulation Biochem J 2008 416 85 97 10.1042/BJ20081012 18636971

[B177] Shea MJ King DL Conboy MJ Mariani BD Kafatos FC Proteins that bind to *Drosophila* chorion *cis*-regulatory elements: a new C[[2]]H[[2]] zinc finger protein and a C[[2]]C[[2]] steroid receptor-like component Genes Dev 1990 4 1128 10.1101/gad.4.7.1128 2120114

[B178] Jagadeeshan S Singh RS Rapid evolution of outer egg membrane proteins in the *Drosophila melanogaster* subgroup: a case of ecologically driven evolution of female reproductive traits Mol Biol Evol 2007 24 929 938 10.1093/molbev/msm009 17244601

[B179] Leclerc RF Regier JC Evolution of chorion gene families in lepidoptera: characterization of 15 cDNAs from the gypsy moth J Mol Evol 1994 39 244 254 10.1007/BF00160148 7932786

[B180] Xu Y Fu Q Li S He N Silkworm egg proteins at the germ-band formation stage and a functional analysis of BmEP80 protein Insect Biochem Mol Biol 2011 41 572 581 10.1016/j.ibmb.2011.03.009 21457781

[B181] Mpakou VE Nezis IP Stravopodis DJ Margaritis LH Papassideri IS Different modes of programmed cell death during oogenesis of the silkmoth Bombyx mori Autophagy 2008 4 97 100 17986869 10.4161/auto.5205

[B182] Hou YC Chittaranjan S Barbosa SG McCall K Gorski SM Effector caspase Dcp-1 and IAP protein Bruce regulate starvation-induced autophagy during *Drosophila melanogaster* oogenesis J Cell Biol 2008 182 1127 1139 10.1083/jcb.200712091 18794330 PMC2542474

[B183] Zhang J-Y Pan M-H Sun Z-Y Huang S-J Yu Z-S Liu D Zhao D-H Lu C The genomic underpinnings of apoptosis in the silkworm, *Bombyx mori* BMC Genom 2010 11 611 10.1186/1471-2164-11-611 PMC3091752 21040523

[B184] Yu J Zheng Y Dong J Klusza S Deng W-M Pan D Kibra functions as a tumor suppressor protein that regulates Hippo signaling in conjunction with Merlin and Expanded Dev Cell 2010 18 288 10.1016/j.devcel.2009.12.012 20159598 PMC2858562

[B185] Jankovics F Sinka R Lukacsovich T Erdelyi M Moesin crosslinks actin and cell membrane in *Drosophila* oocytes and is required for Oskar anchoring Curr Biol 2002 12 2060 2065 10.1016/S0960-9822(02)01256-3 12477397

[B186] Sarkar S Lakhotia SC Hsp60C is required in follicle as well as germline cells during oogenesis in *Drosophila melanogaster* Dev Dyn 2008 237 1334 1347 10.1002/dvdy.21524 18386820

[B187] Cobreros L Fernández-Miñán A Luque CM González-Reyes A Martín-Bermudo MD A role for the chaperone Hsp70 in the regulation of border cell migration in the *Drosophila* ovary Mech Dev 2008 125 1048 1058 10.1016/j.mod.2008.07.006 18718532

[B188] Qian S Hongo S Jacobs-Lorena M Antisense ribosomal protein gene expression specifically disrupts oogenesis in *Drosophila melanogaster* Proc Natl Acad Sci 1988 85 9601 9605 10.1073/pnas.85.24.9601 3144001 PMC282811

[B189] Starr DJ Cline TW A host parasite interaction rescues *Drosophila* oogenesis defects Nature 2002 418 76 79 10.1038/nature00843 12097909

[B190] Kremer N Voronin D Charif D Mavingui P Mollereau B Vavre F *Wolbachia* interferes with ferritin expression and iron metabolism in insects PLoS Path 2009 5 e1000630 10.1371/journal.ppat.1000630 PMC2759286 19851452

[B191] Stouthamer R Breeuwer JAJ Hurst GDD *Wolbachia pipientis*: Microbial manipulator of arthropod reproduction Annu Rev Microbiol 1999 53 71 102 10.1146/annurev.micro.53.1.71 10547686

[B192] Dedeine F Vavre F Fleury F Loppin B Hochberg ME Boulétreau M Removing symbiotic *Wolbachia* bacteria specifically inhibits oogenesis in a parasitic wasp Proc Natl Acad Sci 2001 98 6247 6252 10.1073/pnas.101304298 11353833 PMC33453

[B193] Serbus LR Ferreccio A Zhukova M McMorris CL Kiseleva E Sullivan W A feedback loop between Wolbachia and the *Drosophila gurken* mRNP complex influences Wolbachia titer J Cell Sci 2011 124 4299 4308 10.1242/jcs.092510 22193955 PMC3258112

[B194] Horner VL Wolfner MF Transitioning from egg to embryo: Triggers and mechanisms of egg activation Dev Dyn 2008 237 527 544 10.1002/dvdy.21454 18265018

[B195] Cui J Sackton KL Horner VL Kumar KE Wolfner MF Wispy, the *Drosophila* homolog of GLD-2, is required during oogenesis and egg activation Genetics 2008 178 2017 2029 10.1534/genetics.107.084558 18430932 PMC2323793

[B196] Chomczynski P Sacchi N Single-step method of RNA isolation by acid guanidinium thiocyanate-phenol-chloroform extraction Anal Biochem 1987 162 156 159 2440339 10.1006/abio.1987.9999

[B197] Li J Li X Chen Y Yang Z Guo S Solexa sequencing based transcriptome analysis of *Helicoverpa armigera* larvae Mol Biol Rep 2012 39 11051 11059 10.1007/s11033-012-2008-y 23065207

[B198] Goecks J Nekrutenko A Taylor J Team TG Galaxy: a comprehensive approach for supporting accessible, reproducible, and transparent computational research in the life sciences Genome Biol 2010 11 R86 10.1186/gb-2010-11-8-r86 20738864 PMC2945788

[B199] Blankenberg D Gordon A Von Kuster G Coraor N Taylor J Nekrutenko A Team tG: **Manipulation of FASTQ data with Galaxy** Bioinformatics 2010 26 1783 1785 10.1093/bioinformatics/btq281 20562416 PMC2894519

[B200] Trapnell C Pachter L Salzberg SL TopHat: discovering splice junctions with RNA-Seq Bioinformatics 2009 25 1105 1111 10.1093/bioinformatics/btp120 19289445 PMC2672628

[B201] Roberts A Trapnell C Donaghey J Rinn J Pachter L Improving RNA-Seq expression estimates by correcting for fragment bias Genome Biol 2011 12 R22 10.1186/gb-2011-12-3-r22 21410973 PMC3129672

[B202] Conesa A Götz S García-Gómez JM Terol J Talón M Robles M Blast2GO: a universal tool for annotation, visualization and analysis in functional genomics research Bioinformatics 2005 21 3674 3676 10.1093/bioinformatics/bti610 16081474

[B203] Pfaffl MW Horgan GW Dempfle L Relative expression software tool (REST©) for group-wise comparison and statistical analysis of relative expression results in real-time PCR Nucleic Acids Res 2002 30 e36 10.1093/nar/30.9.e36 11972351 PMC113859

[B204] Tarazona S García-Alcalde F Dopazo J Ferrer A Conesa A Differential expression in RNA-seq: A matter of depth Genome Res 2011 21 2213 2223 10.1101/gr.124321.111 21903743 PMC3227109

[B205] Colborn JM Byrd BD Koita OA Krogstad DJ Estimation of copy number using SYBR Green: confounding by AT-rich DNA and by variation in amplicon length Am J Trop Med Hyg 2008 79 887 892 19052298

