# Supplementary material for: Unscrambling butterfly oogenesis
Source: BMC Genomics. 2013 Apr 26;14:283. doi: 10.1186/1471-2164-14-283 (PMC3654919; doi:10.1186/1471-2164-14-283)
Supplement: Additional file 1 — Oogenesis genes. Contains a tabulated and fully referenced list of genes identified from the literature, which have been studied in the context of insect oogenesis and maternal regulation of early embryogenesis. The vast majority of papers concern the fruitfly Drosophila melanogaster and the silkmoth Bombyx mori. Many genes have multiple functions during oogenesis, but to avoid repetition, and keep the size of the Table manageable, each gene has been listed only once in the functional context for which it is probably best known. Referencing has been kept to a minimum, highlighting key papers and databases. Hyperlinks have been provided for almost all of the genes listed, which will provide full database information on their myriad functions and further references. Presence (Y) or absence (N) of orthologs in the Pararge aegeria combined oocyte and ovariole transcriptome are indicated. [file 1471-2164-14-283-S1.pdf]

Additional file 2 – Essential oogenesis genes

Genes identified from the literature that have been studied in the context of insect oogenesis and maternal regulation of early embryogenesis. Furthermore, although for a number of genes functionality during oogenesis can be inferred, their expression during oogenesis has not always been verified experimentally, and wherever this was found to be the case, it has been clearly highlighted in the table. FlyBase [1] and SilkBase [2] were used as a starting point to conduct the comprehensive literature search. The vast majority of papers thus mainly concern the model species; the fruitfly *Drosophila melanogaster* (*Dm*) and the silkmoth *Bombyx mori* (*Bm*). The species discussed in the cited papers are indicated in the table (abbreviations given below). Furthermore, for *D. melanogaster* genes, a high-throughput developmental time series database was consulted for FPKM-based gene expression levels [3] as well as an *in-situ* database for maternal transcript contribution to the oocyte [4]. Many genes have multiple functions during oogenesis, but to avoid repetition, and to keep the size of the data table manageable, each gene has been listed only once in the broad functional context for which it is probably best known. Referencing has been kept to a minimum out of necessity, highlighting key papers or expression databases. Wherever informative to do so, hyperlinks have been provided for the genes listed, and these hyperlinks provide full database information on each gene’s myriad of functions, plus further references. Presence (Y), possible presence (Y?) or absence (N) of orthologs in the *Pararge aegeria* combined oocyte and ovariole transcriptome are indicated.

Species abbreviations: *Aa*: *Aedes aegypti*; *Sp*: *Sarcophaga peregrina*; *At*: *Asobara tabida*; *Hc*: *Hyalophora cecropia*; *Md*: *Musca domestica*; *Fm*: *Fenneropenaeus merguiensis*; *Bg*: *Blattella germanica*; *Ms*: *Manduca sexta*; *Nv*: *Nasonia vitripennis*;

Tc: *Tribolium castaneum*; *Rp*: *Rhodnius prolixus*; *Am*: *Apis mellifera*; *Of*: *Oncopeltus fasciatus*; *Pi*: *Plodia interpunctella*

Genes	Gene abbrev.	Present	Species
**Genes functioning early for the maintenance and division of germ-line and ovarian somatic stem cells**			
armadillo [4-6]	*arm*	Y	*Dm*
axin; axis inhibition protein [5]	*axn*	Y	*Dm*
dishevelled [7]	*dsh*	Y	*Dm*
shaggy; gsk-3 [4, 5]	*sgg; Zw3*	Y	*Dm*
sugarless; UDP glucose6 dehydrogenase [8]	*sgl; UDPGDH*	Y	*Dm*
legless [4, 8, 9]	*lgs; BCL9*	Y	*Dm*
pygopus [8, 9]	*pygo; gam*	Y	*Dm*
wingless [5]	*wg*	Y?	*Dm*
wntless; evenness interrupted [4, 10]	*wls; Evi*	Y	*Dm*
hedgehog [11-14]	*hh*	Y	*Dm*
shifted; wnt inhibitory factor 1 precursor [15]	*shf; wif1*	Y	*Dm*
costa [11-14]	*cos2*	N	*Dm*
skinny hedgehog; hedgehog acyltransferase; CG32281 [16]	*ski*	Y	*Dm*
roadkill; similar to *speckle-type POZ protein* [4, 17]	*rdx*	Y	*Dm*
patched [11, 14]	*ptc*	N	*Dm*
smoothened [14]	*smo*	Y	*Dm*
cubitus interruptus [11, 12, 14]	*ci*	Y	*Dm*
engrailed [11]	*en*	N	*Dm*
pangolin [8, 18]	*pan; Tcf/LEF*	Y	*Tc; Dm*
wnt oncogene analog 4 [19]	*wnt4*	N	*Dm*
dicer-1 [20, 21]	*dcr-1*	Y	*Dm; Bg*
loquacious [20]	*loqs*	Y	*Dm*
mir-184 [22]	*mir-184*	N	*Dm*
effete [23-25]	*eff; UbcD1*	Y	*Dm*
fs(1)Yb [6, 26, 27]	*Yb*	N	*Dm*
fused; similar to *serine/threonine kinase 36* [28]	*fu*	Y	*Dm*
Suppressor of fused [4, 28]	*Su(fu)*	Y	*Dm*
bicaudal [29]	*bic*	Y	*Dm*
otefin [30, 31]	*ote*	N	*Dm*
piwi [32, 33]	*piwi*	Y	*Dm*
pelota [34]	*pelo*	Y	*Dm*
pumillio [4, 6, 35-38]	*pum*	Y	*Dm*
penguin [4, 39]	*pen*	Y	*Dm*
sans fille; U1 small nuclear ribonucleoprotein A; fs(1)1621 [40]	*snf*	Y	*Dm*
bric a brac [26, 41]	*bab*	N	*Dm*
shutdown [42, 43]	*shu*	Y	*Dm*
FK506-binding protein [4, 42]	*FKBP59*	Y	*Dm*
vasa; vasa-like gene (vasa homolog in Lepidoptera*)* [44-47]	*vas; vlg*	Y	*Dm; Bm*
outstretched [48]	*upd; sisc*	N	*Dm*
bag of marbles [6, 49-52]	*bam*	N	*Dm*
mei-p26 [53-55]	*mei-p26*	N	*Dm*
brain tumor [4, 53, 55]	*brat*	Y	*Dm*
benign gonial cell neoplasm [50, 51, 56]	*bgcn*	N	*Dm*
within bgcn [56]	*wibg; pym*	Y	*Dm*

decapentaplegic [57-60]	*dpp*	Y	*Dm*
kekkon5 [61]	*kek5*	N	*Dm*
Mothers against dpp [4, 57, 62]	*Mad*	Y	*Dm*
Smad on X [63]	*Smad2; Smox*	Y	*Dm*
saxophone (type I Dpp receptor) [57]	*sax*	N	*Dm*
thick veins (type I Dpp receptor) [57, 60, 64]	*tkv*	Y	*Dm*
punt (type II Dpp receptor) [57]	*pnt*	N	*Dm*
medea [4, 57]	*med; SMAD4*	N	*Dm*
Daughters against dpp [57, 62]	*Dad*	N	*Dm*
glass bottom boat [65]	*gbb*	Y	*Dm*
dullard [4, 66]	*dd*	Y	*Dm*
quo vadis; schnurri [67]	*quo; shn*	N	*Dm*
lethal with a checkpoint kinase [58]	*smurf; lack*	Y	*Dm*
supernumerary limbs [68]	*slimb*	Y	*Dm*
starry night; flamingo [19]	*stan; fmi*	N	*Dm*
roughened; similar to *ras-related protein rap-1a; enhancer of faf;* similar to *Bombyx mori ras3* [69]	*r; rap1; dras3*	Y	*Dm*
ras-associated protein 2-like; ras-related protein 2	*rap2l*	Y	*Dm*
fruitless isoform a [70-72]	*fru*	Y	*Dm*
fruitless isoform k [70-72]	*fru*	Y	*Dm*
fruitless [70-72]	*fru*	Y	*Dm*
sex-lethal [73, 74]	*sxl*	N	*Dm*
pre-mRNA-splicing regulator wtap; similar to *female lethal d;* CG6315 [75]	*fl(2)d*	N	*Dm*
maleless; ATP-*dependent RNA helicase a-like* [76]	*mle; dhx9; nap*	Y	*Dm*
lamin c [5, 67]	*lamc*	Y	*Dm*
clift; eyes absent [77]	*cli; eya*	Y	*Dm*
slowmo [78]	*slmo*	Y	*Dm*
**Genes affecting the cytoskeleton and actomyosin contractile ring assembly**			
abnormal spindle (a microtubule-associated protein) [79]	*asp*	N	*Dm*
javelin-like (microtubule-associated protein); similar to CG3563 [80, 81]	*jvl*	Y	*Dm*
mini spindles (microtubule-associated protein; belongs to *xmap215/tog family of genes)* [82, 83]	*msps; xmap215*	Y	*Dm*
a-kinase anchor protein 200 [84]	*akap200*	N	*Dm*
capulet; act up, bcDNA:ld24380, CG5061 [85]	*capt*	N	*Dm*
cdc42 [86, 87]	*cdc42*	Y	*Dm*
*Bombyx mori cdc42 small effector 2-like protein (*LOC692865*)* [4]	*cdc42-sep2; spec2*	Y	*Dm*
p21/cdc42/rac1 activated kinase [86, 88]	*pak*	Y	*Dm*
rac1; ras-related c3 botulinum toxin substrate 1 [86]	*rac1*	Y	*Dm*
specifically Rac1 associated protein; Fmr1-interacting protein	*sra-1; cyfip*	Y	*Its role in oogenesis not described*
engulfment and cell motility protein; ced-12 homolog [89]	*ced-12; elmo*	Y	*Dm*
centrosomin [83, 90]	*cnn*	Y	*Dm*
aurora-a [91, 92]	*aur*	Y	*Dm*
chickadee (homolog of *profilin)* [93]	*chic*	Y	*Dm*
citron; sticky [94]	*sti; dck*	N	*Dm*

focal adhesion kinase-like; fak56(D) [95]	*fak56D*	Y	*Dm*
diaphanous [96]	*dia*	Y	*Dm*
frizzled; frizzled-7-like	*fz7-l*	Y	*Its role in oogenesis not described*
frizzled; frizzled-2-like [4]	*fz2-l*	Y	*Dm*
chromosome bows; mast; orbit; clasp [97]	*chb*	N	*Dm*
shotgun; E-Cadherin [4, 98-100]	*shg; E-Cad*	Y	*Dm*
mushroom body defect [101]	*mud*	N	*Dm*
dishevelled associated activator of morphogenesis-1 [4]	*daam-1*	Y	*Dm*
karst (also known as *betaheavy spectrin)* [102, 103]	*kst*	Y	*Dm*
flightless I [104]	*fliI*	Y	*Dm*
klarsicht [105, 106]	*klar; marb*	Y	*Dm*
muscle-specific protein 300 [4, 105, 106]	*msp-300*	Y	*Dm*
lissencephaly-1 [107]	*lis-1*	Y	*Dm*
cortactin(-like) [108]	*cortactin*	Y	*Dm*
src oncogene at 42a [108]	*src42a*	Y	*Dm*
src oncogene 1 [69, 109]	*src64b*	Y	*Dm*
α actinin [110, 111]	*actn*	Y	*Dm*
ovarian tumor; fs(1)m101; fs(1)231 [112, 113]	*otu*	N	*Dm*
Guanyl cyclase at 32e [114, 115]	*Gyc32e*	N	*Dm*
Guanylyl cyclase at 76c; receptor-type Guanylate cyclase [116]	*Gyc76c*	Y	*Dm*
stand still [117-119]	*stil*	N	*Dm*
hold up [120]	*hup*	N	*Dm*
dicephalic [121]	*dic*	N	*Dm*
kelch [122-124]	*kel*	Y	*Dm*
similar to *kelch domain containing 4* [3]	*klhdcp*	Y	*Its role in oogenesis not described*
cullin 3 [124]	*cul3*	Y	*Dm*
dedicator of cytokinesis 6,7; similar to CG11376 [125]	*dock6; dock7*	Y	*Dm*
myoblast city; dedicator of cytokinesis 1 [126-128]	*mbc; dock180*	Y	*Dm*
spaghetti squash; myosin light polypeptide 9; myosin regulatory light chain 9 [128, 129]	*sqh; mrlc*	Y	*Dm*
nonmuscle myosin essential light chain; myosin II essential light chain	*mlc-c*	Y	*Its role in oogenesis not described*
myosin regulatory light chain interacting protein	*mylip*	Y	*Its role in oogenesis not described*
genghis kahn; cdc42 binding protein kinase alpha or beta [86]	*gek; cdc42bpb*	Y	*Dm*
jaguar/myosin VI [130]	*jar; mhc95f; myo6*	Y	*Dm;* Bees
myosin heavy chain (similar to CG17927) [131, 132]	*mhc*	Y	*Dm*
myosin heavy chain 2; zipper [131]	*zip; mhc2*	Y	*Dm*
myosin light chain kinase; bent; titin-like [88, 129, 133]	*bt*	Y	*Dm*
myosin 1 light chain; myosin alkali light chain 1 [129]	*mlc*	Y	*Dm*
myosin 1; myosin 61f [134]	*myo1b*	Y	*Dm*
dilute class unconventional myosin; myosin V; myosin-Va [135-137]	*myoV; myo-Va; didum*	Y	*Dm;* Bees
unconventional myosin class XV	*myo10a*	Y	*Its role in oogenesis not*

			described
myosin heavy chain like [4]	*mhcl*	Y	*Dm*
CG17293*; WD40 protein type* [4]	*wdr82*	Y	*Dm*
washout [138, 139]	*wash; p63; p65*	N	*Dm*
james bond [140]	*bond*	N	*Dm*
kette; hem-protein; similar to *membrane-associated protein hem (dhem-2);* similar to *membrane-associated protein gex-3* [141]	*hem; kte; nap1; dhem2*	Y	*Dm*
short stop; kakapo; similar to *bullous pemphigoid antigen 1 (Homo sapiens); microtubule-actin cross linking factor 1* [142, 143]	*shot*	Y	*Dm*
vacuolar protein sorting 35 [4]	*vps35*	Y	*Dm*
rotund; racGTPase-activating protein; roughened eye [4, 144]	*rn; roe; rnracgap*	Y	*Dm*
twinstar; actin-depolymerizing factor 1 cofilin [145, 146]	*tsr*	Y	*Dm*
slingshot [3]	*mkp; ssh*	Y	*Its role in oogenesis not described*
subito; double or nothing; Bombyx mori kinesin-like protein c [147, 148]	*sub*	Y	*Dm*
IplI-aurora-like kinase; aurora b (kinase) [148, 149]	*aurb*	Y	*Dm*
tumbleweed; racGAP50c; similar to *racGTPase-activating protein* [148]	*tum; racGAP*	Y	*Dm*
arp2; actin-related protein 14d [150]	*arp2; arp14d*	Y	*Dm*
arp3; actin-related protein 66b [150]	*arp3; arp66b*	Y	*Dm*
suppressor of profilin 2 (also known as *arpc1)* [150]	*sop2; arpc1; arc41*	Y	*Dm*
arp2/3 complex subunit p34; arpc2 [150]	*arpc2; arc-p34*	Y	*Dm*
arp2/3 complex 21kD subunit p21; arpc3b [150]	*arpc3; arpc3b*	Y	*Dm*
arp2/3 complex subunit p20; arpc4 [150]	*arpc4; arc-p20*	Y	*Dm*
arp2/3 complex 16kD subunit p16; arpc5 [150]	*arpc5; p16-arc*	Y	*Dm*
kinesin associated protein 3 [151]	*kap3; kap*	Y	*Dm*
kinesin-like protein at 68d; kinesin II; kinesin-2 [152]	*klp5; klp68d*	Y	*Dm*
kinesin-like protein at 64d; kinesin family member 3a [152]	*klp64d; kif3a*	Y	*Dm*
pericentrin-like protein (cp309) [153]	*cp309*	N	*Dm*
rho-type Guanine exchange factor; pak-interacting exchange factor; AGAP007877 [154]	*rtgef; dpix*	Y	*Dm*
*SCAR; actin binding protein; (*in vertebrates*) wiskott-aldrich syndrome protein family member 2; wasp family protein member 2* [139, 155]	*SCAR; wave*	Y	*Dm*
quail; villin [156]	*qua*	Y	*Dm*
**Genes acting early in the egg for oocyte determination (including fusome formation) and formation of the anterior-posterior axis**			
transitional endoplasmic reticulum ATPase; ter94 [157, 158]	*ter94*	Y	*Dm*
capping protein alpha [159]	*cpa*	Y	*Dm*
leonardo [160, 161]	*14-3-3zeta; leo*	Y	*Dm; Bm*
bazooka [162]	*baz; par3*	Y	*Dm*
bicaudal C [163]	*bicC*	Y	*Dm*
bicaudal D [6, 164]	*bicD*	Y	*Dm*
bicaudal D-related [4]	CG32137	Y	*Dm*

glued; dynactin [165, 166]	*gl*	Y	*Dm*
egalitarian; 3'-5' exonuclease domain-like-containing protein [4, 6, 167, 168]	*egl*	Y	*Dm*
stonewall; fs(3)02024 [6, 169, 170]	*stwl*	N	*Dm*
egghead; zeste-white 4; beta-1,4-mannosyltransferase [171]	*egh; zw4; bre3*	Y	*Dm*
4ehp [172]	*4ehp*	N	*Dm*
pipsqueak (BTB/POZ containing gene) [173]	*psq*	N	*Dm*
BTB/POZ domain containing gene [174]	*BTB-POZ*	Y	VERTEBRATES (FISH)
BTB domain containing protein 2 [174]	*BTBd2*	Y	VERTEBRATES (FISH)
spindle c [175, 176]	*spnc*	N	*Dm*
coracle; band 4.1-like protein [103]	*cora*	Y	*Dm*
alpha spectrin [103, 177]	*alpha-spec*	Y	*Dm*
beta spectrin [103, 177]	*beta-spec*	Y	*Dm*
hu-li tai shao [103, 177, 178]	*hts*	Y	*Dm*
ankyrin; similar to *ankyrin 2,3/unc44;* AGAP002272-PA [103, 179]	*ank*	Y	*Dm*
neuroglian [102]	*ceb; nrg*	Y	*Dm*
inscuteable [180]	*insc*	N	*Dm*
sec61 alpha [181]	*sec61 alpha*	Y	*Dm*
sec61 gamma	*sec61 gamma*	Y	*Its role in oogenesis not described*
sec63 [182]	*sec63*	Y	*Dm*
tropomodulin [182]	*tmod*	Y	*Dm*
p38 MAPK [183, 184]	*p38MAPK*	Y	*Dm*
protein kinase a; cAMP-dependent protein kinase 1; dc0, pka [185]	*pka-c1*	Y	*Dm*
cAMP-dependent protein kinase r1 [186]	*pka-r1*	Y	*Dm*
cAMP-dependent protein kinase r2 [187]	*pka-r2*	Y	*Dm*
atypical protein kinase c; CG10261 [188]	*apkc*	N	*Dm*
typical protein kinase c	*pkc*	Y	*Its role in oogenesis not described*
protein kinase c inhibitor; similar to CG2862 [189]	*pkc inhibitor*	Y	*Its role in oogenesis not described*
rab-protein 6; small (monomeric) GTPase [190]	*rab6*	Y	*Dm*
rhino [191]	*rhi*	N	*Dm*
ß1 tubulin 1 [192]	*tub1*	Y	*Dm*
ß1 tubulin 2 [192]	*tub2*	Y	*Dm*
β-tubulin at 60d [192-194]	*tub3; betatub60d*	Y	*Dm*
β-tubulin at 56d [192-194]	*betatub56d*	Y	*Dm*
homologous to *Drosophila γ-tubulin at 37c; gamma tubulin* (in general) [83, 195-197]	*gammatub37c; gamma tub 1*	Y	*Dm*
gamma-tubulin complex component 3; lethal (1) discs degenerate 4 [196]	*tubgcp3; gcp3; dgrip91*	Y	*Dm*
gamma-tubulin complex component 2; gamma-tubulin ring protein 84 (Drosophila) [196]	*tubgcp2; gcp2; dgrip84*	Y	*Dm*
alpha tubulin tua1; similar to *Drosophila alpha-tubulin at 84b* [195]	*atub; tua1*	Y	*Dm*
alpha tubulin tua2; similar to *Drosophila alpha-tubulin at 84b* [195]	*atub; tua2*	Y	*Dm*
deadlock [195]	*del*	N	*Dm*
mo25; calcium-binding protein 39 [4]	*mo25*	Y	*Dm*

14-3-3ε [198]	*14-3-3epsilon*	Y	*Dm*
par-1; map/microtubule affinity-regulating kinase [162, 198-200]	*par-1*	Y	*Dm*
serine/threonine kinase lkb1; partitioning defective 4 [201]	*lkb1; par4; stk11*	Y	*Dm*
partitioning defective 6 [188, 202]	*par-6*	N	*Dm*
combgap [3, 203]	*cg; mig*	Y	*Dm*
dynein heavy chain 64C; cytoplasmic dynein heavy chain [204-206]	*dhc64c; dhc*	Y	*Dm*
cut up [207]	*ddlc-1; cdlc1; dynein light chain*	Y	*Dm*
kinesin heavy chain [205, 208, 209]	*khc*	Y	*Dm*
kinesin light chain [208, 209]	*klc*	Y	*Dm*
rhomboid-2; stem cell tumor; brother of rhomboid [210]	*stet; rho-2*	N	*Dm*
ensconsin [211, 212]	*ens*	Y	*Dm*
*helicase at 25e; ATP-dependent RNA helicase; ddx39 (*in vertebrates*)* [213]	*hel25E; ddx39*	Y	*Dm*
licorne; similar to *dual specificity mitogen-activated protein kinase kinase 3;* similar to *dual specificity mitogen-activated protein kinase kinase (*in *Nasonia); dual specificity mitogen-activated protein kinase kinase 6 (*mainly in vertebrates*)* [183]	*lic; MAPKK; mek3*	Y	*Dm*
protein tyrosine phosphatase 10D [214]	*ptp10D*	Y	*Dm*
protein tyrosine phosphatase 4E; similar to *protein tyrosine phosphatase 10D* [214]	*ptp4E*	Y	*Dm*
**Genes influencing the cell cycle - regulators of mitosis (e.g. endocycling and selective amplification of chorion genes) and meiosis**			
archipelago; WD repeat domain containing 7 [23, 215]	*ago*	N	*Dm*
dacapo [215]	*dap*	N	*Dm*
coiled coil domain containing protein 25 [216]	*ccdc25*	Y	*Hs*
breast cancer 2, early onset homolog [217]	*brca2*	Y	*Dm*
chiffon [4, 218]	*chif*	N	*Dm*
cyclin-dependent kinase 1; cell division cycle 2 [219, 220]	*cdk1; cdc2*	Y	*Dm*
cyclin-dependent kinase 2 [221]	*cdk2*	Y	*Dm*
cyclin-dependent kinase 4 [221]	*cdk4*	Y	*Dm*
cyclin-dependent kinase 5 [222]	*cdk5*	Y	*Dm*
cyclin-dependent kinase 7 [223]	*cdk7; mo15*	Y	*Dm*
cyclin-dependent kinase 8 [3, 4]	*cdk8*	Y	*Dm*
cyclin-dependent kinase 9 [3]	*cdk9*	Y	*Dm*
cyclin-dependent kinase 10 homolog; cdc2-related kinase	*cdk10*	Y	*Its role in oogenesis not described*
cyclin A [224-226]	*cycA*	Y	*Dm*
cyclin B [219, 225, 226]	*cycB*	Y	*Dm*
cyclin B3; l(3)l6540 [219, 227]	*cycB3*	Y	*Dm*
cyclin C [228]	*cycC*	Y	*Dm*
cyclin D [221]	*cycD*	Y	*Dm*
cyclin E [218, 229]	*cycE*	N	*Dm*
COP9 complex homolog subunit 5 [230, 231]	*csn5*	Y	*Dm*
COP9 complex subunit 3 [230]	*csn3; dch3*	Y	*Dm*
COP9 complex subunit 4 [230]	*csn4; dch4*	Y	*Dm*

COP9 complex subunit 6 [230]	*csn6*	Y	*Dm*
COP9 complex subunit 7 [230]	*csn7*	Y	*Dm*
COP9 complex subunit 8 [230, 232]	*csn8*	Y	*Dm*
cyclin H [223]	*cycH*	Y	*Dm*
cyclin J [233, 234]	*cycJ*	N	*Dm*
cyclin K [3, 4]	*cycK*	Y	*Dm*
cyclin L1; CG16903 [4]	*cycL1*	Y	*Dm*
cyclin T [3, 4]	*cycT*	Y	*Dm*
cyclin fold protein; cyclin Y [4]	*cycfp; cycY*	Y	*Dm*
cyclin M2	*cycM2; cnnM2*	Y	*Its role in oogenesis not described*
cyclin-dependent kinase subunit 30a [227, 235]	*cks30a*	Y	*Dm*
cyclin-dependent kinase subunit 85a [235]	*cks85a*	Y	*Dm*
diminutive; dmyc [236]	*dm*	Y	*Dm*
e2f1 [229, 237, 238]	*e2f1*	Y	*Dm*
e2f5 [229, 237]	*e2f5*	N	*Dm*
dp; e2f dimerization partner 2 [229, 237]	*dp; tfdp2*	Y	*Dm*
sin3a [239]	*sin3a*	Y	*Dm*
geminin [240]	*geminin*	Y	*Dm*
matrimony [4, 241, 242]	*mtrm; d52*	N	*Dm*
imaginal discs arrested [243]	*ida*	N	*Dm*
twine [220, 244, 245]	*twe*	N	*Dm*
string; cdc25 phosphatase [215, 244, 245]	*stg*	N	*Dm*
microcephalin [246]	*MCPH1*	N	*Dm*
inducer of meiosis 4; mta70 homologue [247, 248]	*ime4*	Y	*Dm*
greatwall; mast-like [4, 92, 249]	*gwl*	Y	*Dm*
polo (kinase); l(3)01673 [164, 241, 249]	*polo*	Y	*Dm*
loki; checkpoint kinase 2 [83, 250, 251]	*lok; chk2*	Y	*Dm; Bm*
always early; a *lin9* homolog [54]	*aly*	Y	*Dm*
pavarotti; kinesin family member 23 [90, 252, 253]	*kif23; pav*	Y	*Dm*
morula (anaphase-promoting complex subunit) [254]	*mr*	Y	*Dm*
proliferating cell nuclear antigen (mutagen-sensitive 209) [255]	*mus209; pcna*	Y	*Dm*
mutagen-sensitive 304 [256]	*atrip; mus304*	N	*Dm*
myb oncogene-like [257, 258]	*myb*	Y	*Dm*
the myb-muvb complex subunit lin-52 [259]	*lin-52*	Y	*Dm*
myb transforming protein; similar to CG6905 [4]	*mybtp*	Y	*Dm*
pitchoune [260]	*pit*	Y	*Dm*
rad51(-like); spindle A [175]	*rad51; spna*	Y	*Dm*
tribbles [261]	*trbl*	Y	*Dm*
fizzy; cdc20 [227, 262]	*fzy; cdc20*	Y	*Dm*
accessory gland peptide 70a; sex peptide (transferred to female by male - no mRNA expected in female) [263]	*acp70a; sp*	N	*Dm*
meiotic 41 (which is the *Drosophila atm/atr homolog)* [264, 265]	*mei-41; fs(1)m37*	N	*Dm*
meiotic from via Salaria 332 [4, 266, 267]	*mei-S332*	N	*Dm*
mei-4 (Forkhead domain containing) [268]	*mei4*	Y	*Its role in oogenesis not described*
mei-W68 [264, 269]	*mei-W68*	N	*Dm*
cortex [266, 270, 271]	*cort*	Y	*Dm*
grauzone [266, 272]	*grau*	N	*Dm*

CG1647; zinc-finger protein [3, 4]	CG1647	Y	*Dm*
btk family kinase at 29a [109]	*btk29a; tec29a*	Y	*Dm*
mutator 2 [273, 274]	*mu2*	N	*Dm*
myelin transcription factor 1 [275]	*myt1*	N	*Dm*
orientation disrupter [276, 277]	*ord*	N	*Dm*
mei-218 [276, 277]	*mei-218*	N	*Dm*
altered disjunction; mps1 (a kinetochore-associated protein kinase) [278, 279]	*ald; mps1*	N	*Dm*
no distributive disjunction [280, 281]	*nod*	N	*Dm*
sarah; nebula [282]	*sra; nla*	Y	*Dm*
calcineurin a [283]	*cana*	Y	*Dm*
calcineurin b [283]	*canb*	Y	*Dm*
mei-38 [276]	*mei38*	N	*Dm*
ubiquitin conjugating enzyme E2 rad6	*ubcd6; rad6*	Y	*Its role in oogenesis not described*
alpha-endosulfine [220]	*endos*	Y	*Dm*
early girl; CG17033 [220]	*elgi*	Y	*Dm*
encore [6, 23, 265]	*enc*	N	*Dm*
cullin 1 [23]	*cul1; lin19*	Y	*Dm*
cullin 2 [284]	*cul2*	N	*Dm*
cullin 4 (a and b) [285]	*cul4*	Y	*Dm*
double parked [285, 286]	*dup*	Y	*Dm*
cullin 5 [284, 287]	*cul5*	Y	*Dm*
gustavus; Bombyx sequence BHIBMGA008896-PA homologous to spry domain-containing socs box protein 4 (ssb4) [287, 288]	*gus; ssb4*	Y	*Dm*
ubiquitin conjugating enzyme 2; l(2)k13206 [23]	*ubcd2*	Y	*Dm*
ubiquitin conjugating enzyme e2 d4 [267, 289]	*ubcd4*	Y	*Dm*
origin recognition complex subunit 1 [290, 291]	*ORC1*	Y	*Dm*
origin recognition complex subunit 2; l(3)88ab [238, 285, 286]	*ORC2*	Y	*Dm*
origin recognition complex subunit 5; l(2)34df [238, 292]	*ORC5*	Y	*Dm*
achintya [293, 294]	*zaa*	Y	*Dm*
vismay [293, 294]	*vis*	N	*Dm*
minichromosome maintenance 2 protein [238]	*mcm2*	Y	*Dm*
retinoblastoma-family protein 1 [238]	*rbf1; rb1*	N	*Dm*
grapes; serine/threonine-protein kinase chk1 [54, 295]	*chk1; lemp; grp*	N	*Dm*
missing oocyte [296, 297]	*mio*	N	*Dm*
megator [297]	*mtor*	Y	*Dm*
nucleoporin 44a; similar to *sec13-like protein* [297]	*seh1; nup44a*	Y	*Dm*
nucleoporin 154; tulipano [298, 299]	*nup154; zk; nup32d; tlp*	Y	*Dm*
kinesin-like protein ncd; non-claret disjunctional; claret segregational [300, 301]	*ncd*	Y	*Dm*
kinesin-13 motor; kinesin-like protein 10a; kinesin-like protein a (in *Bombyx mori*)[302]	*klp10a; klpa*	Y	*Dm*
similar to *Bombyx mori kinesin-like protein b*	*klpb*	Y	*Bm*
crossover suppressor on 2 of Manheim [4, 303-306]	*mei-910; c(2)M*	N	*Dm*
crossover suppressor on 3 of Gowen [303-306]	*c(3)G*	N	*Dm*
corona [303, 307]	*cona*	N	*Dm*

nipped-B [4, 308]	*nipped-B*	Y	*Dm*
pch2 [4, 306, 309]	*pch2*	N	*Dm*
Guanylate kinase-associated protein mars; hurp [310]	*hurp; dhrp/Gkap; mars*	Y	*Dm*
**Genes acting early in the egg to establish dorsal-ventral polarity (dorsal group)**			
cappuccino; formin 1/2 [143, 311, 312]	*capu*	Y	*Dm*
spire [312]	*spir*	Y	*Dm*
cornichon [313]	*cni*	Y	*Dm*
fs(1)k10 [314, 315]	*fs(1)k10*	N	*Dm*
sec61 beta [316]	*sec61 beta*	Y	*Dm*
mirror; iroquois-class homeodomain protein irx [317]	*mirr*	N	*Dm*
groucho; Enhancer of split m9/10 [318]	*gro; E(spl)m9/10*	Y	*Dm*
capicua [4, 318-320]	*cic*	Y	*Dm*
gurken [209, 314, 321-326]	*grk*	N	*Dm*
trailer hitch [4, 163]	*tral*	N	*Dm*
maelstrom [83, 327, 328]	*mael*	Y	*Dm*
pipe (encoding a sulfotransferase) [321, 324, 329, 330]	*pip*	N	*Dm*
okra (a spindle gene); rad54; rad54-like [331-333]	*okr; rad54*	Y	*Dm*
spindle B [175, 333]	*spnB*	N	*Dm*
spindle D [175, 333]	*spnD*	N	*Dm*
orb; oo18 RNA-binding protein [334]	*orb*	N	*Dm*
heterogeneous nuclear RNA-binding protein 40; squid [335-337]	*sqd; hrp40*	Y	*Dm*
heterogeneous nuclear ribonucleoprotein at 27c; similar to *Bombyx mori hnrnpa/b-like 28* [336, 338]	*hrp48; hrb27c; hnrnpa/b-like 28*	Y	*Dm*
heterogeneous nuclear ribonucleoprotein at 87f; similar to *Bombyx mori heterogeneous nuclear ribonucleoprotein a1* [337, 339]	*hrp36; p11*	Y	*Dm*
transportin; importin 3, karyopherin beta 2b [335]	*impβ2*	Y	*Dm*
**Genes acting in follicle cells early and late (including the dorsal group) and promoting their motility such as border cell migration (and in *Drosophila* important for choriogenesis and dorsal appendage formation)**			
capping protein beta [159]	*cpb*	Y	*Dm*
hepatocyte growth factor regulated tyrosine kinase substrate [340]	*hrs*	Y	*Dm*
Calpain-B [4, 341]	*CalpB*	N	*Dm*
big brain [342-344]	*bib*	N	*Dm*
brainiac [345]	*brn*	Y	*Dm*
mastermind [346-348]	*mam*	N	*Dm*
neuralized [4, 349]	*neur*	Y	*Dm*
derailed [350]	*drl; lio*	N	*Dm*
delta [348, 351]	*dl*	Y	*Dm*
notch; abruptex (ax), split (spl) [342, 348, 351]	*N*	Y	*Dm*
presenilin [352, 353]	*psn*	Y	*Dm*
nicastrin [354]	*nct*	Y	*Dm*
gamma-secretase subunit aph-1; anterior pharynx defective 1; presenilin-stabilization factor [355]	*aph1*	Y	*Dm*
presenilin enhancer [355]	*pen-2*	Y	*Dm*

strawberry notch [356]	*sno*	Y	*Dm*
notchless	*nle*	Y	*Its role in oogenesis not described*
cut; similar to *CCAAT displacement protein;* similar to *homeobox protein cut* [357, 358]	*ct; cux*	N	*Dm*
fringe [317, 359]	*fng*	Y	*Dm*
bunched; shortsighted [125]	*bun*	Y	*Dm*
dodo; similar to *Bombyx mori rotamase pin1* [360]	*dod*	Y	*Dm*
Broad-Complex core protein isoform 6 [361, 362]	*br; Br-C*	Y	*Dm*
zinc finger and BTB domain-containing protein weak homology to *Broad-Complex core protein isoforms 1, 2, 3, 4, 5* [361, 362]	*br; Br-C*	Y	*Dm*
daughterless [4, 348]	*da*	Y	*Dm*
ets at 97D; tiny eggs [4, 363, 364]	*ets97D; tny*	N	*Dm*
pointed; similar to *protein c-ets1* [4, 319, 350, 365, 366]	*pnt; D-ets-1*	N	*Dm*
dystroglycan [367]	*dg*	Y	*Dm*
discs lost; tight junction pdz protein patj [368]	*dlt*	Y	*Dm*
filamin; cheerio [369]	*fln; cher*	Y	*Dm*
jitterbug; filamin-related [370]	*jbug*	Y	*Dm*
leukocyte-antigen-related-like; tyrosine-protein phosphatase lar [214, 371]	*lar*	N	*Dm*
discs large [372]	*dlg1*	Y	*Dm*
scribble(d) [182, 373]	*scrib*	Y	*Dm*
singed [374]	*sn*	Y	*Dm*
slow border cells; homologous to *Bombyx C/EBP* [261, 370, 375, 376]	*slbo; bmC/EBP*	Y	*Dm; Bm*
midline fasciclin [370]	*mfas*	N	*Dm*
brinker [377, 378]	*brk*	Y	*Dm*
egf-r; torpedo; der [324, 361, 379]	*egfr; der*	Y?	*Dm*
rhomboid-1; rhomboid; veinlet [324, 380, 381]	*rho*	N	*Dm*
spitz [4, 48, 380]	*spi*	Y	*Dm*
ovarian serine protease encoding *nudel* [321, 382, 383]	*ndl*	Y	*Dm; Bm*
kekkon-1 [384]	*kek1*	N	*Dm*
vein (similar to a vertebrate *neuregulin)* [380]	*vn*	N	*Dm*
argos [380]	*aos*	Y	*Dm*
18 wheeler [324, 370, 385]	*18w*	Y	*Dm*
hopscotch [104, 340]	*hop; jak*	N	*Dm*
star; asteroid [4, 386]	*S*	N	*Dm*
keren; gritz [126]	*krn*	N	*Dm*
PDGF- and VEGF-receptor related [126]	*PVR*	Y	*Dm*
innexin 2 [4, 387, 388]	*inx2*	Y	*Dm*
innexin 3 [4, 387]	*inx3*	Y	*Dm*
zero population growth [4, 387, 388]	*inx4; zpg*	Y	*Dm*
crumbs [368, 389]	*crb*	Y	*Dm*
stardust; weakly similar to *maguk p55 subfamily member 5* [188]	*sdt; std*	Y	*Dm*
quit [43, 118]	*qui*	N	*Dm*
dual-specificity a-kinase anchor protein spoonbill; CG3249; homologous to *akap149* [390-392]	*spoon; yu*	N	*Dm*
lethal (2) giant larvae [393]	*lgl*	Y	*Dm*
myosin light chain 2; similar to *Bombyx mori myosin*	*mlc-2*	Y	*Dm*

regulatory light chain 2 [132]			
deep orange; Vacuolar sorting protein 18 [4, 394]	*dor; Vps18*	Y	*Dm*
Vacuolar protein sorting 9; sprint; rab GDP/GTP exchange factor (gef) [395]	*Vps9; spri*	Y	*Dm*
twinfilin [396]	*twf*	Y	*Dm*
toucan [397]	*toc*	Y	*Dm*
abrupt [398]	*ab*	N	*Dm*
taiman/ p160 coactivator fisc [398-402]	*DAIB1; tai*	Y	*Dm;* Various
puckered; hearty; similar to *dual specificity phosphatase 10* [390, 403]	*puc; hrt*	N	*Dm*
misshapen; traf2 and nck interacting kinase; homolog of *serine/threonine-protein kinase mig-15 (c. elegans)* [404]	*msn; tnik*	Y	*Dm*
fusilli; e(cacte10)7 [405]	*fus*	Y	*Dm*
dribble; krr1 small subunit processome component homolog [406]	*dbe*	Y	*Dm*
kuzbanian; similar to *disintegrin and metalloproteinase domain-containing protein 10* [370, 407]	*kuz*	Y	*Dm*
tie; tie-like receptor tyrosine kinase [370]	*tie*	N	*Dm*
fk506-binding protein (fkbp13) [370]	*fkbp13*	Y	*Dm*
m6; myelin protolipid [408]	*m6*	Y	*Dm*
tanc2-like rolling pebbles; antisocial [132]	*ants; rols*	Y	*Dm*
amphiphysin; bridging integrator [409]	*damph*	Y	*Dm*
fasciclin II [409]	*fas2*	N	*Dm*
semaphorin; fasciclin-IV [4]	*fas4; sema-1a*	Y	*Dm*
kayak [410]	*kay; fos*	Y	*Dm*
src homology 2, ankyrin repeat, tyrosine kinase [411, 412]	*shark*	Y	*Dm*
bullwinkle [411, 413]	*bwk*	N	*Dm*
basket; jun amino terminal kinase (djnk); c-jun nh2-terminal kinase [411]	*bsk*	Y	*Dm*
Cad74A [324, 414]	*Cad74A*	N	*Dm*
locomotion defects; regulator of g protein signaling (rgs) [415]	*loco*	Y	*Dm*
blistered; serum response factor; pruned [416]	*bs; serf*	N	*Dm*
calmodulin-binding protein related to a rab3 gdp/gtp exchange protein; weakly similar to *denn domain-containing protein 4c* [417]	*crag*	Y	*Dm*
G protein-coupled receptor kinase 1; similar to *beta-adrenergic receptor kinase 2* [418]	*Gprk1*	Y	*Its role in oogenesis not described*
G protein-coupled receptor kinase 2; similar to *beta-adrenergic receptor kinase 1* [419]	*Gprk2*	Y	*Dm*
rutabaga; similar to *ca(2+)/calmodulin-responsive adenylate cyclase;* similar to *adenylate cyclase 1* [419]	*rut*	Y	*Dm*
dunce; cAMP-specific 3',5'-cyclic phosphodiesterase [4, 419]	*dnc*	Y	*Dm*
jun related antigen [420]	*jra*	Y	*Dm*
myocardin-related transcription factor [421]	*mrtf*	Y	*Dm*
similar to *rolling stone* [132, 422]	*rost*	Y	*Dm*
jing [423]	*jing*	N	*Dm*
yan; anterior open; similar to *ets DNA-binding protein pokkuri* [365, 424]	*aop*	Y	*Dm*
adherens junction protein p120; armadillo repeat	*p120ctn*	Y	*Dm*

protein; catenin delta; CG17484 [425]			
G protein sα 60a; G protein alpha s subunit GS1 (Bombyx mori) [426]	*G-salpha60a*	N	*Dm*
protein tyrosine phosphatase 99a [214]	*ptp99a*	N	*Dm*
diacyl glycerol kinase ε [427]	*dgkε*	N	*Dm*
ovary protein-29kD [428]	*op29*	N	*Dm*
ran-binding protein m [429]	*ranbpm*	Y	*Dm*
**Follicle ring canal genes**			
weakly similar to *Drosophila melanogaster visgun* [4, 253]	*vsg*	Y	*Dm*
female sterile (1) nasrat [253, 430, 431]	*fs(1)nas*	N	*Dm*
weakly similar to *actin-binding protein anillin; scraps* [4, 432]	*ani; scra*	Y	*Dm*
**Terminal genes**			
corkscrew; similar to *protein tyrosine phosphatase, non-receptor type 11* [214, 433, 434]	*csw; ptpn11*	Y	*Dm*
dead ringer [332, 435, 436]	*dri*	Y	*Dm*
torso [4, 437-439]	*tor*	N	*Dm*
torsolike [437, 438]	*tsl*	Y	*Dm*
trunk [46, 437, 438, 440]	*trk*	N	*Dm*
female sterile (1) homeotic; fragile-chorion membrane protein [441]	*fs(1)h*	Y	*Dm*
ras1 [442]	*ras1; ras85d*	Y	*Dm*
raf; raf1; pole hole; raf kinase; effector of ras [443]	*raf; raf1; phl*	Y	*Dm*
signal transducer and activator (stat) [444, 445]	*stat; stat92e*	Y	*Dm*
rolled; map kinase (MAPK) [445]	*rl; MAPK; erk*	Y	*Dm*
downstream of raf1 [446]	*dsor1*	N	*Dm*
hemipterous; mitogen-activated protein kinase kinase [447]	*hep; MAPKK; mkk7*	Y	*Dm*
growth arrest and DNA-damage inducible 45 [447]	*gadd45*	N	*Dm*
shc-adaptor protein; shc-transforming protein 1; src homology 2 domain containing; CG3715 [89, 324]	*shc*	N	*Dm*
**Ovarian nuage genes and genes involved in piRNA pathway**			
capsuléen; Arginine n-methyltransferase 5 [448]	*csul; prmt5*	Y	*Dm*
valois [4, 46, 448]	*vls*	N	*Dm*
aubergine (related to eIF2c; a piwi protein) [33, 449-451]	*aub*	Y	*Dm*
ATP-dependent helicase; *cap; belle* [452, 453]	*cap; bel*	Y	*Dm*
cutoff [454]	*cuff*	N	*Dm*
squash [450]	*squ*	N	*Dm*
piwi-like protein; argonaute 3 [455, 456]	*AGO3; siwi*	Y	*Dm; Bm*
zucchini [4, 27, 450]	*zuc*	N	*Dm*
tudor; similar to *tudor domain containing 6* [46, 457-459]	*tud*	Y	*Dm*
krimper [27, 460]	*mtc; krimp*	N	*Dm*
tejas; similar to *tudor domain containing 5* [27, 461]	*tej; TDRD5*	Y	*Dm*
vreteno; similar to CG4771 [4, 27]	*vret*	N	*Dm*
similar to *tudor domain containing* CG9925 and	*TDRD1*	Y	*Dm*

CG9684 [4, 27]			
similar to CG8920; similar to *tudor domain containing 7* [4, 27]	*TDRD7*	Y	*Dm*
homeless; fs(3); spindle E; similar to *tudor domain containing 9* [27, 175, 462]	*hls; spnE; TDRD9*	Y	*Dm*
CG14303*; similar to tudor domain containing 4* [27]	*TDRD4*	N	*Dm*
tudor-SN [4, 33, 463]	*tudor-SN*	Y	*Dm*
Brother of Yb; CG11133 [4, 27, 464]	*BoYb*	N	*Dm*
Sister of Yb; CG31755 [27, 464]	*SoYb*	N	*Dm*
**Ovarian processing bodies**			
Nonsense-mediated mRNA 3 [4, 465, 466]	*Nmd3*	Y	*Dm*
regulator of nonsense transcripts 1; nonsense mRNA reducing factor 1; up-frameshift suppressor 1 homolog [465, 466]	*rent1; norf1; Upf1*	Y	*Its role in oogenesis not described*
similar to *Upf2 regulator of nonsense transcripts homolog* [465, 466]	*Upf2*	Y	*Its role in oogenesis not described*
similar to *Bombyx mori Upf3 regulator of nonsense transcripts-like protein B* [465, 466]	*Upf3*	Y	*Its role in oogenesis not described*
no-on-and-no-off-transient C [465, 466]	*smg1*	Y	*Its role in oogenesis not described*
smg5 [4, 465, 466]	*smg5*	Y	*Dm*
telomerase-binding protein est1a; similar to *smg6 homolog, nonsense mediated mRNA decay factor* [4, 465, 466]	*smg6*	Y	*Dm*
decapping protein 1 [467, 468]	*Dcp1*	Y	*Dm*
decapping protein 2 [467]	*Dcp2*	Y	*Dm*
pacman; 5'-3' exoribonuclease 1 [467, 469]	*XRN1; pcm*	N	*Dm*
EDC4; Ge-1 [4, 470]	*Ge-1*	N	*Dm*
**Posterior group genes**			
apontic [4, 471]	*apt*	N	*Dm*
nanos; nanos-like (LOC100125608) [472-477]	*nos-like*	Y	*Dm; Bm;* Various
nanos-M [472, 473, 477]	*nos-M*	Y	Lepidoptera
nanos-P [472, 473, 477]	*nos-P*	N	Lepidoptera
nanos-O [472, 473, 477]	*nos-O*	Y	Lepidoptera
shavenbaby; ovo [4, 63]	*ovo*	Y	*Dm*
armitage [4, 27, 478]	*armi*	Y	*Dm*
*arrest (*also known as *bruno)* [4, 479-482]	*aret/bru*	Y	*Dm*
lasp [483]	*lasp*	Y	*Dm*
oskar [4, 143, 186, 448, 470, 478, 483-492]	*osk*	N	*Dm;* Various
poly(a)-binding protein [4, 493]	*pAbp*	Y	*Dm*
Eukaryotic translation initiation factor 4AIII [488]	*eIF4AIII*	Y	*Dm*
barentsz; eIF4aIII binding protein; weak localizer [487, 488]	*wkl; btz*	Y	*Dm*
syntaxin 1a [157]	*syx1a*	Y	*Dm*
moesin-like; dmoesin (ezrin, radixin, moesin gene) [491]	*moe; ERM1*	Y	*Dm*
Eukaryotic translation initiation factor 4e	*cup; fs(2)cup;*	Y	*Dm*

transporter similar to *cup* [113]	*fs(1)cup*		
Eukaryotic translation initiation factor 2α [494]	*eIF2alpha*	Y	*Dm*
miranda [143, 489, 495]	*mira*	N	*Dm*
mago nashi [4, 496]	*mago*	Y	*Dm*
tsunagi/y14 [496]	*tsu/y14*	Y	*Dm*
ranshi; similar to *zinc finger protein 195;* CG9793 [496]	*ranshi*	Y	*Dm*
glorund [4, 497]	*glo; p67*	N	*Dm*
smaug [4, 492, 497]	*smg*	Y	*Dm*
twin; CCR4 (part of CCR4-Not complex*)* [4, 498, 499]	*twin; CCR4*	N	*Dm*
not1 (part of CCR4-Not complex*)* [4, 498, 499]	*Not1*	Y	*Dm*
not2 (part of CCR4-Not complex*); Regena* [4, 498, 499]	*Not2; Rga*	Y	*Dm*
not3 (part of CCR4-Not complex*); l(2)nc136* [63, 498, 499]	*Not3*	Y	*Dm*
chromatin assembly factor 1 (part of CCR4-Not complex*);* similar to CG4236 [492, 498, 499]	*caf1*	Y	*Dm*
Pop2; similar to CG5684*; CCR4-Not transcription complex subunit 7* [4, 492, 498, 499]	*Pop2*	Y	*Dm*
hiiragi (Poly A Polymerase) [4, 500]	*hrg; PAP*	Y	*Dm*
rabenosyn-5; rabenosyn [501]	*rbsn-5*	Y	*Dm*
ypsilon schachtel (Bombyx mori Y-box protein) [502]	*yps; ybp*	Y	*Dm*
ubiquitin specific protease 9; fat facets [503]	*faf*	Y	*Dm*
hephaestus; polypyrimidine tract-binding protein; heterogeneous nuclear ribonucleoprotein I [4, 504]	*heph; ptb; hnrnp I*	Y	*Dm*
synaptotagmin [157]	*syt 1; syt*	Y	*Dm*
synaptotagmin; similar to *Drosophila melanogaster extended synaptotagmin 2* [157]	*esyt2*	Y	*Dm*
**In relation to oskar and pole plasm - control of endocytosis in germline and germline viability**			
rab-protein 11 [182, 505]	*rab11*	Y	*Dm*
rab-protein 5 [4, 506]	*rab5*	Y	*Dm*
skittles; pip5k (type 1*)* [4, 506, 507]	*pip5k*	Y	*Dm*
rap1 GTPase activating protein [508]	*rapgap*	Y	*Dm*
germ cell-less [4, 509, 510]	*gcl*	N	*Dm*
stambha a; CG8739*; protein efr3 homolog b; rolling blackout* [509]	*cmp44e ; stma*	Y	*Dm*
myoglianin [511, 512]	*myo; myg*	N	*Dm*
mitochondrial small ribosomal RNA [513]	*mtsrRNA; 12s rRNA*	N	*Dm*
**Anterior system genes**			
bicoid [4, 143, 172, 322, 475, 514-520]	*bcd*	N	*Dm*
muscle excess 3 [521]	*mex-3*	Y	*Tc;* Various
orthodenticle; Drosophila ocelliless [477, 518, 522-525]	*oc; otd*	Y	*Nv*; Various
exuperantia [4, 46, 516, 526]	*exu*	Y	*Dm; Am*
swallow; fs(1)1502 [4, 527-529]	*swa*	N	*Dm*
maternal expression at 31B [502]	*me31B*	Y	*Dm*
staufen [4, 46, 475]	*stau*	Y	*Dm; Am*
bicoid-interacting protein 3 [4, 172]	*bin3*	Y	*Dm*

larp1 [4, 172]	*larp1*	Y	*Dm*
Eukaryotic initiation factor 4E; similar to *Bombyx mori Eukaryotic initiation factor 4E-2* [4, 172]	*eIF4E*	Y	*Dm*
argonaute 2 [172]	*AGO2*	Y	*Dm*
caudal [4, 172, 477, 525, 530-533]	*cad*	Y	*Dm;* Various
hunchback [4, 35, 36, 523-525, 534-536]	*hb*	N	*Dm;* Various
**Chromatin regulation during oogenesis; general transcription; maternal regulation of zygotic gene expression**			
DNA polymerase α 180KD; DNA polymerase alpha catalytic subunit [537]	*DNApol-α180*	Y	*Dm*
RNA polymerase II transcriptional coactivator single stranded-binding protein c31a [538]	*ssb-c31a*	Y	*Dm*
polyadenylate-binding protein 2 [91, 539]	*rox2; pabp2*	Y	*Dm*
high mobility group protein; structure specific recognition protein. fact complex subunit ssrp1 [540]	*ssrp; ssrp1*	Y	*Dm*
similar to *Drosophila melanogaster high mobility group protein d;* similar to *Bombyx mori high mobility group protein 1b* [541]	*HMGd; HMG1b*	Y	*Dm*
domina; jumeau [542]	*jumu/dom*	Y	*Dm*
modulo [543]	*mod*	N	*Dm*
lysine-specific histone demethylase 1; suppressor of variegation 3-3 [544]	*suv3-3; su(var)3-3; lsd1*	Y	*Dm*
histone methyltransferase 4-20; suppressor of variegation 4-20 [3, 4]	*suv4-20; su(var)4-20*	Y	*Dm*
Drosophila melanogaster suppressor of variegation 3-9 [545, 546]	*suv3-9; su(var)3-9*	Y	*Dm*
pitkin(dominant) [547]	*ptn(d)*	N	*Dm*
Eukaryotic translation initiation factor 2 gamma subunit [545, 546]	*eIF2g*	Y	*Dm*
suppressor of variegation 2-10; protein inhibitor of activated stat [548]	*su(var)2-10; pias; zimp; zimpb;*	Y	*Dm*
eggless [4, 545, 549]	*egg; SETDB1*	Y	*Dm*
histone h3k9 methyltransferase dg9A [550]	*g9A*	N	*Dm*
modifier of mdg4 [85, 551]	*mod(mdg4); e(var)3-93d*	Y	*Dm*
suppressor of hairy wing [551-554]	*su(hw)*	Y	*Dm*
trithorax-like [4, 555]	*trl; GAGA; gaf; e(var)3; e(var)62*	N	*Dm*
brahma; SWI/SNF-related matrix-associated actin-dependent regulator of chromatin subfamily A member; transcription activator brg1 [556, 557]	*smarca4; brm*	Y	*Dm*
marcal1; SWI/SNF-related matrix-associated actin-dependent regulator of chromatin subfamily A member [3]	*marcal1; smarcal1*	Y	*Its role in oogenesis not described*
snf5-related 1; SWI/SNF-related matrix-associated actin-dependent regulator of chromatin subfamily B member 1 [557]	*snr1; bap45*	Y	*Dm*
brg-1 associated factor; SWI/SNF-related matrix-associated actin-dependent regulator of chromatin subfamily d member 1; brahma associated protein 60kD [3, 558]	*bap60*	Y	*Dm*
dalao; brahma-associated protein 111kD; SWI/SNF-related matrix-associated actin-dependent regulator of chromatin subfamily E [3, 4]	*bap111; dalao*	Y	*Dm*

moira [559]	*mor; bap155*	Y	*Dm*
imitation swi [556]	*dnurf; iswi; dchrac*	Y	*Dm*
Brahma associated protein 170kD [560]	*bap170*	Y	*Dm*
Brahma associated protein 55kD [3, 4]	*bap55*	Y	*Dm*
helicase *domino* [561]	*dom*	Y	*Dm*
etl1 homologue; SWI/SNF-related matrix-associated actin-dependent regulator of chromatin subfamily A containing dead/h box 1 [3, 4]	*etl1; smarcad*	Y	*Dm*
Enhancer of zeste [4, 562]	*E(z)*	Y	*Dm*
extra sex combs [3, 563]	*esc*	Y	*Dm*
additional sex combs [564]	*asx*	Y	*Dm*
sex comb on midleg [565]	*scm*	N	*Dm*
multi sex combs [566]	*mxc*	N	*Dm*
polyhomeotic [567]	*ph-p*	N	*Dm*
sex combs extra; similar to *E3 ubiquitin-protein ligase ring1 (Bombyx mori)* [3, 564, 567]	*sce; dring*	Y	*Dm*
polycomb [568]	*ph*	Y	*Dm*
Enhancer of polycomb [4, 569]	*E(pc)*	Y	*Dm*
posterior sex combs [570, 571]	*psc*	Y	*Dm*
lethal (3) 73ah; similar to *polycomb group ring finger protein 3* [572]	*l(3)73ah*	Y	*Dm*
activating transcription factor; homologous to *Bombyx activating transcription factor of chaperone* [573]	*atf-2*	Y	*Dm*
cyclic-amp response element binding protein (1,2,3)[574-576]	*creb; dcreba*	Y	*Dm*
creb binding protein; similar to *nejire*	*crebbp(a)*	Y	*Its role in oogenesis not described*
retinoblastoma binding protein	*rbp*	Y	*Its role in oogenesis not described*
retinoblastoma binding protein 2 (jumonji/arid domain containing); little imaginal discs [577]	*rbp2; lid*	Y	*Dm*
similar to *retinoblastoma binding protein 6*	*rbp6*	Y	*Its role in oogenesis not described*
tousled-like kinase [578]	*tlk*	Y	*Dm*
no child left behind; similar to *wd repeat protein* [579]	*nclb*	Y	*Dm*
Arginine methyltransferase 1; Arginine n-methyltransferase 1 [580]	*DART1; prmt1*	N	*Dm*
Arginine methyltransferase 2; Arginine n-methyltransferase 2 [580]	*DART2; prmt2*	N	*Dm*
Arginine methyltransferase 3; Arginine n-methyltransferase 3 [580]	*DART3; prmt3*	Y	*Dm*
Arginine methyltransferase 4; histone-Arginine methyltransferase carm 1 [580]	*DART4; prmt4*	Y	*Dm*
Arginine methyltransferase 6; Arginine n-methyltransferase 6 [580]	*DART6; prmt6*	N	*Dm*
Arginine methyltransferase 7; Arginine n-methyltransferase 7 [580]	*DART7; prmt7*	Y	*Dm*
Arginine methyltransferase 8; Arginine n-methyltransferase 8 [580]	*DART8; prmt8*	N	*Dm*
Arginine methyltransferase 9; Arginine n-methyltransferase 9 [580]	*DART9; prmt9*	N	*Dm*
absent, small, or homeotic discs 1 [581]	*ash-1; ash; dash*	Y	*Dm*
bj1 protein; homolog of *regulator of chromatin*	*rangef; rcc1*	Y	*Dm*

condensation 1 [582]			
homolog of *regulator of chromatin condensation 2;* similar to CG9135 [3]	*rcc2*	Y	*Its role in oogenesis not described*
DNA polymerase interacting tpr containing protein [3]	*dpit47*	Y	*Its role in oogenesis not described*
DNA polymerase α (180kD) [537]	*DNApol-α180; pola*	Y	*Dm*
DNA polymerase delta [583]	*DNApol-delta*	Y	*Dm*
DNA polymerase ε [584]	*DNApol-ε; pole*	Y	*Dm*
similar to *DNA polymerase ε subunit 2* [584]	*DNApol-ε; pole2*	Y	*Dm*
similar to *DNA polymerase ε subunit 3* [584]	*DNApol-ε; pole3*	Y	*Dm*
DNA polymerase eta [3]	*DNApol-eta; drad30a*	Y	*Its role in oogenesis not described*
DNA polymerase iota [3]	*drad30b; DNApol-iota*	Y	*Its role in oogenesis not described*
DNA polymerase zeta; similar to *mutagen-sensitive 205; rev3-like* [585]	*DNApol-zeta; mus205*	Y	*Dm*
replication protein a1 [586]	*rpa1*	Y	*Dm*
replication protein a2 [587]	*rpa2*	Y	*Dm*
replication protein a3	*rpa3*	Y	*Its role in oogenesis not described*
replication factor c 38kD subunit [238]	*rfc38*	Y	*Dm*
(Bombyx mori) replication factor c subunit 2; rfc40 [238]	*rfc40; bm- rfc2*	Y	*Dm*
(Bombyx mori) replication factor c4; CG8142 [238]	*bm-rfc4*	Y	*Dm*
(Bombyx mori) replication factor c (activator 1) 5; Drosophila replication factor c subunit 3 [238]	*rfc3*	Y	*Dm*
germ line transcription factor 1; replication factor 1 [238, 588]	*rfc1; gnf1*	Y	*Dm*
recombination repair protein 1 [585, 589]	*rrp1*	Y	*Dm*
rev7 [585]	*rev7*	N	*Dm*
trf4-1; sigma DNA polymerase	*trf4-1*	Y	*Its role in oogenesis not described*
topoisomerase 1; topoisomerase i [590-592]	*top1*	Y	*Dm*
topoisomerase 2; topoisomerase II [54]	*top2; topII*	Y	*Dm*
topoisomerase 3 alpha; topoisomerase III aplha [3]	*topIII-alpha*	Y	*Its role in oogenesis not described*
topoisomerase 3 beta; topoisomerase III beta [593]	*topIII-beta*	Y	*Dm*
minichromosome maintenance 3 [594]	*mcm3*	Y	*Dm*
minichromosome maintenance 5 [594, 595]	*mcm5*	Y	*Dm*
minichromosome maintenance 6; fs(1)k1214 [594, 595]	*mcm6*	Y	*Dm*
minichromosome maintenance 7 [594]	*mcm7*	Y	*Dm*
minichromosome maintenance 8; recombination-defective [596]	*mcm8; rec*	Y	*Dm*
DNA methyltransferase 2 [597]	*mt2*	Y	*Dm*
poly-(adp-ribose) polymerase [598]	*parp*	Y	*Dm*
TATA box binding protein-related factor 2 [63, 599]	*Trf2; tlf*	N	*Dm*
TATA box binding protein [599]	*Tbp*	Y	*Dm*
tbp-associated factor 250kD [600]	*taf250; taf1*	Y	*Dm*
trithorax-related [601]	*trr*	Y	*Dm*
supercoiling factor	*scf; dcb-45*	Y	*Dm*

bx42; ski-interacting protein [602]	*skip*	Y	*Dm*
boundary element-associated factor of 32KD [603]	*beaf32*	N	*Dm*
Histone h4 [604]	*H4*	Y	*Dm*
Histone h3.3 [605]	*H3.3*	Y	*Dm*
Histone h2a [604, 606]	*H2a*	Y	*Dm*
Histone h2a variant [606, 607]	*H2a.v*	Y	*Dm*
mutagen-sensitive 308 [4, 608]	*PolQ; mus308*	Y	*Dm*
rpd3 [609]	*hdac1; rpd3; hdac*	Y	*Dm*
mbd-like [610]	*mbd2/3; mbd-like*	Y	*Dm*
mediator complex subunit 6 [611]	*med6*	Y	*Dm*
mitochondrial single stranded DNA-binding protein [612]	*mtssb*	Y	*Dm*
homolog of *recq* [613]	*recq5*	Y	*Dm*
hen1 [614]	*dmhen1; pimet*	Y	*Dm*
Eukaryotic translation initiation factor 4G [172]	*eIF4G*	Y	*Dm*
Eukaryotic translation initiation factor 4A [615]	*eIF4A*	Y	*Dm*
Eukaryotic translation initiation factor 5 [92]	*eIF5*	Y	*Dm*
retrotransposon gypsy\envelope [616-618]	*gypsy\env*	N	*Dm*
jim [619]	*ovk; ovfc.k; jim*	Y	*Dm*
zelda; vielfaltig [4, 620-622]	*vfl; zld*	N	*Dm*
Fcp1 RNA polymerase II CTD phosphatase; CG12252 [623]	*fcp1*	Y	*Dm*
**Alternative splicing**			
poly-u-binding splicing factor; half pint [624]	*pubsf; hfp; puf60/68*	Y	*Dm*
peanuts; ATP-dependent RNA helicase *dhx8; deah box protein 8* [33, 463]	*pea; prp22*	Y	*Its role in oogenesis not described in great detail*
p-element somatic inhibitor [338]	*psi*	Y	*Dm*
**Other genes involved in oogenesis (including mitochondrial functioning)**			
clueless; CG8443*; Eukaryotic translation initiation factor 3 subunit* [625]	*clu*	Y	*Dm*
*enabled; vasp (*similar to *vasodilator-stimulated phosphoprotein)* [159]	*ena*	N	*Dm*
**Maternal effect genes I - Ribosomal machinery needed for increased ovarian protein synthesis and early embryogenesis**			
nop5 [626]	*nop5*	Y	*Dm*
ribosomal protein l8 [627]	*rpl8*	Y	*Dm*
ribosomal protein l40; ubiquitin 52-aa extension protein [628]	*rpl40; dub52*	Y	*Dm*
ribosomal protein s27a [629]	*rps27a; dub80*	Y	*Dm*
ribosomal protein lp2; ribosomal protein a1; 60s acidic ribosomal protein p2 [630, 631]	*rplp2*	Y	*Dm*
ribosomal protein l32; ribosomal protein 49 [632]	*rpl32; rp49*	Y	*Dm*
ribosomal protein s3a [633]	*rps3a; c3*	Y	*Dm*
pescadillo; CG4364 [4]	*pesc*	Y	*Dm*
minifly; nucleolar protein at 60b; similar to *h/aca ribonucleoprotein complex subunit 4* [634, 635]	*nop60b*	Y	*Dm*
**Maternal effect genes II - (encoding various types of proteins including enzymes)**			

needed for early embryogenesis and germ cell formation - maternal transcripts present			
homologous to *Bombyx UDP-glucosyltransferase protein 3* [636]	*ugt3*	Y	*Its role in oogenesis not described*
mrityu [637]	*mri*	Y	*Dm*
similar to *en protein binding/engrailed nuclear homeoprotein-regulated protein; msr-110* [4]	*msr-110*	Y	*Dm*
UDP-glucose-glycoprotein glucosyltransferase [636]	*ugt*	Y	*Dm*
abstrakt [638, 639]	*abs*	Y	*Dm; Bm*
terribly reduced optic lobes; perlecan; zeste-white 1 [640]	*trol; pcan; zw1*	Y	*Dm*
TBC1 domain family member 1; weakly similar to *Drosophila melanogaster pollux* [641]	*plx*	Y	*Dm*
no poles [642]	*nopo*	Y	*Dm*
ariadne 1 [643]	*ari-1*	Y	*Dm*
ariadne 2 [643]	*ari-2*	Y	*Dm*
ubiquitin conjugating enzyme e2 - homolog of *ubc7; courtless* [54, 644]	*crl*	Y	*Dm*
out at first [645]	*oaf*	Y	*Dm*
extra macrochaetae [646, 647]	*emc*	Y	*Dm*
wings up a; troponin 1 [648]	*tn1; tpn1; wupa*	Y	*Dm*
troponin c	*tpnc; tnc47d*	Y	*Its role in oogenesis not described*
troponin t; wings up b; upheld [132]	*tpnt; wupb*	Y	*Dm*
tropomyosin 1 or 2 [490, 649]	*tm1; tm2*	Y	*Dm*
alcohol dehydrogenase [4, 650, 651]	*adh*	Y	*Dm*
polar granule component [4, 652, 653]	*pgc*	N	*Dm*
type III alcohol dehydrogenase; iron-containing dehydrogenase [652]	*t3dh; adhfe1*	Y	*Dm*
plutonium [653-655]	*plu*	N	*Dm*
pan gu [653-655]	*png*	N	*Dm*
giant nuclei [653-655]	*gnu*	N	*Dm*
germ cell guidance factor wunen; phosphatidate phosphatase [656]	*wun*	Y	*Dm*
receptor for activated protein kinase c rack 1 [657, 658]	*rack1*	Y	*Dm*
shuttle craft; transcriptional repressor nf-x1 [659]	*stc*	Y	*Dm*
muscleblind [660]	*mbl*	Y	*Dm*
grainyhead [661]	*NTF-1; grh*	Y	*Dm*
dorsal (Drosophila); embryonic polarity protein dorsal (Bombyx - 2 isoforms*)* [662-665]	*dl*	Y	*Dm*
dorsal switch protein [63]	*dsp1; ssrp2*	Y	*Dm*
tosca; exonuclease 1 [666]	*tos*	Y	*Dm*
Darkener of apricot; dual specificity protein kinase clk2 [4, 667]	*Doa*	Y	*Dm*
clipper; cleavage and polyadenylation specific factor 4 [668]	*clp; cpsf30*	Y	*Dm*
vrille [669]	*vri; jf23*	Y	*Dm*
absent md neurons and olfactory sensilla [670]	*amos*	N	*Dm*
baboon; activin receptor type 1 [671]	*ATR1*	Y	*Dm*
eyelid; osa [668]	*eld; osa*	Y	*Dm*
gonadal [672]	*gdl*	Y	*Dm*
éclair; transmembrane emp24 protein transport domain containing 9 [4, 64]	*eca*	Y	*Dm*

baiser; transmembrane trafficking protein [4, 64]	*bai*	Y	*Dm*
logjam [673, 674]	*loj*	Y	*Dm*
p24-related-1; CG1967*;* similar to *membrane trafficking protein emp24/gp25/p24 family member* [673]	*p24-1*	Y	*Dm*
CG3564*; transmembrane emp24 domain-containing protein 2; copi-coated vesicle membrane protein p24* [673]	*chop24*	Y	*Dm*
bancal; (similar to) *heterogeneous nuclear ribonucleoprotein K* [675]	*hrb57A; q18*	Y	*Dm*
maternal transcript 89BA [676]	*mat89BA*	N	*Dm*
asunder; maternal transcript 89BB [676]	*mat89BB; asun*	Y	*Dm*
diadenosine tetraphosphatase; similar to *bis(5-nucleosyl)-tetraphosphatase* [676]	*datp*	Y	*Dm*
dopa decarboxylase; aromatic-l-amino-acid decarboxylase [677-679]	*ddc*	Y	*Dm*
hairless [680, 681]	*h*	N	*Dm*
suppressor of hairless; j kappa-recombination signal-binding protein [680, 682]	*su(h)*	Y	*Dm*
transcription termination factor lodestar; horka [683]	*horka; ids*	Y	*Dm*
raspberry; inosine monophosphate dehydrogenase [684]	*ras*	Y	*Dm*
misato [685, 686]	*mst; lb20*	Y	*Dm*
peanut; similar to *septin 7* [416, 687, 688]	*pnut*	Y	*Dm*
septin 1; innocent bystander [687, 688]	*sep-1; iby*	Y	*Dm*
septin 2 [687]	*sep-2*	Y	*Dm*
septin and tuftelin interacting protein; elongator complex protein 2; septin interacting protein 1 [689]	*stip*	Y	*Dm*
kurz; similar to ATP-*dependent RNA helicase dhx37* [315]	*kz*	Y	*Dm*
pebble [690]	*pbl*	Y	*Dm*
numb [691]	*numb; nb*	Y	*Dm*
catalase [4, 692]	*cat*	Y	*Dm*
superoxide dismutase [693, 694]	*sod1; csod; cu/znsod*	Y	*Dm*
disc proliferation abnormal [695]	*mcm4; dpa*	Y	*Dm*
Fragile x mental retardation 1 [696-699]	*Fmr1*	Y	*Dm*
female sterile (2) ketel; karyopherin beta 1; importin β [700, 701]	*ketel; imp-beta*	Y	*Dm*
karyopherin beta 3 [700, 702]	*karyβ3*	Y	*Dm*
cas/cse1 segregation protein; export karyopherin cas/cse1p [700]	*cas*	Y	*Dm*
importin alpha 1; karyopherin α1 [700]	*imp alpha 1*	Y	*Dm*
importin alpha 2; karyopherin α2; pendulin [700, 703]	*imp alpha 2*	Y	*Dm*
importin alpha 3; karyopherin α3 [700]	*imp alpha 3*	Y	*Dm*
imaginal disc growth factor 1 [704]	*idgf; idgf1*	Y	*Dm*
imaginal disc growth factor 2 [704]	*idgf2*	N	*Dm*
imaginal disc growth factor 3 [704]	*idgf3*	N	*Dm*
imaginal disc growth factor 4 [704]	*idgf4*	N	*Dm*
kinesin-like protein at 61f; urchin; kinesin-like protein klp2 (in Bombyx mori) [152, 705]	*klp61f; klp2*	Y	*Dm*
kinesin-like protein at 67a; kinesin 8 [152]	*klp67a; klp3*	N	*Dm*
puromycin sensitive aminopeptidase [706]	*psa*	Y	*Dm*

cask ortholog; calmodulin-dependent kinase [707]	*caki; cmg; camguk*	Y	*Dm*
signal transducing adaptor molecule [149, 708]	*stam*	Y	*Dm*
histone acetyltransferase kat2b; histone acetyltransferase pcaf; general control of amino acid synthesis protein 5-like 2 [709]	*pcaf; gcn5*	Y	*Dm*
ada2b [709]	*ada2b*	Y	*Dm*
s-adenosyl-methyl transferase mraw; CG14683	*mraw*	Y	*Its role in oogenesis not described*
c-terminal binding protein; hairy-interacting protein; similar to *2-hydroxyacid dehydrogenase* [710]	*ctbp*	Y	*Dm*
reticulated [711]	*ret*	N	*Dm*
furin 1; similar to *convertase subtilisin/kexin;* similar to *furin-like convetase* [712]	*fur1*	N	*Dm*
windbeutel; thioredoxin-like motif containing gene [321, 713]	*wbl*	Y	*Dm*
jafrac1; thioredoxin peroxidase 1; thiol peroxiredoxin [714]	*jafrac1; dpx-4783*	Y	*Dm*
deadhead; thioredoxin [715]	*trx-1; trx*	N	*Dm*
thioredoxin-like; similar to *Bombyx mori thioredoxin*	*trxl*	Y	*Its role in oogenesis not described*
thioredoxin-2; similar to *Bombyx mori thioredoxin-like* [716]	*trx2*	Y	*Dm*
yema gene 2.8 [717]	*yemg2.8*	N	*Dm*
yema gene 3.4 [717]	*yemg3.4*	N	*Dm*
yema gene 3a [717]	*yemg3a*	N	*Dm*
yema gene 3b [717]	*yemg3b*	N	*Dm*
yema gene 3c [717]	*yemg3c*	N	*Dm*
yema gene 4 [717]	*yemg4*	N	*Dm*
yema gene 9.5 [717]	*yemg9.5*	N	*Dm*
yemanuclein α; similar to *ubinuclein* [717]	*yemalpha*	Y	*Dm*
wings down; pourquoi-pas; serendipity-cognate [718]	*pqp; wdn; sry-h1*	Y	*Dm*
serendipity delta; serendipity δ [719]	*sry-delta*	Y	*Dm*
serendipity α [719]	*sry-alpha*	Y	*Dm*
heat shock RNA ω [720]	*hsr-omega*	N	*Dm*
tiovivo; nebbish; kinesin-like protein at 38b [721-723]	*klp38b; tio; neb*	N	*Dm*
GTP-binding protein alpha-subunit; G protein α 73b [724]	*Galpha73b*	N	*Dm*
Guanine nucleotide-binding protein G(I) subunit [426]	*GalphaI*	N	*Dm*
G protein β-subunit 13f; heterotrimeric guanine nucleotide-binding protein beta subunit (Bombyx mori) [725]	*Gbeta13f*	Y	*Dm*
G protein γ 1; CG8261 [726]	*Ggamma1; bro4*	Y	*Dm*
protein tyrosine phosphatase 69d [214]	*ptp69d*	N	*Dm*
similar to *serine/threonine kinase pelle;* homologous to *irak-4* [81, 727-730]	*pll*	Y	*Dm*
gastrulation-defective [81, 730, 731]	*gd*	Y	*Dm*
short gastrulation [730, 732, 733]	*sog*	N	*Dm*
tube [81, 730, 731]	*tub*	Y	*Dm*
similar to *Bombyx mori spätzle 1* [81, 730, 731]	*spz*	Y	*Dm*
weckle [734]	*wek*	N	*Dm*

cactus [4, 663, 735]	*cact*	Y	*Dm*
BzArgOEtase (Bombyx mori); similar to *easter; clip-domain serine protease subfamily B* [81, 727, 730, 731, 736]	*ea*	Y	*Dm*
similar to *snake (Drosophila melanogaster);* similar to *serine protease 21 (Manduca sexta); clip-domain serine protease subfamily c* [81, 727, 730, 731]	*snk*	Y	*Dm*
toll [665, 730, 737-739]	*tl*	N	*Dm*
similar to *Bombyx mori calpain;* weakly similar to *Drosophila melanogaster Calpain-A* [4, 740]	*CalpA*	Y	*Dm*
similar to *brokenheart;* similar to *G protein oalpha 47A; Guanine nucleotide-binding protein G(o) subunit alpha; G protein alpha subunit go* [426]	*G-olpha47A*	Y	*Dm*
concertina; Guanine nucleotide-binding protein subunit alpha-13 [741]	*conc*	N	*Dm*
SNF1A/AMP-activated protein kinase - alpha subunit [742]	*SNF1-AMPK-alpha subunit*	Y	*Dm*
SNF1A/AMP-activated protein kinase - beta subunit [742]	*SNF1-AMPK-beta subunit*	Y	*Dm*
SNF1A/AMP-activated protein kinase - gamma subunit [742]	*SNF1-AMPK-gamma subunit*	Y	*Dm*
IGF-II mRNA-binding protein [92]	*imp; MRE11*	Y	*Dm*
similar to *G protein alpha q; G protein α49b*	*Gαq; Galpha49b*	Y	*Its role in oogenesis not described*
map kinase activated protein-kinase-2 [743]	*mk2; MAPK-ak2*	Y	*Dm*
ptb-associated splicing factor; weakly similar to *Drosophila no on or off transient a* [602, 744]	*psf*	Y	*Dm*
palmitoyl-protein thioesterase 1 [745]	*ppt1*	Y	*Dm*
abl tyrosine kinase [69, 159, 746]	*abl*	Y	*Dm*
Abelson interacting protein [4]	*Abi*	Y	*Dm*
wing blister; homologous to *laminin alpha 2 (merosin)* [747]	*wb*	N	*Dm*
supervillin; CG33232 [748]	*svil*	Y	*Dm*
cyclope; cytochrome c oxidase subunit vic [749]	*cype*	Y	*Dm*
la autoantigen-like [750]	*la*	Y	*Dm*
tramtrack [63]	*ttk; ttk69*	Y	*Dm*
high mobility group protein b1; dorsal switch protein 1 [751-753]	*HMGb1; dsp1; ssrp2*	Y	*Dm*
zinc finger protein 43c [754]	*az2*	N	*Dm*
maverick [755]	*mav*	N	*Dm*
shibire; dynamin [69]	*shi; dyn*	Y	*Dm*
protein o-fucosyltransferase 1; similar to *Bombyx mori fut12 gene* [756]	*pofut1*	Y	*Dm*
protein o-fucosyltransferase 2; similar to *Bombyx mori fut13 gene*	*pofut2*	Y	*Its role in oogenesis not described*
similar to *bloated tubules; sodium/chloride dependent transporter* [757]	*blot*	Y	*Dm*
gastrulation defective protein 1 homolog; CG5543; similar to *WD repeat-containing 70 protein* [4]	CG5543	Y	*Dm*
high mobility group protein 20a	*HMG20a*	Y	*Its role in oogenesis not described*
high mobility group box-containing protein 4; hmg-box protein hmg2l1	*HMGx4*	Y	*Its role in oogenesis not described*
calcium atpase at 60a; sarcoplasmic/endoplasmic reticulum calcium atpase [758]	*serca; kum; dserca; cap60a*	Y	*Dm*

dacapo [4, 759]	*chakra; dap*	N	*Dm*
dead box protein 73d;cyclin-dependent kinase interactor 4; ATP-*dependent RNA helicase ddx51* [760]	*dpb73d*	N	*Dm*
liprin-α [761]	*liprin-a*	N	*Dm*
mitochondrial acyl carrier protein 1; nadh-ubiquinone oxidoreductase acyl carrier protein [762]	*mtacp1*	N	*Dm*
mitochondrial assembly regulatory factor; mitofusin [763]	*marf; mfn; mfn2*	Y	*Dm*
ripped pocket; gonad-specific amiloride-sensitive sodium channel 1 [764]	*rpk; gnac1*	N	*Dm*
kurtz; similar to *beta-arrestin 1* [765]	*krz*	Y	*Dm*
ubiquitin carboxy-terminal hydrolase; CG4265 [766]	*uch*	Y	*Dm*
lark [767]	*lark*	Y	*Dm*
polypeptide n-acetylgalactosaminyltransferase 35a [768]	*pgant35a*	N	*Dm*
semaphorin-5c [769]	*sema-5c*	N	*Dm*
semaphorin 1b [769]	*sema-1b*	N	*Dm*
selenophosphate synthetase 1; selenide, water dikinase [770]	*sps1*	Y	*Dm*
selenophosphate synthetase 2; selenide, water dikinase 2 [770]	*sps2*	N	*Dm*
sodium/potassium exchanging and transporting ATPase subunit beta 1 nervana 1 [4, 771]	*nrv1*	Y	*Dm; Bg*
sodium/potassium exchanging and transporting ATPase subunit beta 2 nervana 2 [771]	*nrv2*	Y	*Bg*
**heat shock proteins (in ovaries and as maternal effects) and their control of protein abundance during oogenesis**			
similar to *heat shock factor a2 (Bombyx mori)* [772]	*hsf-2a*	Y	*Dm*
similar to *heat shock factor b (Bombyx mori)* [772]	*hsfb*	Y	*Dm*
similar to *heat shock factor c (Bombyx mori)* [772]	*hsfc*	Y	*Dm*
heat shock factor binding protein 1-like; CG5446 [4, 773]	*hsfbp1; hsbpsb*	Y	*Dm*
19.5 kDa heat shock protein (Bombyx mori)	*19.5hsp*	Y	*Its role in oogenesis not described*
trap1 ; hsp90-like [774]	*trap1*	Y	*Dm*
(Bombyx mori) heat shock protein 1; similar to *Drosophila lethal (2) essential for life* and *hsp27* [775-777]	*hsp1*	Y	*Dm*
(Bombyx mori small heat shock protein, shsp) - heat shock protein 19.9; similar to *Drosophila lethal (2) essential for life* [775]	*hsp19.9*	Y	*Dm*
(Bombyx mori small heat shock protein, shsp) - heat shock protein 20.1; similar to *Drosophila lethal (2) essential for life* [775]	*hsp20.1*	Y	*Dm*
(Bombyx mori small heat shock protein, shsp) - heat shock protein 20.4; similar to *Drosophila lethal (2) essential for life* [775]	*hsp20.4*	Y	*Dm*
(Bombyx mori small heat shock protein, shsp) - heat shock protein 20.8; similar to *Drosophila lethal (2) essential for life* [775]	*hsp20.8*	Y	*Dm*
(Bombyx mori small heat shock protein, shsp) - heat shock protein 23.7; similar to *Drosophila lethal (2) essential for life* [775]	*hsp23.7*	Y	*Dm*
heat shock protein 21.4	*hsp21.4*	Y	*Its role in*

			oogenesis not described
heat shock cognate protein 70-4; heat shock protein cognate 4 [494, 778, 779]	*hsc70-4; hsc4*	Y	*Dm*
heat shock cognate protein 70; heat shock protein cognate 3 [160, 780]	*hsc70; hsc3; hsc70-3*	Y	*Dm; Bm*
heat shock cognate protein 70cb [4]	*hsc70cb*	Y	*Dm*
heat shock protein cognate 5 [4]	*hsc5*	Y	*Dm*
similar to *Bombyx mori heat shock protein 40 homolog DNAj-1* [781]	*hsp40; DNAj*	Y	*Dm*
heat shock protein 60 [782]	*hsp60*	Y	*Dm*
similar to *heat shock protein 68; heat shock protein 70-like* [160, 772, 781]	*hsp70*	Y	*Dm; Bm*
heat shock protein 83; heat shock protein 90 [494]	*hsp90*	Y	*Dm*
endoplasmin; 94 kDa glucose-regulated protein; similar to *Drosophila glycoprotein 93; heat shock protein 90 kDa beta member 1* [783]	*gp93*	Y	*Dm*
hsc70/hsp90-organisng protein hop [780]	*hop*	Y	*Dm*
CG11267*; heat shock 10kDa protein* [4]	CG11267	Y	*Dm*
CG1416*; activator of 90 kDa heat shock protein ATPase homolog; Bombyx mori bm44* [4]	*bm44*	Y	*Dm*
RNA polymerase II 140kD subunit [772]	*rpII140*	Y	*Dm*
samui [784]	*samui*	Y	*Bm*
**Vitellogenesis, lipid storage, ovarian maturation and hormonal regulation of oogenesis**			
apolipophorin-III [785, 786]	*apoLp-III*	Y	Lepidoptera
apolipophorin precursor; Drosophila CG11064 [787, 788]	*apoLp; apolp1/2*	Y	*Dm;* Lepidoptera
lipophorin receptor [787, 789, 790]	*Lpr1/2*	Y	*Dm;* Lepidoptera
arylphorin (subunit beta); sex-specific storage-protein 2 [791-796]	*hex2; sp2*	Y	Lepidoptera
vitellogenin (protein cleaved into vitellin light chain (vl), vitellin light chain rare isoform, vitellin heavy chain rare isoform and vitellin heavy chain (vh)) [797-799]	*Vg; Vtg*	Y	*Dm;* Various
vitellogenin receptor; yolkless [800-802]	*yl; VgR*	Y	*Dm;* Various
*spherulin-2a (*similar to *Plodia interpunctella yp4)*[803]	*yp4*	Y	*Pi*
chico [801, 804]	*chico; IRS*	Y	*Dm;* Various
Bombyxin genes[805]	*bbxA1; bbxA3*	Y	*Bm*
insulin-like receptor [801, 804]	*InR*	Y	*Dm*
ribosomal protein l10a [806, 807]	*rpl10ab*	Y	*Dm; Fm*
60s ribosomal protein l10; qm protein homolog [806, 807]	*qm*	Y	*Dm; Fm*
string of pearls; ribosomal protein s2 [808, 809]	*sop; rp2*	Y	*Dm;* Various
resistance to juvenile hormone; methoprene-tolerant [401, 402, 810]	*met*	Y	*Dm*
ultraspiracle; rxr type hormone receptor [4, 811-815]	*usp; cf1*	Y	*Dm;* Various
ecdysone receptor [4, 812, 816, 817]	*EcR*	Y	*Dm;* Various
start1 [4, 818]	*start1*	Y	*Dm*
defective in the avoidance of repellents dare; adrenodoxin reductase [819, 820]	*dare*	Y	*Dm*
ecdysone-induced protein 74 [816]	*E74*	N	*Dm*

ecdysone-induced protein 75b (75a,b,c and d) [816, 821]	*E75*	Y	*Dm; Bm*
homologous to *Bombyx mori c-cbl-associated protein (cap) transcript variant a* [822]	*bmcap-a*	Y	*Bm*
follicle specific protein [823]	*fsp-I*	N	*Ms*
similar to *Bombyx mori egg-specific protein (LOC693022)* [824-826]	*ESP*	N	*Bm*
calmodulin [827-829]	*cam*	Y	*Dm; Bg; Of*
calmodulin-binding protein (striatin); weak homology to CG7392 [4, 85]	*striatin*	Y	*Dm*
calmodulin dependent protein kinase [4, 830]	*camk*	Y	*Dm*
hormone receptor 3; Drosophila hormone receptor-like in 46 [370, 826, 831]	*hr3; hr46*	Y	*Dm; Aa; Bm*
hepatocyte nuclear factor 4 isoform a [832]	*hnf-4a*	Y	*Bm*
hepatocyte nuclear factor 4 isoform b [832]	*hnf-4b*	Y	*Bm*
juvenile hormone esterase [833]	*jhe*	N	*Dm*
juvenile hormone esterase binding protein; weak homology to *Drosophila* CG3776 [4, 834]	*JHEbp; DmP29*	Y	*Dm*
juvenile hormone epoxide hydrolase [833]	*JHEH*	Y	*Dm*
homologous to *Bombyx juvenile hormone epoxide hydrolase-like protein 1* [835]	*jheh-lp1*	Y	*Bm*
homologous to *Bombyx juvenile hormone epoxide hydrolase-like protein 3* [835]	*jheh-lp3*	Y	*Bm*
homologous to *Bombyx juvenile hormone epoxide hydrolase-like protein 5* [835]	*jheh-lp5*	Y	*Bm*
juvenile hormone binding protein; homologous to *Drosophila* CG1532 [4]	*JHbp*	Y	*Bm; Dm*
juvenile hormone binding protein (hemolymph) [836, 837]	*hJHbp*	Y	*Ms*
cytosolic juvenile hormone binding protein 36 KDa subunit [836]	*cJHbp*	Y	*Ms*
takeout [819, 838]	*to*	Y	*Dm*
similar to *niemann-pick type c-2; ecdysteroid-regulated 16 kDa protein precursor* [839]	*npc2a; esr16*	Y	*Dm*
ecdysone-induced protein 63e [840]	*Eip63E; cdc2-63E*	N	*Dm*
similar to *sgt1 protein homolog ecdysoneless* [841, 842]	*ecd*	Y	*Dm*
cytochrome p450 (E-class, group I) protein disembodied [843]	*dib; cyp302a1*	N	*Dm*
halfway; singed wings [844]	*hfw; swi*	Y	*Dm*
clathrin light chain [845]	*chc*	Y	*Dm*
clathrin heavy chain [845]	*clc*	Y	*Dm*
ced-6 [846]	*ced-6*	Y	*Dm*
wnt receptor l(2)43Ea boca [4, 847, 848]	*boca*	Y	*Dm*
jagunal [4, 849]	*jagn*	Y	*Dm*
exocyst complex component sec5 [4, 845, 847, 849]	*sec5*	Y	*Dm*
exocyst complex component sec6 [4, 849]	*sec6*	Y	*Dm*
protein phosphatase 2a regulatory subunit b'; widerborst [850, 851]	*wdb; PP2Ab'*	Y	*Dm*
protein phosphatase 2a regulatory subunit b 55kDa; twins [850, 851]	*PP2Ab55kDa*	Y	*Dm*
protein phosphatase 2a regulatory subunit b gamma [850, 851]	*PP2Agamma*	Y	*Dm*
protein phosphatase 2a regulatory subunit a (65 kDa); homologous to *Drosophila protein phosphatase 2a at 29b* [850, 851]	*PP2Aa*	Y	*Dm*

microtubule star; protein phosphatase 2a catalytic subunit c [850, 851]	*mts; PP2Ac*	Y	*Dm*
lipid storage droplet 1; perilipin 1 [852]	*lsd1; plin-1; plin1*	Y	*Dm*
lipid storage droplet 2 [853, 854]	*lsd2*	Y	*Dm*
lipase-1 [855]	*lip-1*	Y	*Dm*
serine/threonine protein kinase akt [819, 851, 856, 857]	*akt; akt1*	Y	*Dm*
liquid facets-related [858]	*lqfr*	Y	*Dm*
liquid facets [4]	*lqf*	Y	*Dm*
garnet [4, 859]	*g*	Y	*Dm*
cationic amino acid transporter; slimfast [3, 4]	*slif*	Y	*Dm*
ornithine decarboxylase [860]	*odc*	Y	*Dm*
ornithine decarboxylase antizyme; gutfeeling [4, 861]	*guf; Oda; az*	Y	*Dm*
**General growth regulators (including the hippo pathway)**			
serine/threonine kinase 3-like (hippo; STE20)[4, 862-865]	*hpo*	Y	*Dm*
salvador [4, 863]	*sav*	Y	*Dm*
warts [863]	*wts*	Y	*Dm*
mob as tumor suppressor [4, 863]	*mats; mob1*	N	*Dm*
mob-2	*mob2*	Y	*Its role in oogenesis not described*
preimplantation protein; mps one binder kinase activator-like 4 [4]	*mob4-like*	Y	*Dm*
hindsight; pebbled [863]	*hnt*	Y	*Dm*
expanded [862, 863]	*ex*	Y	*Dm*
merlin [862, 863]	*mer; ERM2*	N	*Dm*
kibra; CG33967 [862, 864]	*kibra*	Y	*Dm*
yorkie; yap65-like protein [865]	*yki*	Y	*Dm*
phosphatidylinositol 4-kinase alpha [864]	*PI4kIIIalpha*	Y	*Dm*
bitesize; synaptotagmin-like [4, 866]	*btsz*	Y	*Dm*
par-domain protein 1; CG17888 [4, 211, 867]	*pdp1*	Y	*Dm*
**Control of growth - programmed cell death regulation (including of pole cells) – a autophagy - response to starvation**			
p53 [4, 250, 868]	*p53*	Y	*Dm; Bm*
p35 [250, 869]	*p35*	N	*Dm*
death executioner Bcl-2 homologue [4, 869, 870]	*debcl*	N	*Dm*
homologous to *bruce and Bombyx bir-superfamily domain protein - survivin-1* [4, 871]	*bruce; survivin-1*	Y	*Dm*
bir-superfamily domain protein - inhibitor of apoptosis 1; thread [4, 872, 873]	*iap1; th; diap1*	Y	*Dm*
bir-superfamily domain protein - inhibitor of apoptosis 2 [872]	*iap2; diap2*	Y	*Dm*
ubiquitin conjugation enzyme E2; bendless [4, 642]	*ubc13; ben*	Y	*Dm*
b-cell lymphoma protein 2 (bcl-2) protein - buffy [874]	*buffy*	Y	*Dm*
autophagy-specific gene 1; serine/threonine-protein kinase unc-51 [4, 872, 874]	*atg1*	Y	*Dm*
autophagy-specific gene 2 [874]	*atg2*	Y	*Dm*
autophagy-specific gene 3 [4]	*atg3; aut1*	Y	*Dm*

autophagy-specific gene 4 [4]	*atg4*	Y	*Dm*
autophagy-specific gene 5 [4, 872]	*atg5*	Y	*Dm*
autophagy-specific gene 6; beclin-1 [870]	*atg6*	Y	*Dm*
autophagy-specific gene 7 [4, 870, 875]	*atg7*	Y	*Dm*
autophagy-specific gene 8 [872, 875]	*atg8*	Y	*Dm*
autophagy-specific gene 12 [874]	*atg12*	Y	*Dm*
autophagy-specific gene 13 [872]	*atg13*	N	*Dm*
phosphotidylinositol 3 kinase 59f [872]	*pi3k59f; vps34*	Y	*Dm*
cell death activator-b [876]	*cide-b*	Y	*Its role in oogenesis not described*
cell cycle and apoptosis regulatory protein 1	*ccar1*	Y	*Its role in oogenesis not described*
longitudinals-lacking [4, 877]	*lola*	Y	*Dm*
translationally controlled tumour protein [806, 878]	*tctp*	Y	*Fm*
apoptosis linked protein 2 [4]	*alg-2*	Y	*Dm*
quaking related 54b; sam50 [4, 879]	*qkr; sam50*	Y	*Dm*
held out wings [4, 879]	*how*	Y	*Dm*
spinster [880]	*spin*	Y	*Dm*
death executioner caspase related to apopain/yama; decay; caspase 3 [881]	*decay*	N	*Dm*
death caspase 1 [870, 874, 882]	*dcp-1*	N	*Dm*
death related ced-3/nedd2-like protein; dredd/dcp-2 [883]	*dredd*	Y	*Dm*
*ice; drice; caspase-1 (*in *Bombyx mori)* [884]	*ice*	Y	*Dm*
dronc; nedd2-like caspase [885]	*dronc; nc*	Y	*Dm*
dynamin related protein 1 [870, 886]	*drp1*	Y	*Dm*
similar to *optic atrophy 1-like* [870]	*opa1-like*	Y	*Dm*
resistance to juvenile hormone; methoprene-tolerant [4, 810]	*met*	Y	*Dm*
deterin [887]	*det*	N	*Dm*
tao-1 [4, 888]	*tao-1*	Y	*Dm*
melted [4, 889]	*melt*	N	*Dm*
midway [890]	*mdy*	N	*Dm*
pita [891]	*pita*	Y	*Dm*
plenty of sh3s [819]	*posh*	N	*Dm*
phosphoinositide-dependent kinase 1 dstpk61 [4, 819]	*dstpk61*	Y	*Dm*
dream [4, 819]	*strica; dream*	N	*Dm*
target of rapamycin [819]	*tor*	Y	*Dm*
thor [819, 889]	*thor*	N	*Dm*
death associated molecule related to mch2; daydream [892]	*damm*	N	*Dm*
ecdysone-induced protein 28/29kD; methionine-s-sulfoxide reductase [893]	*Eip28/29; Eip71CD*	Y	*Dm*
modifier of rpr and grim, ubiquitously expressed; weak homology to *ubiquitin-conjugating enzyme E2 D4* [4, 894]	*morgue*	N	*Dm*
**Response to starvation - cytoplasmic SMN protein-containing granules snRNP bodies (U bodies)**			
survival motor neuron protein; CG17454 [895]	*smn*	Y	*Dm*

**Immune defense**			
hemolin; p4 [896]	*p4*	Y	*Hc*
hemolin interacting protein; yippee [3, 897]	*yip*	Y	*Hc; Dm*
yippee interacting protein 2 [3]	*yip2*	Y	*Dm*
cecropin A [896]	*cecA*	Y	*Hc*
weak homology to *cecropin B*	*cecB*	Y	*Its role in oogenesis not described*
homology to *Bombyx serpin-1* and *Drosophila spn4/42Da* [4, 898]	*srp1; spn4/42Da*	Y	*Dm*
homology to *Bombyx serpin-2* and *Drosophila spn4/42Da* [4, 898]	*srp2; spn4/42Da*	Y	*Dm*
homology to *Bombyx serpin-3* and *Drosophila spn27A* [4, 663, 898]	*srp3; spn27A*	Y	*Dm*
homology to *Bombyx serpin-4* and *Drosophila spn28D* [898]	*srp4; spn28D*	Y	*Its role in oogenesis not described*
homology to *Bombyx serpin-5* and *Drosophila spn77Ba* [898]	*srp5; spn77Ba*	Y	*Its role in oogenesis not described*
homology to *Bombyx serpin-6* and *Drosophila spn88Ea* [4, 898]	*srp6; spn88Ea*	Y	*Dm*
homology to *Bombyx serpin-10* and *Drosophila spn100a* [898]	*srp10; spn100A*	Y	*Its role in oogenesis not described*
homology to *Bombyx serpin-11* and *Drosophila spn100A* [898]	*srp11; spn100A*	Y	*Its role in oogenesis not described*
homology to *Bombyx serpin-13* and *Drosophila spn28d* [898]	*srp13; spn28D*	Y	*Its role in oogenesis not described*
MAPKK4 [4, 184]	*mkk4; MAPKK4*	Y	*Dm*
similar to *Bombyx mori clip domain serine protease 4;* similar to *manduca sexta hemolymph proteinase 17* [727]	*bmclip4*	Y	*Its role in oogenesis not described*
similar to *Bombyx mori clip domain serine protease 11;* similar to *manduca sexta serine proteinase-like protein 1* [727]	*bmclip11*	Y	*Its role in oogenesis not described*
**Subgrouping - transfer molecules**			
transferrin [899]	*tf; tsf*	Y	*Sp*
Ferritin 2 – light chain homolog [4, 899, 900]	*FER2-LCH*	Y	*Dm; Sp; At*
Ferritin 1/3 – heavy chain homolog [4, 160, 899, 900]	*FER1/3-HCH*	Y	*Dm; Sp; At; Bm*
FK506-binding protein 2; FK506-binding protein 12 (in Bombyx mori) [4, 901]	*FKBP12*	Y	*Dm*
FK506-binding protein 1 [4, 902]	*FKBP39*	Y	*Dm*
weakly similar to *refractory to sigma p* [4, 903]	*ref(2)p*	Y	*Dm*
similar to *bmrelish1 and bmrelish2; nuclear factor nf-kappa-b p110 subunit isoform 1 or 2;* weakly similar to *Drosophila melanogaster relish* [4, 904]	*rel*	Y	*Dm*
hemomucin [4, 905]	*rrm5; hmu*	Y	*Dm*
smt3 activating enzyme 2 [4, 689]	*sae2; sip2; uba2*	Y	*Dm*
galactin; galactose specific c-type lectin [906]	*lectin-galc1*	N	*Dm*
***Wolbachia* infection detected in *Pararge aegeria* ovaries** [900, 907, 908]			

**Circadian (related) genes with ovarian functions - necessary for oocyte maturation**			
clock [909, 910]	*clk*	N	*Dm*
period [909-911]	*per*	Y	*Dm*
timeless [909-911]	*tim*	Y	*Dm*
diapause bioclock protein; time interval measuring enzyme-esterase a4 [912]	*time-ea4*	Y	*Bm*
open rectifier potassium (k+) channel 1 [913]	*ork1*	N	*Dm*
(6-4)-photolyase; cryptochrome [914]	*phr6-4*	Y	*Dm*
**Maternal effect genes to facilitate yolk consumption by the embryos**			
cathepsin l-like cysteine protease; Bombyx cysteine protease; cysteine proteinase-1 [915-917]	*bcp; cl; cp1*	Y	*Bm; Dm;* Various
cathepsin b; cathepsin b-like cysteine proteinase [918]	*catb*	Y	*Md*
cathepsin d; aspartic protease [919, 920]	*catd*	Y	*Rp*
cathepsin f-like cysteine protease; CG12163 [4]	*catf*	Y	*Dm*
ecdysteroid-phosphate phosphatase [921]	*EPPase*	Y	*Bm*
vacuolar proton atpase; vacuolar h+ atpase subunit 100-1 [922, 923]	*mva; v100; vha100-1*	Y	Various
vacuolar proton atpase; vacuolar h+ atpase subunit 100-2 [922, 923]	*vha100-2*	Y	Various
h+ transporting atpase v0 subunit d; vacuolar h+ atpase subunit ac39-1 [922, 923]	*vhaac39-1*	Y	Various
vacuolar atp synthase subunit d; vacuolar h+ atpase subunit 36-1 [922, 923]	*mvd; vha36-1*	Y	Various
CG7899; *acid phosphatase 1* [918, 924, 925]	*acph-1; ap*	N	*Dm; Md; Rp*
primo-1; acid phosphatase isoenzyme [918, 924]	*primo-1*	Y	*Md; Rp*
**Eggshell formation - vitelline membrane formation and choriogenesis**			
proto-oncogene tyrosine-protein kinase Ret [711, 926, 927]	*Ret*	N	*Dm*
weak homology to *Bombyx mori vitelline membrane associated protein p30* [928]	*VMP30*	Y	*Bm*
Bombyx mori vitelline membrane protein 90 [929]	*VMP90*	N	*Bm*
vitelline membrane 32e [930, 931]	*VM32e; VMP32e*	N	*Dm*
vitelline membrane 26a [931-934]	*VM26a*	N	*Dm*
vitelline membrane 26b [931, 932]	*VM26b*	N	*Dm*
vitelline membrane 26ac [932]	*VM26Ac; tu-3*	N	*Dm*
vitelline membrane 34ca [931, 933]	*VM34c*	N	*Dm*
femcoat [935, 936]	*femcoat*	N	*Dm*
follicle cell protein 26Aa; palisade [937]	*psd; fcp26Aa; tu-1*	N	*Dm*
cad99c [937, 938]	*cad99c; ca-10*	Y	*Dm*
crinkled; myosin-VIIa [939]	*ck; myoVIIa*	Y	*Dm*
vitelline membrane like [940]	*vml*	N	*Dm*
high mobility group protein a [941]	*HMGa*	Y	*Bm*
egg protein 80 [942]	*EP80*	Y	*Bm*
follicle cell protein 3c [934]	*fcp3c*	Y	*Dm*
chromodomain helicase DNA binding protein 1 [943]	*chd1*	Y	*Bm*
chorion peroxidase; peroxinectin-related protein [944]	*pxt*	Y	*Dm*

gataβ; transcription factor BCFI [826]	*GATAβ*	Y	*Bm*
chorion transcription factor cf2 [815, 945, 946]	*cf2*	Y	*Dm*
chorion b-ZIP transcription factor [376]	*CbZ*	Y	*Bm*
chorion protein 15 (Drosophila melanogaster); CG6519 [889, 931, 947]	*cp15; s15*	N	*Dm*
chorion protein 16 (Drosophila melanogaster); CG6533 [889, 931, 947]	*cp16; s16*	N	*Dm*
chorion protein 18 (Drosophila melanogaster); CG6517 [889, 931, 947]	*cp18; s18*	N	*Dm*
chorion protein 19 (Drosophila melanogaster); CG6524 [889, 947]	*cp19; s19*	N	*Dm*
chorion protein 36 (Drosophila melanogaster); CG1478 [889, 931, 948, 949]	*cp36; s36*	N	*Dm*
chorion protein 38 (Drosophila melanogaster); CG11213 [889, 931, 947, 948]	*cp38; s38*	N	*Dm*
chorion protein a at 7f (Drosophila melanogaster); CG33962 [947]	*cp7fa*	N	*Dm*
chorion protein b at 7f (Drosophila melanogaster); CG15350 [947]	*cp7fb*	N	*Dm*
chorion protein c at 7f (Drosophila melanogaster); CG15351 [947]	*cp7fc*	N	*Dm*
defective chorion 1 [950-952]	*dec1*	N	*Dm*
Lepidopteran *chorion* genes [953-955]	*chorion genes*	Y	Lepidoptera
**Egg activation, ovulation, gene regulation in oviduct upon mating and maternal effect genes involved in fertilisation**			
CG12251*; aquaporin* [956, 957]	*aqp*	N	*Bg; Dm*
CG7777*;* similar to *Bombyx mori aquaporin* [4, 925]	CG7777	N	*Dm*
fs(1)m19; wispy; similar to *poly(a) polymerase cid (pap) (caffein-induced death protein)* [4, 958]	*wisp*	Y	*Dm*
paramyosin [133]	*prm*	Y	*Dm*
hir histone cell cycle regulation defective; hira; sesame [604, 959]	*ssm; hira; dhh*	N	*Dm*
DNA polymerase α 73kD [604]	*DNApol-α73*	N	*Dm*
centromere identifier [604]	*cid*	N	*Dm*
kinesin-like protein at 3A; similar to *chromosome-associated kinesin kif4A* [783]	*klp3A*	Y	*Dm*
CG17838*; Syncrip;* similar to *heterogeneous nuclear ribonucleoprotein Q* [211]	*syp*	Y	*Dm*
octopamine receptor in mushroom bodies; G-protein-coupled receptor for octopamine [960, 961]	*oamb*	Y	*Dm*
tyramine β hydroxylase [962]	*tbh*	N	*Dm*
yin; opt1; oligopeptide transporter [963, 964]	*yin; opt1*	Y	*Dm*
**Additional genes - not clear what their function might be, but play roles as maternal effects and egg production**			
ectoderm-expressed 4; sarm1 [4]	*ect4; sarm1*	Y	*Dm*
k3	*k3*	Y	*Its role in oogenesis not described*
Bombyx mori ovary specific non-coding RNA [160]		N	*Bm*
lots wife; drop dead; CG5652 [965]	*lwf*	Y	*Dm*
protein tyrosine phosphatase prl [966]	*prl-1*	Y	*Dm*

kruppel-homolog [967, 968]	*krh1*	Y	*Dm; Aedes sp.*
minibrain	*mnb*	Y	*Its role in oogenesis not described*
elav [969]	*elav*	Y	*Its role in oogenesis not described*

References Table

1. **FlyBase** [http://www.flybase.org]
2. **SilkBase** [http://silkbase.ab.a.u-tokyo.ac.jp]
3. Gelbart WM, Emmert DB: **FlyBase high throughput expression pattern data Beta Version.** 2010.
4. **BDGP insitu homepage** [http://insitu.fruitfly.org/cgi-bin/ex/insitu.pl]
5. Song X, Xie T: ***wingless* signaling regulates the maintenance of ovarian somatic stem cells in *Drosophila*.** *Development* 2003, **130:**3259-3268.
6. Deng W, Lin H: **Asymmetric germ cell division and oocyte determination during *Drosophila* oogenesis.** *Int Rev Cytol* 2001, **203:**93-138.
7. Theisen H, Purcell J, Bennett M, Kansagara D, Syed A, Marsh JL: ***dishevelled* is required during *wingless* signaling to establish both cell polarity and cell identity.** *Development* 1994, **120:**347-360.
8. Jordan KC, Hatfield SD, Tworoger M, Ward EJ, Fischer KA, Bowers S, Ruohola-Baker H: **Genome wide analysis of transcript levels after perturbation of the EGFR pathway in the *Drosophila* ovary.** *Dev Dyn* 2005, **232:**709-724.
9. Thompson BJ: **A complex of Armadillo, Legless, and Pygopus coactivates dTCF to activate wingless target genes.** *Curr Biol* 2004, **14:**458-466.
10. Banziger C, Soldini D, Schuett C, Zipperlen P, Hausmann G, Basler K: **Wntless, a conserved membrane protein dedicated to the secretion of Wnt proteins from signaling cells.** *Cell* 2006, **125:**509-522.
11. Forbes AJ, Spradling AC, Ingham PW, Lin H: **The role of segment polarity genes during early oogenesis in Drosophila.** *Development* 1996, **122:**3283-3294.

12. Forbes AJ, Lin H, Ingham PW, Spradling AC: ***hedgehog* is required for the proliferation and specification of ovarian somatic cells prior to egg chamber formation in *Drosophila*.** *Development* 1996, **122:**1125-1135.

13. Zhang Y, Kalderon D: **Regulation of cell proliferation and patterning in *Drosophila* oogenesis by Hedgehog signaling.** *Development* 2000, **127:**2165-2176.

14. Zhang Y, Kalderon D: **Hedgehog acts as a somatic stem cell factor in the *Drosophila* ovary.** vol. 410. pp. 599-604; 2001:599-604.

15. Gorfinkiel N, Sierra J, Callejo A, Ibanez C, Guerrero I: **The *Drosophila* ortholog of the human Wnt inhibitor factor *Shifted* controls the diffusion of lipid-modified Hedgehog.** *Dev Cell* 2005, **8:**241-253.

16. Chamoun Z, Mann RK, Nellen D, von Kessler DP, Bellotto M, Beachy PA, Basler K: **Skinny hedgehog, an acyltransferase required for palmitoylation and activity of the hedgehog signal.** *Science* 2001, **293:**2080-2084.

17. Kent D, Bush EW, Hooper JE: **Roadkill attenuates Hedgehog responses through degradation of Cubitus interruptus.** *Development* 2006, **133:**2001-2010.

18. Peel AD, Averof M: **Early asymmetries in maternal transcript distribution associated with a cortical microtubule network and a polar body in the beetle *Tribolium castaneum*.** *Dev Dyn* 2010, **239:**2875-2887.

19. Cohen ED, Mariol MC, Wallace RMH, Weyers J, Kamberov YG, Pradel J, Wilder EL: **DWnt4 regulates cell movement and focal adhesion kinase during *Drosophila* ovarian morphogenesis.** *Dev Cell* 2002, **2:**437-448.

20. Forstemann K, Tomari Y, Du T, Vagin VV, Denli AM, Bratu DP, Klattenhoff C, Theurkauf WE, Zamore PD: **Normal microRNA maturation and germ-line stem cell maintenance requires Loquacious, a double-stranded RNA-binding domain protein.** *PLoS Biol* 2005, **3:**e236.

21. Tanaka ED, Piulachs M-D: **Dicer-1 is a key enzyme in the regulation of oogenesis in panoistic ovaries.** *Biol Cell* 2012, **104:**452-461.

22. Iovino N, Pane A, Gaul U: ***miR-184* has multiple roles in *Drosophila* female germline development.** *Dev Cell* 2009, **17:**123-133.

23. Ohlmeyer JT, Schüpbach T: **Encore facilitates SCF-Ubiquitin-proteasome-dependent proteolysis during *Drosophila* oogenesis.** *Development* 2003, **130:**6339-6349.

24. Lilly MA, de Cuevas M, Spradling AC: **Cyclin A associates with the fusome during germline Cyst formation in the *Drosophila* ovary.** *Dev Biol* 2000, **218:**53-63.

25. Chen D, Wang Q, Huang H, Xia L, Jiang X, Kan L, Sun Q, Chen D: **Effete-mediated degradation of Cyclin A is essential for the maintenance of germline stem cells in *Drosophila*.** *Development* 2009, **136:**4133-4142.

26. King FJ, Lin H: **Somatic signaling mediated by *fs(1)Yb* is essential for germline stem cell maintenance during *Drosophila* oogenesis.** *Development* 1999, **126:**1833-1844.

27. Handler D, Olivieri D, Novatchkova M, Gruber FS, Meixner K, Mechtler K, Stark A, Sachidanandam R, Brennecke J: **A systematic analysis of *Drosophila* TUDOR domain-containing proteins identifies Vreteno and the Tdrd12 family as essential primary piRNA pathway factors.** *EMBO J* 2011, **30:**3977-3993.

28. Besse F, Busson D, Pret AM: **Fused-dependent Hedgehog signal transduction is required for somatic cell differentiation during *Drosophila* egg chamber formation.** *Development* 2002, **129:**4111-4124.

29. Markesich DC, Gajewski KM, Nazimiec ME, Beckingham K: ***bicaudal* encodes the *Drosophila* NAC homolog, a component of the ribosomal translational machinery.** *Development* 2000, **127:**559.

30. Jiang X, Xia L, Chen D, Yang Y, Huang H, Yang L, Zhao Q, Shen L, Wang J, Chen D: **Otefin, a nuclear membrane protein, determines the fate of germline stem cells in *Drosophila* via interaction with Smad complexes.** *Dev Cell* 2008, **14:**494-506.

31. Ashery-Padan R, Ulitzur N, Arbel A, Goldberg M, Weiss AM, Maus N, Fisher PA, Gruenbaum Y: **Localization and posttranslational modifications of otefin, a protein required for vesicle attachment to chromatin, during *Drosophila melanogaster* development.** *Mol Cell Biol* 1997, **17:**4114-4123.

32. Malone CD, Brennecke J, Dus M, Stark A, McCombie WR, Sachidanandam R, Hannon GJ: **Specialized piRNA pathways act in germline and somatic tissues of the *Drosophila* ovary.** *Cell* 2009, **137:**522-535.

33. Vourekas A, Kirino Y, Mourelatos Z: **Elective affinities: a Tudor-Aubergine tale of germline partnership.** *Genes Dev* 2010, **24:**1963-1966.

34. Xi R, Doan C, Liu D, Xie T: **Pelota controls self-renewal of germline stem cells by repressing a Bam-independent differentiation pathway.** *Development* 2005, **132:**5365-5374.

35. Murata Y, Wharton RP: **Binding of pumilio to maternal *hunchback* mRNA is required for posterior patterning in *Drosophila* embryos.** *Cell* 1995, **80:**747-756.

36. Weidmann CA, Goldstrohm AC: ***Drosophila* Pumilio protein contains multiple autonomous repression domains that regulate mRNAs independently of Nanos and Brain Tumor.** *Mol Cell Biol* 2012, **32:**527-540.

37. Parisi M, Lin H: **The *Drosophila* pumilio gene encodes two functional protein isoforms that play multiple roles in germline development, gonadogenesis, oogenesis and embryogenesis.** *Genetics* 1999, **153:**235-250.

38. Lin H, Spradling AC: **A novel group of *pumilio* mutations affects the asymmetric division of germline stem cells in the *Drosophila* ovary.** *Development* 1997, **124:**2463-2476.

39. Maleszka R, Hanes SD, Hackett RL, de Couet HG, Miklos GL: **The *Drosophila melanogaster dodo* (*dod*) gene, conserved in humans, is functionally interchangeable with the *ESS1* cell division gene of *Saccharomyces cerevisiae*.** *Proceedings of the National Academy of Sciences* 1996, **93:**447-451.

40. Gollin SM, King RC: **Studies of f*s(1)1621*, a mutation producing ovarian tumors in *Drosophila melanogaster*.** *Dev Genet* 1981, **2:**203-218.

41. Couderc JL, Godt D, Zollman S, Chen J, Li M, Tiong S, Cramton SE, Sahut-Barnola I, Laski FA: **The *bric a brac* locus consists of two paralogous genes encoding BTB/POZ domain proteins and acts as a homeotic and morphogenetic regulator of imaginal development in *Drosophila*.** *Development* 2002, **129:**2419-2433.

42. Munn K, Steward R: **The *shut-down* gene of *Drosophila melanogaster* encodes a novel FK506-binding protein essential for the formation of germline cysts during oogenesis.** *Genetics* 2000, **156:**245-256.

43. Tirronen M, Partanen M, Heino TO, Heino TI, Roos C: **Analyses of the *Drosophila quit, ovarian tumor* and *shut down* mutants in oocyte differentiation using in situ hybridisation.** *Mech Dev* 1993, **40:**113-126.

44. Nakao H: **Isolation and characterization of a *Bombyx vasa*-like gene.** *Dev Genes Evol* 1999, **209:**312-316.

45. Nakao H, Hatakeyama M, Lee JM, Shimoda M, Kanda T: **Expression pattern of *Bombyx* vasa-like (BmVLG) protein and its implications in germ cell development.** *Dev Genes Evol* 2006, **216:**94-99.

46. Schüpbach T, Wieschaus E: **Maternal-effect mutations altering the anterior-posterior pattern of the *Drosophila* embryo.** *Roux's Arch Dev Biol* 1986, **195:**302-317.

47. Kugler JM, Woo JS, Oh BH, Lasko P: **Regulation of *Drosophila* Vasa *In Vivo* through Paralogous Cullin-RING E3 Ligase Specificity Receptors.** *Mol Cell Biol* 2010, **30:**1769-1782.

48. Spradling A, Fuller MT, Braun RE, Yoshida S: **Germline Stem Cells.** *Cold Spring Harb Perspect Biol* 2011, **3**.

49. McKearin DM, Spradling AC: ***bag-of-marbles*: a *Drosophila* gene required to initiate both male and female gametogenesis.** *Genes Dev* 1990, **4:**2242-2251.

50. Perinthottathil S, Kim C: **Chapter Nineteen - Bam and Bgcn in *Drosophila* germline stem cell differentiation.** In *Vitamins and Hormones. Volume* Volume 87. Edited by Gerald L: Academic Press; 2011: 399-416

51. Lavoie CA, Ohlstein B, McKearin DM: **Localization and function of bam protein require the *benign gonial cell neoplasm* gene product.** *Dev Biol* 1999, **212:**405-413.

52. Gancz D, Lengil T, Gilboa L: **Coordinated regulation of niche and stem cell precursors by hormonal signaling.** *PLoS Biol* 2011, **9:**e1001202.

53. Neumuller RA, Betschinger J, Fischer A, Bushati N, Poernbacher I, Mechtler K, Cohen SM, Knoblich JA: **Mei-P26 regulates microRNAs and cell growth in the *Drosophila* ovarian stem cell lineage.** *Nature* 2008, **454:**241-245.

54. Mukai M, Kitadate Y, Arita K, Shigenobu S, Kobayashi S: **Expression of meiotic genes in the germline progenitors of *Drosophila* embryos.** *Gene Expr Patterns* 2006, **6:**256.

55. Li Y, Maines JZ, Tastan OY, McKearin DM, Buszczak M: **Mei-P26 regulates the maintenance of ovarian germline stem cells by promoting BMP signaling.** *Development* 2012, **139:**1547-1556.

56. Ohlstein B, Lavoie CA, Vef O, Gateff E, McKearin DM: **The *Drosophila* cystoblast differentiation factor, *benign gonial cell neoplasm*, is related to DExH-box proteins and interacts genetically with *bag-of-marbles*.** *Genetics* 2000, **155:**1809-1819.

57. Xie T, Spradling AC: ***decapentaplegic* is essential for the maintenance and division of germline stem cells in the *Drosophila* ovary.** *Cell* 1998, **94:**251-260.

58. Casanueva MO, Ferguson EL: **Germline stem cell number in the *Drosophila* ovary is regulated by redundant mechanisms that control Dpp signaling.** *Development* 2004, **131:**1881-1890.

59. Kawase E, Wong MD, Ding BC, Xie T: **Gbb/Bmp signaling is essential for maintaining germline stem cells and for repressing *bam* transcription in the *Drosophila* testis.** *Development* 2004, **131:**1365-1375.

60. Niepielko MG, Hernaiz-Hernandez Y, Yakoby N: **BMP signaling dynamics in the follicle cells of multiple *Drosophila* species.** *Dev Biol* 2011, **354:**151-159.

61. Evans TA, Haridas H, Duffy JB: **Kekkon5 is an extracellular regulator of BMP signaling.** *Dev Biol* 2009, **326:**36-46.

62. Gilboa L, Lehmann R: **Soma-germline interactions coordinate homeostasis and growth in the *Drosophila* gonad.** *Nature* 2006, **443:**97-100.

63. Yatsu J, Hayashi M, Mukai M, Arita K, Shigenobu S, Kobayashi S: **Maternal RNAs encoding transcription factors for germline-specific gene expression in *Drosophila* embryos.** *Int J Dev Biol* 2008, **52:**913-923.

64. Bartoszewski S, Luschnig S, Desjeux I, Grosshans J, Nüsslein-Volhard C: ***Drosophila* p24 homologues *eclair* and *baiser* are necessary for the activity of the maternally expressed Tkv receptor during early embryogenesis.** *Mech Dev* 2004, **121:**1259-1273.

65. Song XQ, Wong MD, Kawase E, Xi RW, Ding BC, McCarthy JJ, Xie T: **Bmp signals from niche cells directly repress transcription of a differentiation-promoting gene, *bag of marbles*, in germline stem cells in the *Drosophila* ovary.** *Development* 2004, **131:**1353-1364.

66. Liu Z, Matsuoka S, Enoki A, Yamamoto T, Furukawa K, Yamasaki Y, Nishida Y, Sugiyama S: **Negative modulation of bone morphogenetic protein signaling by Dullard during wing vein formation in *Drosophila*.** *Dev Growth Differ* 2011, **53:**822-841.

67. Xie T, Spradling AC: **A niche maintaining germ line stem cells in the *Drosophila* ovary.** *Science* 2000, **290:**328-330.

68. Muzzopappa M, Wappner P: **Multiple roles of the F-box protein Slimb in *Drosophila* egg chamber development.** *Development* 2005, **132:**2561-2571.

69. Kimchie Z, Segev O, Lev Z: **Maternal and embryonic transcripts of *Drosophila* proto-oncogenes are expressed in Schneider 2 culture cells but not in *l(2)gl* transformed neuroblasts.** *Cell Diff Dev* 1989, **26:**79-86.

70. Ryner LC, Goodwin SF, Castrillon DH, Anand A, Villella A, Baker BS, Hall JC, Taylor BJ, Wasserman SA: **Control of male sexual behavior and sexual orientation in *Drosophila* by the *fruitless* gene.** *Cell* 1996, **87:**1079-1089.

71. Casper A, Van Doren M: **The control of sexual identity in the *Drosophila* germline.** *Development* 2006, **133:**2783-2791.

72. Casper AL, Van Doren M: **The establishment of sexual identity in the *Drosophila* germline.** *Development* 2009, **136:**3821-3830.

73. Pauli D, Oliver B, Mahowald AP: **The role of the ovarian tumor locus in *Drosophila melanogaster* germ line sex determination.** *Development* 1993, **119:**123-134.

74. Chau J, Kulnane LS, Salz HK: ***Sex-lethal* enables germline stem cell differentiation by down-regulating Nanos protein levels during *Drosophila* oogenesis.** *Proceedings of the National Academy of Sciences* 2012, **109:**9465-9470.

75. Penalva LOF, Ruiz MF, Ortega A, Granadino B, Vicente L, Segarra C, Valcarcel J, Sanchez L: **The *Drosophila fl(2)d* gene, required for female-specific splicing of *Sxl* and *tra* pre-mRNAs, encodes a novel nuclear protein with a HQ-rich domain.** *Genetics* 2000, **155:**129-139.

76. Oliver B, Pauli D: **Suppression of distinct ovo phenotypes in the *Drosophila* female germline by maleless− and Sex-lethalM.** *Dev Genet* 1998, **23:**335-346.

77. Bai J, Montell D: **Eyes absent, a key repressor of polar cell fate during *Drosophila* oogenesis.** *Development* 2002, **129:**5377-5388.

78. Reeve S, Carhan A, Dee CT, Moffat KG: ***slowmo* is required for *Drosophila* germline proliferation.** *Genesis* 2007, **45:**66-75.

79. Riparbelli MG, Massarelli C, Robbins LG, Callaini G: **The abnormal spindle protein is required for germ cell mitosis and oocyte differentiation during *Drosophila* oogenesis.** *Exp Cell Res* 2004, **298:**96.

80. Dubin-Bar D, Bitan A, Bakhrat A, Amsalem S, Abdu U: ***Drosophila javelin-like* encodes a novel microtubule-associated protein and is required for mRNA localization during oogenesis.** *Development* 2011, **138:**4661-4671.

81. Anderson KV, Nüsslein-Volhard C: **Information for the dorsal-ventral pattern of the *Drosophila* embryo is stored as maternal mRNA.** *Nature* 1984, **311:**223.

82. Moon W, Hazelrigg T: **The *Drosophila* microtubule-associated protein Mini Spindles is required for cytoplasmic microtubules in oogenesis.** *Curr Biol* 2004, **14:**1957.

83. Sato K, Nishida KM, Shibuya A, Siomi MC, Siomi H: **Maelstrom coordinates microtubule organization during *Drosophila* oogenesis through interaction with components of the MTOC.** *Genes Dev* 2011, **25:**2361-2373.

84. Jackson SM, Berg CA: **An A-kinase anchoring protein is required for Protein kinase A regulatory subunit localization and morphology of actin

structures during oogenesis in *Drosophila*. *Development* 2002, **129:**4423-4433.

85. Perrimon N, Lanjuin A, Arnold C, Noll E: **Zygotic lethal mutations with maternal effect phenotypes in *Drosophila melanogaster*. II. Loci on the second and third chromosomes identified by *P*-element-induced mutations.** *Genetics* 1996, **144:**1681-1692.
86. Murphy AM, Montell DJ: **Cell type-specific roles for Cdc42, Rac, and RhoL in *Drosophila* oogenesis.** *J Cell Biol* 1996, **133:**617-630.
87. Genova JL, Jong S, Camp JT, Fehon RG: **Functional analysis of *Cdc42* in actin filament assembly, epithelial morphogenesis, and cell signaling during *Drosophila* development.** *Dev Biol* 2000, **221:**181-194.
88. Vlachos S, Harden N: **Genetic evidence for antagonism between Pak protein kinase and Rho1 Small GTPase signaling in regulation of the actin cytoskeleton during *Drosophila* oogenesis.** *Genetics* 2011, **187:**501-512.
89. Bianco A, Poukkula M, Cliffe A, Mathieu J, Luque CM, Fulga TA, Rørth P: **Two distinct modes of guidance signalling during collective migration of border cells.** *Nature* 2007, **448:**362-365.
90. Dobie KW, Kennedy CD, Velasco VM, McGrath TL, Weko J, Patterson RW, Karpen GH: **Identification of chromosome inheritance modifiers in *Drosophila melanogaster*.** *Genetics* 2001, **157:**1623-1637.
91. Kai T, Williams D, Spradling AC: **The expression profile of purified *Drosophila* germline stem cells.** *Dev Biol* 2005, **283:**486-502.
92. Lecuyer E, Yoshida H, Parthasarathy N, Alm C, Babak T, Cerovina T, Hughes TR, Tomancak P, Krause HM: **Global analysis of mRNA localization**

reveals a prominent role in organizing cellular architecture and function. *Cell* 2007, **131:**174-187.

93. Verheyen EM, Cooley L: **Profilin mutations disrupt multiple actin-dependent processes during *Drosophila* development.** *Development* 1994, **120:**717-728.

94. Sweeney SJ, Campbell P, Bosco G: **Drosophila *sticky/citron kinase* is a regulator of cell-cycle progression, genetically interacts with Argonaute 1 and modulates epigenetic gene silencing.** *Genetics* 2008, **178:**1311-1325.

95. Fox GL, Rebay I, Hynes RO: **Expression of DFak56, a *Drosophila* homolog of vertebrate focal adhesion kinase, supports a role in cell migration in vivo.** *Proceedings of the National Academy of Sciences* 1999, **96:**14978-14983.

96. Castrillon DH, Wasserman SA: ***diaphanous* is required for cytokinesis in *Drosophila* and shares domains of similarity with the products of the *limb deformity* gene.** *Development* 1994, **120:**3367-3377.

97. Mathe E, Inoue YH, Palframan W, Brown G, Glover DM: **Orbit/Mast, the CLASP orthologue of *Drosophila*, is required for asymmetric stem cell and cystocyte divisions and development of the polarised microtubule network that interconnects oocyte and nurse cells during oogenesis.** *Development* 2003, **130:**901-915.

98. Tepass U, Gruszynski-Defeo E, Haag TA, Omatyar L, Torok T, Hartenstein V: ***shotgun* encodes *Drosophila* E-cadherin and is preferentially required during cell rearrangement in the neurectoderm and other morphogenetically active epithelia.** *Genes Dev* 1996, **10:**672-685.

99. Ji Y, Tulin AV: **Poly(ADP-ribose) controls DE-cadherin-dependent stem cell maintenance and oocyte localization.** *Nature Communications* 2012, **3:**760.

100. Gonzalez-Reyes A, St. Johnston D: **The *Drosophila* AP axis is polarised by the cadherin-mediated positioning of the oocyte.** *Development* 1998, **125:**3635-3644.

101. Yu JX, Guan Z, Nash HA: **The mushroom body defect gene product is an essential component of the meiosis II spindle apparatus in *Drosophila* oocytes.** *Genetics* 2006, **173:**243-253.

102. Dubreuil RR, Maddux PB, Grushko TA, MacVicar GR: **Segregation of two spectrin isoforms: polarized membrane-binding sites direct polarized membrane skeleton assembly.** *Mol Biol Cell* 1997, **8:**1933-1942.

103. de Cuevas M, Lee JK, Spradling AC: **Alpha-spectrin is required for germline cell division and differentiation in the *Drosophila* ovary.** *Development* 1996, **122:**3959-3968.

104. Perrimon N, Engstrom L, Mahowald AP: **Zygotic lethals with specific maternal effect phenotypes in *Drosophila melanogaster*. I. Loci on the X chromosome.** *Genetics* 1989, **121:**333-352.

105. Technau M, Roth S: **The *Drosophila* KASH domain proteins Msp-300 and Klarsicht and the SUN domain protein Klaroid have no essential function during oogenesis.** *Fly* 2008, **2:**82-91.

106. Xie X, Fischer JA: **On the roles of the *Drosophila* KASH domain proteins Msp-300 and Klarsicht.** *Fly* 2008, **2:**74-81.

107. Liu Z, Xie T, Steward R: ***Lis1*, the *Drosophila* homolog of a human lissencephaly disease gene, is required for germline cell division and oocyte differentiation.** *Development* 1999, **126:**4477-4488.

108. Somogyi K, Rørth P: **Cortactin modulates cell migration and ring canal morphogenesis during *Drosophila* oogenesis.** *Mech Dev* 2004, **121:**57-64.

109. Roulier EM, Panzer S, Beckendorf SK: **The Tec29 tyrosine kinase is required during *Drosophila* embryogenesis and interacts with Src64 in ring canal development.** *Mol Cell* 1998, **1:**819-829.

110. Wahlstrom G, Lahti V-P, Pispa J, Roos C, Heino TI: ***Drosophila* non-muscle a-actinin is localized in nurse cell actin bundles and ring canals, but is not required for fertility.** *Mech Dev* 2004, **121:**1377.

111. Wahlstrom G, Norokorpi H-L, Heino TI: ***Drosophila* a-actinin in ovarian follicle cells is regulated by EGFR and Dpp signalling and required for cytoskeletal remodelling.** *Mech Dev* 2006, **123:**801.

112. King RC, Riley SF, Cassidy JD, White PE, Paik YK: **Giant polytene chromosomes from the ovaries of a *Drosophila* mutant.** *Science* 1981, **212:**441-443.

113. Keyes LN, Spradling AC: **The *Drosophila* gene *fs(2)cup* interacts with *otu* to define a cytoplasmic pathway required for the structure and function of germ-line chromosomes.** *Development* 1997, **124:**1419-1431.

114. Gigliotti S, Rotoli D, Manzi A, Graziani F, Malva C: **Female sterile mutations and egg chamber development in *Drosophila melanogaster*.** *Int J Dev Biol* 2000, **44:**581-589.

115. Gigliotti S, Cavaliere V, Manzi A, Tino A, Graziani F, Malva C: **A membrane *guanylate cyclase Drosophila* homolog gene exhibits maternal and zygotic expression.** *Dev Biol* 1993, **159:**450-461.

116. Liu W, Yoon J, Burg M, Chen L, Pak WL: **Molecular characterization of two *Drosophila* guanylate cyclases expressed in the nervous system.** *J Biol Chem* 1995, **270:**12418-12427.

117. Mulligan PK, Campos AR, Jacobs JR: **Mutations in the gene *stand still* disrupt germ cell differentiation in *Drosophila* ovaries.** *Dev Genet* 1996, **18:**316-326.

118. Gutzeit HO, Arendt D: **Blocked endocytotic uptake by the oocyte causes accumulation of vitellogenins in the haemolymph of the female-sterile mutants quit[PX61] and stand still[PS34] of *Drosophila*.** *Cell Tissue Res* 1994, **275:**291-298.

119. Sahut-Barnola I, Pauli D: **The *Drosophila* gene *stand still* encodes a germline chromatin-associated protein that controls the transcription of the ovarian tumor gene.** *Development* 1999, **126:**1917-1926.

120. Rotoli D, Andone S, Tortiglione C, Manzi A, Malva C, Graziani F: ***hold up* is required for establishment of oocyte positioning, follicle cell fate and egg polarity and cooperates with *Egfr* during *Drosophila* oogenesis.** *Genetics* 1998, **148:**767-773.

121. McCaffrey R, St Johnston D, Gonzalez-Reyes A: **A novel mutant phenotype implicates *dicephalic* in cyst formation in the *Drosophila* ovary.** *Dev Dyn* 2006, **235:**908-917.

122. Robinson DN, Cant K, Cooley L: **Morphogenesis of *Drosophila* ovarian ring canals.** *Development* 1994, **120:**2015-2025.

123. Xue F, Cooley L: ***kelch* encodes a component of intercellular bridges in *Drosophila* egg chambers.** *Cell* 1993, **72:**681-693.

124. Hudson AM, Cooley L: ***Drosophila* Kelch functions with Cullin-3 to organize the ring canal actin cytoskeleton.** *J Cell Biol* 2010, **188:**29-37.

125. Dobens LL, Hsu T, Twombly V, Gelbart WM, Raftery LA, Kafatos FC: **The *Drosophila bunched* gene is a homologue of the growth factor stimulated mammalian *TSC-22* sequence and is required during oogenesis.** *Mech Dev* 1997, **65:**197.

126. McDonald JA, Pinheiro EM, Kadlec L, Schüpbach T, Montell DJ: **Multiple EGFR ligands participate in guiding migrating border cells.** *Dev Biol* 2006, **296:**94-103.

127. Erickson MRS, Galletta BJ, Abmayr SM: ***Drosophila myoblast city* encodes a conserved protein that is essential for myoblast fusion, dorsal closure, and cytoskeletal organization.** *J Cell Biol* 1997, **138:**589-603.

128. Karess RE, Chang XJ, Edwards KA, Kulkarni SJ, Aguilera I, Kiehart DP: **The regulatory light chain of nonmuscle myosin is encoded by *spaghetti-squash*, a gene required for cytokinesis in *Drosophila*.** *Cell* 1991, **65:**1177-1189.

129. Jordan P, Karess R: **Myosin light chain–activating phosphorylation sites are required for oogenesis in *Drosophila*.** *J Cell Biol* 1997, **139:**1805-1819.

130. Geisbrecht ER, Montell DJ: **Myosin VI is required for E-cadherin-mediated border cell migration.** *Nat Cell Biol* 2002, **4:**616-620.

131. Wheatley S, Kulkarni S, Karess R: ***Drosophila* nonmuscle myosin II is required for rapid cytoplasmic transport during oogenesis and for axial nuclear migration in early embryos.** *Development* 1995, **121:**1937-1946.

132. Borghese L, Fletcher G, Mathieu J, Atzberger A, Eades WC, Cagan RL, Rørth P: **Systematic analysis of the transcriptional switch inducing migration of border cells.** *Dev Cell* 2006, **10:**497-508.

133. Kapelnikov A, Zelinger E, Gottlieb Y, Rhrissorrakrai K, Gunsalus KC, Heifetz Y: **Mating induces an immune response and developmental switch in the *Drosophila* oviduct.** *Proceedings of the National Academy of Sciences* 2008, **105:**13912-13917.

134. Morgan NS, Heintzelman MB, Mooseker MS: **Characterization of myosin-IA and myosin-IB, two unconventional myosins associated with the *Drosophila* brush border cytoskeleton.** *Dev Biol* 1995, **172:**51-71.

135. MacIver B, McCormack A, Slee R, Bownes M: **Identification of an essential gene encoding a class-V unconventional myosin in *Drosophila melanogaster*.** *Eur J Biochem* 1998, **257:**529-537.

136. Bonafe N, Sellers JR: **Molecular characterization of myosin V from *Drosophila melanogaster*.** *J Muscle Res Cell Motil* 1998, **19:**129-141.

137. Patricio K, Calabria LK, Peixoto PM, Espindola FS, Da Cruz-Landim C: **Characterization and localization of dynein and myosins V and VI in the ovaries of queen bees.** *Cell Biol Int* 2010, **34:**1041-1047.

138. Liu R, Abreu-Blanco MT, Barry KC, Linardopoulou EV, Osborn GE, Parkhurst SM: **Wash functions downstream of Rho and links linear and branched actin nucleation factors.** *Development* 2009, **136:**2849-2860.

139. Rodriguez-Mesa E, Abreu-Blanco MT, Rosales-Nieves AE, Parkhurst SM: **Developmental expression of *Drosophila* Wiskott-Aldrich Syndrome family proteins.** *Dev Dyn* 2012, **241:**608-626.

140. Szafer-Glusman E, Giansanti MG, Nishihama R, Bolival B, Pringle J, Gatti M, Fuller MT: **A role for very-long-chain fatty acids in furrow ingression during cytokinesis in *Drosophila* spermatocytes.** *Curr Biol* 2008, **18:**1426-1431.

141. Baumgartner S, Martin D, Chiquet-Ehrismann R, Sutton J, Desai A, Huang I, Kato K, Hromas R: **The HEM proteins: A novel family of tissue-specific transmembrane proteins expressed from invertebrates through mammals with an essential function in oogenesis.** *J Mol Biol* 1995, **251:**41-49.

142. Röper K, Brown NH: **A Spectraplakin is enriched on the fusome and organizes microtubules during oocyte specification in *Drosophila*.** *Curr Biol* 2004, **14:**99-110.

143. Chang CW, Nashchekin D, Wheatley L, Irion U, Dahlgaard K, Montague TG, Hall J, St Johnston D: **Anterior-posterior axis specification in *Drosophila* oocytes: identification of novel *bicoid* and *oskar* mRNA localization factors.** *Genetics* 2011, **188:**883-896.

144. Hoemann CD, Bergeret E, Guichard A, Griffin-Shea R: **Alternative splicing of the *Drosophila melanogaster rotund RacGAP* gene.** *Gene* 1996, **168:**135-141.

145. Chen J, Godt D, Gunsalus K, Kiss I, Goldberg M, Laski FA: **Cofilin/ADF is required for cell motility during *Drosophila* ovary development and oogenesis.** *Nat Cell Biol* 2001, **3:**204-209.

146. Zhang L, Luo J, Wan P, Wu J, Laski F, Chen J: **Regulation of cofilin phosphorylation and asymmetry in collective cell migration during morphogenesis.** *Development* 2011, **138:**455-464.

147. Wu C, Singaram V, McKim KS: ***mei-38* is required for chromosome segregation during meiosis in *Drosophila* females.** *Genetics* 2008, **180:**61-72.

148. Jang JK, Rahman T, McKim KS: **The kinesinlike protein Subito contributes to central spindle assembly and organization of the meiotic spindle in *Drosophila* oocytes.** *Mol Biol Cell* 2005, **16:**4684-4694.

149. Mesilaty-Gross S, Reich A, Motro B, Wides R: **The *Drosophila STAM* gene homolog is in a tight gene cluster, and its expression correlates to that of the adjacent gene *ial*.** *Gene* 1999, **231:**173-186.

150. Hudson AM, Cooley L: **A subset of dynamic actin rearrangements in *Drosophila* requires the Arp2/3 complex.** *J Cell Biol* 2002, **156:**677-687.

151. Pflanz R, Peter A, Schafer U, Jackle H: **Follicle separation during *Drosophila* oogenesis requires the activity of the Kinesin II-associated polypeptide Kap in germline cells.** *EMBO Rep* 2004, **5:**510-514.

152. Stewart RJ, Pesavento P, Woerpel DN, Goldstein LSB: **Identification and partial characterization of six members of the kinesin superfamily in *Drosophila*.** *Proceedings of the National Academy of Sciences* 1991, **88:**8470-8474.

153. Januschke J, Gervais L, Gillet L, Keryer G, Bornens M, Guichet A: **The centrosome-nucleus complex and microtubule organization in the *Drosophila* oocyte.** *Development* 2006, **133:**129-139.

154. Werner LA, Manseau LJ: **A *Drosophila* gene with predicted rhoGEF, pleckstrin homology and SH3 domains is highly expressed in morphogenic tissues.** *Gene* 1997, **187:**107-114.

155. Zallen JA, Cohen Y, Hudson AM, Cooley L, Wieschaus E, Schejter ED: **SCAR is a primary regulator of Arp2/3-dependent morphological events in *Drosophila*.** *J Cell Biol* 2002, **156:**689-701.

156. Mahajan-Miklos S, Cooley L: **The villin-like protein encoded by the *Drosophila quail* gene is required for actin bundle assembly during oogenesis.** *Cell* 1994, **78:**291-301.

157. Ruden DM, Sollars V, Wang X, Mori D, Alterman M, Lu X: **Membrane fusion proteins are required for *oskar* mRNA localization in the *Drosophila* egg chamber.** *Dev Biol* 2000, **218:**314-325.

158. Leon A, McKearin D: **Identification of TER94, an AAA ATPase protein, as a Bam-dependent component of the *Drosophila* fusome.** *Mol Biol Cell* 1999, **10:**3825-3834.

159. Gates J, Nowotarski SH, Yin H, Mahaffey JP, Bridges T, Herrera C, Homem CC, Janody F, Montell DJ, Peifer M: **Enabled and Capping protein play important roles in shaping cell behavior during *Drosophila* oogenesis.** *Dev Biol* 2009, **333:**90-107.

160. Funaguma S, Hashimoto S, Suzuki Y, Omuro N, Sugano S, Mita K, Katsuma S, Shimada T: **SAGE analysis of early oogenesis in the silkworm, *Bombyx mori*.** *Insect Biochem Mol Biol* 2007, **37:**147-154.

161. Skoulakis EM, Davis RL: **Olfactory learning deficits in mutants for *leonardo,* a *Drosophila* gene encoding a 14-3-3 protein.** *Neuron* 1996, **17:**931.

162. Vaccari T, Ephrussi A: **The fusome and microtubules enrich Par-1 in the oocyte, where it effects polarization in conjunction with Par-3, BicD, Egl, and dynein.** *Curr Biol* 2002, **12:**1524-1528.

163. Kugler JM, Chicoine J, Lasko P: **Bicaudal-C associates with a Trailer Hitch/Me31B complex and is required for efficient Gurken secretion.** *Dev Biol* 2009, **328:**160.

164. Mirouse V, Formstecher E, Couderc J-L: **Interaction between Polo and BicD proteins links oocyte determination and meiosis control in *Drosophila*.** *Development* 2006, **133:**4005-4013.

165. Swaroop A, Sun JW, Paco-Larson ML, Garen A: **Molecular organization and expression of the genetic locus *glued in Drosophila melanogaster*.** *Mol Cell Biol* 1986, **6:**833-841.

166. McGrail M, Gepner J, Silvanovich A, Ludmann S, Serr M, Hays TS: **Regulation of cytoplasmic dynein function in vivo by the *Drosophila* Glued complex.** *J Cell Biol* 1995, **131:**411-425.

167. Carpenter AT: **Egalitarian and the choice of cell fates in *Drosophila melanogaster* oogenesis.** *Ciba Foundation Symposium* 1994, **182:**223-246; discussion 246-254.

168. Mach JM, Lehmann R: **An Egalitarian-BicaudalD complex is essential for oocyte specification and axis determination in *Drosophila*.** *Genes Dev* 1997, **11:**423-435.

169. Maines JZ, Park JK, Williams M, McKearin DM: **Stonewalling *Drosophila* stem cell differentiation by epigenetic controls.** *Development* 2007, **134:**1471-1479.

170. Clark KA, McKearin DM: **The *Drosophila stonewall* gene encodes a putative transcription factor essential for germ cell development.** *Development* 1996, **122:**937-950.

171. Rubsam R, Hollmann M, Simmerl E, Lammermann U, Schafer MA, Buning J, Schafer U: **The *egghead* gene product influences oocyte differentiation by follicle cell-germ cell interactions in *Drosophila melanogaster*.** *Mech Dev* 1998, **72:**131-140.

172. Singh N, Morlock H, Hanes SD: **The Bin3 RNA methyltransferase is required for repression of *caudal* translation in the *Drosophila* embryo.** *Dev Biol* 2011, **352:**104-115.

173. Horowitz H, Berg CA: **The *Drosophila pipsqueak* gene encodes a nuclear BTB-domain-containing protein required early in oogenesis.** *Development* 1996, **122:**1859-1871.

174. Smith THL, Stedronsky K, Morgan B, McGowan RA: **Identification and isolation of a BTBPOZ-containing gene expressed in oocytes and early embryos of the zebrafish *Danio rerio*.** *Genome* 2006, **49:**808-814.

175. Gonzalez-Reyes A, Elliott H, St. Johnston D: **Oocyte determination and the origin of polarity in *Drosophila*: the role of the *spindle* genes.** *Development* 1997, **124:**4927.

176. Gonzalez-Reyes A, St. Johnston RD: **Role of oocyte position in establishment of anterior-posterior polarity in *Drosophila*.** *Science* 1994, **266:**639.

177. McKearin D: **The *Drosophila* fusome, organelle biogenesis and germ cell differentiation: If you build it….** *Bioessays* 1997, **19:**147-152.

178. Lin H, Yue L, Spradling AC: **The *Drosophila* fusome, a germline-specific organelle, contains membrane skeletal proteins and functions in cyst formation.** *Development* 1994, **120:**947-956.

179. Dubreuil RR, Yu J: **Ankyrin and beta-spectrin accumulate independently of alpha-spectrin in *Drosophila*.** *Proceedings of the National Academy of Sciences* 1994, **91:**10285-10289.

180. Lin H, Spradling AC: **Fusome asymmetry and oocyte determination in *Drosophila*.** *Dev Genet* 1995, **16:**6-12.

181. Snapp EL, Iida T, Frescas D, Lippincott-Schwartz J, Lilly MA: **The fusome mediates intercellular endoplasmic reticulum connectivity in *Drosophila* ovarian cysts.** *Mol Biol Cell* 2004, **15:**4512-4521.

182. Lighthouse DV, Buszczak M, Spradling AC: **New components of the *Drosophila* fusome suggest it plays novel roles in signaling and transport.** *Dev Biol* 2008, **317:**59-71.

183. Suzanne M, Irie K, Glise B, Agnes F, Mori E, Matsumoto K, Noselli S: **The *Drosophila* p38 MAPK pathway is required during oogenesis for egg asymmetric development.** *Genes Dev* 1999, **13:**1464-1474.

184. Han ZS, Enslen H, Hu X, Meng X, Wu IH, Barrett T, Davis RJ, Ip YT: **A conserved p38 mitogen-activated protein kinase pathway regulates *Drosophila* immunity gene expression.** *Mol Cell Biol* 1998, **18:**3527-3539.

185. Lane ME, Kalderon D: **Localization and functions of protein kinase A during *Drosophila* oogenesis.** *Mech Dev* 1995, **49:**191-200.

186. Yoshida S, Muller HA, Wodarz A, Ephrussi A: **PKA-R1 spatially restricts Oskar expression for *Drosophila* embryonic patterning.** *Development* 2004, **131:**1401-1410.

187. Park SK, Sedore SA, Cronmiller C, Hirsh J: **Type II cAMP-dependent protein kinase-deficient *Drosophila* are viable but show developmental,**

circadian, and drug response phenotypes. *J Biol Chem* 2000, **275:**20588-20596.

188. Wang Y, Riechmann V: **The role of the actornyosin cytoskeleton in coordination of tissue growth during *Drosophila* oogenesis.** *Curr Biol* 2007, **17:**1349-1355.

189. Yoshimura Y, Nakamura Y, Ando M, Jinno M, Nanno T, Oda T, Koyama N, Shiokawa S: **Protein kinase C mediates gonadotropin-releasing hormone agonist-induced meiotic maturation of follicle-enclosed rabbit oocytes.** *Biol Reprod* 1992, **47:**118-125.

190. Coutelis J-B, Ephrussi A: **Rab6 mediates membrane organization and determinant localization during *Drosophila* oogenesis.** *Development* 2007, **134:**1419-1430.

191. Volpe AM, Horowitz H, Grafer CM, Jackson SM, Berg CA: ***Drosophila rhino* encodes a female-specific chromo-domain protein that affects chromosome structure and egg polarity.** *Genetics* 2001, **159:**1117-1134.

192. Theurkauf WE, Alberts BM, Jan YN, Jongens TA: **A central role for microtubules in the differentiation of *Drosophila* oocytes.** *Development* 1993, **118:**1169-1180.

193. Buttgereit D, Leiss D, Michiels F, Renkawitz-Pohl R: **During *Drosophila* embryogenesis the 1 tubulin gene is specifically expressed in the nervous system and the apodemes.** *Mech Dev* 1991, **33:**107-118.

194. Kimble M, Incardona J, Raff EC: **A variant -tubulin isoform of *Drosophila melanogaster* (-3) is expressed primarily in tissues of mesodermal origin in embryos and pupae, and is utilized in populations of transient microtubules.** *Dev Biol* 1989, **131:**415-429.

195. Wehr K, Swan A, Schüpbach T: **Deadlock, a novel protein of *Drosophila*, is required for germline maintenance, fusome morphogenesis and axial patterning in oogenesis and associates with centrosomes in the early embryo.** *Dev Biol* 2006, **294:**406-417.

196. Wiese C: **Distinct Dgrip84 Isoforms Correlate with Distinct γ-Tubulins in *Drosophila*.** *Mol Biol Cell* 2008, **19:**368-377.

197. Tavosanis G, Llamazares S, Goulielmos G, Gonzalez C: **Essential role for [gamma]-tubulin in the acentriolar female meiotic spindle of *Drosophila*.** *EMBO J* 1997, **16:**1809-1819.

198. Benton R, Palacios IM, St. Johnston D: ***Drosophila* 14-3-3/PAR-5 is an essential mediator of PAR-1 function in axis formation.** *Dev Cell* 2002, **3:**659-671.

199. Shulman JM, Benton R, St. Johnston D: **The *Drosophila* homolog of *C. elegans* PAR-1 organizes the oocyte cytoskeleton and directs *oskar* mRNA localization to the posterior pole.** *Cell* 2000, **101:**377-388.

200. Tomancak P, Piano F, Riechmann V, Gunsalus KC, Kemphues KJ, Ephrussi A: **A *Drosophila melanogaster* homologue of *Caenorhabditis elegans* par-1 acts at an early step in embryonic-axis formation.** *Nat Cell Biol* 2000, **2:**458-460.

201. Martin SG, St Johnston D: **A role for *Drosophila* LKB1 in anterior-posterior axis formation and epithelial polarity.** *Nature* 2003, **421:**379-384.

202. Huynh J-R, Petronczki M, Knoblich JA, Johnston DS: **Bazooka and PAR-6 are required with PAR-1 for the maintenance of oocyte fate in *Drosophila*.** *Curr Biol* 2001, **11:**901-906.

203. Beatty RA, Waddington CH: **XVIII.—Studies on reproduction in wild-type and female sterile mutants of *Drosophila melanogaster* (Meig).** *Proc R Soc Edin* 1949, **63:**249-270.

204. McGrail M, Hays TS: **The microtubule motor cytoplasmic dynein is required for spindle orientation during germline cell divisions and oocyte differentiation in *Drosophila*.** *Development* 1997, **124:**2409-2419.

205. Mische S, Li M, Serr M, Hays TS: **Direct observation of regulated ribonucleoprotein transport across the nurse cell/oocyte boundary.** *Mol Biol Cell* 2007, **18:**2254-2263.

206. Brendza RP, Serbus LR, Saxton WM, Duffy JB: **Posterior localization of dynein and dorsal-ventral axis formation depend on kinesin in *Drosophila* oocytes.** *Curr Biol* 2002, **12:**1541-1545.

207. Dick T, Ray K, Salz HK, Chia W: **Cytoplasmic dynein (*ddlc1*) mutations cause morphogenetic defects and apoptotic cell death in *Drosophila melanogaster*.** *Mol Cell Biol* 1996, **16:**1966-1977.

208. Palacios IM, St. Johnston D: ***Kinesin light chain*-independent function of the *Kinesin heavy chain* in cytoplasmic streaming and posterior localisation in the *Drosophila* oocyte.** *Development* 2002, **129:**5473-5485.

209. Ganguly S, Williams LS, Palacios IM, Goldstein RE: **Cytoplasmic streaming in *Drosophila* oocytes varies with kinesin activity and correlates with the microtubule cytoskeleton architecture.** *Proceedings of the National Academy of Sciences* 2012, **109:**15109-15114.

210. Guichard A, Roark M, Ronshaugen M, Bier E: ***brother of rhomboid*, a *rhomboid*-related gene expressed during early *Drosophila* oogenesis, promotes EGF-R/MAPK signaling.** *Dev Biol* 2000, **226:**255-266.

211. Mack PD, Kapelnikov A, Heifetz Y, Bender M: **Mating-responsive genes in reproductive tissues of female *Drosophila melanogaster*.** *Proceedings of the National Academy of Sciences* 2006, **103:**10358-10363.

212. Sung H-H, Telley IA, Papadaki P, Ephrussi A, Surrey T, Rørth P: ***Drosophila* ensconsin promotes productive recruitment of kinesin-1 to microtubules.** *Dev Cell* 2008, **15:**866-876.

213. Eberl DF, Lorenz LJ, Melnick MB, Sood V, Lasko P, Perrimon N: **A new enhancer of position-effect variegation in *Drosophila melanogaster* encodes a putative RNA helicase that binds chromosomes and is regulated by the cell cycle.** *Genetics* 1997, **146:**951-963.

214. Fitzpatrick KA, Gorski SM, Ursuliak Z, Price JV: **Expression of protein tyrosine phosphatase genes during oogenesis in *Drosophila melanogaster*.** *Mech Dev* 1995, **53:**171-183.

215. Shcherbata HR, Althauser C, Findley SD, Ruohola-Baker H: **The mitotic-to-endocycle switch in *Drosophila* follicle cells is executed by Notch-dependent regulation of G1/S, G2/M and M/G1 cell-cycle transitions.** *Development* 2004, **131:**3169-3181.

216. Kocabas AM, Crosby J, Ross PJ, Otu HH, Beyhan Z, Can H, Tam W-L, Rosa GJM, Halgren RG, Lim B, et al: **The transcriptome of human oocytes.** *Proceedings of the National Academy of Sciences* 2006, **103:**14027-14032.

217. Klovstad M, Abdu U, Schüpbach T: ***Drosophila brca2* is required for mitotic and meiotic DNA repair and efficient activation of the meiotic recombination checkpoint.** *PLoS Genet* 2008, **4:**e31.

218. Calvi BR, Lilly MA, Spradling AC: **Cell cycle control of chorion gene amplification.** *Genes Dev* 1998, **12:**734-744.

219. Jacobs HW, Knoblich JA, Lehner CF: ***Drosophila* Cyclin B3 is required for female fertility and is dispensable for mitosis like Cyclin B.** *Genes Dev* 1998, **12:**3741-3751.

220. Von Stetina JR, Tranguch S, Dey SK, Lee LA, Cha B, Drummond-Barbosa D: **Alpha-Endosulfine is a conserved protein required for oocyte meiotic maturation in *Drosophila*.** *Development* 2008, **135:**3697-3706.

221. Chen X, Oh SW, Zheng Z, Chen HW, Shin HH, Hou SX: **Cyclin D-Cdk4 and cyclin E-Cdk2 regulate the Jak/STAT signal transduction pathway in *Drosophila*.** *Dev Cell* 2003, **4:**179-190.

222. Sauer K, Weigmann K, Sigrist S, Lehner CF: **Novel members of the cdc2-related kinase family in *Drosophila*: cdk4/6, cdk5, PFTAIRE, and PITSLRE kinase.** *Mol Biol Cell* 1996, **7:**1759-1769.

223. Larochelle S, Pandur J, Fisher RP, Salz HK, Suter B: **Cdk7 is essential for mitosis and for in vivo Cdk-activating kinase activity.** *Genes Dev* 1998, **12:**370-381.

224. Vardy L, Pesin JA, Orr-Weaver TL: **Regulation of Cyclin A protein in meiosis and early embryogenesis.** *Proceedings of the National Academy of Sciences* 2009, **106:**1838-1843.

225. Dalby B, Glover DM: **3' non-translated sequences in *Drosophila* cyclin B transcripts direct posterior pole accumulation late in oogenesis and peri-nuclear association in syncytial embryos.** *Development* 1992, **115:**989-997.

226. Whitfield WGF, Gonzalez C, Sanchez-Herrero E, Glover DM: **Transcripts of one of two *Drosophila* cyclin genes become localized in pole cells during embryogenesis.** *Nature* 1989, **338:**337-340.

227. Swan A, Schüpbach T: **The Cdc20 (Fzy)/Cdh1-related protein, Cort, cooperates with Fzy in cyclin destruction and anaphase progression in meiosis I and II in *Drosophila*.** *Development* 2007, **134:**891-899.

228. Lahue EE, Smith AV, Orr-Weaver TL: **A novel cyclin gene from *Drosophila* complements CLN function in yeast.** *Genes Dev* 1991, **5:**2166-2175.

229. Royzman I, Orr-Weaver TL: **S phase and differential DNA replication during *Drosophila* oogenesis.** *Genes Cells* 1998, **3:**767-776.

230. Doronkin S, Djagaeva I, Beckendorf SK: **The COP9 signalosome promotes degradation of Cyclin E during early *Drosophila* oogenesis.** *Dev Cell* 2003, **4:**699-710.

231. Doronkin S, Djagaeva I, Beckendorf SK: **CSN5/Jab1 mutations affect axis formation in the *Drosophila* oocyte by activating a meiotic checkpoint.** *Development* 2002, **129:**5053-5064.

232. Oren-Giladi P, Krieger O, Edgar BA, Chamovitz DA, Segal D: **Cop9 signalosome subunit 8 (CSN8) is essential for *Drosophila* development.** *Genes Cells* 2008, **13:**221-231.

233. Kolonin MG, Finley Jr RL: **A Role for Cyclin J in the Rapid Nuclear Division Cycles of Early *Drosophila* Embryogenesis.** *Dev Biol* 2000, **227:**661-672.

234. Althoff F, Viktorinova I, Kastl J, Lehner CF: ***Drosophila* Cyclin J is a mitotically stable Cdk1 partner without essential functions.** *Dev Biol* 2009, **333:**263-272.

235. Swan A, Barcelo G, Schüpbach T: ***Drosophila Cks30A* interacts with Cdk1 to target Cyclin A for destruction in the female germline.** *Development* 2005, **132:**3669-3678.

236. Gallant P, Shiio Y, Cheng PF, Parkhurst SM, Eisenman RN: **Myc and Max homologs in *Drosophila*.** *Science* 1996, **274:**1523-1527.

237. Myster DL, Bonnette PC, Duronio RJ: **A role for the DP subunit of the E2F transcription factor in axis determination during *Drosophila* oogenesis.** *Development* 2000, **127:**3249-3261.

238. Cayirlioglu P, Ward WO, Key SCS, Duronio RJ: **Transcriptional repressor functions of *Drosophila* E2F1 and E2F2 cooperate to inhibit genomic DNA synthesis in ovarian follicle cells.** *Mol Cell Biol* 2003, **23:**2123-2134.

239. Pennetta G, Pauli D: **The *Drosophila sin3* gene encodes a widely distributed transcription factor essential for embryonic viability.** *Dev Genes Evol* 1998, **208:**531-536.

240. Quinn LM, Herr A, McGarry TJ, Richardson H: **The *Drosophila* Geminin homolog: roles for Geminin in limiting DNA replication, in anaphase and in neurogenesis.** *Genes Dev* 2001, **15:**2741-2754.

241. Xiang Y, Takeo S, Florens L, Hughes SE, Huo L-J, Gilliland WD, Swanson SK, Teeter K, Schwartz JW, Washburn MP, et al: **The Inhibition of Polo Kinase by matrimony maintains G2 Arrest in the meiotic cell cycle.** *PLoS Biol* 2007, **5:**e323.

242. Harris D, Orme C, Kramer J, Namba L, Champion M, Palladino MJ, Natzle J, Hawley RS: **A deficiency screen of the major autosomes identifies a gene (*matrimony*) that is haplo-insufficient for achiasmate segregation in *Drosophila* oocytes.** *Genetics* 2003, **165:**637-652.

243. Bentley AM, Williams BC, Goldberg ML, Andres AJ: **Phenotypic characterization of *Drosophila ida* mutants: defining the role of APC5 in cell cycle progression.** *J Cell Sci* 2002, **115:**949-961.

244. Courtot C, Fankhauser C, Simanis V, Lehner CF: **The *Drosophila* cdc25 homolog *twine* is required for meiosis.** *Development* 1992, **116:**405-416.

245. Alphey L, Jimenez J, White-Cooper H, Dawson I, Nurse P, Glover DM: ***twine*, a cdc25 homolog that functions in the male and female germline of *Drosophila*.** *Cell* 1992, **69:**977-988.

246. Rickmyre JL, Dasgupta S, Ooi DL, Keel J, Lee E, Kirschner MW, Waddell S, Lee LA: **The *Drosophila* homolog of *MCPH1*, a human microcephaly gene, is required for genomic stability in the early embryo.** *J Cell Sci* 2007, **120:**3565-3577.

247. Hongay CF, Orr-Weaver TL: ***Drosophila* Inducer of MEiosis 4 (IME4) is required for Notch signaling during oogenesis.** *Proceedings of the National Academy of Sciences* 2011, **108:**14855-14860.

248. Hongay C, Fink G, Orr-Weaver T: **Role of *Drosophila* Ime4 and Ime2 in the initiation of meiosis.** In *Program and Abstracts 49th Annual Drosophila Research Conference, San Diego, CA, 2008*. pp. 74; 2008:74.

249. Archambault V, Zhao X, White-Cooper H, Carpenter ATC, Glover DM: **Mutations in *Drosophila Greatwall/Scant* reveal its roles in mitosis and meiosis and interdependence with polo kinase.** *PLoS Genet* 2007, **3:**e200.

250. Bakhrat A, Pritchett T, Peretz G, McCall K, Abdu U: ***Drosophila* Chk2 and p53 proteins induce stage-specific cell death independently during oogenesis.** *Apoptosis* 2010, **15:**1425-1434.

251. Oishi I, Sugiyama S, Otani H, Yamamura H, Nishida Y, Minami Y: **A novel *Drosophila* nuclear protein serine/threonine kinase expressed in the germline during its establishment.** *Mech Dev* 1998, **71:**49-63.

252. Minestrini G, Mathe E, Glover DM: **Domains of the Pavarotti kinesin-like protein that direct its subcellular distribution: effects of mislocalisation on the tubulin and actin cytoskeleton during *Drosophila* oogenesis.** *J Cell Sci* 2002, **115:**725.

253. Airoldi SJ, McLean PF, Shimada Y, Cooley L: **Intercellular protein movement in syncytial *Drosophila* follicle cells.** *J Cell Sci* 2011, **124:**4077-4086.

254. Reed BH, Orr-Weaver TL: **The *Drosophila* gene *morula* inhibits mitotic functions in the endo cell cycle and the mitotic cell cycle.** *Development* 1997, **124:**3543-3553.

255. Yamaguchi M, Nishida Y, Moriuchi T, Hirose F, Hui CC, Suzuki Y, Matsukage A: ***Drosophila* proliferating cell nuclear antigen (cyclin) gene: structure, expression during development, and specific binding of homeodomain proteins to its 5'-flanking region.** *Mol Cell Biol* 1990, **10:**872-879.

256. Brodsky MH, Sekelsky JJ, Tsang G, Hawley RS, Rubin GM: ***mus304* encodes a novel DNA damage checkpoint protein required during *Drosophila* development.** *Genes Dev* 2000, **14:**666-678.

257. Beall EL, Manak JR, Zhou S, Bell M, Lipsick JS, Botchan MR: **Role for a *Drosophila* Myb-containing protein complex in site-specific DNA replication.** *Nature* 2002, **420:**833-837.

258. Georlette D, Ahn S, MacAlpine DM, Cheung E, Lewis PW, Beall EL, Bell SP, Speed T, Manak JR, Botchan MR: **Genomic profiling and expression studies reveal both positive and negative activities for the *Drosophila***

Myb-MuvB/dREAM complex in proliferating cells. *Genes Dev* 2007, **21:**2880-2896.

259. Bhaskar PK, Mukherjee A, Mutsuddi M: **Dynamic pattern of expression of *dlin52*, a member of the Myb/MuvB complex, during *Drosophila* development.** *Gene Expr Patterns* 2012, **12:**77-84.

260. Zaffran S, Chartier A, Gallant P, Astier M, Arquier N, Doherty D, Gratecos D, Semeriva M: **A *Drosophila* RNA helicase gene, *pitchoune*, is required for cell growth and proliferation and is a potential target of d-Myc.** *Development* 1998, **125:**3571-3584.

261. Rørth P, Szabo K, Texido G: **The level of C/EBP protein is critical for cell migration during *Drosophila* oogenesis and is tightly controlled by regulated degradation.** *Mol Cell* 2000, **6:**23-30.

262. Dawson IA, Roth S, Artavanis-Tsakonas S: **The *Drosophila* cell cycle gene *fizzy* is required for normal degradation of cyclins A and B during mitosis and has homology to the *CDC20* gene of *Saccharomyces cerevisiae*.** *J Cell Biol* 1995, **129:**725.

263. Chen PS, Stumm-Zollinger E, Aigaki T, Balmer J, Bienz M, Bohlen P: **A male accessory gland peptide that regulates reproductive behavior of female *Drosophila melanogaster*.** *Cell* 1988, **54:**291-298.

264. Ghabrial A, Schüpbach T: **Activation of a meiotic checkpoint regulates translation of Gurken during *Drosophila* oogenesis.** *Nat Cell Biol* 1999, **1:**354-357.

265. Van Buskirk C, Hawkins NC, Schüpbach T: **Encore is a member of a novel family of proteins and affects multiple processes in *Drosophila* oogenesis.** *Development* 2000, **127:**4753-4762.

266. Page AW, Orr-Weaver TL: **The *Drosophila* genes *grauzone* and *cortex* are necessary for proper female meiosis.** *J Cell Sci* 1996, **109:**1707-1715.

267. Kerrebrock AW, Moore DP, Wu JS, Orr-Weaver TL: **mei-S332, a *Drosophila* protein required for sister-chromatid cohesion, can localize to meiotic centromere regions.** *Cell* 1995, **83:**247-256.

268. Kumar R, Bourbon H-M, de Massy B: **Functional conservation of Mei4 for meiotic DNA double-strand break formation from yeasts to mice.** *Genes Dev* 2010, **24:**1266-1280.

269. McKim KS, Hayashi-Hagihara A: ***mei-W68* in *Drosophila melanogaster* encodes a Spo11 homolog: evidence that the mechanism for initiating meiotic recombination is conserved.** *Genes Dev* 1998, **12:**2932-2942.

270. Riparbelli MG: ***cortex,* a maternal-effect mutation showing mitotic defects during early embryogenesis of *Drosophila melanogaster*.** *Anim Biol* 1993, **2:**11-17.

271. Chu T, Henrion G, Haegeli V, Strickland S: ***cortex,* a *Drosophila* gene required to complete oocyte meiosis, is a member of the Cdc20/fizzy protein family.** *Genesis* 2001, **29:**141-152.

272. Chen B, Harms E, Chu T, Henrion G, Strickland S: **Completion of meiosis in *Drosophila* oocytes requires transcriptional control by Grauzone, a new zinc finger protein.** *Development* 2000, **127:**1243-1251.

273. Kasravi A, Walter MF, Brand S, Mason JM, Biessmann H: **Molecular cloning and tissue-specific expression of the *mutator2* gene (*mu2*) in *Drosophila melanogaster*.** *Genetics* 1999, **152:**1025-1035.

274. Mason JM, Champion LE, Hook G: **Germ-line effects of a mutator, *mu2,* in *Drosophila melanogaster*.** *Genetics* 1997, **146:**1381-1397.

275. Jin Z, Homola EM, Goldbach P, Choi YH, Brill JA, Campbell SD: ***Drosophila* Myt1 is a Cdk1 inhibitory kinase that regulates multiple aspects of cell cycle behavior during gametogenesis.** *Development* 2005, **132:**4075-4085.

276. Bickel SE, Orr-Weaver TL, Balicky EM: **The sister-chromatid cohesion protein ORD is required for chiasma maintenance in *Drosophila* oocytes.** *Curr Biol* 2002, **12:**925-929.

277. Orr-Weaver TL: **Meiosis in *Drosophila*: seeing is believing.** *Proceedings of the National Academy of Sciences* 1995, **92:**10443-10449.

278. Gilliland WD, Hughes SE, Cotitta JL, Takeo S, Xiang Y, Hawley RS: **The multiple roles of Mps1 in *Drosophila* female meiosis.** *PLoS Genet* 2007, **3:**e113.

279. O'Tousa J: **Meiotic chromosome behavior influenced by mutation-altered disjunction in *Drosophila melanogaster* females.** *Genetics* 1982, **102:**503.

280. Zhang P, Knowles BA, Goldstein LSB, Hawley RS: **A kinesin-like protein required for distributive chromosome segregation in *Drosophila*.** *Cell* 1990, **62:**1053.

281. Theurkauf WE, Hawley RS: **Meiotic spindle assembly in *Drosophila* females: behavior of nonexchange chromosomes and the effects of mutations in the nod kinesin-like protein.** *J Cell Biol* 1992, **116:**1167-1180.

282. Ejima A, Tsuda M, Takeo S, Ishii K, Matsuo T, Aigaki T: **Expression level of *sarah,* a homolog of DSCR1, is critical for ovulation and female courtship behavior in *Drosophila melanogaster*.** *Genetics* 2004, **168:**2077-2087.

283. Takeo S, Tsuda M, Akahori S, Matsuo T, Aigaki T: **The calcineurin regulator Sra plays an essential role in female meiosis in *Drosophila*.** *Curr Biol* 2006, **16:**1435-1440.

284. Ayyub C: **Cullin-5 and cullin-2 play a role in the development of neuromuscular junction and the female germ line of *Drosophila*.** *J Genet* 2011, **90:**239-249.

285. Lin HC, Wu JT, Tan BC, Chien CT: **Cul4 and DDB1 regulate Orc2 localization, BrdU incorporation and Dup stability during gene amplification in *Drosophila* follicle cells.** *J Cell Sci* 2009, **122:**2393-2401.

286. Whittaker AJ, Royzman I, Orr-Weaver TL: ***Drosophila* Double parked: a conserved, essential replication protein that colocalizes with the origin recognition complex and links DNA replication with mitosis and the down-regulation of S phase transcripts.** *Genes Dev* 2000, **14:**1765-1776.

287. Kugler JM, Lem C, Lasko P: **Reduced *cul-5* activity causes aberrant follicular morphogenesis and germ cell loss in *Drosophila* oogenesis.** *PLoS ONE* 2010, **5:**e9048.

288. Styhler S, Nakamura A, Lasko P: **VASA localization requires the SPRY-domain and SOCS-box containing protein, GUSTAVUS.** *Dev Cell* 2002, **3:**865-876.

289. Canning M, Kirby R, Finnegan D: **UbcD4, a ubiquitin-conjugating enzyme in *Drosophila melanogaster* expressed in pole cells.** *Mol Genet Genomics* 2002, **266:**907-913.

290. Chesnokov I, Gossen M, Remus D, Botchan M: **Assembly of functionally active *Drosophila* origin recognition complex from recombinant proteins.** *Genes Dev* 1999, **13:**1289-1296.

291. Asano M, Wharton RP: **E2F mediates developmental and cell cycle regulation of ORC1 in *Drosophila*.** *EMBO J* 1999, **18:**2435-2448.

292. Cayirlioglu P, Bonnette PC, Dickson MR, Duronio RJ: ***Drosophila E2f2* promotes the conversion from genomic DNA replication to gene amplification in ovarian follicle cells.** *Development* 2001, **128:**5085-5098.

293. Ayyar S, Jiang J, Collu A, White-Cooper H, White RA: ***Drosophila* TGIF is essential for developmentally regulated transcription in spermatogenesis.** *Development* 2003, **130:**2841-2852.

294. Wang Z, Mann RS: **Requirement for two nearly identical TGIF-related homeobox genes in *Drosophila* spermatogenesis.** *Development* 2003, **130:**2853-2865.

295. Fogarty P, Campbell SD, Abu-Shumays R, de Saint Phalle B, Yu KR, Uy GL, Goldberg ML, Sullivan W: **The *Drosophila grapes* gene is related to checkpoint gene *chk-1 rad27* and is required for late syncytial division fidelity.** *Curr Biol* 1997, **7:**418-426.

296. Iida T, Lilly MA: ***missing oocyte* encodes a highly conserved nuclear protein required for the maintenance of the meiotic cycle and oocyte identity in *Drosophila*.** *Development* 2004, **131:**1029-1039.

297. Senger S, Csokmay J, Tanveer A, Jones TI, Sengupta P, Lilly MA: **The nucleoporin Seh1 forms a complex with Mio and serves an essential tissue-specific function in *Drosophila* oogenesis.** *Development* 2011, **138:**2133-2142.

298. Kiger AA, Gigliotti S, Fuller MT: **Developmental genetics of the essential *Drosophila* nucleoporin *nup154*: allelic differences due to an outward-directed promoter in the P-element 3' end.** *Genetics* 1999, **153:**799-812.

299. Gigliotti S, Callaini G, Andone S, Riparbelli MG, Pernas-Alonso R, Hoffmann G, Graziani F, Malva C: ***Nup154,* a new *Drosophila* gene essential for male and female gametogenesis is related to the *nup155* vertebrate nucleoporin gene.** *J Cell Biol* 1998, **142:**1195-1207.

300. Hatsumi M, Endow SA: **The *Drosophila* ncd microtubule motor protein is spindle-associated in meiotic and mitotic cells.** *J Cell Sci* 1992, **103:**1013-1020.

301. Komma DJ, Horne AS, Endow SA: **Separation of meiotic and mitotic effects of *claret non-disjunctional* on chromosome segregation in *Drosophila*.** *EMBO J* 1991, **10:**419-424.

302. Zou J, Hallen MA, Yankel CD, Endow SA: **A microtubule-destabilizing kinesin motor regulates spindle length and anchoring in oocytes.** *J Cell Biol* 2008, **180:**459-466.

303. Takeo S, Lake CM, Morais-de-Sá E, Sunkel CE, Hawley RS: **Synaptonemal complex-dependent centromeric clustering and the initiation of synapsis in *Drosophila* oocytes.** *Curr Biol* 2011, **21:**1845-1851.

304. Tanneti NS, Landy K, Joyce EF, McKim KS: **A pathway for synapsis Initiation during zygotene in *Drosophila* oocytes.** *Curr Biol* 2011, **21:**1852-1857.

305. Manheim EA, McKim KS: **The synaptonemal complex component C(2)M regulates meiotic crossing over in *Drosophila*.** *Curr Biol* 2003, **13:**276-285.

306. Joyce EF, McKim KS: **Meiotic checkpoints and the interchromosomal effect on crossing over in *Drosophila* females.** *Fly* 2011, **5:**134-140.

307. Page SL, Khetani RS, Lake CM, Nielsen RJ, Jeffress JK, Warren WD, Bickel SE, Hawley RS: ***corona* is required for higher-order assembly of**

transverse filaments into full-length synaptonemal complex in *Drosophila* oocytes. *PLoS Genet* 2008, **4:**e1000194.

308. Gause M, Webber HA, Misulovin Z, Haller G, Rollins RA, Eissenberg JC, Bickel SE, Dorsett D: **Functional links between *Drosophila* Nipped-B and cohesin in somatic and meiotic cells.** *Chromosoma* 2008, **117:**51-66.

309. Joyce EF, McKim KS: **Chromosome axis defects Induce a checkpoint-mediated delay and Interchromosomal effect on crossing over during *Drosophila* meiosis.** *PLoS Genet* 2010, **6**.

310. Bennett D, Alphey L: **Cloning and expression of *mars*, a novel member of the guanylate kinase associated protein family in *Drosophila*.** *Gene Expr Patterns* 2004, **4:**529-535.

311. Emmons S, Phan H, Calley J, Chen W, James B, Manseau L: ***cappuccino*, a *Drosophila* maternal effect gene required for polarity of the egg and embryo, is related to the vertebrate *limb deformity* locus.** *Genes Dev* 1995, **9:**2482-2494.

312. Dahlgaard K, Raposo AA, Niccoli T, St Johnston D: **Capu and Spire assemble a cytoplasmic actin mesh that maintains microtubule organization in the *Drosophila* oocyte.** *Dev Cell* 2007, **13:**539-553.

313. Roth S, Neuman-Silberberg FS, Barcelo G, Schüpbach T: **cornichon and the EGF receptor signaling process are necessary for both anterior-posterior and dorsal-ventral pattern formation in *Drosophila*.** *Cell* 1995, **81:**967.

314. Schüpbach T: **Germ line and soma cooperate during oogenesis to establish the dorsoventral pattern of egg shell and embryo in *Drosophila melanogaster*.** *Cell* 1987, **49:**699-707.

315. Haenlin M, Roos C, Cassab A, Mohier E: **Oocyte-specific transcription of *fs(1)K10*: a *Drosophila* gene affecting dorsal-ventral developmental polarity.** *EMBO J* 1987, **6:**801-807.

316. Kelkar A, Dobberstein B: **Sec61beta, a subunit of the Sec61 protein translocation channel at the endoplasmic reticulum, is involved in the transport of Gurken to the plasma membrane.** *BMC Cell Biol* 2009, **10:**11.

317. Zhao D, Woolner S, Bownes M: **The Mirror transcription factor links signalling pathways in *Drosophila* oogenesis.** *Dev Genes Evol* 2000, **210:**449-457.

318. Cinnamon E, Gur-Wahnon D, Helman A, St Johnston D, Jimenez G, Paroush Z: **Capicua integrates input from two maternal systems in *Drosophila* terminal patterning.** *EMBO J* 2004, **23:**4571-4582.

319. Fuchs A, Cheung LS, Charbonnier E, Shvartsman SY, Pyrowolakis G: **Transcriptional interpretation of the EGF receptor signaling gradient.** *Proceedings of the National Academy of Sciences* 2012, **109:**1572-1577.

320. Jimenez G, Guichet A, Ephrussi A, Casanova J: **Relief of gene repression by torso RTK signaling: role of *capicua* in *Drosophila* terminal and dorsoventral patterning.** *Genes Dev* 2000, **14:**224-231.

321. Hong CC, Hashimoto C: **An unusual mosaic protein with a protease domain, encoded by the *nudel* gene, is involved in defining embryonic dorsoventral polarity in *Drosophila*.** *Cell* 1995, **82:**785-794.

322. Kugler JM, Lasko P: **Localization, anchoring and translational control of *oskar, gurken, bicoid* and *nanos* mRNA during *Drosophila* oogenesis.** *Fly* 2009, **3:**15-28.

323. Geng C, Macdonald PM: **Identification of genes that influence gurken expression.** *Fly* 2007, **1:**259-267.

324. Yakoby N, Bristow CA, Gong D, Schafer X, Lembong J, Zartman JJ, Halfon MS, Schüpbach T, Shvartsman SY: **A combinatorial code for pattern formation in *Drosophila* oogenesis.** *Dev Cell* 2008, **15:**725-737.

325. Neuman-Silberberg FS, Schüpbach T: **The *Drosophila* TGF-alpha-like protein Gurken: expression and cellular localization during *Drosophila* oogenesis.** *Mech Dev* 1996, **59:**105-113.

326. Neuman-Silberberg FS, Schüpbach T: **Dorsoventral axis formation in *Drosophila* depends on the correct dosage of the gene *gurken*.** *Development* 1994, **120:**2457-2463.

327. Pek JW, Lim AK, Kai T: ***Drosophila* maelstrom ensures proper germline stem cell lineage differentiation by repressing *microRNA-7*.** *Dev Cell* 2009, **17:**417-424.

328. Clegg NC, Findley SF, Mahowald AM, Ruohola-Baker H: ***maelstrom* is required to position the MTOC in stage 2-6 *Drosophila* oocytes.** *Dev Genes Evol* 2001, **211:**44.

329. Technau M, Knispel M, Roth S: **Molecular mechanisms of EGF signaling-dependent regulation of pipe, a gene crucial for dorsoventral axis formation in *Drosophila*.** *Dev Genes Evol* 2012, **222:**1-17.

330. Zhang Z, Zhu X, Stevens LM, Stein D: **Distinct functional specificities are associated with protein isoforms encoded by the *Drosophila* dorsal-ventral patterning gene *pipe*.** *Development* 2009, **136:**2779-2789.

331. Kooistra R, Vreeken K, Zonneveld JBM, de Jong A, Eeken JCJ, Osgood CJ, Buerstedde JM, Lohman PHM, Pastink A: **The *Drosophila melanogaster***

RAD54 **homolog,** ***DmRAD54,*** **is involved in the repair of radiation damage and recombination.** *Mol Cell Biol* 1997, **17:**6097.

332. Schüpbach T, Wieschaus E: **Female sterile mutations on the second chromosome of** ***Drosophila melanogaster*****. II. Mutations blocking oogenesis or altering egg morphology.** *Genetics* 1991, **129:**1119-1136.

333. Ghabrial A, Ray RP, Schüpbach T: ***okra*** **and** ***spindle-B*** **encode components of the** ***RAD52*** **DNA repair pathway and affect meiosis and patterning in** ***Drosophila*** **oogenesis.** *Genes Dev* 1998, **12:**2711-2723.

334. Christerson LB, McKearin DM: ***orb*** **is required for anteroposterior and dorsoventral patterning during** ***Drosophila*** **oogenesis.** *Genes Dev* 1994, **8:**614-628.

335. Norvell A, Kelley RL, Wehr K, Schüpbach T: **Specific isoforms of** ***squid,*** **a** ***Drosophila*** **hnRNP, perform distinct roles in Gurken localization during oogenesis.** *Genes Dev* 1999, **13:**864-876.

336. Norvell A, Debec A, Finch D, Gibson L, Thoma B: **Squid is required for efficient posterior localization of** ***oskar*** **mRNA during** ***Drosophila*** **oogenesis.** *Dev Genes Evol* 2005, **215:**340-349.

337. Kelley RL: **Initial organization of the** ***Drosophila*** **dorsoventral axis depends on an RNA-binding protein encoded by the** ***squid*** **gene.** *Genes Dev* 1993, **7:**948-960.

338. Siebel CW, Admon A, Rio DC: **Soma-specific expression and cloning of PSI, a negative regulator of P element pre-mRNA splicing.** *Genes Dev* 1995, **9:**269-283.

339. Haynes SR, Johnson D, Raychaudhuri G, Beyer AL: **The *Drosophila Hrb87F* gene encodes a new member of the A and B hnRNP protein group.** *Nucleic Acids Res* 1991, **19:**25-31.

340. Beccari S, Teixeira Ls, Rørth P: **The JAK/STAT pathway is required for border cell migration during *Drosophila* oogenesis.** *Mech Dev* 2002, **111:**115-123.

341. Kokai E, Paldy FS, Somogyi K, Chougule A, Pal M, Kerekes E, Deak P, Friedrich P, Dombradi V, Adam G: **CalpB modulates border cell migration in *Drosophila* egg chambers.** *BMC Dev Biol* 2012, **12:**20.

342. Larkin MK, Holder K, Yost C, Giniger E, Ruohola-Baker H: **Expression of constitutively active Notch arrests follicle cells at a precursor stage during *Drosophila* oogenesis and disrupts the anterior-posterior axis of the oocyte.** *Development* 1996, **122:**3639-3650.

343. Lehmann R, Dietrich U, Jimenez F, Campos-Ortega JA: **Mutations of early neurogenesis in *Drosophila*.** *Roux's Arch Dev Biol* 1981, **190:**226.

344. Jiménez F, Campos-Ortega JA: **Maternal effects of zygotic mutants affecting early neurogenesis in *Drosophila*.** *Dev Genes Evol* 1982, **191:**191.

345. Goode S, Wright D, Mahowald AP: **The neurogenic locus *brainiac* cooperates with the *Drosophila* EGF receptor to establish the ovarian follicle and to determine its dorsal-ventral polarity.** *Development* 1992, **116:**177-192.

346. Bettler D, Pearson S, Yedvobnick B: **The nuclear protein encoded by the *Drosophila* neurogenic gene *mastermind* is widely expressed and associates with specific chromosomal regions.** *Genetics* 1996, **143:**859.

347. Schmid AT, Tinley TL, Yedvobnick B: **Transcription of the neurogenic gene *mastermind* during *Drosophila* development.** *J Exp Zool* 1996, **274:**207.

348. Cummings CA, Cronmiller C: **The *daughterless* gene functions together with *Notch* and *Delta* in the control of ovarian follicle development in *Drosophila*.** *Development* 1994, **120:**381.

349. Boulianne GL, de la Concha A, Campos-Ortega JA, Jan LY, Jan YN: **The *Drosophila* neurogenic gene *neuralized* encodes a novel protein and is expressed in precursors of larval and adult neurons.** *EMBO J* 1991, **10:**2975-2983.

350. Larkin MK, Deng WM, Holder K, Tworoger M, Clegg N, Ruohola-Baker H: **Role of Notch pathway in terminal follicle cell differentiation during *Drosophila* oogenesis.** *Dev Genes Evol* 1999, **209:**301-311.

351. Bender LB, Kooh PJ, Muskavitch MA: **Complex function and expression of *Delta* during *Drosophila* oogenesis.** *Genetics* 1993, **133:**967.

352. Ye Y, Fortini ME: **Characterization of *Drosophila* Presenilin and its colocalization with Notch during development.** *Mech Dev* 1998, **79:**199-211.

353. Boulianne GL, Livne-Bar I, Humphreys JM, Liang Y, Lin C, Rogaev E, St. George-Hyslop P: **Cloning and characterization of the *Drosophila presenilin* homologue.** *Neuroreport* 1997, **8:**1025-1029.

354. López-Schier H, Johnston DS: ***Drosophila* Nicastrin is essential for the intramembranous cleavage of Notch.** *Dev Cell* 2002, **2:**79-89.

355. Hu Y, Fortini ME: **Different cofactor activities in gamma-secretase assembly: evidence for a nicastrin-Aph-1 subcomplex.** *J Cell Biol* 2003, **161:**685-690.

356. Majumdar A, Nagaraj R, Banerjee U: ***strawberry notch* encodes a conserved nuclear protein that functions downstream of *Notch* and regulates gene expression along the developing wing margin of *Drosophila*.** *Genes Dev* 1997, **11:**1341.

357. Blochlinger K, Jan LY, Jan YN: **Postembryonic patterns of expression of *cut*, a locus regulating sensory organ identity in *Drosophila*.** *Development* 1993, **117:**441-450.

358. Jackson SM, Blochlinger K: ***cut* interacts with Notch and protein kinase A to regulate egg chamber formation and to maintain germline cyst integrity during *Drosophila* oogenesis.** *Development* 1997, **124:**3663-3672.

359. Zhao D, Clyde D, Bownes M: **Expression of fringe is down regulated by Gurken/Epidermal growth factor receptor signalling and is required for the morphogenesis of ovarian follicle cells.** *J Cell Sci* 2000, **113:**3781-3794.

360. Hsu T, McRackan D, Vincent TS, Gert De Couet H: ***Drosophila* Pin1 prolyl isomerase Dodo is a MAP kinase signal responder during oogenesis.** *Nat Cell Biol* 2001, **3:**538.

361. Zartman JJ, Cheung LS, Niepielko MG, Bonini C, Haley B, Yakoby N, Shvartsman SY: **Pattern formation by a moving morphogen source.** *Phys Biol* 2011, **8:**045003.

362. Huang RY, Orr WC: **Broad-complex function during oogenesis in *Drosophila melanogaster*.** *Dev Genet* 1992, **13:**277-288.

363. Schulz RA, The SM, Hogue DA, Galewsky S, Guo Q: ***Ets* oncogene-related gene *Elg* functions in *Drosophila* oogenesis.** *Proceedings of the National Academy of Sciences* 1993, **90:**10076.

364. Gajewski KM, Schulz RA: **Requirement of the ETS domain transcription factor D-ELG for egg chamber patterning and development during *Drosophila* oogenesis.** *Oncogene* 1995, **11:**1033.

365. Schober M, Rebay I, Perrimon N: **Function of the ETS transcription factor Yan in border cell migration.** *Development* 2005, **132:**3493-3504.

366. Morimoto AM, Jordan KC, Tietze K, Britton JS, O'Neill EM, Ruohola-Baker H: **Pointed, an ETS domain transcription factor, negatively regulates the EGF receptor pathway in *Drosophila* oogenesis.** *Development* 1996, **122:**3745-3754.

367. Deng W-M, Schneider M, Frock R, Castillejo-Lopez C, Gaman EA, Baumgartner S, Ruohola-Baker H: **Dystroglycan is required for polarizing the epithelial cells and the oocyte in *Drosophila*.** *Development* 2003, **130:**173-184.

368. Tanentzapf G, Smith C, McGlade J, Tepass U: **Apical, lateral, and basal polarization cues contribute to the development of the follicular epithelium during *Drosophila* oogenesis.** *J Cell Biol* 2000, **151:**891.

369. Sokol NS, Cooley L: ***Drosophila* filamin is required for follicle cell motility during oogenesis.** *Dev Biol* 2003, **260:**260.

370. Wang X, Bo J, Bridges T, Dugan KD, Pan TC, Chodosh LA, Montell DJ: **Analysis of cell migration using whole-genome expression profiling of migratory cells in the *Drosophila* ovary.** *Dev Cell* 2006, **10:**483-495.

371. Frydman HM, Spradling AC: **The receptor-like tyrosine phosphatase Lar is required for epithelial planar polarity and for axis determination within *Drosophila* ovarian follicles.** *Development* 2001, **128:**3209.

372. Goode S, Perrimon N: **Inhibition of patterned cell shape change and cell invasion by Discs large during *Drosophila* oogenesis.** *Genes Dev* 1997, **11:**2532-2544.

373. Bilder D, Perrimon N: **Localization of apical epithelial determinants by the basolateral PDZ protein Scribble.** *Nature* 2000, **403:**676.

374. Paterson J, O'Hare K: **Structure and transcription of the *singed* Locus of *Drosophila melanogaster*.** *Genetics* 1991, **129:**1073.

375. Montell DJ, Rørth P, Spradling AC: ***slow border cells,* a locus required for a developmentally regulated cell migration during oogenesis, encodes *Drosophila* C/EBP.** *Cell* 1992, **71:**51-62.

376. Sourmeli S, Papantonis A, Lecanidou R: **A novel role for the *Bombyx* Slbo homologue, BmC/EBP, in insect choriogenesis.** *Biochem Biophys Res Commun* 2005, **337:**713-719.

377. Shravage BV, Altmann G, Technau M, Roth S: **The role of Dpp and its inhibitors during eggshell patterning in *Drosophila*.** *Development* 2007, **134:**2261-2271.

378. Chen Y, Schüpbach T: **The role of brinker in eggshell patterning.** *Mech Dev* 2006, **123:**395-406.

379. Queenan AM, Ghabrial A, Schüpbach T: **Ectopic activation of *torpedo/Egfr,* a *Drosophila* receptor tyrosine kinase, dorsalizes both the eggshell and the embryo.** *Development* 1997, **124:**3871-3880.

380. Wasserman JD, Freeman M: **An autoregulatory cascade of EGF receptor signaling patterns the *Drosophila* egg.** *Cell* 1998, **95:**355-364.

381. Ruohola-Baker H, Greil E, Chou TB, Baker D, Jan LY, Jan YN: **Spatially localized rhomboid is required for establishment of the dorsal-ventral axis in *Drosophila* oogenesis.** *Cell* 1993, **73:**953.

382. Steen PW, Tian S, Tully SE, Cravatt BF, LeMosy EK: **Activation of Snake in a serine protease cascade that defines the dorsoventral axis is atypical and *pipe*-independent in *Drosophila* embryos.** *FEBS Lett* 2010, **584:**3557-3560.

383. Chasan R, Jin Y, Anderson KV: **Activation of the easter zymogen is regulated by five other genes to define dorsal-ventral polarity in the *Drosophila* embryo.** *Development* 1992, **115:**607-616.

384. Ghiglione C, Carraway KL, Amundadottir LT, Boswell RE, Perrimon N, Duffy JB: **The transmembrane molecule kekkon 1 acts in a feedback loop to negatively regulate the activity of the *Drosophila* EGF receptor during oogenesis.** *Cell* 1999, **96:**847-856.

385. Kleve CD, Siler DA, Syed SK, Eldon ED: **Expression of *18-wheeler* in the follicle cell epithelium affects cell migration and egg morphology in *Drosophila*.** *Dev Dyn* 2006, **235:**1953-1961.

386. Sapir A, Schweitzer R, Shilo BZ: **Sequential activation of the EGF receptor pathway during *Drosophila* oogenesis establishes the dorsoventral axis.** *Development* 1998, **125:**191-200.

387. Bohrmann J, Zimmermann J: **Gap junctions in the ovary of *Drosophila melanogaster:* localization of innexins 1, 2, 3 and 4 and evidence for intercellular communication via innexin-2 containing channels.** *BMC Dev Biol* 2008, **8:**111.

388. Mukai M, Kato H, Hira S, Nakamura K, Kita H, Kobayashi S: **Innexin2 gap junctions in somatic support cells are required for cyst formation and for egg chamber formation in *Drosophila*.** *Mech Dev* 2011, **128:**510-523.

389. Tepass U, Knust E: **Phenotypic and developmental analysis of mutations at the *crumbs* locus, a gene required for the development of epithelia in *Drosophila melanogaster*.** *Roux's Arch Dev Biol* 1990, **199:**189.

390. Neuman-Silberberg FS: ***Drosophila* female sterile mutation *spoonbill* interferes with multiple pathways in oogenesis.** *Genesis* 2007, **45:**369-381.

391. Motola S, Neuman-Silberberg FS: ***spoonbill,* a new *Drosophila* female-sterile mutation, interferes with chromosome organization and dorsal-ventral patterning of the egg.** *Dev Dyn* 2004, **230:**535-545.

392. Hadad M, Bresler-Musikant T, Neuman-Silberberg FS: ***Drosophila spoonbill* encodes a dual-specificity A-kinase anchor protein essential for oogenesis.** *Mech Dev* 2011, **128:**471-482.

393. Fichelson P, Jagut M, Lepanse S, Lepesant JA, Huynh JR: ***lethal giant larvae* is required with the *par* genes for the early polarization of the *Drosophila* oocyte.** *Development* 2010, **137:**815-824.

394. Sevrioukov EA, He JP, Moghrabi N, Sunio A, Kramer H: **A role for the *deep orange* and *carnation* eye color genes in lysosomal delivery in *Drosophila*.** *Mol Cell* 1999, **4:**479-486.

395. Szabo K, Jekely G, Rørth P: **Cloning and expression of *sprint,* a *Drosophila* homologue of RIN1.** *Mech Dev* 2001, **101:**259-262.

396. Wahlstrom G, Vartiainen M, Yamamoto L, Mattila PK, Lappalainen P, Heino TI: **Twinfilin is required for actin-dependent developmental processes in *Drosophila*.** *J Cell Biol* 2001, **155:**787-795.

397. Grammont M, Dastugue B, Couderc JL: **The *Drosophila toucan* (*toc*) gene is required in germline cells for the somatic cell patterning during oogenesis.** *Development* 1997, **124:**4917-4926.

398. Jang AC, Chang YC, Bai J, Montell D: **Border-cell migration requires integration of spatial and temporal signals by the BTB protein Abrupt.** *Nat Cell Biol* 2009, **11:**569-579.

399. McDonald JA, Pinheiro EM, Montell DJ: **PVF1, a PDGF/VEGF homolog, is sufficient to guide border cells and interacts genetically with Taiman.** *Development* 2003, **130:**3469-3478.

400. Bai J, Uehara Y, Montell DJ: **Regulation of invasive cell behavior by taiman, a *Drosophila* protein related to AIB1, a steroid receptor coactivator amplified in breast cancer.** *Cell* 2000, **103:**1047-1058.

401. Li M, Mead EA, Zhu J: **Heterodimer of two bHLH-PAS proteins mediates juvenile hormone-induced gene expression.** *Proceedings of the National Academy of Sciences* 2011, **108:**638-643.

402. Charles J-P, Iwema T, Epa VC, Takaki K, Rynes J, Jindra M: **Ligand-binding properties of a juvenile hormone receptor, Methoprene-tolerant.** *Proceedings of the National Academy of Sciences* 2011, **108:**21128-21133.

403. Mathieu J, Sung HH, Pugieux C, Soetaert J, Rørth P: **A sensitized PiggyBac-based screen for regulators of border cell migration in *Drosophila*.** *Genetics* 2007, **176:**1579-1590.

404. Treisman JE, Ito N, Rubin GM: ***misshapen* encodes a protein kinase involved in cell shape control in *Drosophila*.** *Gene* 1997, **186:**119-125.

405. Wakabayashi-Ito N, Belvin MP, Bluestein DA, Anderson KV: ***fusilli,* an essential gene with a maternal role in *Drosophila* embryonic dorsal-ventral patterning.** *Dev Biol* 2001, **229:**44-54.

406. Chan HYE, Brogna S, O'Kane CJ: **Dribble, the *Drosophila* KRR1p homologue, is involved in rRNA processing.** *Mol Biol Cell* 2001, **12:**1409-1419.

407. Dornier E, Coumailleau F, Ottavi JF, Moretti J, Boucheix C, Mauduit P, Schweisguth F, Rubinstein E: **TspanC8 tetraspanins regulate ADAM10/Kuzbanian trafficking and promote Notch activation in flies and mammals.** *J Cell Biol* 2012, **199:**481-496.

408. Paula Zappia M, Adriana Brocco M, Billi SC, Frasch AC, Fernanda Ceriani M: **M6 Membrane Protein Plays an Essential Role in *Drosophila* Oogenesis.** *PLoS ONE* 2011, **6**.

409. Szafranski P, Goode S: **A Fasciclin 2 morphogenetic switch organizes epithelial cell cluster polarity and motility.** *Development* 2004, **131:**2023-2036.

410. Boyle MJ, French RL, Cosand KA, Dorman JB, Kiehart DP, Berg CA: **Division of labor: Subsets of dorsal-appendage-forming cells control the shape of the entire tube.** *Dev Biol* 2010, **346:**68-79.

411. Tran DH, Berg CA: ***bullwinkle* and *shark* regulate dorsal-appendage morphogenesis in *Drosophila* oogenesis.** *Development* 2003, **130:**6273-6282.

412. Fernandez R, Takahashi F, Liu Z, Steward R, Stein D, Stanley ER: **The *Drosophila* shark tyrosine kinase is required for embryonic dorsal closure.** *Genes Dev* 2000, **14:**604-614.

413. Dorman JB, James KE, Fraser SE, Kiehart DP, Berg CA: ***bullwinkle* is required for epithelial morphogenesis during *Drosophila* oogenesis.** *Dev Biol* 2004, **267:**320-341.

414. Zartman JJ, Yakoby N, Bristow CA, Zhou X, Schlichting K, Dahmann C, Shvartsman SY: **Cad74A is regulated by BR and is required for robust dorsal appendage formation in *Drosophila* oogenesis.** *Dev Biol* 2008, **322:**289-301.

415. Pathirana S, Zhao D, Bownes M: **The *Drosophila* RGS protein Loco is required for dorsal/ventral axis formation of the egg and embryo, and nurse cell dumping.** *Mech Dev* 2001, **109:**137-150.

416. Schnorr JD, Holdcraft R, Chevalier B, Berg CA: ***Ras1* interacts with multiple new signaling and cytoskeletal loci in *Drosophila* eggshell patterning and morphogenesis.** *Genetics* 2001, **159:**609-622.

417. Denef N, Chen Y, Weeks SD, Barcelo G, Schüpbach T: **Crag regulates epithelial architecture and polarized deposition of basement membrane proteins in *Drosophila*.** *Dev Cell* 2008, **14:**354-364.

418. Cassill JA, Whitney M, Joazeiro CA, Becker A, Zuker CS: **Isolation of *Drosophila* genes encoding G protein-coupled receptor kinases.** *Proceedings of the National Academy of Sciences* 1991, **88:**11067-11070.

419. Lannutti BJ, Schneider LE: **Gprk2 Controls cAMP Levels in *Drosophila* Development.** *Dev Biol* 2001, **233:**174-185.

420. Suzanne M, Perrimon N, Noselli S: **The *Drosophila* JNK pathway controls the morphogenesis of the egg dorsal appendages and micropyle.** *Dev Biol* 2001, **237:**282-294.

421. Somogyi K, Rørth P: **Evidence for tension-based regulation of *Drosophila* MAL and SRF during invasive cell migration.** *Dev Cell* 2004, **7:**85-93.

422. Paululat A, Goubeaud A, Damm C, Knirr S, Burchard S, Renkawitz-Pohl R: **The mesodermal expression of *rolling stone* (*rost*) is essential for myoblast fusion in *Drosophila* and encodes a potential transmembrane protein.** *J Cell Biol* 1997, **138:**337-348.

423. Liu Y, Montell DJ: ***jing:* a downstream target of *slbo* required for developmental control of border cell migration.** *Development* 2001, **128:**321-330.

424. Price MD, Lai Z: **The *yan* gene is highly conserved in *Drosophila* and its expression suggests a complex role throughout development.** *Dev Genes Evol* 1999, **209:**207-217.

425. Myster SH, Cavallo R, Anderson CT, Fox DT, Peifer M: ***Drosophila* p120catenin plays a supporting role in cell adhesion but is not an essential adherens junction component.** *J Cell Biol* 2003, **160:**433-449.

426. Wolfgang WJ, Quan F, Thambi N, Forte M: **Restricted spatial and temporal expression of G-protein alpha subunits during *Drosophila* embryogenesis.** *Development* 1991, **113:**527-538.

427. Frolov MV, Benevolenskaya EV, Birchler JA: **Molecular analysis of a novel *Drosophila* diacylglycerol kinase, *DGKe*.** *Biochim Biophys Acta* 2001, **1538:**339-352.

428. Bohrmann J: **Antisera against a channel-forming 16 kda protein inhibit dye-coupling and bind to cell membranes in *Drosophila* ovarian follicles.** *J Cell Sci* 1993, **105:**513-518.

429. Dansereau DA, Lasko P: **RanBPM regulates cell shape, arrangement, and capacity of the female germline stem cell niche in *Drosophila melanogaster*.** *J Cell Biol* 2008, **182:**963-977.

430. Nystul T, Spradling A: **An epithelial niche in the *Drosophila* ovary undergoes long-range stem cell replacement.** *Cell Stem Cell* 2007, **1:**277-285.

431. Cernilogar FM, Fabbri F, Andrenacci D, Taddei C, Gargiulo G: ***Drosophila* vitelline membrane cross-linking requires the *fs(1)Nasrat, fs(1)polehole* and chorion genes activities.** *Dev Genes Evol* 2001, **211:**573-580.

432. de Cuevas M, Spradling AC: **Morphogenesis of the *Drosophila* fusome and its implications for oocyte specification.** *Development* 1998, **125:**2781-2789.

433. Perkins LA, Larsen I, Perrimon N: ***corkscrew* encodes a putative protein tyrosine phosphatase that functions to transduce the terminal signal from the receptor tyrosine kinase torso.** *Cell* 1992, **70:**225-236.

434. Perkins LA, Johnson MR, Melnick MB, Perrimon N: **The nonreceptor protein tyrosine phosphatase corkscrew functions in multiple receptor tyrosine kinase pathways in *Drosophila*.** *Dev Biol* 1996, **180:**63-81.

435. Shandala T, Kortschak RD, Saint R: **The *Drosophila retained/dead ringer* gene and ARID gene family function during development.** *Int J Dev Biol* 2002, **46:**423-430.

436. Shandala T, Kortschak RD, Gregory S, Saint R: **The *Drosophila dead ringer* gene is required for early embryonic patterning through regulation of *argos* and *buttonhead* expression.** *Development* 1999, **126:**4341-4349.

437. Savant-Bhonsale S, Montell DJ: ***torso-like* encodes the localized determinant of *Drosophila* terminal pattern formation.** *Genes Dev* 1993, **7:**2548-2555.

438. Stevens LM, Frohnhofer HG, Klingler M, Nüsslein-Volhard C: **Localized requirement for *torso-like* expression in follicle cells for development of terminal anlagen of the *Drosophila* embryo.** *Nature* 1990, **346:**660-663.

439. Klingler M, Erdelyi M, Szabad J, Nüsslein-Volhard C: **Function of *torso* in determining the terminal anlagen of the *Drosophila* embryo.** *Nature* 1988, **335:**275-277.

440. Schüpbach T, Wieschaus E: **Germline autonomy of maternal-effect mutations altering the embryonic body pattern of *Drosophila*.** *Dev Biol* 1986, **113:**443-448.

441. Florence BL, Faller DV: ***Drosophila* female sterile (1) homeotic is a multifunctional transcriptional regulator that is modulated by Ras signaling.** *Dev Dyn* 2008, **237:**554-564.

442. Schnorr JD, Berg CA: **Differential activity of *Ras1* during patterning of the *Drosophila* dorsoventral axis.** *Genetics* 1996, **144:**1545-1557.

443. Ambrosio L, Mahowald AP, Perrimon N: **Requirement of the *Drosophila raf* homologue for *torso* function.** *Nature* 1989, **342:**288-291.

444. Xi R, McGregor JR, Harrison DA: **A Gradient of JAK pathway activity patterns the anterior-posterior axis of the follicular epithelium.** *Dev Cell* 2003, **4:**167-177.

445. Li J, Xia F, Li WX: **Coactivation of STAT and Ras is required for germ cell proliferation and invasive migration in *Drosophila*.** *Dev Cell* 2003, **5:**787-798.

446. Hsu JC, Perrimon N: **A temperature-sensitive MEK mutation demonstrates the conservation of the signaling pathways activated by receptor tyrosine kinases.** *Genes Dev* 1994, **8:**2176-2187.

447. Peretz G, Bakhrat A, Abdu U: **Expression of the *Drosophila melanogaster* GADD45 homolog (CG11086) affects egg asymmetric development that is mediated by the c-Jun N-terminal kinase pathway.** *Genetics* 2007, **177:**1691-1702.

448. Anne J, Mechler BM: **Valois, a component of the nuage and pole plasm, is involved in assembly of these structures, and binds to Tudor and the methyltransferase Capsuléen.** *Development* 2005, **132:**2167-2177.

449. Creed TM, Loganathan SN, Varonin D, Jackson CA, Arkov AL: **Novel role of specific Tudor domains in Tudor-Aubergine protein complex assembly and distribution during *Drosophila* oogenesis.** *Biochem Biophys Res Commun* 2010, **402:**384-389.

450. Pane A, Wehr K, Schüpbach T: ***zucchini* and *squash* encode two putative nucleases required for rasiRNA production in the *Drosophila* germline.** *Dev Cell* 2007, **12:**851-862.

451. Harris AN, Macdonald PM: ***aubergine* encodes a *Drosophila* polar granule component required for pole cell formation and related to eIF2C.** *Development* 2001, **128:**2823-2832.

452. Johnstone O, Deuring R, Bock R, Linder P, Fuller MT, Lasko P: **Belle is a *Drosophila* DEAD-box protein required for viability and in the germ line.** *Dev Biol* 2005, **277:**92-101.

453. Kibanov MV, Egorova KS, Ryazansky SS, Sokolova OA, Kotov AA, Olenkina OM, Stolyarenko AD, Gvozdev VA, Olenina LV: **A novel**

organelle, the piNG-body, in the nuage of *Drosophila* male germ cells is associated with piRNA-mediated gene silencing. *Mol Biol Cell* 2011, **22:**3410-3419.

454. Chen Y, Pane A, Schüpbach T: ***cutoff* and *aubergine* mutations result in retrotransposon upregulation and checkpoint activation in *Drosophila*.** *Curr Biol* 2007, **17:**637-642.

455. Kawaoka S, Hayashi N, Suzuki Y, Abe H, Sugano S, Tomari Y, Shimada T, Katsuma S: **The *Bombyx* ovary-derived cell line endogenously expresses PIWI/PIWI-interacting RNA complexes.** *RNA* 2009.

456. Malone CD, Brennecke J, Dus M, Stark A, McCombie WR, Sachidanandam R, Hannon GJ: **Specialized piRNA pathways act in germline and somatic tissues of the *Drosophila* ovary.** *Cell* 2009, **137:**522-535.

457. Anne J: **Targeting and anchoring Tudor in the pole plasm of the *Drosophila* oocyte.** *PLoS ONE* 2010, **5:**e14362.

458. Anne J: **C-terminal moiety of Tudor contains its in vivo activity in *Drosophila*.** *PLoS ONE* 2010, **5:**e14378.

459. Bardsley A, McDonald K, Boswell RE: **Distribution of tudor protein in the *Drosophila* embryo suggests separation of functions based on site of localization.** *Development* 1993, **119:**207-219.

460. Lim AK, Kai T: **Unique germ-line organelle, nuage, functions to repress selfish genetic elements in *Drosophila melanogaster*.** *Proceedings of the National Academy of Sciences* 2007, **104:**6714-6719.

461. Patil VS, Kai T: **Repression of retroelements in *Drosophila* germline via piRNA pathway by the tudor domain protein tejas.** *Curr Biol* 2010, **20:**724-730.

462. Gillespie DE, Berg CA: ***homeless* is required for RNA localization in *Drosophila* oogenesis and encodes a new member of the DE-H family of RNA-dependent ATPases.** *Genes Dev* 1995, **9:**2495-2508.

463. Friberg A, Corsini L, Mourão A, Sattler M: **Structure and ligand binding of the extended Tudor domain of *D. melanogaster* Tudor-SN.** *J Mol Biol* 2009, **387:**921.

464. Szakmary A, Reedy M, Qi H, Lin H: **The Yb protein defines a novel organelle and regulates male germline stem cell self-renewal in *Drosophila melanogaster*.** *J Cell Biol* 2009, **185:**613-627.

465. Rehwinkel J, Letunic I, Raes J, Bork P, Izaurralde E: **Nonsense-mediated mRNA decay factors act in concert to regulate common mRNA targets.** *RNA* 2005, **11:**1530-1544.

466. Eulalio A, Behm-Ansmant I, Izaurralde E: **P bodies: at the crossroads of post-transcriptional pathways.** *Nat Rev Mol Cell Biol* 2007, **8:**9-22.

467. Lin MD, Jiao X, Grima D, Newbury SF, Kiledjian M, Chou TB: ***Drosophila* processing bodies in oogenesis.** *Dev Biol* 2008, **322:**276-288.

468. Lin MD, Fan SJ, Hsu WS, Chou TB: ***Drosophila* decapping protein 1, dDcp1, is a component of the *oskar* mRNP complex and directs its posterior localization in the oocyte.** *Dev Cell* 2006, **10:**601-613.

469. Till DD, Linz B, Seago JE, Elgar SJ, Marujo PE, de L. Elias M, Arraiano CM, McClellan JA, McCarthy JEG, Newbury SF: **Identification and developmental expression of a 5'-3' exoribonuclease from *Drosophila melanogaster*.** *Mech Dev* 1998, **79:**51-55.

470. Fan S-J, Marchand V, Ephrussi A: ***Drosophila* Ge-1 promotes P Body formation and *oskar* mRNA localization.** *PLoS ONE* 2011, **6:**e20612.

471. Lie YS, Macdonald PM: **Apontic binds the translational repressor Bruno and is implicated in regulation of *oskar* mRNA translation.** *Development* 1999, **126:**1129-1138.

472. Zhao G, Chen K, Yao Q, Wang W, Wang Y, Mu R, Chen H, Yang H, Zhou H: **The *nanos* gene of *Bombyx mori* and its expression patterns in developmental embryos and larvae tissues.** *Gene Expr Patterns* 2008, **8:**254-260.

473. Nakao H, Matsumoto T, Oba Y, Niimi T, Yaginuma T: **Germ cell specification and early embryonic patterning in *Bombyx mori* as revealed by *nanos* orthologues.** *Evol Dev* 2008, **10:**546-554.

474. Kobayashi S, Yamada M, Asaoka M, Kitamura T: **Essential role of the posterior morphogen nanos for germline development in *Drosophila*.** *Nature* 1996, **380:**708-711.

475. Nüsslein-Volhard C, Frohnhofer HG, Lehmann R: **Determination of anteroposterior polarity in *Drosophila*.** *Science* 1987, **238:**1675-1681.

476. Schmitt-Engel C, Cerny AC, Schoppmeier M: **A dual role for *nanos* and *pumilio* in anterior and posterior blastodermal patterning of the short-germ beetle *Tribolium castaneum*.** *Dev Biol* 2012, **364:**224-235.

477. Nakao H: **Anterior and posterior centers jointly regulate *Bombyx* embryo body segmentation.** *Dev Biol* 2012, **371:**293-301.

478. Cook HA, Koppetsch BS, Wu J, Theurkauf WE: **The *Drosophila* SDE3 homolog *armitage* is required for *oskar* mRNA silencing and embryonic axis specification.** *Cell* 2004, **116:**817-829.

479. Kim-Ha J, Kerr K, Macdonald PM: **Translational regulation of *oskar* mRNA by Bruno, an ovarian RNA-binding protein, is essential.** *Cell* 1995, **81:**403-412.

480. Moore J, Han H, Lasko P: **Bruno negatively regulates *germ cell-less* expression in a BRE-independent manner.** *Mech Dev* 2009, **126:**503-516.

481. Webster PJ, Liang L, Berg CA, Lasko P, Macdonald PM: **Translational repressor *bruno* plays multiple roles in development and is widely conserved.** *Genes Dev* 1997, **11:**2510-2521.

482. Sugimura I, Lilly MA: **Bruno inhibits the expression of mitotic cyclins during the prophase I meiotic arrest of *Drosophila* oocytes.** *Dev Cell* 2006, **10:**127-135.

483. Suyama R, Jenny A, Curado S, Pellis-van Berkel W, Ephrussi A: **The actin-binding protein Lasp promotes Oskar accumulation at the posterior pole of the *Drosophila* embryo.** *Development* 2009, **136:**95-105.

484. Lynch JA, Ozuak O, Khila A, Abouheif E, Desplan C, Roth S: **The phylogenetic origin of *oskar* coincided with the origin of maternally provisioned germ plasm and pole cells at the base of the Holometabola.** *PLoS Genet* 2011, **7:**e1002029.

485. Lehmann R, Nüsslein-Volhard C: **Abdominal segmentation, pole cell formation, and embryonic polarity require the localized activity of *oskar*, a maternal gene in *Drosophila*.** *Cell* 1986, **47:**141-152.

486. Ewen-Campen B, Srouji JR, Schwager EE, Extavour CG: ***oskar* predates the evolution of germ plasm in insects.** *Curr Biol* 2012, **22:**2278-2283.

487. van Eeden FJM, Palacios IM, Petronczki M, Weston MJD, St. Johnston D: **Barentsz is essential for the posterior localization of *oskar* mRNA and colocalizes with it to the posterior pole.** *J Cell Biol* 2001, **154:**511-524.

488. Palacios IM, Gatfield D, St. Johnston D, Izaurralde E: **An eIF4AIII-containing complex required for mRNA localization and nonsense-mediated mRNA decay.** *Nature* 2004, **427:**753-757.

489. Irion U, Adams J, Chang CW, St Johnston D: **Miranda couples *oskar* mRNA/Staufen complexes to the *bicoid* mRNA localization pathway.** *Dev Biol* 2006, **297:**522-533.

490. Zimyanin VL, Belaya K, Pecreaux J, Gilchrist MJ, Clark A, Davis I, St Johnston D: **In vivo imaging of *oskar* mRNA transport reveals the mechanism of posterior localization.** *Cell* 2008, **134:**843-853.

491. Jankovics F, Sinka R, Lukacsovich T, Erdelyi M: **Moesin crosslinks actin and cell membrane in *Drosophila* oocytes and is required for Oskar anchoring.** *Curr Biol* 2002, **12:**2060-2065.

492. Zaessinger S, Busseau I, Simonelig M: **Oskar allows *nanos* mRNA translation in *Drosophila* embryos by preventing its deadenylation by Smaug/CCR4.** *Development* 2006, **133:**4573-4583.

493. Vazquez-Pianzola P, Urlaub H, Suter B: **Pabp binds to the *osk* 3′UTR and specifically contributes to *osk* mRNA stability and oocyte accumulation.** *Dev Biol* 2011, **357:**404-418.

494. Pisa V, Cozzolino M, Gargiulo S, Ottone C, Piccioni F, Monti M, Gigliotti S, Talamo F, Graziani F, Pucci P, Verrotti AC: **The molecular chaperone Hsp90 is a component of the cap-binding complex and interacts with the**

translational repressor Cup during *Drosophila* oogenesis. *Gene* 2009, **432:**67-74.

495. Piccioni F, Ottone C, Brescia P, Pisa V, Siciliano G, Galasso A, Gigliotti S, Graziani F, Verrotti AC: **The translational repressor Cup associates with the adaptor protein Miranda and the mRNA carrier Staufen at multiple time-points during *Drosophila* oogenesis.** *Gene* 2009, **428:**47-52.

496. Lewandowski JP, Sheehan KB, Bennett Jr PE, Boswell RE: **Mago Nashi, Tsunagi/Y14, and Ranshi form a complex that influences oocyte differentiation in *Drosophila melanogaster*.** *Dev Biol* 2010, **339:**307-319.

497. Andrews S, Snowflack DR, Clark IE, Gavis ER: **Multiple mechanisms collaborate to repress nanos translation in the *Drosophila* ovary and embryo.** *RNA* 2011, **17:**967-977.

498. Temme C, Zhang L, Kremmer E, Ihling C, Chartier A, Sinz A, Simonelig M, Wahle E: **Subunits of the *Drosophila* CCR4-NOT complex and their roles in mRNA deadenylation.** *RNA* 2010, **16:**1356-1370.

499. Chicoine J, Benoit P, Gamberi C, Paliouras M, Simonelig M, Lasko P: **Bicaudal-C recruits CCR4-NOT deadenylase to target mRNAs and regulates oogenesis, cytoskeletal organization, and its own expression.** *Dev Cell* 2007, **13:**691-704.

500. Juge F, Zaessinger S, Temme C, Wahle E, Simonelig M: **Control of poly(A) polymerase level is essential to cytoplasmic polyadenylation and early development in *Drosophila*.** *EMBO J* 2002, **21:**6603-6613.

501. Tanaka T, Nakamura A: **The endocytic pathway acts downstream of Oskar in *Drosophila* germ plasm assembly.** *Development* 2008, **135:**1107-1117.

502. Nakamura A, Amikura R, Hanyu K, Kobayashi S: **Me31B silences translation of oocyte-localizing RNAs through the formation of cytoplasmic RNP complex during *Drosophila* oogenesis.** *Development* 2001, **128:**3233-3242.

503. Fischer-Vize JA, Rubin GM, Lehmann R: **The *fat facets* gene is required for *Drosophila* eye and embryo development.** *Development* 1992, **116:**985-1000.

504. Besse F, López de Quinto S, Marchand V, Trucco A, Ephrussi A: ***Drosophila* PTB promotes formation of high-order RNP particles and represses *oskar* translation.** *Genes Dev* 2009, **23:**195-207.

505. Jankovics F, Sinka R, Erdelyi M: **An interaction type of genetic screen reveals a role of the *Rab11* gene in *oskar* mRNA localization in the developing *Drosophila melanogaster* oocyte.** *Genetics* 2001, **158:**1177-1188.

506. Compagnon J, Gervais L, Roman MS, Chamot-Boeuf S, Guichet A: **Interplay between Rab5 and PtdIns(4,5)P2 controls early endocytosis in the *Drosophila* germline.** *J Cell Sci* 2009, **122:**25-35.

507. Hassan BA, Prokopenko SN, Breuer S, Zhang B, Paululat A, Bellen HJ: ***skittles,* a *Drosophila* phosphatidylinositol 4-phosphate 5-Kinase, is required for cell viability, germline development and bristle morphology, but not for neurotransmitter release.** *Genetics* 1998, **150:**1527-1537.

508. Chen F, Barkett M, Ram KT, Quintanilla A, Hariharan IK: **Biological characterization of *Drosophila* Rapgap1, a GTPase activating protein for Rap1.** *Proceedings of the National Academy of Sciences* 1997, **94:**12485-12490.

509. Faulkner DL, Dockendorff TC, Jongens TA: **Clonal analysis of *cmp44E*, which encodes a conserved putative transmembrane protein, indicates a requirement for cell viability in *Drosophila*.** *Dev Genet* 1998, **23:**264-274.

510. Jongens TA, Hay B, Jan LY, Jan YN: **The *germ cell-less* gene product: a posteriorly localized component necessary for germ cell development in *Drosophila*.** *Cell* 1992, **70:**569-584.

511. Szuperak M, Zvara A, Erdelyi M: **Identification of germ plasm-enriched mRNAs in *Drosophila melanogaster* by the cDNA microarray technique.** *Gene Expr Patterns* 2005, **5:**717-723.

512. Lo PCH, Frasch M: **Sequence and expression of *myoglianin*, a novel *Drosophila* gene of the TGF- superfamily.** *Mech Dev* 1999, **86:**171-175.

513. Kashikawa M, Amikura R, Nakamura A, Kobayashi S: **Mitochondrial small ribosomal RNA is present on polar granules in early cleavage embryos of *Drosophila melanogaster*.** *Dev Growth Differ* 1999, **41:**495-502.

514. Frohnhoefer HG, Nüsslein-Volhard C: **Organization of anterior pattern in the *Drosophila* embryo by the maternal gene *bicoid*.** *Nature* 1986, **324:**120-125.

515. Berleth T, Burri M, Thoma G, Bopp D, Richstein S, Frigerio G, Noll M, Nüsslein-Volhard C: **The role of localization of *bicoid* RNA in organizing the anterior pattern of the *Drosophila* embryo.** *EMBO J* 1988, **7:**1749-1756.

516. St Johnston D, Driever W, Berleth T, Richstein S, Nüsslein-Volhard C: **Multiple steps in the localization of *bicoid* RNA to the anterior pole of the *Drosophila* oocyte.** *Development* 1989, **107 Suppl:**13-19.

517. Wang S, Hazelrigg T: **Implications for *bcd* mRNA localization from spatial distribution of *exu* protein in *Drosophila* oogenesis.** *Nature* 1994, **369:**400-403.

518. Lynch J, Desplan C: **Evolution of development: beyond Bicoid.** *Curr Biol* 2003, **13:**R557-559.

519. Weil TT, Xanthakis D, Parton R, Dobbie I, Rabouille C, Gavis ER, Davis I: **Distinguishing direct from indirect roles for *bicoid* mRNA localization factors.** *Development* 2010, **137:**169-176.

520. He F, Ren J, Wang W, Ma J: **Evaluating the *Drosophila* Bicoid morphogen gradient system through dissecting the noise in transcriptional bursts.** *Bioinformatics* 2012, **28:**970-975.

521. Schoppmeier M, Fischer S, Schmitt-Engel C, Lˆhr U, Klingler M: **An ancient anterior patterning system promotes *caudal* repression and head formation in Ecdysozoa.** *Curr Biol* 2009, **19:**1811-1815.

522. Lynch JA, Brent AE, Leaf DS, Pultz MA, Desplan C: **Localized maternal *orthodenticle* patterns anterior and posterior in the long germ wasp *Nasonia*.** *Nature* 2006, **439:**728-732.

523. Wilson MJ, Dearden PK: **Diversity in insect axis formation: two *orthodenticle* genes and *hunchback* act in anterior patterning and influence dorsoventral organization in the honeybee (*Apis mellifera*).** *Development* 2011, **138:**3497-3507.

524. McGregor AP: **Wasps, beetles and the beginning of the ends.** *Bioessays* 2006, **28:**683-686.

525. Rosenberg MI, Lynch JA, Desplan C: **Heads and tails: evolution of antero-posterior patterning in insects.** *Biochim Biophys Acta* 2009, **1789:**333-342.

526. MacDonald PM, Luk SK, Kilpatrick M: **Protein encoded by the *exuperantia* gene is concentrated at sites of *bicoid* mRNA accumulation in *Drosophila* nurse cells but not in oocytes or embryos.** *Genes Dev* 1991, **5:**2455-2466.

527. Schnorrer F, Bohmann K, Nüsslein-Volhard C: **The molecular motor dynein is involved in targeting *swallow* and *bicoid* RNA to the anterior pole of *Drosophila* oocytes.** *Nat Cell Biol* 2000, **2:**185-190.

528. Hegde J, Stephenson EC: **Distribution of swallow protein in egg chambers and embryos of *Drosophila melanogaster*.** *Development* 1993, **119:**457-470.

529. Stephenson EC, Chao YC, Fackenthal JD: **Molecular analysis of the *swallow* gene of *Drosophila melanogaster*.** *Genes Dev* 1988, **2:**1655-1665.

530. Mlodzik M, Fjose A, Gehring WJ: **Isolation of *caudal*, a *Drosophila* homeobox-containing gene with maternal expression, whose transcripts form a concentration gradient at the pre-blastoderm stage.** *EMBO J* 1985, **4:**2961-2969.

531. Rivera-Pomar R, Lu X, Perrimon N, Taubert H, Jaeckle H: **Activation of posterior gap gene expression in the *Drosophila* blastoderm.** *Nature* 1995, **376:**253-256.

532. Nakao H: **Characterization of *Bombyx* embryo segmentation process: expression profiles of *engrailed, even-skipped, caudal,* and *wnt1/wingless* homologues.** *J Exp Zool (Mol Dev Evol)* 2010, **314B:**224-231.

533. Xu X, Xu PX, Suzuki Y: **A maternal homeobox gene, *Bombyx caudal*, forms both mRNA and protein concentration gradients spanning anteroposterior axis during gastrulation.** *Development* 1994, **120:**277-285.

534. He Z, Cao Y, Chen B, Li T: **Expression of *hunchback* during oogenesis and embryogenesis in *Locusta migratoria manilensis* (Meyen).** *Sci China Life Sci* 2011, **54:**146-151.

535. Patel NH, Hayward DC, Lall S, Pirkl NR, DiPietro D, Ball EE: **Grasshopper *hunchback* expression reveals conserved and novel aspects of axis formation and segmentation.** *Development* 2001, **128:**3459-3472.

536. Tautz D: **Regulation of the *Drosophila* segmentation gene *hunchback* by two maternal morphogenetic centres.** *Nature* 1988, **332:**281-284.

537. Melov S, Vaughan H, Cotterill S: **Molecular characterisation of the gene for the 180 kDa subunit of the DNA polymerase-primase of *Drosophila melanogaster*.** *J Cell Sci* 1992, **102:**847-856.

538. Lacoste J, Codani-Simonart S, Best-Belpomme M, Peronnet F: **Characterization and cloning of p11, a transrepressor of *Drosophila melanogaster* retrotransposon 1731.** *Nucleic Acids Res* 1995, **23:**5073-5079.

539. Benoit B, Nemeth A, Aulner N, Kuhn U, Simonelig M, Wahle E, Bourbon HM: **The *Drosophila* poly(A)-binding protein II is ubiquitous throughout *Drosophila* development and has the same function in mRNA polyadenylation as its bovine homolog *in vitro*.** *Nucleic Acids Res* 1999, **27:**3771-3778.

540. Hsu T, King DL, Labonne C, Kafatos FC: **A *Drosophila* single-strand DNA/RNA-binding factor contains a high-mobility-group box and is enriched in the nucleolus.** *Proceedings of the National Academy of Sciences* 1993, **90:**6488.

541. Stroumbakis ND, Tolias PP: **Localized maternal and zygotic expression of the gene encoding *Drosophila* HMG D.** *Biochim Biophys Acta* 1994, **1218:**245.

542. Strodicke M, Karberg S, Korge G: ***Domina* (*Dom*), a new *Drosophila* member of the FKH/WH gene family, affects morphogenesis and is a suppressor of position-effect variegation.** *Mech Dev* 2000, **96:**67-78.

543. Krejci E, Garzino V, Mary C, Bennani N, Pradel J: ***modulo,* a new maternally expressed *Drosophila* gene encodes a DNA-binding protein with distinct acidic and basic regions.** *Nucleic Acids Res* 1989, **17:**8101-8116.

544. Eliazer S, Shalaby NA, Buszczak M: **Loss of lysine-specific demethylase 1 nonautonomously causes stem cell tumors in the *Drosophila* ovary.** *Proceedings of the National Academy of Sciences* 2011, **108:**7064-7069.

545. Yoon J, Lee KS, Park JS, Yu K, Paik SG, Kang YK: **dSETDB1 and SU(VAR)3-9 sequentially function during germline-stem cell differentiation in *Drosophila melanogaster*.** *PLoS ONE* 2008, **3:**e2234.

546. Krauss V, Reuter G: **Two genes become one. The genes encoding heterochromatin protein SU(VAR)3-9 and translation initiation factor subunit eIF-2 are joined to a dicistronic unit in holometabolic insects.** *Genetics* 2000, **156:**1157-1167.

547. Kuhfittig S, Szabad J, Schotta G, Hoffmann J, Máthé E, Reuter G: ***pitkinD,* a novel gain-of-function enhancer of position-effect variegation, affects chromatin regulation during oogenesis and early embryogenesis in *Drosophila*.** *Genetics* 2001, **157:**1227-1244.

548. Mohr SE, Boswell RE: ***Zimp* encodes a homologue of mouse *Miz1* and *PIAS3* and is an essential gene in *Drosophila melanogaster*.** *Gene* 1999, **229:**109-116.

549. Clough E, Moon W, Wang S, Smith K, Hazelrigg T: **Histone methylation is required for oogenesis in *Drosophila*.** *Development* 2007, **134:**157-165.

550. Lee KS, Yoon J, Park JS, Kang YK: ***Drosophila* G9a is implicated in germ cell development.** *Insect Mol Biol* 2010, **19:**131-139.

551. Gause M, Morcillo P, Dorsett D: **Insulation of enhancer-promoter communication by a gypsy transposon insert in the *Drosophila cut* gene: cooperation between Suppressor of Hairy-wing and Modifier of mdg4 proteins.** *Mol Cell Biol* 2001, **21:**4807-4817.

552. Kim J, Shen B, Rosen C, Dorsett D: **The DNA-binding and enhancer-blocking domains of the *Drosophila* suppressor of Hairy-wing protein.** *Mol Cell Biol* 1996, **16:**3381-3392.

553. Klug WS, Bodenstein D, King RC: **Oogenesis in the suppressor of hairy-wing mutant of *Drosophila melanogaster.* I. Phenotypic characterization and transplantation experiments.** *J Exp Zool* 1968, **167:**151-156.

554. Soshnev AA, He B, Baxley RM, Jiang N, Hart CM, Tan K, Geyer PK: **Genome-wide studies of the multi-zinc finger *Drosophila* Suppressor of Hairy-wing protein in the ovary.** *Nucleic Acids Res* 2012.

555. Bhat KM, Farkas G, Karch F, Gyurkovics H, Gausz J, Schedl P: **The GAGA factor is required in the early *Drosophila* embryo not only for transcriptional regulation but also for nuclear division.** *Development* 1996, **122:**1113-1124.

556. Elfring LK, Deuring R, McCallum CM, Peterson CL, Tamkun JW: **Identification and characterization of *Drosophila* relatives of the yeast transcriptional activator SNF2/SWI2.** *Mol Cell Biol* 1994, **14:**2225-2234.

557. Dingwall AK, Beek SJ, McCallum CM, Tamkun JW, Kalpana GV, Goff SP, Scott MP: **The *Drosophila* Snr1 and Brm proteins are related to yeast SWI/SNF proteins and are components of a large protein complex.** *Mol Biol Cell* 1995, **6:**777-791.

558. Möller A, Avila FW, Erickson JW, Jäckle H: ***Drosophila* BAP60 is an essential component of the Brahma complex, required for gene activation and repression.** *J Mol Biol* 2005, **352:**329-337.

559. Crosby MA, Miller C, Alon T, Watson KL, Verrijzer CP, Goldman-Levi R, Zak NB: **The *trithorax* group gene *moira* encodes a Brahma-associated putative chromatin-remodeling factor in *Drosophila melanogaster*.** *Mol Cell Biol* 1999, **19:**1159.

560. Rendina R, Strangi A, Avallone B, Giordano E: **Bap170, a subunit of the Drosophila PBAP chromatin remodeling complex, negatively regulates the EGFR signaling.** *Genetics* 2010, **186:**167-181.

561. Ruhf ML, Braun A, Papoulas O, Tamkun JW, Randsholt N, Meister M: **The *domino* gene of *Drosophila* encodes novel members of the SWI2/SNF2 family of DNA-dependent ATPases, which contribute to the silencing of homeotic genes.** *Development* 2001, **128:**1429-1441.

562. Shearn A, Hersperger G, Hersperger E: **Genetic analysis of two allelic temperature-sensitive mutants of *Drosophila melanogaster* both of which are zygotic and maternal-effect lethals.** *Genetics* 1978, **89:**341.

563. Lawrence PA, Johnston P, Struhl G: **Different requirements for homeotic genes in the soma and germ line of *Drosophila*.** *Cell* 1983, **35:**27-34.

564. Breen TR, Duncan IM: **Maternal expression of genes that regulate the Bithorax complex of *Drosophila melanogaster*.** *Dev Biol* 1986, **118:**442.

565. Bornemann D, Miller E, Simon J: **The *Drosophila Polycomb* group gene *Sex comb on midleg (Scm)* encodes a zinc finger protein with similarity to polyhomeotic protein.** *Development* 1996, **122:**1621-1630.

566. Docquier F, Saget O, Forquignon F, Randsholt NB, Santamaria P: **The *multi sex combs* gene of *Drosophila melanogaster* is required for proliferation of the germline.** *Roux's Arch Dev Biol* 1996, **205:**203.

567. Fritsch C, Beuchle D, Muller J: **Molecular and genetic analysis of the Polycomb group gene *Sex combs extra/Ring* in *Drosophila*.** *Mech Dev* 2003, **120:**949.

568. Paro R, Zink B: **The *Polycomb* gene is differentially regulated during oogenesis and embryogenesis of *Drosophila melanogaster*.** *Mech Dev* 1993, **40:**37.

569. Soto MC, Chou TB, Bender W: **Comparison of germline mosaics of genes in the Polycomb group of *Drosophila melanogaster*.** *Genetics* 1995, **140:**231.

570. Martin EC, Adler PN: **The *Polycomb* group gene *Posterior Sex Combs* encodes a chromosomal protein.** *Development* 1993, **117:**641.

571. Li X, Han Y, Xi R: **Polycomb group genes *Psc* and *Su(z)2* restrict follicle stem cell self-renewal and extrusion by controlling canonical and noncanonical Wnt signaling.** *Genes Dev* 2011, **24:**933.

572. Irminger-Finger I, Nothiger R: **The *Drosophila melanogaster gene lethal(3)73Ah* encodes a ring finger protein homologous to the oncoproteins MEL-18 and BMI-1.** *Gene* 1995, **163:**203-208.

573. Seong K-H, Li D, Shimizu H, Nakamura R, Ishii S: **Inheritance of stress-induced, ATF-2-dependent epigenetic change.** *Cell* 2011, **145:**1049-1061.

574. Smolik-Utlaut SM, Rose RE, Goodman RH: **A cyclic AMP-responsive element-binding transcriptional activator in *Drosophila melanogaster,* dCREB-A, is a member of the leucine zipper family.** *Mol Cell Biol* 1992, **12:**4123-4131.

575. Abel T, Bhatt R, Maniatis T: **A *Drosophila* CREB/ATF transcriptional activator binds to both fat body- and liver-specific regulatory elements.** *Genes Dev* 1992, **6:**466-480.

576. Song H, Sun Y, Zhang Y, Li M: **Molecular cloning and characterization of *Bombyx mori CREB* gene.** *Arch Insect Biochem Physiol* 2009, **71:**31-44.

577. Di Stefano L, Walker JA, Burgio G, Corona DFV, Mulligan P, Näär AM, Dyson NJ: **Functional antagonism between histone H3K4 demethylases in vivo.** *Genes Dev* 2011, **25:**17-28.

578. Carrera P, Moshkin YM, Gronke S, Sillje HH, Nigg EA, Jackle H, Karch F: **Tousled-like kinase functions with the chromatin assembly pathway regulating nuclear divisions.** *Genes Dev* 2003, **17:**2578-2590.

579. Casper AL, Baxter K, Van Doren M: ***no child left behind* encodes a novel chromatin factor required for germline stem cell maintenance in males but not females.** *Development* 2011, **138:**3357-3366.

580. Boulanger MC, Miranda TB, Clarke S, Di Fruscio M, Suter B, Lasko P, Richard S: **Characterization of the *Drosophila* protein arginine methyltransferases DART1 and DART4.** *Biochem J* 2004, **379:**283-289.

581. Tripoulas N, Hersperger E, la Jeunesse D, Shearn A: **Molecular genetic analysis of the *Drosophila melanogaster* gene *absent, small or homeotic discs1 (ash1)*.** *Genetics* 1994, **137:**1027-1038.

582. Frasch M: **The maternally expressed *Drosophila* gene encoding the chromatin-binding protein BJ1 is a homolog of the vertebrate gene *Regulator of Chromatin Condensation, RCC1*.** *EMBO J* 1991, **10:**1225-1236.

583. Chiang CS, Mitsis PG, Lehman IR: **DNA polymerase delta from embryos of *Drosophila melanogaster*.** *Proceedings of the National Academy of Sciences* 1993, **90:**9105-9109.

584. Oshige M, Yoshida H, Hirose F, Takata K, Inoue YH, Aoyagi N, Yamaguchi M, Koiwai O, Matsukage A, Sakaguchi K: **Molecular cloning and expression during development of the *Drosophila* gene for the catalytic subunit of DNA polymerase.** *Gene* 2000, **256:**93-100.

585. Takeuchi R, Ruike T, Nakamura R, Shimanouchi K, Kanai Y, Abe Y, Ihara A, Sakaguchi K: ***Drosophila* DNA polymerase zeta interacts with recombination repair protein 1, the *Drosophila* homologue of human abasic endonuclease 1.** *J Biol Chem* 2006, **281:**11577-11585.

586. Mitsis PG: **Phosphorylation and localization of replication protein A during oogenesis and early embryogenesis of *Drosophila melanogaster*.** *Dev Biol* 1995, **170:**445-456.

587. Crest J, Oxnard N, Ji JY, Schubiger G: **Onset of the DNA replication checkpoint in the early *Drosophila* embryo.** *Genetics* 2007, **175:**567-584.

588. Frank LH, Cheung HK, Cohen RS: **Identification and characterization of *Drosophila* female germ line transcriptional control elements.** *Development* 1992, **114:**481-491.

589. Sander M, Lowenhaupt K, Lane WS, Rich A: **Cloning and characterization of *Rrp1*, the gene encoding *Drosophila* strand transferase: carboxy-terminal homology to DNA repair endo/exonucleases.** *Nucleic Acids Res* 1991, **19:**4523-4529.

590. Zhang CX, Chen AD, Gettel NJ, Hsieh TS: **Essential functions of DNA topoisomerase I in *Drosophila melanogaster*.** *Dev Biol* 2000, **222:**27-40.

591. Lee MP, Brown SD, Chen A, Hsieh TS: **DNA topoisomerase I is essential in *Drosophila melanogaster*.** *Proceedings of the National Academy of Sciences* 1993, **90:**6656-6660.

592. Brown SD, Zhang CX, Chen AD, Hsieh T: **Structure of the *Drosophila* DNA topoisomerase I gene and expression of messages with different lengths in the 3' untranslated region.** *Gene* 1998, **211:**195-203.

593. Wilson TM, Chen AD, Hsieh T: **Cloning and characterization of *Drosophila* topoisomerase III. Relaxation of hypernegatively supercoiled dna.** *J Biol Chem* 2000, **275:**1533-1540.

594. Ohno K, Hirose F, Inoue YH, Takisawa H, Mimura S, Hashimoto Y, Kiyono T, Nishida Y, Matsukage A: **cDNA cloning and expression during development of *Drosophila melanogaster* MCM3, MCM6 and MCM7.** *Gene* 1998, **217:**177-186.

595. Schwed G, May N, Pechersky Y, Calvi BR: ***Drosophila* minichromosome maintenance 6 is required for chorion gene amplification and genomic replication.** *Mol Biol Cell* 2002, **13:**607-620.

596. Grell RF, Generoso EE: **Time of recombination in the *Drosophila melanogaster* oocyte.** *Chromosoma* 1980, **81:**339-348.

597. Lyko F, Whittaker AJ, Orr-Weaver TL, Jaenisch R: **The putative *Drosophila* methyltransferase gene *dDnmt2* is contained in a transposon-like element and is expressed specifically in ovaries.** *Mech Dev* 2000, **95:**215-217.

598. Hanai S, Uchida M, Kobayashi S, Miwa M, Uchida K: **Genomic organization of *Drosophila* Poly(ADP-ribose) polymerase and distribution of its mRNA during development.** *J Biol Chem* 1998, **273:**11881-11886.

599. Kopytova DV, Krasnov AN, Kopantceva MR, Nabirochkina EN, Nikolenko JV, Maksimenko O, Kurshakova MM, Lebedeva LA, Yerokhin MM, Simonova OB, et al: **Two isoforms of *Drosophila* TRF2 are involved in embryonic development, premeiotic chromatin condensation, and proper differentiation of germ cells of both sexes.** *Mol Cell Biol* 2006, **26:**7492-7505.

600. Usakin L, Abad J, Vagin VV, de Pablos B, Villasante A, Gvozdev VA: **Transcription of the 1.688 satellite DNA family is under the control of RNA interference machinery in *Drosophila melanogaster* ovaries.** *Genetics* 2007, **176:**1343-1349.

601. Sedkov Y, Benes JJ, Berger JR, Riker KM, Tillib S, Jones RS, Mazo A: **Molecular genetic analysis of the *Drosophila trithorax-related* gene which encodes a novel SET domain protein.** *Mech Dev* 1999, **82:**171-179.

602. Frasch M, Saumweber H: **Two proteins from *Drosophila* nuclei are bound to chromatin and are detected in a series of puffs on polytene chromosomes.** *Chromosoma* 1989, **97:**272-281.

603. Roy S, Gilbert MK, Hart CM: **Characterization of *BEAF* mutations isolated by homologous recombination in *Drosophila*.** *Genetics* 2007, **176:**801-813.

604. Loppin B, Berger F, Couble P: **The *Drosophila* maternal gene *sesame* is required for sperm chromatin remodeling at fertilization.** *Chromosoma* 2001, **110:**430-440.

605. Akhmanova AS, Bindels PCT, Xu J, Miedema K, Kremer H, Hennig W: **Structure and expression of histone H3.3 genes in *Drosophila melanogaster* and *Drosophila hydei*.** *Genome* 1995, **38:**586-600.

606. Cermelli S, Guo Y, Gross SP, Welte MA: **The lipid-droplet proteome reveals that droplets are a protein-storage depot.** *Curr Biol* 2006, **16:**1783-1795.

607. van Daal A, Elgin SC: **A histone variant, H2AvD, is essential in *Drosophila melanogaster*.** *Mol Biol Cell* 1992, **3:**593-602.

608. Harris PV, Mazina OM, Leonhardt EA, Case RB, Boyd JB, Burtis KC: **Molecular cloning of *Drosophila mus308*, a gene involved in DNA cross-link repair with homology to prokaryotic DNA polymerase I genes.** *Mol Cell Biol* 1996, **16:**5764-5771.

609. Chen G, Fernandez J, Mische S, Courey AJ: **A functional interaction between the histone deacetylase rpd3 and the corepressor groucho in *Drosophila* development.** *Genes Dev* 1999, **13:**2218-2230.

610. Marhold J, Zbylut M, Lankenau DH, Li M, Gerlich D, Ballestar E, Mechler BM, Lyko F: **Stage-specific chromosomal association of *Drosophila* dMBD2/3 during genome activation.** *Chromosoma* 2002, **111:**13-21.

611. Gim BS, Park JM, Yoon JH, Kang C, Kim YJ: ***Drosophila* med6 is required for elevated expression of a large but distinct set of developmentally regulated genes.** *Mol Cell Biol* 2001, **21:**5242-5255.

612. Stroumbakis N, Li Z, Tolias PP: **RNA- and single-stranded DNA-binding (SSB) proteins expressed during *Drosophila melanogaster* oogenesis: a homolog of bacterial and eukaryotic mitochondrial SSBs.** *Gene* 1994, **143:**171-177.

613. Jeong SM, Kawasaki K, Juni N, Shibata T: **Identification of *Drosophila melanogaster* RECQE as a member of a new family of RecQ homologues that is preferentially expressed in early embryos.** *Mol Gen Genet* 2000, **263:**183-193.

614. Horwich MD, Li C, Matranga C, Vagin V, Farley G, Wang P, Zamore PD: **The *Drosophila* RNA methyltransferase, DmHen1, modifies germline piRNAs and single-stranded siRNAs in RISC.** *Curr Biol* 2007, **17:**1265-1272.

615. Dorn R, Morawietz H, Reuter G, Saumweber H: **Identification of an essential *Drosophila* gene that is homologous to the translation initiation factor eIF-4A of yeast and mouse.** *Mol Gen Genet* 1993, **237:**233-240.

616. Pelisson A, Song SU, Prud'homme N, Smith PA, Bucheton A, Corces VG: **Gypsy transposition correlates with the production of a retroviral envelope-like protein under the tissue-specific control of the *Drosophila flamenco* gene.** *EMBO J* 1994, **13:**4401-4411.

617. Song SU, Gerasimova T, Kurkulos M, Boeke JD, Corces VG: **An Env-like protein encoded by a *Drosophila* retroelement: evidence that *gypsy* is an infectious retrovirus.** *Genes Dev* 1994, **8:**2046-2057.

618. Chalvet F, Teysset L, Terzian C, Prud'homme N, Santamaria P, Bucheton A, Pelisson A: **Proviral amplification of the *Gypsy* endogenous retrovirus of *Drosophila melanogaster* involves *env*-independent invasion of the female germline.** *EMBO J* 1999, **18:**2659-2669.

619. Doerflinger H, Lepesant JA, Yanicostas C: **Differential expression of the *Drosophila* zinc finger gene *jim* in the follicular epithelium.** *Mech Dev* 1999, **86:**177-182.

620. Liang HL, Nien CY, Liu HY, Metzstein MM, Kirov N, Rushlow C: **The zinc-finger protein Zelda is a key activator of the early zygotic genome in *Drosophila*.** *Nature* 2008, **456:**400-403.

621. Kanodia J, Liang H-L, Kim Y, Lim B, Zhan M, Lu H, Rushlow C, Shvartsman S: **Pattern formation by graded and uniform signals in the early *Drosophila* embryo.** *Biophys J* 2012, **102:**427-433.

622. Nien C-Y, Liang H-L, Butcher S, Sun Y, Fu S, Gocha T, Kirov N, Manak JR, Rushlow C: **Temporal coordination of gene networks by Zelda in the early *Drosophila* embryo.** *PLoS Genet* 2011, **7:**e1002339.

623. Juhasz I, Villanyi Z, Tombacz I, Boros IM: **High Fcp1 phosphatase activity contributes to setting an intense transcription rate required in *Drosophila* nurse and follicular cells for egg production.** *Gene* 2012, **509:**60-67.

624. Van Buskirk C, Schüpbach T: ***half pint* regulates alternative splice site selection in *Drosophila*.** *Dev Cell* 2002, **2:**343-353.

625. Cox RT, Spradling AC: ***clueless*, a conserved *Drosophila* gene required for mitochondrial subcellular localization, interacts genetically with *parkin*.** *Dis Model Mech* 2009, **2:**490-499.

626. Vorbruggen G, Onel S, Jackle H: **Restricted expression and subnuclear localization of the *Drosophila* gene *Dnop5*, a member of the Nop/Sik family of the conserved rRNA processing factors.** *Mech Dev* 2000, **90:**305-308.

627. Ruhf ML, Meister M: **The *Drosophila* homologue of ribosomal protein L8.** *Insect Biochem Mol Biol* 1999, **29:**349-353.

628. Cabrera HL, Barrio R, Arribas C: **Structure and expression of the *Drosophila* ubiquitin-52-amino-acid fusion-protein gene.** *Biochem J* 1992, **286:**281-288.

629. Barrio R, del Arco A, Cabrera HL, Arribas C: **Structure and expression of the *Drosophila* ubiquitin-80-amino-acid fusion-protein gene.** *Biochem J* 1994, **302:**237-244.

630. Qian S, Hongo S, Jacobs-Lorena M: **Antisense ribosomal protein gene expression specifically disrupts oogenesis in *Drosophila melanogaster*.** *Proceedings of the National Academy of Sciences* 1988, **85:**9601-9605.

631. Kay MA, Jacobs-Lorena M: **Selective translational regulation of ribosomal protein gene expression during early development of *Drosophila melanogaster*.** *Mol Cell Biol* 1985, **5:**3583-3592.

632. Vincent A, O'Connell P, Gray MR, Rosbash M: ***Drosophila* maternal and embryo mRNAs transcribed from a single transcription unit use alternate combinations of exons.** *EMBO J* 1984, **3:**1003-1013.

633. Reynaud E, Bolshakov VN, Barajas V, Kafatos FC, Zurita M: **Antisense suppression of the putative ribosomal protein S3A gene disrupts ovarian development in *Drosophila melanogaster*.** *Mol Gen Genet* 1997, **256:**462-467.

634. Phillips B, Billin AN, Cadwell C, Buchholz R, Erickson C, Merriam JR, Carbon J, Poole SJ: **The *Nop60B* gene of *Drosophila* encodes an essential nucleolar protein that functions in yeast.** *Mol Gen Genet* 1998, **260:**20-29.

635. Giordano E, Peluso I, Senger S, Furia M: ***minifly*, A *Drosophila* gene required for ribosome biogenesis.** *J Cell Biol* 1999, **144:**1123-1133.

636. Parker CG, Fessler LI, Nelson RE, Fessler JH: ***Drosophila* UDP-glucose:glycoprotein glucosyltransferase: Sequence and characterization of an enzyme that distinguishes between denatured and native proteins.** *EMBO J* 1995, **14:**1294-1303.

637. Krauchunas AR, Horner VL, Wolfner MF: **Protein phosphorylation changes reveal new candidates in the regulation of egg activation and early embryogenesis in *D. melanogaster*.** *Dev Biol* 2012, **370:**125-134.

638. Irazoqui JE, Ng A, Xavier RJ, Ausubel FM: **Role for beta-catenin and HOX transcription factors in *Caenorhabditis elegans* and mammalian host epithelial-pathogen interactions.** *Proceedings of the National Academy of Sciences* 2008.

639. Schmucker D, Vorbruggen G, Yeghiayan P, Fan HQ, Jackle H, Gaul U: **The *Drosophila* gene *abstrakt*, required for visual system development, encodes a putative RNA helicase of the DEAD box protein family.** *Mech Dev* 2000, **91:**189-196.

640. Garcia-Bellido A, Robbins LG: **Viability of female germ-line cells homozygous for zygotic lethals in *Drosophila melanogaster*.** *Genetics* 1983, **103:**235-247.

641. Zhang SD, Kassis J, Olde B, Mellerick DM, Odenwald WF: **Pollux, a novel *Drosophila* adhesion molecule, belongs to a family of proteins expressed in plants, yeast, nematodes, and man.** *Genes Dev* 1996, **10:**1108-1119.

642. Merkle JA, Rickmyre JL, Garg A, Loggins EB, Jodoin JN, Lee E, Wu LP, Lee LA: ***no poles* encodes a predicted E3 ubiquitin ligase required for early embryonic development of *Drosophila*.** *Development* 2009, **136:**449-459.

643. Aguilera M, Oliveros M, Martinez-Padron M, Barbas JA, Ferrus A: ***Ariadne-1*. A vital *Drosophila* gene is required in development and defines a new conserved family of ring-finger proteins.** *Genetics* 2000, **155:**1231-1244.

644. Orgad S, Rosenfeld G, Greenspan RJ, Segal D: ***courtless*, the *Drosophila* UBC7 homolog, is involved in male courtship behavior and spermatogenesis.** *Genetics* 2000, **155:**1267-1280.

645. Bergstrom DE, Merli CA, Cygan JA, Shelby R, Blackman RK: **Regulatory autonomy and molecular characterization of the *Drosophila out at first* gene.** *Genetics* 1995, **139:**1331-1346.

646. Ellis HM: **Embryonic expression and function of the *Drosophila* helix-loop-helix gene, *extramacrochaetae*.** *Mech Dev* 1994, **47:**65-72.

647. Papadia S, Tzolovsky G, Zhao D, Leaper K, Clyde D, Taylor P, Asscher E, Kirk G, Bownes M: ***emc* has a role in dorsal appendage fate formation in *Drosophila* oogenesis.** *Mech Dev* 2005, **122:**961-974.

648. Sahota VK, Grau BF, Mansilla A, Ferrús A: **Troponin I and Tropomyosin regulate chromosomal stability and cell polarity.** *J Cell Sci* 2009, **122:**2623-2631.

649. Hales KH, Meredith JE, Storti RV: **Transcriptional and post-transcriptional regulation of maternal and zygotic cytoskeletal**

tropomyosin mRNA during *Drosophila* development correlates with specific morphogenic events. *Dev Biol* 1994, **165:**639-653.

650. Anderson SM, Brown MR, McDonald JF: **Tissue-specific expression of the *Drosophila Adh* gene: a comparison of in situ hybridization and immunocytochemistry.** *Genetica* 1991, **84:**95-100.

651. Visa N, Fibla J, Gonzalez-Duarte R, Santa-Cruz MC: **Progressive redistribution of alcohol dehydrogenase during vitellogenesis in *Drosophila melanogaster*: characterization of ADH-positive bodies in mature oocytes.** *Cell Tissue Res* 1992, **268:**217-224.

652. Nakamura A, Amikura R, Mukai M, Kobayashi S, Lasko PF: **Requirement for a noncoding RNA in *Drosophila* polar granules for germ cell establishment.** *Science* 1996, **274:**2075-2079.

653. Tadros W, Houston SA, Bashirullah A, Cooperstock RL, Semotok JL, Reed BH, Lipshitz HD: **Regulation of maternal transcript destabilization during egg activation in *Drosophila*.** *Genetics* 2003, **164:**989-1001.

654. Shamanski FL, Orr-Weaver TL: **The *Drosophila plutonium* and *pan gu* genes regulate entry into S phase at fertilization.** *Cell* 1991, **66:**1289-1300.

655. Fenger DD, Carminati JL, Burney-Sigman DL, Kashevsky H, Dines JL, Elfring LK, Orr-Weaver TL: **PAN GU: a protein kinase that inhibits S phase and promotes mitosis in early *Drosophila* development.** *Development* 2000, **127:**4763-4774.

656. Zhang N, Zhang J, Cheng Y, Howard K: **Identification and genetic analysis of *wunen*, a gene guiding *Drosophila melanogaster* germ cell migration.** *Genetics* 1996, **143:**1231-1241.

657. Kadrmas JL, Smith MA, Pronovost SM, Beckerle MC: **Characterization of RACK1 function in *Drosophila* development.** *Dev Dyn* 2007, **236:**2207-2215.

658. Costello JC, Dalkilic MM, Beason SM, Gehlhausen JR, Patwardhan R, Middha S, Eads BD, Andrews JR: **Gene networks in *Drosophila melanogaster*: integrating experimental data to predict gene function.** *Genome Biol* 2009, **10:**R97.

659. Stroumbakis ND, Li Z, Tolias PP: **A homolog of human transcription factor NF-X1 encoded by the *Drosophila shuttle craft* gene is required in the embryonic central nervous system.** *Mol Cell Biol* 1996, **16:**192-201.

660. Vicente M, Monferrer L, Poulos MG, Houseley J, Monckton DG, O'Dell KMC, Swanson MS, Artero RD: **Muscleblind isoforms are functionally distinct and regulate *α-actinin* splicing.** *Differentiation* 2007, **75:**427-440.

661. Huang JD, Dubnicoff T, Liaw GJ, Bai Y, Valentine SA, Shirokawa JM, Lengyel JA, Courey AJ: **Binding sites for transcription factor NTF-1/Elf-1 contribute to the ventral repression of *decapentaplegic*.** *Genes Dev* 1995, **9:**3177-3189.

662. Steward R, McNally FJ, Schedl P: **Isolation of the *dorsal* locus of *Drosophila*.** *Nature* 1984, **311:**262-265.

663. Reeves GT, Stathopoulos A: **Graded Dorsal and differential gene regulation in the *Drosophila* embryo.** *Cold Spring Harb Perspect Biol* 2009, **1**.

664. Lund VK, DeLotto Y, DeLotto R: **Endocytosis is required for Toll signaling and shaping of the Dorsal/NF-kB morphogen gradient during *Drosophila***

embryogenesis. *Proceedings of the National Academy of Sciences* 2010, **107:**18028-18033.

665. Morisato D, Anderson KV: **Signaling pathways that establish the dorsal-ventral pattern of the Drosophila embryo.** *Annu Rev Genet* 1995, **29:**371-399.

666. Digilio FA, Pannuti A, Lucchesi JC, Furia M, Polito LC: ***Tosca*: A *Drosophila* gene encoding a nuclease specifically expressed in the female germline.** *Dev Biol* 1996, **178:**90-100.

667. Yun B, Farkas R, Lee K, Rabinow L: **The *Doa* locus encodes a member of a new protein kinase family and is essential for eye and embryonic development in *Drosophila melanogaster*.** *Genes Dev* 1994, **8:**1160-1173.

668. Bai C, Tolias PP: **Cleavage of RNA hairpins mediated by a developmentally regulated CCCH zinc finger protein.** *Mol Cell Biol* 1996, **16:**6661-6667.

669. George H, Terracol R: **The *vrille* gene of *Drosophila* is a maternal enhancer of *decapentaplegic* and encodes a new member of the bZIP family of transcription factors.** *Genetics* 1997, **146:**1345-1363.

670. Goulding SE, zur Lage P, Jarman AP: ***amos*, a proneural gene for *Drosophila* olfactory sense organs that is regulated by *lozenge*.** *Neuron* 2000, **25:**69-78.

671. Wrana JL, Tran H, Attisano L, Arora K, Childs SR, Massague J, O'Connor MB: **Two distinct transmembrane serine/threonine kinases from *Drosophila melanogaster* form an activin receptor complex.** *Mol Cell Biol* 1994, **14:**944-950.

672. Schulz RA, Butler BA: **Overlapping genes of *Drosophila melanogaster*: organization of the *z600-gonadal-Eip28/29* gene cluster.** *Genes Dev* 1989, **3:**232-242.

673. Boltz KA, Ellis LL, Carney GE: ***Drosophila melanogaster* p24 genes have developmental, tissue-specific, and sex-specific expression patterns and functions.** *Dev Dyn* 2007, **236:**544-555.

674. Carney GE, Taylor BJ: ***logjam* encodes a predicted EMP24/GP25 protein that is required for *Drosophila* oviposition behavior.** *Genetics* 2003, **164:**173-186.

675. Charroux B, Angelats C, Fasano L, Kerridge S, Vola C: **The levels of the *bancal* product, a *Drosophila* homologue of vertebrate hnRNP K protein, affect cell proliferation and apoptosis in imaginal disc cells.** *Mol Cell Biol* 1999, **19:**7846-7856.

676. Stebbings L, Grimes BR, Bownes M: **A testis-specifically expressed gene is embedded within a cluster of maternally expressed genes at 89B in *Drosophila melanogaster*.** *Dev Genes Evol* 1998, **208:**523-530.

677. Konrad KD, Marsh JL: **Developmental expression and spatial distribution of *dopa decarboxylase* in *Drosophila*.** *Dev Biol* 1987, **122:**172-185.

678. Spencer CA, Gietz RD, Hodgetts RB: **Overlapping transcription units in the dopa decarboxylase region of *Drosophila*.** *Nature* 1986, **322:**279-281.

679. Gietz RD, Hodgetts RB: **An analysis of dopa decarboxylase expression during embryogenesis in *Drosophila melanogaster*.** *Dev Biol* 1985, **107:**142-155.

680. Maier D, Nagel AC, Johannes B, Preiss A: **Subcellular localization of Hairless protein shows a major focus of activity within the nucleus.** *Mech Dev* 1999, **89:**195-199.

681. Bang AG, Posakony JW: **The *Drosophila* gene *hairless* encodes a novel basic protein that controls alternative cell fates in adult sensory organ development.** *Genes Dev* 1992, **6:**1752-1769.

682. Schweisguth F, Posakony JW: **Suppressor of *Hairless,* the *Drosophila* homolog of the mouse recombination signal-binding protein gene, controls sensory organ cell fates.** *Cell* 1992, **69:**1199-1212.

683. Girdham CH, Glover DM: **Chromosome tangling and breakage at anaphase result from mutations in *lodestar,* a *Drosophila* gene encoding a putative nucleoside triphosphate-binding protein.** *Genes Dev* 1991, **5:**1786-1799.

684. Slee R, Bownes M: **The *raspberry* locus encodes *Drosophila* inosine monophosphate dehydrogenase.** *Mol Gen Genet* 1995, **248:**755-766.

685. Miklos GLG, Yamamoto MT, Burns RG, Maleszka R: **An essential cell division gene of *Drosophila,* absent from *Saccharomyces,* encodes an unusual protein with tubulin-like and myosin-like peptide motifs.** *Proceedings of the National Academy of Sciences* 1997, **94:**5189-5194.

686. Maleszka R, de Couet HG, Miklos GLG: **Data transferability from model organisms to human beings: insights from the functional genomics of the *flightless* region of *Drosophila*.** *Proceedings of the National Academy of Sciences* 1998, **95:**3731-3736.

687. Djagaeva I, Doronkin S, Beckendorf SK: **Src64 is involved in fusome development and karyosome formation during *Drosophila* oogenesis.** *Dev Biol* 2005, **284:**143-156.

688. Fares H, Peifer M, Pringle JR: **Localization and possible functions of *Drosophila* septins.** *Mol Biol Cell* 1995, **6:**1843-1859.

689. Shih HP, Hales KG, Pringle JR, Peifer M: **Identification of septin-interacting proteins and characterization of the Smt3/SUMO-conjugation system in *Drosophila*.** *J Cell Sci* 2002, **115:**1259-1271.

690. Prokopenko SN, Saint R, Bellen HJ: **Tissue distribution of *PEBBLE* RNA and Pebble protein during *Drosophila* embryonic development.** *Mech Dev* 2000, **90:**269-273.

691. Uemura T, Shepherd S, Ackerman L, Jan LY, Jan YN: ***numb*, a gene required in determination of cell fate during sensory organ formation in *Drosophila* embryos.** *Cell* 1989, **58:**349-360.

692. Klichko VI, Radyuk SN, Orr WC: **Profiling catalase gene expression in *Drosophila melanogaster* during development and aging.** *Arch Insect Biochem Physiol* 2004, **56:**34-50.

693. Radyuk SN, Klichko VI, Orr WC: **Profiling Cu,Zn-superoxide dismutase expression in *Drosophila melanogaster*--a critical regulatory role for intron/exon sequence within the coding domain.** *Gene* 2004, **328:**37-48.

694. Parkes TL, Kirby K, Phillips JP, Hilliker AJ: **Transgenic analysis of the cSOD-null phenotypic syndrome in *Drosophila*.** *Genome* 1998, **41:**642-651.

695. Feger G, Vassin H, Su TT, Wolff E, Jan LY, Jan YN: ***dpa*, A member of the MCM family, is required for mitotic DNA replication but not endoreplication in *Drosophila*.** *EMBO J* 1995, **14:**5387-5398.

696. Tessier CR, Broadie K: **Molecular and genetic analysis of the *Drosophila* model of fragile X syndrome.** *Results Probl Cell Differ* 2012, **54:**119-156.

697. Pepper AS, Beerman RW, Bhogal B, Jongens TA: **Argonaute2 suppresses *Drosophila fragile X* expression preventing neurogenesis and oogenesis defects.** *PLoS ONE* 2009, **4:**e7618.

698. Wan L, Dockendorff TC, Jongens TA, Dreyfuss G: **Characterization of dFMR1, a *Drosophila melanogaster* homolog of the Fragile X Mental Retardation protein.** *Mol Cell Biol* 2000, **20:**8536-8547.

699. Zarnescu DC, Jin P, Betschinger J, Nakamoto M, Wang Y, Dockendorff TC, Feng Y, Jongens TA, Sisson JC, Knoblich JA, et al: **Fragile X protein functions with lgl and the par complex in flies and mice.** *Dev Cell* 2005, **8:**43-52.

700. Ratan R, Mason DA, Sinnot B, Goldfarb DS, Fleming RJ: ***Drosophila* Importin alpha1 performs paralog-specific functions essential for gametogenesis.** *Genetics* 2008, **178:**839-850.

701. Lippai M, Tirian L, Boros I, Mihaly J, Erdelyi M, Belecz I, Mathe E, Posfai J, Nagy A, Udvardy A, et al: **The *Ketel* gene encodes a *Drosophila* homologue of Importin.** *Genetics* 2000, **156:**1889-1900.

702. Colas JF, Launay JM, Maroteaux L: **Maternal and zygotic control of serotonin biosynthesis are both necessary for *Drosophila* germband extension.** *Mech Dev* 1999, **87:**67-76.

703. Gorjanacz M, Adam G, Torok I, Mechler BM, Szlanka T, Kiss I: **Importin-alpha2 Is critically required for the assembly of ring canals during *Drosophila* oogenesis.** *Dev Biol* 2002, **251:**271-282.

704. Kawamura K, Shibata T, Saget O, Peel D, Bryant PJ: **A new family of growth factors produced by the fat body and active on *Drosophila* imaginal disc cells.** *Development* 1999, **126:**211-219.

705. Heck MMS, Pereira A, Pesavento P, Yannoni Y, Spradling AC, Goldstein LSB: **The kinesin-like protein KLP61F is essential for mitosis in *Drosophila*.** *J Cell Biol* 1993, **123:**665-679.

706. Schulz C, Perezgasga L, Fuller MT: **Genetic analysis of *dPsa*, the *Drosophila* orthologue of puromycin-sensitive aminopeptidase, suggests redundancy of aminopeptidases.** *Dev Genes Evol* 2001, **211:**581-588.

707. Martin JR, Ollo R: **A new *Drosophila* Ca[2+]/calmodulin-dependent protein kinase (Caki) is localized in the central nervous system and implicated in walking speed.** *EMBO J* 1996, **15:**1865-1876.

708. Galasso A, Pane LS, Russo M, Grimaldi MR, Verrotti AC, Gigliotti S, Graziani F: ***dSTAM* expression pattern during wild type and mutant egg chamber development in *D. melanogaster*.** *Gene Expr Patterns* 2007, **7:**730-737.

709. Qi D, Larsson J, Mannervik M: ***Drosophila* Ada2b is required for viability and normal Histone H3 acetylation.** *Mol Cell Biol* 2004, **24:**8080-8089.

710. Poortinga G, Watanabe M, Parkhurst SM: ***Drosophila* CtBP: a Hairy-interacting protein required for embryonic segmentation and Hairy-mediated transcriptional repression.** *EMBO J* 1998, **17:**2067-2078.

711. Hahn M, Bishop JM: **Expression pattern of *Drosophila ret* suggests a common ancestral origin between the metamorphosis precursors in insect endoderm and the vertebrate enteric neurons.** *Proceedings of the National Academy of Sciences* 2001, **98:**1053-1058.

712. Hayflick JS, Wolfgang WJ, Forte MA, Thomas G: **A unique Kex2-like endoprotease from *Drosophila melanogaster* is expressed in the central nervous system during early embryogenesis.** *J Neurosci* 1992, **12:**705-717.

713. Konsolaki M, Schüpbach T: ***windbeutel*, a gene required for dorsoventral patterning in *Drosophila*, encodes a protein that has homologies to vertebrate proteins of the endoplasmic reticulum.** *Genes Dev* 1998, **12:**120-131.

714. DeGennaro M, Hurd TR, Siekhaus DE, Biteau B, Jasper H, Lehmann R: **Peroxiredoxin stabilization of DE-cadherin promotes primordial germ cell adhesion.** *Dev Cell* 2011, **20:**233-243.

715. Salz HK, Flickinger TW, Mittendorf E, Pellicena-Palle A, Petschek JP, Albrecht EB: **The *Drosophila* maternal effect locus *deadhead* encodes a thioredoxin homolog required for female meiosis and early embryonic development.** *Genetics* 1994, **136:**1075-1086.

716. Tsuda M, Ootaka R, Ohkura C, Kishita Y, Seong KH, Matsuo T, Aigaki T: **Loss of *Trx-2* enhances oxidative stress-dependent phenotypes in *Drosophila*.** *FEBS Lett* 2010, **584:**3398-3401.

717. Ait-Ahmed O, Thomas-Cavallin M, Joblet C, Capri M: **Expression in the central nervous system of a subset of the *yema* maternally acting genes during *Drosophila* embryogenesis. Post-embryonic expression extends to imaginal discs and spermatocytes.** *Cell Diff Dev* 1990, **31:**53-65.

718. Vincent A, Kejzlarova-Lepesant J, Segalat L, Yanicostas C, Lepesant JA: **sry h-1, a new *Drosophila melanogaster* multifingered protein gene showing maternal and zygotic expression.** *Mol Cell Biol* 1988, **8:**4459-4468.

719. Payre F, Yanicostas C, Vincent A: **Serendipity, a *Drosophila* zinc finger protein present in embryonic nuclei at the onset of zygotic gene transcription.** *Dev Biol* 1989, **136:**469-480.

720. Mutsuddi M, Lakhotia SC: **Spatial expression of the *hsr-omega (93D)* gene in different tissues of *Drosophila melanogaster* and identification of promoter elements controlling its developmental expression.** *Dev Genet* 1995, **17:**303-311.

721. Alphey L, Parker L, Hawcroft G, Guo Y, Kaiser K, Morgan G: **KLP38B: A mitotic kinesin-related protein that binds PP1.** *J Cell Biol* 1997, **138:**395-409.

722. Ohkura H, Torok T, Tick G, Hoheisel J, Kiss I, Glover DM: **Mutation of a gene for a *Drosophila* kinesin-like protein, *Klp38B,* leads to failure of cytokinesis.** *J Cell Sci* 1997, **110:**945-954.

723. Molina I, Baars S, Brill JA, Hales KG, Fuller MT, Ripoll P: **A chromatin-associated kinesin-related protein required for normal mitotic chromosome segregation in *Drosophila*.** *J Cell Biol* 1997, **139:**1361-1371.

724. Quan F, Wolfgang WJ, Forte M: **A *Drosophila* G-protein subunit, *Gf* , expressed in a spatially and temporally restricted pattern during *Drosophila* development.** *Proceedings of the National Academy of Sciences* 1993, **90:**4236-4240.

725. Yarfitz S, Niemi GA, McConnell JL, Fitch CL, Hurley JB: **A G protein in the *Drosophila* compound eye is different from that in the brain.** *Neuron* 1991, **7:**429-438.

726. Ray K, Ganguly R: **Organization and expression of the *Drosophila melanogaster D-Gamma1* gene encoding the G-protein gamma subunit.** *Gene* 1994, **148:**315-319.

727. Tanaka H, Ishibashi J, Fujita K, Nakajima Y, Sagisaka A, Tomimoto K, Suzuki N, Yoshiyama M, Kaneko Y, Iwasaki T, et al: **A genome-wide analysis of genes and gene families involved in innate immunity of *Bombyx mori*.** *Insect Biochem Mol Biol* 2008, **38:**1087.

728. Shelton CA, Wasserman SA: ***pelle* encodes a protein kinase required to establish dorsoventral polarity in the *Drosophila* embryo.** *Cell* 1993, **72:**515.

729. Muller-Holtkamp F, Knipple DC, Seifert E, Jaeckle H: **An early role of maternal mRNA in establishing the dorsoventral pattern in *pelle* mutant *Drosophila* embryos.** *Dev Biol* 1985, **110:**238.

730. Cho YS, Stevens LM, Sieverman KJ, Nguyen J, Stein D: **A ventrally localized protease in the *Drosophila* egg controls embryo dorsoventral polarity.** *Curr Biol* 2012, **22:**1013-1018.

731. Stein D, Nüsslein-Volhard C: **Multiple extracellular activities in *Drosophila* egg perivitelline fluid are required for establishment of embryonic dorsal-ventral polarity.** *Cell* 1992, **68:**429.

732. Araujo H, Bier E: **sog and dpp exert opposing maternal functions to modify Toll signaling and pattern the dorsoventral axis of the *Drosophila* embryo.** *Development* 2000, **127:**3631.

733. Carneiro K, Fontenele M, Negreiros E, Lopes E, Bier E, Araujo H: **Graded maternal short gastrulation protein contributes to embryonic dorsal–ventral patterning by delayed induction.** *Dev Biol* 2006, **296:**203-218.

734. Chen LY, Wang JC, Hyvert Y, Lin HP, Perrimon N, Imler JL, Hsu JC: **Weckle is a zinc finger adaptor of the Toll pathway in dorsoventral patterning of the *Drosophila* embryo.** *Curr Biol* 2006, **16:**1183-1193.

735. Lynch JA, Roth S: **The evolution of dorsal–ventral patterning mechanisms in insects.** *Genes Dev* 2011, **25:**107-118.

736. Jang IH, Chosa N, Kim SH, Nam HJ, Lemaitre B, Ochiai M, Kambris Z, Brun S, Hashimoto C, Ashida M, et al: **A Spatzle-processing enzyme required for toll signaling activation in *Drosophila* innate immunity.** *Dev Cell* 2006, **10:**45-55.

737. Gay NJ, Keith FJ: **Regulation of translation and proteolysis during the development of embryonic dorso-ventral polarity in *Drosophila*. Homology of easter proteinase with Limulus proclotting enzyme and translational activation of Toll receptor synthesis.** *Biochim Biophys Acta* 1992, **1132:**290-296.

738. Gerttula S, Jin YS, Anderson KV: **Zygotic expression and activity of the *Drosophila Toll* gene, a gene required maternally for embryonic dorsal-ventral pattern formation.** *Genetics* 1988, **119:**123-133.

739. Anderson KV, Jurgens G, Nüsslein-Volhard C: **Establishment of dorsal-ventral polarity in the *Drosophila* embryo. Genetic studies on the role of the Toll gene product.** *Cell* 1985, **42:**779.

740. Theopold U, Pinter M, Daffre S, Tryselius Y, Friedrich P, Nassel DR, Hultmark D: ***CalpA,* a *Drosophila* calpain homolog specifically expressed in a small set of nerve, midgut and blood cells.** *Mol Cell Biol* 1995, **15:**824-834.

741. Parks S, Wieschaus E: **The *Drosophila* gastrulation gene *concertina* encodes a G -like protein.** *Cell* 1991, **64:**447-458.

742. Mirouse V, Swick LL, Kazgan N, St Johnston D, Brenman JE: **LKB1 and AMPK maintain epithelial cell polarity under energetic stress.** *J Cell Biol* 2007, **177:**387-392.

743. Larochelle S, Suter B: **The *Drosophila melanogaster* homolog of the mammalian MAPK-activated protein kinase-2 (MAPKAPK-2) lacks a proline-rich N terminus.** *Gene* 1995, **163:**209-214.

744. Rendahl KG, Jones KR, Kulkarni SJ, Bagully SH, Hall JC: **The *dissonance* mutation at the *no-on-transient-A* locus of *Drosophila melanogaster*: genetic control of courtship song and visual behaviors by a protein with putative RNA-binding motifs.** *J Neurosci* 1992, **12:**390-407.

745. Glaser RL, Hickey AJ, Chotkowski HL, Chu-LaGraff Q: **Characterization of *Drosophila* palmitoyl-protein thioesterase 1.** *Gene* 2003, **312:**271-279.

746. Wadsworth SC, Madhavan K, Bilodeau-Wentworth D: **Maternal inheritance of transcripts from three *Drosophila src*-related genes.** *Nucleic Acids Res* 1985, **13:**2153-2170.

747. Martin D, Zusman S, Li X, Williams EL, Khare N, DaRocha S, Chiquet-Ehrismann R, Baumgartner S: ***wing blister*, a new *Drosophila* laminin alpha chain required for cell adhesion and migration during embryonic and imaginal development.** *J Cell Biol* 1999, **145:**191-201.

748. Buszczak M, Paterno S, Lighthouse D, Bachman J, Planck J, Owen S, Skora AD, Nystul TG, Ohlstein B, Allen A, et al: **The Carnegie protein trap library: A versatile tool for *Drosophila* developmental studies.** *Genetics* 2007, **175:**1505-1531.

749. Szuplewski S, Terracol R: **The *cyclope* gene of *Drosophila* encodes a cytochrome c oxidase subunit VIc homolog.** *Genetics* 2001, **158:**1629-1643.

750. Bai C, Li Z, Tolias PP: **Developmental characterization of a *Drosophila* RNA-binding protein homologous to the human systemic lupus erythematosus-associated La/SS-B autoantigen.** *Mol Cell Biol* 1994, **14:**5123-5129.

751. Decoville M, Giraud-Panis MJ, Mosrin-Huaman C, Leng M, Locker D: **HMG boxes of DSP1 protein interact with the Rel homology domain of transcription factors.** *Nucleic Acids Res* 2000, **28:**454-462.

752. Brickman JM, Adam M, Ptashne M: **Interactions between an HMG-1 protein and members of the Rel family.** *Proceedings of the National Academy of Sciences* 1999, **96:**10679-10683.

753. Canaple L, Decoville M, Leng M, Locker D: **The *Drosophila DSP1* gene encoding an HMG 1-like protein: genomic organization, evolutionary conservation and expression.** *Gene* 1997, **184:**285-290.

754. McCormack A, MacIver B, Bownes M: **Cloning and expression of *az2*, a putative zinc finger transcription factor from *Drosophila melanogaster*.** *Dev Genes Evol* 1998, **208:**172-174.

755. Nguyen M, Parker L, Arora K: **Identification of *maverick*, a novel member of the TGF-beta superfamily in *Drosophila*.** *Mech Dev* 2000, **95:**201-206.

756. Okajima T, Irvine KD: **Regulation of notch signaling by *O*-linked fucose.** *Cell* 2002, **111:**893-904.

757. Johnson K, Knust E, Skaer H: ***bloated tubules* (*blot*) encodes a *Drosophila* member of the neurotransmitter transporter family required for organisation of the apical cytocortex.** *Dev Biol* 1999, **212:**440-454.

758. Magyar A, Bakos E, Varadi A: **Structure and tissue-specific expression of the *Drosophila melanogaster* organellar-type Ca[2+]-ATPase gene.** *Biochem J* 1995, **310:**757-763.

759. de Nooij JC, Graber KH, Hariharan IK: **Expression of the cyclin-dependent kinase inhibitor Dacapo is regulated by Cyclin E.** *Mech Dev* 2000, **97:**73-83.

760. Patterson LF, Harvey M, Lasko PF: ***Dbp73D,* a *Drosophila* gene expressed in ovary, encodes a novel D-E-A-D box protein.** *Nucleic Acids Res* 1992, **20:**3063-3067.

761. Hofmeyer K, Maurel-Zaffran C, Sink H, Treisman JE: **Liprin-*α* has LAR-independent functions in R7 photoreceptor axon targeting.** *Proceedings of the National Academy of Sciences* 2006, **103:**11595-11600.

762. Ragone G, Caizzi R, Moschetti R, Barsanti P, De Pinto V, Caggese C: **The *Drosophila melanogaster* gene for the NADH:ubiquinone oxidoreductase acyl carrier protein: developmental expression analysis and evidence for alternatively spliced forms.** *Mol Gen Genet* 1999, **261:**690-697.

763. Hwa JJ, Hiller MA, Fuller MT, Santel A: **Differential expression of the *Drosophila* mitofusin genes *fuzzy onions* (*fzo*) and *dmfn*.** *Mech Dev* 2002, **116:**213-216.

764. Darboux I, Lingueglia E, Champigny G, Coscoy S, Barbry P, Lazdunski M: **dGNaC1, a gonad-specific amiloride-sensitive Na+ channel.** *J Biol Chem* 1998, **273:**9424-9429.

765. Roman G, He J, Davis RL: ***kurtz,* a novel nonvisual arrestin, is an essential neural gene in *Drosophila*.** *Genetics* 2000, **155:**1281-1295.

766. Zhang N, Wilkinson K, Bownes M: **Cloning and analysis of expression of a ubiquitin carboxyl terminal hydrolase expressed during oogenesis in *Drosophila melanogaster*.** *Dev Biol* 1993, **157:**214-223.

767. McNeil GP, Zhang X, Roberts M, Jackson FR: **Maternal function of a retroviral-type zinc-finger protein is essential for *Drosophila* development.** *Dev Genet* 1999, **25:**387-396.

768. Schwientek T, Bennett EP, Flores C, Thacker J, Hollmann M, Reis CA, Behrens J, Mandel U, Keck B, Schaefer MA, et al: **Functional conservation of subfamilies of putative UDP-*N*-acetylgalactosamine:polypeptide N-acetylgalactosaminyltransferases in *Drosophila, Caenorhabditis elegans,* and mammals. One subfamily composed of *l(2)35Aa* is essential in *Drosophila*.** *J Biol Chem* 2002, **277:**22623-22638.

769. Khare N, Fascetti N, DaRocha S, Chiquet-Ehrismann R, Baumgartner S: **Expression patterns of two new members of the semaphorin family in *Drosophila* suggest early functions during embryogenesis.** *Mech Dev* 2000, **91:**393-397.

770. Hirosawa-Takamori M, Jackle H, Vorbruggen G: **The *class 2 selenophosphate synthetase* gene of *Drosophila* contains a functional mammalian-type SECIS.** *EMBO Rep* 2000, **1:**441-446.

771. Irles P, Silva-Torres FA, Piulachs M-D: **RNAi reveals the key role of Nervana 1 in cockroach oogenesis and embryo development.** *Insect Biochem Mol Biol*.

772. Wang Z, Lindquist S: **Developmentally regulated nuclear transport of transcription factors in *Drosophila* embryos enable the heat shock response.** *Development* 1998, **125:**4841-4850.

773. Lebo MS, Sanders LE, Sun F, Arbeitman MN: **Somatic, germline and sex hierarchy regulated gene expression during *Drosophila* metamorphosis.** *BMC Genomics* 2009, **10:**80.

774. Pflanz R, Hoch M: ***dtrap-1* encodes a novel member of the heat shock super family of proteins and is expressed in derivatives of all three germ layers during *Drosophila* embryogenesis.** *Mech Dev* 2000, **96:**219-222.

775. Flatt T, Min KJ, D'Alterio C, Villa-Cuesta E, Cumbers J, Lehmann R, Jones DL, Tatar M: ***Drosophila* germ-line modulation of insulin signaling and lifespan.** *Proceedings of the National Academy of Sciences* 2008, **105:**6368-6373.

776. Pauli D, Arrigo AP, Vazquez J, Tonka CH, Tissieres J: **Expression of the small heat shock genes during *Drosophila* development: comparison of the accumulation of *Hsp23* and *Hsp27* mRNAs and polypeptides.** *Genome* 1989, **31:**671-676.

777. Marin R, Tanguay R: **Stage-specific localization of the small heat shock protein Hsp27 during oogenesis in *Drosophila melanogaster*.** *Chromosoma* 1996, **105:**142-149.

778. Nakahara K, Kim K, Sciulli C, Dowd SR, Minden JS, Carthew RW: **Targets of microRNA regulation in the *Drosophila* oocyte proteome.** *Proceedings of the National Academy of Sciences* 2005, **102:**12023-12028.

779. Palter KB, Watanabe M, Stinson L, Mahowald AP, Craig EA: **Expression and localization of *Drosophila melanogaster* Hsp70 cognate proteins.** *Mol Cell Biol* 1986, **6:**1187-1203.

780. Kankare M, Salminen T, Laiho A, Vesala L, Hoikkala A: **Changes in gene expression linked with adult reproductive diapause in a northern malt fly species: a candidate gene microarray study.** *BMC Ecol* 2010, **10:**3.

781. Cobreros L, Fernández-Miñán A, Luque CM, González-Reyes A, Martín-Bermudo MD: **A role for the chaperone Hsp70 in the regulation of border cell migration in the *Drosophila* ovary.** *Mech Dev* 2008, **125:**1048-1058.

782. Sarkar S, Lakhotia SC: **Hsp60C is required in follicle as well as germline cells during oogenesis in *Drosophila melanogaster*.** *Dev Dyn* 2008, **237:**1334-1347.

783. Williams BC, Dernburg AF, Puro J, Nokkala S, Goldberg ML: **The *Drosophila* kinesin-like protein KLP3A is required for proper behavior of male and female pronuclei at fertilization.** *Development* 1997, **124:**2365-2376.

784. Moribe Y, Niimi T, Yamashita O, Yaginuma T: ***Samui*, a novel cold-inducible gene, encoding a protein with a BAG domain similar to silencer of death domains (SODD/BAG-4), isolated from *Bombyx* diapause eggs.** *Eur J Biochem* 2001, **268:**3432-3442.

785. Cole KD, Fernando-Warnakulasuriya GP, Boguski MS, Freeman M, Gordon JI, Clark WA, Law JH, Wells MA: **Primary structure and comparative sequence analysis of an insect apolipoprotein. Apolipophorin-III from *Manduca sexta*.** *J Biol Chem* 1987, **262:**11794-11800.

786. Yamauchi Y, Hoeffer C, Yamamoto A, Takeda H, Ishihara R, Maekawa H, Sato R, Su-Il S, Sumida M, Wells MA, Tsuchida K: **cDNA and deduced amino acid sequences of apolipophorin-IIIs from *Bombyx mori* and *Bombyx mandarina*.** *Arch Insect Biochem Physiol* 2000, **43:**16-21.

787. Parra-Peralbo E, Culi J: ***Drosophila* lipophorin receptors mediate the uptake of neutral lipids in oocytes and imaginal disc cells by an endocytosis-independent mechanism.** *PLoS Genet* 2011, **7:**e1001297.

788. Sundermeyer K, Hendricks JK, Prasad SV, Wells MA: **The precursor protein of the structural apolipoproteins of lipophorin: cDNA and deduced amino acid sequence.** *Insect Biochem Mol Biol* 1996, **26:**735-738.

789. Tufail M, Takeda M: **Insect vitellogenin/lipophorin receptors: Molecular structures, role in oogenesis, and regulatory mechanisms.** *J Insect Physiol* 2009, **55:**88-104.

790. Gopalapillai R, Kadono-Okuda K, Tsuchida K, Yamamoto K, Nohata J, Ajimura M, Mita K: **Lipophorin receptor of *Bombyx mori*: cDNA cloning, genomic structure, alternative splicing, and isolation of a new isoform.** *J Lipid Res* 2006, **47:**1005-1013.

791. Kumaran AK, Memmel NA, Wang C, Trewitt PM: **Developmental regulation of arylphorin gene activity in fat body cells and gonadal sheath cells of *Galleria mellonella*.** *Insect Biochem Mol Biol* 1993, **23:**145-151.

792. Manohar D, Gullipalli D, Dutta-Gupta A: **Ecdysteroid-mediated expression of hexamerin (arylphorin) in the rice moth, *Corcyra cephalonica*.** *J Insect Physiol* 2010, **56:**1224-1231.

793. Telfer WH, Keim PS, Law JH: **Arylphorin, a new protein from *Hyalophora cecropia*: Comparisons with calliphorin and manducin.** *Insect Biochemistry* 1983, **13:**601-613.

794. Willott E, Wang XY, Wells MA: **cDNA and gene sequence of *Manduca sexta* arylphorin, an aromatic amino acid-rich larval serum protein. Homology to arthropod hemocyanins.** *J Biol Chem* 1989, **264:**19052-19059.

795. Shimada T, Kurimoto Y, Kobayashi M: **Phylogenetic relationship of silkmoths inferred from sequence data of the arylphorin gene.** *Mol Phylogen Evol* 1995, **4:**223-234.

796. Fujii T, Sakurai H, Izumi S, Tomino S: **Structure of the gene for the arylphorin-type storage protein SP 2 of *Bombyx mori*.** *J Biol Chem* 1989, **264:**11020-11025.

797. Yano K-i, Sakurai MT, Izumi S, Tomino S: ***vitellogenin* gene of the silkworm, *Bombyx mori*: Structure and sex-dependent expression.** *FEBS Lett* 1994, **356:**207-211.

798. Guidugli KR, Piulachs M-D, BelléS X, LourençO AP, Simões ZLP: **Vitellogenin expression in queen ovaries and in larvae of both sexes of *Apis mellifera*.** *Arch Insect Biochem Physiol* 2005, **59:**211-218.

799. Brennan MD, Weiner AJ, Goralski TJ, Mahowald AP: **The follicle cells are a major site of vitellogenin synthesis in *Drosophila melanogaster*.** *Dev Biol* 1982, **89:**225-236.

800. Schonbaum CP, Lee S, Mahowald AP: **The *Drosophila* yolkless gene encodes a vitellogenin receptor belonging to the low density lipoprotein receptor superfamily.** *Proceedings of the National Academy of Sciences* 1995, **92:**1485-1489.

801. Richard DS, Rybczynski R, Wilson TG, Wang Y, Wayne ML, Zhou Y, Partridge L, Harshman LG: **Insulin signaling is necessary for vitellogenesis in *Drosophila melanogaster* independent of the roles of juvenile hormone and ecdysteroids: female sterility of the *chico1* insulin signaling mutation is autonomous to the ovary.** *J Insect Physiol* 2005, **51:**455-464.

802. Schonbaum CP, Perrino JJ, Mahowald AP: **Regulation of the vitellogenin receptor during *Drosophila melanogaster* oogenesis.** *Mol Biol Cell* 2000, **11:**511-521.

803. Perera OP, Shirk PD: **cDNA of YP4, a follicular epithelium yolk protein subunit, in the moth, *Plodia interpunctella*.** *Arch Insect Biochem Physiol* 1999, **40:**157-164.

804. Böhni R, Riesgo-Escovar J, Oldham S, Brogiolo W, Stocker H, Andruss BF, Beckingham K, Hafen E: **Autonomous control of cell and organ size by CHICO, a *Drosophila* homolog of vertebrate IRS1–4.** *Cell* 1999, **97:**865-875.

805. Iwami M, Tanaka A, Hano N, Sakurai S: **Bombyxin gene expression in tissues other than brain detected by reverse transcription-polymerase chain reaction (RT-PCR) and in situ hybridization.** *Experientia* 1996, **52:**882-887.

806. Wonglapsuwan M, Miyazaki T, Loongyai W, Chotigeat W: **Characterization and biological activity of the ribosomal protein L10a of the white shrimp: *Fenneropenaeus merguiensis* De Man during vitellogenesis.** *Mar Biotechnol* 2010, **12:**230-240.

807. Wonglapsuwan M, Chotigeat W, Timmons A, McCall K: **RpL10A regulates oogenesis progression in the banana prawn *Fenneropenaeus merguiensis* and *Drosophila melanogaster*.** *Gen Comp Endocrinol* 2011, **173:**356-363.

808. Cramton SE, Laski FA: ***string of pearls* encodes *Drosophila* ribosomal protein S2, has *minute*-like characteristics, and is required during oogenesis.** *Genetics* 1994, **137:**1039-1048.

809. Barrio R, del Arco A, Cabrera HL, Arribas C: **Cloning and analysis of the S2 ribosomal protein cDNA from *Drosophila*.** *Nucleic Acids Res* 1993, **21:**351.

810. Ashok M, Turner C, Wilson TG: **Insect juvenile hormone resistance gene homology with the bHLH-PAS family of transcriptional regulators.** *Proceedings of the National Academy of Sciences* 1998, **95:**2761-2766.

811. Swevers L, Cherbas L, Cherbas P, Iatrou K: ***Bombyx* EcR (BmEcR) and *Bombyx* USP (BmCF1) combine to form a functional ecdysone receptor.** *Insect Biochem Mol Biol* 1996, **26:**217-221.

812. Swevers L, Drevet JR, Lunke MD, Iatrou K: **The silkmoth homolog of the *Drosophila* ecdysone receptor (BI Isoform): Cloning and analysis of expression during follicular cell differentiation.** *Insect Biochem Mol Biol* 1995, **25:**857-866.

813. Oro AE, McKeown M, Evans RM: **The *Drosophila* retinoid X receptor homolog *ultraspiracle* functions in both female reproduction and eye morphogenesis.** *Development* 1992, **115:**449-462.

814. Yao T-P, Forman BM, Jiang Z, Cherbas L, Chen JD, McKeown M, Cherbas P, Evans RM: **Functional ecdysone receptor is the product of *EcR* and *Ultraspiracle* genes.** *Nature* 1993, **366:**476-479.

815. Shea MJ, King DL, Conboy MJ, Mariani BD, Kafatos FC: **Proteins that bind to *Drosophila* chorion *cis*-regulatory elements: a new C[[2]]H[[2]] zinc finger protein and a C[[2]]C[[2]] steroid receptor-like component.** *Genes Dev* 1990, **4:**1128.

816. Buszczak M, Freeman MR, Carlson JR, Bender M, Cooley L, Segraves WA: **Ecdysone response genes govern egg chamber development during mid-oogenesis in *Drosophila*.** *Development* 1999, **126:**4581-4589.

817. Carney GE, Bender M: **The *Drosophila ecdysone receptor* (*EcR*) gene is required maternally for normal oogenesis.** *Genetics* 2000, **154:**1203-1211.

818. Roth GE, Gierl MS, Vollborn L, Meise M, Lintermann R, Korge G: **The *Drosophila* gene *Start1*: a putative cholesterol transporter and key regulator of ecdysteroid synthesis.** *Proceedings of the National Academy of Sciences* 2004, **101:**1601-1606.

819. Terashima J, Bownes M: **A microarray analysis of genes involved in relating egg production to nutritional intake in *Drosophila melanogaster*.** *Cell Death Differ* 2005, **12:**429-440.

820. Freeman MR, Dobritsa A, Gaines P, Segraves WA, Carlson JR: **The *dare* gene: steroid hormone production, olfactory behavior, and neural degeneration in *Drosophila*.** *Development* 1999, **126:**4591-4602.

821. Swevers L, Eystathioy T, Iatrou K: **The orphan nuclear receptors BmE75A and BmE75C of the silkmoth *Bombyx mori*: hornmonal control and ovarian expression.** *Insect Biochem Mol Biol* 2002, **32:**1643-1652.

822. Georgomanolis T, Iatrou K, Swevers L: ***BmCAP*, a silkmoth gene encoding multiple protein isoforms characterized by SoHo and SH3 domains: Expression analysis during ovarian follicular development.** *Insect Biochem Mol Biol* 2009, **39:**892-902.

823. Van Antwerpen R, Law JH: **Immunocytochemical localization of a follicle specific protein of the hawkmoth *Manduca sexta*.** *Tissue Cell* 1993, **25:**885-892.

824. Sato Y, Yamashita O: **Synthesis and secretion of egg-specific protein from follicle cells of the silkworm, *Bombyx mori*.** *Insect Biochemistry* 1991, **21:**233-238.

825. Sato Y, Yamashita O: **Structure and expression of a gene coding for egg-specific protein in the silkworm, *Bombyx mori*.** *Insect Biochemistry* 1991, **21:**495-505.

826. Eystathioy T, Swevers L, Iatrou K: **The orphan nuclear receptor BmHR3A of *Bombyx mori*: hormonal control, ovarian expression and functional properties.** *Mech Dev* 2001, **103:**107-115.

827. Zhang Y, Kunkel JG: **Most egg calmodulin is a follicle cell contribution to the cytoplasm of the *Blattella germanica* oocyte.** *Dev Biol* 1994, **161:**513-521.

828. Andruss BF, Lu AQ, Beckingham K: **Expression of calmodulin in *Drosophila* is highly regulated in a stage- and tissue-specific manner.** *Dev Genes Evol* 1997, **206:**541-545.

829. Brown PT, Herbert P, Woodruff RI: **Vitellogenesis in *Oncopeltus fasciatus*: PLC/IP3, DAG/PK-C pathway triggered by CaM.** *J Insect Physiol* 2010, **56:**1300-1305.

830. Lorca T, Cruzalegui FH, Fesquet D, Cavadore JC, Mery J, Means A, Doree M: **Calmodulin-dependent protein kinase II mediates inactivation of MPF and CSF upon fertilization of *Xenopus* eggs.** *Nature* 1993, **366:**270-273.

831. Li C, Kapitskaya MZ, Zhu J, Miura K, Segraves W, Raikhel AS: **Conserved molecular mechanism for the stage specificity of the mosquito vitellogenic response to ecdysone.** *Dev Biol* 2000, **224:**96-110.

832. Swevers L, Iatrou K: **The orphan receptor BmHNF-4 of the silkmoth *Bombyx mori*: ovarian and zygotic expression of two mRNA isoforms encoding polypeptides with different activating domains.** *Mech Dev* 1998, **72:**3-13.

833. Wilson TG: **A correlation between juvenile hormone deficiency and vitellogenic oocyte degeneration in *Drosophila melanogaster*.** *Roux's Arch Dev Biol* 1982, **191:**257-263.

834. Liu Z, Li X, Prasifka JR, Jurenka R, Bonning BC: **Overexpression of *Drosophila* juvenile hormone esterase binding protein results in anti-JH effects and reduced pheromone abundance.** *Gen Comp Endocrinol* 2008, **156:**164-172.

835. Seino A, Ogura T, Tsubota T, Shimomura M, Nakakura T, Tan A, Mita K, Shinoda T, Nakagawa Y, Shiotsuki T: **Characterization of juvenile hormone epoxide hydrolase and related genes in the larval development of the silkworm *Bombyx mori*.** *Biosci Biotechnol Biochem* 2010, **74:**1421-1429.

836. Orth AP, Tauchman SJ, Doll SC, Goodman WG: **Embryonic expression of juvenile hormone binding protein and its relationship to the toxic effects of juvenile hormone in *Manduca sexta*.** *Insect Biochem Mol Biol* 2003, **33:**1275-1284.

837. Hammock B, Nowock J, Goodman W, Stamoudis V, Gilbert LI: **The influence of hemolymph-binding protein on juvenile hormone stability and distribution in *Manduca sexta* fat body and imaginal discs in vitro.** *Mol Cell Endocrinol* 1975, **3:**167-184.

838. Noriega FG, Ribeiro JM, Koener JF, Valenzuela JG, Hernandez-Martinez S, Pham VM, Feyereisen R: **Comparative genomics of insect juvenile hormone biosynthesis.** *Insect Biochem Mol Biol* 2006, **36:**366-374.

839. Huang X, Warren JT, Buchanan JA, Gilbert LI, Scott MP: ***Drosophila Niemann-Pick Type C-2* genes control sterol homeostasis and steroid**

biosynthesis: a model of human neurodegenerative disease. *Development* 2007, **134:**3733-3742.

840. Godfrey AC, Kupsco JM, Burch BD, Zimmerman RM, Dominski Z, Marzluff WF, Duronio RJ: ***U7* snRNA mutations in *Drosophila* block histone pre-mRNA processing and disrupt oogenesis.** *RNA* 2006, **12:**396-409.

841. Gaziova I, Bonnette PC, Henrich VC, Jindra M: **Cell-autonomous roles of the *ecdysoneless* gene in *Drosophila* development and oogenesis.** *Development* 2004, **131:**2715-2725.

842. König A, Yatsenko AS, Weiss M, Shcherbata HR: **Ecdysteroids affect *Drosophila* ovarian stem cell niche formation and early germline differentiation.** *EMBO J* 2011, **30:**1549-1562.

843. Chavez VM, Marques G, Delbecque JP, Kobayashi K, Hollingsworth M, Burr J, Natzle JE, O'Connor MB: **The *Drosophila disembodied* gene controls late embryonic morphogenesis and codes for a cytochrome P450 enzyme that regulates embryonic ecdysone levels.** *Development* 2000, **127:**4115-4126.

844. Kokoza EB, Belyaeva ES, Zhimulev IF: **Localization of genes *ecs, dor* and *swi* in eight *Drosophila* species.** *Genetica* 1992, **87:**79-85.

845. Sommer B, Oprins A, Rabouille C, Munro S: **The exocyst component Sec5 is present on endocytic vesicles in the oocyte of *Drosophila melanogaster*.** *J Cell Biol* 2005, **169:**953-963.

846. Jha A, Watkins SC, Traub LM: **The apoptotic engulfment protein Ced-6 participates in clathrin-mediated yolk uptake in *Drosophila* egg chambers.** *Mol Biol Cell* 2012, **23:**1742-1764.

847. Murthy M, Schwarz TL: **The exocyst component Sec5 is required for membrane traffic and polarity in the *Drosophila* ovary.** *Development* 2004, **131:**377-388.

848. Culi J, Mann RS: **Boca, an endoplasmic reticulum protein required for wingless signaling and trafficking of LDL receptor family members in *Drosophila*.** *Cell* 2003, **112:**343-354.

849. Lee S, Cooley L: **Jagunal is required for reorganizing the endoplasmic reticulum during *Drosophila* oogenesis.** *J Cell Biol* 2007, **176:**941-952.

850. Mayer-Jaekel RE, Baumgartner S, Bilbe G, Ohkura H, Glover DM, Hemmings BA: **Molecular cloning and developmental expression of the catalytic and 65-kDa regulatory subunits of protein phosphatase 2A in *Drosophila*.** *Mol Biol Cell* 1992, **3:**287-298.

851. Vereshchagina N, Ramel MC, Bitoun E, Wilson C: **The protein phosphatase PP2A-B' subunit Widerborst is a negative regulator of cytoplasmic activated Akt and lipid metabolism in *Drosophila*.** *J Cell Sci* 2008, **121:**3383-3392.

852. Parisi MJ, Gupta V, Sturgill D, Warren JT, Jallon JM, Malone JH, Zhang Y, Gilbert LI, Oliver B: **Germline-dependent gene expression in distant non-gonadal somatic tissues of *Drosophila*.** *BMC Genomics* 2010, **11:**346.

853. Teixeira L, Rabouille C, Rørth P, Ephrussi A, Vanzo NF: ***Drosophila* Perilipin/ADRP homologue Lsd2 regulates lipid metabolism.** *Mech Dev* 2003, **120:**1071-1081.

854. Grönke S, Beller M, Fellert S, Ramakrishnan H, Jäckle H, Kühnlein RP: **Control of fat storage by a *Drosophila* PAT domain protein.** *Curr Biol* 2003, **13:**603-606.

855. Pistillo D, Manzi A, Tino A, Boyl PP, Graziani F, Malva C: **The *Drosophila melanogaster* lipase homologs: a gene family with tissue and developmental specific expression.** *J Mol Biol* 1998, **276:**877-885.

856. Cavaliere V, Donati A, Hsouna A, Hsu T, Gargiulo G: **dAkt kinase controls follicle cell size during *Drosophila* oogenesis.** *Dev Dyn* 2005, **232:**845-854.

857. Andjelkovic M, Jones PF, Grossniklaus U, Cron P, Schier AF, Dick M, Bilbe G, Hemmings BA: **Developmental regulation of expression and activity of multiple forms of the *Drosophila* RAC protein kinase.** *J Biol Chem* 1995, **270:**4066-4075.

858. Leventis PA, Da Sylva TR, Rajwans N, Wasiak S, McPherson PS, Boulianne GL: **Liquid facets-Related (lqfR) is required for egg chamber morphogenesis during *Drosophila* oogenesis.** *PLoS ONE* 2011, **6:**e25466.

859. Lloyd VK, Sinclair DA, Wennberg R, Warner TS, Honda BM, Grigliatti TA: **A genetic and molecular characterization of the *garnet* gene of *Drosophila melanogaster*.** *Genome* 1999, **42:**1183-1193.

860. Birnbaum MJ, Gilbert LI: **Juvenile hormone stimulation of ornithine decarboxylase activity during vitellogenesis in *Drosophila melanogaster*.** *J Comp Physiol B* 1990, **160:**145-151.

861. Vied C, Halachmi N, Salzberg A, Horabin JI: **Antizyme is a target of Sex-lethal in the *Drosophila* germline and appears to act downstream of Hedgehog to regulate Sex-lethal and Cyclin B.** *Dev Biol* 2003, **253:**214-219.

862. Yu J, Zheng Y, Dong J, Klusza S, Deng W-M, Pan D: **Kibra functions as a tumor suppressor protein that regulates Hippo signaling in conjunction with Merlin and Expanded.** *Dev Cell* 2010, **18:**288.

863. Polesello C, Tapon N: **Salvador-warts-hippo signaling promotes *Drosophila* posterior follicle cell maturation downstream of notch.** *Curr Biol* 2007, **17:**1864-1870.

864. Yan Y, Denef N, Tang C, Schüpbach T: ***Drosophila* PI4KIIIalpha is required in follicle cells for oocyte polarization and Hippo signaling.** *Development* 2011, **138:**1697-1703.

865. Chen H-J, Wang C-M, Wang T-W, Liaw G-J, Hsu T-H, Lin T-H, Yu J-Y: **The Hippo pathway controls polar cell fate through Notch signaling during *Drosophila* oogenesis.** *Dev Biol* 2011, **357:**370-379.

866. Serano J, Rubin GM: **The *Drosophila* synaptotagmin-like protein bitesize is required for growth and has mRNA localization sequences within its open reading frame.** *Proceedings of the National Academy of Sciences* 2003, **100:**13368-13373.

867. Lin SC, Lin MH, Horvath P, Reddy KL, Storti RV: **PDP1, a novel *Drosophila* PAR domain bZIP transcription factor expressed in developing mesoderm, endoderm and ectoderm, is a transcriptional regulator of somatic muscle genes.** *Development* 1997, **124:**4685-4696.

868. Ollmann M, Young LM, Di Como CJ, Karim F, Belvin M, Robertson S, Whittaker K, Demsky M, Fisher WW, Buchman A, et al: ***Drosophila p53* is a structural and functional homolog of the tumor suppressor *p53*.** *Cell* 2000, **101:**91-101.

869. Colussi PA, Quinn LM, Huang DCS, Coombe M, Read SH, Richardson H, Kumar S: **Debcl, a proapoptotic Bcl-2 homologue, is a component of the *Drosophila melanogaster* cell death machinery.** *J Cell Biol* 2000, **148:**703-714.

870. Tanner EA, McCall K: **Mitochondrial regulation of cell death in the *Drosophila* ovary.** *Autophagy* 2011, **7:**793-794.

871. Zhang J-Y, Pan M-H, Sun Z-Y, Huang S-J, Yu Z-S, Liu D, Zhao D-H, Lu C: **The genomic underpinnings of apoptosis in the silkworm, *Bombyx mori*.** *BMC Genomics* 2010, **11:**611.

872. Nezis IP, Shravage BV, Sagona AP, Lamark T, Bjorkoy G, Johansen T, Rusten TE, Brech A, Baehrecke EH, Stenmark H: **Autophagic degradation of dBruce controls DNA fragmentation in nurse cells during late *Drosophila melanogaster* oogenesis.** *J Cell Biol* 2010, **190:**523-531.

873. Geisbrecht ER, Montell DJ: **A role for *Drosophila* IAP1-mediated caspase inhibition in Rac-dependent cell migration.** *Cell* 2004, **118:**111-125.

874. Hou YC, Chittaranjan S, Barbosa SG, McCall K, Gorski SM: **Effector caspase Dcp-1 and IAP protein Bruce regulate starvation-induced autophagy during *Drosophila melanogaster* oogenesis.** *J Cell Biol* 2008, **182:**1127-1139.

875. Tanner EA, Blute TA, Brachmann CB, McCall K: **Bcl-2 proteins and autophagy regulate mitochondrial dynamics during programmed cell death in the *Drosophila* ovary.** *Development* 2011, **138:**327-338.

876. Yang X, Dunning KR, Wu LL, Hickey TE, Norman RJ, Russell DL, Liang X, Robker RL: **Identification of perilipin-2 as a lipid droplet protein regulated in oocytes during maturation.** *Reprod Fertil Dev* 2010, **22:**1262-1271.

877. Paige Bass B, Cullen K, McCall K: **The axon guidance gene *lola* is required for programmed cell death in the *Drosophila* ovary.** *Dev Biol* 2007, **304:**771-785.

878. Kockel L, Kerr KS, Melnick M, Brückner K, Hebrok M, Perrimon N: **Dynamic switch of negative feedback regulation in *Drosophila* Akt–TOR signaling.** *PLoS Genet* 2010, **6:**e1000990.

879. Di Fruscio M, Chen T, Bonyadi S, Lasko P, Richard S: **The identification of two *Drosophila* K homology domain proteins. Kep1 and sam are members of the Sam68 family of GSG domain proteins.** *J Biol Chem* 1998, **273:**30122-30130.

880. Nakano Y, Fujitani K, Kurihara J, Ragan J, Usui-Aoki K, Shimoda L, Lukacsovich T, Suzuki K, Sezaki M, Sano Y, et al: **Mutations in the novel membrane protein spinster interfere with programmed cell death and cause neural degeneration in *Drosophila melanogaster*.** *Mol Cell Biol* 2001, **21:**3775-3788.

881. Dorstyn L, Read SH, Quinn LM, Richardson H, Kumar S: **DECAY, a novel *Drosophila* Caspase related to mammalian Caspase-3 and Caspase-7.** *J Biol Chem* 1999, **274:**30778-30783.

882. McCall K, Steller H: **Requirement for DCP-1 Caspase during *Drosophila* oogenesis.** *Science* 1998, **279:**230-234.

883. Chen P, Rodriguez A, Erskine R, Thach T, Abrams JM: **Dredd, a novel effector of the apoptosis activators reaper, grim, and hid in *Drosophila*.** *Dev Biol* 1998, **201:**202-216.

884. Peterson JS, Barkett M, McCall K: **Stage-specific regulation of caspase activity in *Drosophila* oogenesis.** *Dev Biol* 2003, **260:**113-123.

885. Dorstyn L, Colussi PA, Quinn LM, Richardson H, Kumar S: **DRONC, an ecdysone-inducible *Drosophila* caspase.** *Proceedings of the National Academy of Sciences* 1999, **96:**4307-4312.

886. Mitra K, Rikhy R, Lilly M, Lippincott-Schwartz J: **DRP1-dependent mitochondrial fission initiates follicle cell differentiation during *Drosophila* oogenesis.** *J Cell Biol* 2012, **197:**487-497.

887. Jones G, Jones D, Zhou L, Steller H, Chu Y: **Deterin, a new inhibitor of apoptosis from *Drosophila melanogaster*.** *J Biol Chem* 2000, **275:**22157-22165.

888. Sato K, Hayashi Y, Ninomiya Y, Shigenobu S, Arita K, Mukai M, Kobayashi S: **Maternal Nanos represses hid/skl-dependent apoptosis to maintain the germ line in *Drosophila* embryos.** *Proceedings of the National Academy of Sciences* 2007, **104:**7455-7460.

889. Baker DA, Russell S: **Gene expression during *Drosophila melanogaster* egg development before and after reproductive diapause.** *BMC Genomics* 2009, **10:**242.

890. Buszczak M, Lu X, Segraves WA, Chang TY, Cooley L: **Mutations in the *midway* gene disrupt a *Drosophila* acyl Coenzyme A. diacylglycerol acyltransferase.** *Genetics* 2002, **160:**1511-1518.

891. Peterson JS, Bass BP, Jue D, Rodriguez A, Abrams JM, McCall K: **Noncanonical cell death pathways act during *Drosophila* oogenesis.** *Genesis* 2007, **45:**396-404.

892. Harvey NL, Daish T, Mills K, Dorstyn L, Quinn LM, Read SH, Richardson H, Kumar S: **Characterization of the *Drosophila* Caspase, DAMM.** *J Biol Chem* 2001, **276:**25342-25350.

893. Andres AJ, Cherbas P: **Tissue-specific ecdysone responses regulation of the *Drosophila* genes *eip28-29* and *eip40* during larval development.** *Development* 1992, **116:**865-876.

894. Wing JP, Schreader BA, Yokokura T, Wang Y, Andrews PS, Huseinovic N, Dong CK, Ogdahl JL, Schwartz LM, White K, Nambu JR: ***Drosophila* Morgue is an F box/ubiquitin conjugase domain protein important for *grim-reaper* mediated apoptosis.** *Nat Cell Biol* 2002, **4:**451-456.

895. Buckingham M, Liu JL: **U bodies respond to nutrient stress in *Drosophila*.** *Exp Cell Res* 2011, **317:**2835-2844.

896. Bettencourt R, Assefaw-Redda Y, Faye I: **The insect immune protein hemolin is expressed during oogenesis and embryogenesis.** *Mech Dev* 2000, **95:**301-304.

897. Roxstrom-Lindquist K, Faye I: **The *Drosophila* gene *Yippee* reveals a novel family of putative zinc binding proteins highly conserved among eukaryotes.** *Insect Mol Biol* 2001, **10:**77-86.

898. Zou Z, Picheng Z, Weng H, Mita K, Jiang H: **A comparative analysis of serpin genes in the silkworm genome.** *Genomics* 2009, **93:**367-375.

899. Kurama T, Kurata S, Natori S: **Molecular characterization of an insect transferrin and its selective incorporation into eggs during oogenesis.** *Eur J Biochem* 1995, **228:**229-235.

900. Kremer N, Voronin D, Charif D, Mavingui P, Mollereau B, Vavre F: ***Wolbachia* interferes with ferritin expression and iron metabolism in insects.** *PLoS Path* 2009, **5:**e1000630.

901. Thomson TC, Johnson J: **Inducible somatic oocyte destruction in response to rapamycin requires wild-type regulation of follicle cell epithelial polarity.** *Cell Death Differ* 2010, **17:**1717-1727.

902. Theopold U, dal Zotto L, Hultmark D: **FKBP39, a *Drosophila* member of a family of proteins that bind the immunosuppressive drug FK506.** *Gene* 1995, **156:**247-251.

903. Contamine D, Petitjean AM, Ashburner M: **Genetic resistance to viral infection: the molecular cloning of a *Drosophila* gene that restricts infection by the rhabdovirus sigma.** *Genetics* 1989, **123:**525-533.

904. Hedengren M, Asling B, Dushay MS, Ando I, Ekengren S, Wihlborg M, Hultmark D: **Relish, a central factor in the control of humoral but not cellular immunity in *Drosophila*.** *Mol Cell* 1999, **4:**827-837.

905. Theopold U, Samakovlis C, Erdjument-Bromage H, Dillon N, Axelsson B, Schmidt O, Tempst P, Hultmark D: ***Helix pomatia* lectin, an inducer of *Drosophila* immune response, binds to hemomucin, a novel surface mucin.** *J Biol Chem* 1996, **271:**12708-12715.

906. Tanji T, Ohashi-Kobayashi A, Natori S: **Participation of a galactose-specific C-type lectin in *Drosophila* immunity.** *Biochem J* 2006, **396:**127-138.

907. Dedeine F, Vavre F, Fleury F, Loppin B, Hochberg ME, Boulétreau M: **Removing symbiotic *Wolbachia* bacteria specifically inhibits oogenesis in a parasitic wasp.** *Proceedings of the National Academy of Sciences* 2001, **98:**6247-6252.

908. Fast EM, Toomey ME, Panaram K, Desjardins D, Kolaczyk ED, Frydman HM: ***Wolbachia* enhance *Drosophila* stem cell proliferation and target the germline stem cell niche.** *Science* 2011, **334:**990-992.

909. Kotwica J, Larson MK, Bebas P, Giebultowicz JM: **Developmental profiles of PERIOD and DOUBLETIME in *Drosophila melanogaster* ovary.** *J Insect Physiol* 2009, **55:**419-425.

910. Tobback J, Boerjan B, Vandersmissen HP, Huybrechts R: **The circadian clock genes affect reproductive capacity in the desert locust *Schistocerca gregaria*.** *Insect Biochem Mol Biol* 2011, **41:**313-321.

911. Beaver LM, Rush BL, Gvakharia BO, Giebultowicz JM: **Noncircadian regulation and function of clock genes *period* and *timeless* in oogenesis of *Drosophila melanogaster*.** *J Biol Rhythms* 2003, **18:**463-472.

912. Isobe M, Kai H, Kurahashi T, Suwan S, Pitchayawasin-Thapphasaraphong S, Franz T, Tani N, Higashi K, Nishida H: **The molecular mechanism of the termination of insect diapause, Part 1: A timer Protein, TIME-EA4, in the diapause eggs of the silkworm *Bombyx mori* is a metallo-glycoprotein.** *ChemBioChem* 2006, **7:**1590-1598.

913. Goldstein SA, Price LA, Rosenthal DN, Pausch MH: **ORK1, a potassium-selective leak channel with two pore domains cloned from *Drosophila melanogaster* by expression in *Saccharomyces cerevisiae*.** *Proceedings of the National Academy of Sciences* 1996, **93:**13256.

914. Todo T, Ryo H, Yamamoto K, Toh H, Inui T, Ayaki H, Nomura T, Ikenaga M: **Similarity among the *Drosophila* (6-4)photolyase, a human photolyase homolog, and the DNA photolyase-blue-light photoreceptor family.** *Science* 1996, **272:**109-112.

915. Fagotto F: **Yolk degradation in tick eggs: III. Developmentally regulated acidification of the yolk spheres.** *Dev Growth Differ* 1991, **33:**57-66.

916. Gray YH, Sved JA, Preston CR, Engels WR: **Structure and associated mutational effects of the cysteine proteinase (*CP1*) gene of *Drosophila melanogaster*.** *Insect Mol Biol* 1998, **7:**291-293.

917. Yamahama Y, Uto N, Tamotsu S, Miyata T, Yamamoto Y, Watabe S, Takahashi SY: **In vivo activation of pro-form *Bombyx* cysteine protease (BCP) in silkmoth eggs: localization of yolk proteins and BCP, and acidification of yolk granules.** *J Insect Physiol* 2003, **49:**131-140.

918. Ribolla PEM, Daffre S, De Bianchi AG: **Cathepsin B and acid phosphatase activities during *Musca domestica* embryogenesis.** *Insect Biochem Mol Biol* 1993, **23:**217-223.

919. Fialho E, Nakamura A, Juliano L, Masuda H, Silva-Neto MAC: **Cathepsin D-mediated yolk protein degradation is blocked by acid phosphatase inhibitors.** *Arch Biochem Biophys* 2005, **436:**246-253.

920. Ramos IB, Miranda K, de Souza W, Oliveira DMP, Lima APCA, Sorgine MHF, Machado EA: **Calcium-regulated fusion of yolk granules is important for yolk degradation during early embryogenesis of *Rhodnius prolixus* Stahl.** *J Exp Biol* 2007, **210:**138-148.

921. Yamada R, Yamahama Y, Sonobe H: **Release of ecdysteroid-phosphates from egg yolk granules and their dephosphorylation during early embryonic development in silkworm, *Bombyx mori*.** *Zoolog Science* 2005, **22:**187-198.

922. Fagotto F: **Regulation of yolk degradation, or how to make sleepy lysosomes.** *J Cell Sci* 1995, **108:**3645-3647.

923. Ramos I, Gomes F, Koeller CM, Saito K, Heise N, Masuda H, Docampo R, de Souza W, Machado EA, Miranda K: **Acidocalcisomes as calcium- and polyphosphate-storage compartments during embryogenesis of the insect *Rhodnius prolixus* Stahl.** *PLoS ONE* 2011, **6:**e27276.

924. Fialho E, Silveira AB, Masuda H, Silva-Neto MAC: **Oocyte fertilization triggers acid phosphatase activity during *Rhodnius prolixus* embryogenesis.** *Insect Biochem Mol Biol* 2002, **32:**871-880.

925. Arbeitman MN, Fleming AA, Siegal ML, Null BH, Baker BS: **A genomic analysis of *Drosophila* somatic sexual differentiation and its regulation.** *Development* 2004, **131:**2007-2021.

926. Zartman JJ, Kanodia JS, Yakoby N, Schafer X, Watson C, Schlichting K, Dahmann C, Shvartsman SY: **Expression patterns of cadherin genes in *Drosophila* oogenesis.** *Gene Expr Patterns* 2009, **9:**31-36.

927. Kallijärvi J, Stratoulias V, Virtanen K, Hietakangas V, Heino TI, Saarma M: **Characterization of *Drosophila* GDNF Receptor-Like and evidence for its evolutionarily conserved Interaction with neural cell adhesion molecule (NCAM)/FasII.** *PLoS ONE* 2012, **7:**e51997.

928. Kendirgi F, Swevers L, Iatrou K: **An ovarian follicular epithelium protein of the silkworm (*Bombyx mori*) that associates with the vitelline membrane and contributes to the structural integrity of the follicle.** *FEBS Lett* 2002, **524:**59-68.

929. Sdralia N, Swevers L, Iatrou K: **BmVMP90, a large vitelline membrane protein of the domesticated silkmoth *Bombyx mori*, is an essential component of the developing ovarian follicle.** *Insect Biochem Mol Biol* 2012, **42:**717-727.

930. Gargiulo G, Gigliotti S, Malva C, Graziani F: **Cellular specificity of expression and regulation of *Drosophila* vitelline membrane protein 32E gene in the follicular epithelium: identification of *cis*-acting elements.** *Mech Dev* 1991, **35:**193-203.

931. Jagadeeshan S, Singh RS: **Rapid evolution of outer egg membrane proteins in the *Drosophila melanogaster* subgroup: a case of ecologically driven evolution of female reproductive traits.** *Mol Biol Evol* 2007, **24:**929-938.

932. Popodi E, Minoo P, Burke T, Waring GL: **Organization and expression of a second chromosome follicle cell gene cluster in *Drosophila*.** *Dev Biol* 1988, **127:**248-256.

933. Mindrinos MN, Scherer LJ, Garcini FJ, Kwan H, Jacobs KA, Petri WH: **Isolation and chromosomal location of putative vitelline membrane genes in *Drosophila melanogaster*.** *EMBO J* 1985, **4:**147-153.

934. Burke T, Waring GL, Popodi E, Minoo P: **Characterization and sequence of follicle cell genes selectively expressed during vitelline membrane formation in *Drosophila*.** *Dev Biol* 1987, **124:**441-450.

935. Fakhouri M, Elalayli M, Sherling D, Hall JD, Miller E, Sun X, Wells L, LeMosy EK: **Minor proteins and enzymes of the *Drosophila* eggshell matrix.** *Dev Biol* 2006, **293:**127-141.

936. Kim C, Han K, Kim J, Yi JS, Kim C, Yim J, Kim YJ, Kim-Ha J: **Femcoat, a novel eggshell protein in *Drosophila*: functional analysis by double stranded RNA interference.** *Mech Dev* 2002, **110:**61.

937. Elalayli M, Hall JD, Fakhouri M, Neiswender H, Ellison TT, Han Z, Roon P, Lemosy EK: **Palisade is required in the *Drosophila* ovary for assembly and function of the protective vitelline membrane.** *Dev Biol* 2008, **319:**359-369.

938. Schlichting K, Wilsch-Brauninger M, Demontis F, Dahmann C: **Cadherin Cad99C is required for normal microvilli morphology in *Drosophila* follicle cells.** *J Cell Sci* 2006, **119:**1184-1195.

939. Liu RHS: **Identification and characterization of interaction partners of *Drosophila* cadherin 99C.** University of Toronto, Department of Cell and Systems Biology; 2010.

940. Alatortsev VE: **New genes for vitelline membrane proteins in *Drosophila*.** *Mol Biol* 2006, **40:**330-332.

941. Papantonis A, Vanden Broeck J, Lecanidou R: **Architectural factor HMGA induces promoter bending and recruits C/EBP and GATA during silkmoth chorion gene regulation.** *Biochem J* 2008, **416:**85-97.

942. Xu Y, Fu Q, Li S, He N: **Silkworm egg proteins at the germ-band formation stage and a functional analysis of BmEP80 protein.** *Insect Biochem Mol Biol* 2011, **41:**572-581.

943. Papantonis A, Tsatsarounos S, Broeck JV, Lecanidou R: **CHD1 assumes a central role during follicle development.** *J Mol Biol* 2008, **383:**957-969.

944. Tootle TL, Williams D, Hubb A, Frederick R, Spradling A: ***Drosophila* eggshell production: identification of new genes and coordination by Pxt.** *PLoS ONE* 2011, **6:**e19943.

945. Hsu T, Gogos JA, Kirsh SA, Kafatos FC: **Multiple zinc finger forms resulting from developmentally regulated alternative splicing of a transcription factor gene.** *Science* 1992, **257:**1946.

946. Yu. Mantrova E, Hsu T: **Down-regulation of transcription factor CF2 by *Drosophila* Ras/MAP kinase signaling in oogenesis: cytoplasmic retention and degradation.** *Genes Dev* 1998, **12:**1166-1175.

947. Allan C S: **The organization and amplification of two chromosomal domains containing *Drosophila* chorion genes.** *Cell* 1981, **27:**193-201.

948. Parks S, Wakimoto B, Spradling A: **Replication and expression of an X-linked cluster of *Drosophila* chorion genes.** *Dev Biol* 1986, **117:**294-305.

949. Spradling AC, Mahowald AP: **Amplification of genes for chorion proteins during oogenesis in *Drosophila melanogaster*.** *Proceedings of the National Academy of Sciences* 1980, **77:**1096-1100.

950. Andersson S, Lambertsson A: **Characterization of a novel *Minute*-locus in *Drosophila melanogaster*: a putative ribosomal protein gene.** *Heredity* 1990, **65:**51-57.

951. Waring GL, Hawley RJ, Schoenfeld T: **Multiple proteins are produced from the *dec-1* eggshell gene in *Drosophila* by alternative RNA splicing and proteolytic cleavage events.** *Dev Biol* 1990, **142:**1-12.

952. Bauer BJ, Waring GL: **7C female sterile mutants fail to accumulate early eggshell proteins necessary for later chorion morphogenesis in *Drosophila*.** *Dev Biol* 1987, **121:**349-358.

953. Leclerc RF, Regier JC: **Evolution of chorion gene families in lepidoptera: characterization of 15 cDNAs from the gypsy moth.** *J Mol Evol* 1994, **39:**244-254.

954. Hibner BL, Burke WD, Lecanidou R, Rodakis GC, Eickbush TH: **Organization and expression of three genes from the silkmoth early chorion locus.** *Dev Biol* 1988, **125:**423-431.

955. Regier JC, Weigmann BM, Leclerc RF, Friedlander TP: **Loss of phylogenetic information in chorion gene families of *Bombyx mori* gene conversion.** *Mol Biol Evol* 1994, **11:**72-87.

956. Herraiz A, Chauvigne F, Cerda J, Belles X, Piulachs MD: **Identification and functional characterization of an ovarian aquaporin from the cockroach**

***Blattella germanica* L. (Dictyoptera, Blattellidae).** *J Exp Biol* 2011, **214:**3630-3638.

957. Apel I, Moshelion M, Heifetz Y: **Water channels, oviduct osmolarity and egg activation in *Drosophila*.** *Program and Abstracts 49th Annual Drosophila Research Conference, San Diego, CA, 2008* 2008**:**264C.

958. Cui J, Sackton KL, Horner VL, Kumar KE, Wolfner MF: **Wispy, the *Drosophila* homolog of GLD-2, is required during oogenesis and egg activation.** *Genetics* 2008, **178:**2017-2029.

959. Kirov N, Shtilbans A, Rushlow C: **Isolation and characterization of a new gene encoding a member of the HIRA family of proteins from *Drosophila melanogaster*.** *Gene* 1998, **212:**323-332.

960. Lee H-G, Seong C-S, Kim Y-C, Davis RL, Han K-A: **Octopamine receptor OAMB is required for ovulation in *Drosophila melanogaster*.** *Dev Biol* 2003, **264:**179-190.

961. Lee H-G, Rohila S, Han K-A: **The Octopamine receptor OAMB mediates ovulation via Ca2+/Calmodulin-dependent protein kinase II in the *Drosophila* oviduct epithelium.** *PLoS ONE* 2009, **4:**e4716.

962. Monastirioti M: **Distinct octopamine cell population residing in the CNS abdominal ganglion controls ovulation in *Drosophila melanogaster*.** *Dev Biol* 2003, **264:**38-49.

963. Roman G, Meller V, Wu KH, Davis RL: **The *opt1* gene of *Drosophila melanogaster* encodes a proton-dependent dipeptide transporter.** *Am J Physiol Cell Physiol* 1998, **275:**C857-C869.

964. Amrein H, Axel R: **Genes expressed in neurons of adult male *Drosophila*.** *Cell* 1997, **88:**459-469.

965. Dzitoyeva S, Dimitrijevic N, Manev H: **Identification of a novel *Drosophila* gene, *beltless*, using injectable embryonic and adult RNA interference (RNAi).** *BMC Genomics* 2003, **4:**33.

966. Gan Q, Chepelev I, Wei G, Tarayrah L, Cui K, Zhao K, Chen X: **Dynamic regulation of alternative splicing and chromatin structure in *Drosophila* gonads revealed by RNA-seq.** *Cell Res* 2010, **20:**763-783.

967. Zhu J, Busche JM, Zhang X: **Identification of juvenile hormone target genes in the adult female mosquitoes.** *Insect Biochem Mol Biol* 2010, **40:**23-29.

968. Beck Y, Pecasse F, Richards G: ***Kruppel-homolog* is essential for the coordination of regulatory gene hierarchies in early *Drosophila* development.** *Dev Biol* 2004, **268:**64-75.

969. Kim-Ha J, Kim J, Kim Y-J: **Requirement of RBP9, a *Drosophila* Hu homolog, for regulation of cystocyte differentiation and oocyte determination during oogenesis.** *Mol Cell Biol* 1999, **19:**2505-2514.
