# Supplementary material for: Unscrambling butterfly oogenesis
Source: BMC Genomics. 2013 Apr 26;14:283. doi: 10.1186/1471-2164-14-283 (PMC3654919; doi:10.1186/1471-2164-14-283)
Supplement: Additional file 3 — Overview of the primer pair properties and performance in qPCR conditions. Gives an overview of the forward and reverse primers designed for qPCR of a set of 19 oogenesis and 3 housekeeping genes. Efficiency and R2 values are provided for each of the primers. [file 1471-2164-14-283-S3.pdf]

Additional file 3 - Overview of the primer pair properties and performance in qPCR conditions

An overview of the forward and reverse primers designed for qPCR of a set of 19 genes (originally identified in the *Drosophila melanogaster* literature as important during oogenesis and found to be expressed by *Pararge aegeria* as well during oogenesis on the basis of the transcriptome), and 3 housekeeping genes used as reference genes (R). A fourth housekeeping gene, mitochondrial *COI* was eventually excluded from the reference gene candidates for further analyses despite exhibiting good primer performance due to an observation of an unexpectedly early Cq in NRT controls (<30) which may be indicative of residual mitochondrial DNA in the cDNA preparation. Efficiency and R^2 values determined by CFX Manager are given for each of the primers. Experimental IDs (note, G12 absent) provide a cross-reference to the raw data in Additional file 4.

Exp ID	Target	Primer ID	5'-3' Sequence	Tm (°C)	Amplicon (bp)	Efficiency	R^2
R1	*mt:CoI*	PaCOI.f	AGCCCCCGATATAGCTTTCC	65	112	98%	0.978
		PaCOI.r	TCCTGTTCCTGCTCCATTTTC	65.2			
R2	*RPII215*	PaRPII5.f	CTAAGATTTCGCCCTGGTTG	63.3	90	102%	0.980
		PaRPII5.r	TCTTCTCGGCAATCTGTTCC	64.2			
R3	*Tbp*	PaTbp.f	GATTCCCGATTAGCTGCAAG	63.5	103	85%	0.959
		PaTbp.r	CACAACTGCCAACCATGTTC	64.1			
R4	*Zw*	PaZw.f	TCAAATCTTTAGCCCATCCTG	62.8	119	92%	0.971
		PaZw.r	CGCGTATTATTCCAAAACTGTC	62.1			
G1	*AGO2*	PaAGO2.f	GTCAAAGGGGTGTGGAAATG	64.1	121	134%	0.917
		PaAGO2.r	TTCGGCGGTGCTACAATC	64.8			
G2	*cad*	Pacad.f	CGGATCCTCACCATTTCAAG	64.3	191	133%	0.890
		Pacad.r	TACTGGATTAGGCTGCGACTG	64.1			
G3	*dpp*	Padpp.f	CTCAAGGAGCCAAAACATAACC	63.4	130	124%	0.968
		Padpp.r	ACTCAGCCTCATCGTCTGTAGC	64.7			
G4	*egl*	Paegl.f	GGGAGGAAAAATTGTGGTTG	63	178	139%	0.931
		Paegl.r	CGTCCAGAATCCTCTCGTTTAG	63.8			
G5	*elav*	Paelav.f	TGGACTATTCGCACAATTCG	63.5	110	96%	0.984
		Paelav.r	TGACGTTGTGAATGGCTCTC	64			
G6	*exu*	Paexu.f	CTCTGCGGCGTATATGATTG	63	142	153%	0.842
		Paexu.r	TTCCAGCTCCGTATGAACAG	62.8			
G7	*Fmr1*	PaFmr1.f	TGATAAGGGGCGAGTTCC	62.6	101	98%	0.976
		PaFmr1.r	CGTCTTGGCAGTCTTGGAG	63.7			
G8	*mnb*	Pamnb.f	GGAACGGAAGCTCTACAATGAC	63.7	131	100%	0.982
		Pamnb.r	TCACAACCTGTCCAAACGAG	63.8			
G9	*nos-like*	Panos-l.f	AGCGTGTTTTCTCCGTTAGC	63.2	184	130%	0.844
		Panos-l.r	TCTGTTCCGCTGGATCTTC	63			
G10	*nos-M*	Panos-m.f	AGCCTCCAAAGAAACCAACAG	64.2	150	84%	0.989
		Panos-m.r	TGTCCACGTATTTCAGCAAAAG	63.7			
G11	*nos-O*	Panos-o.f	GTTTTTGGCGGCTTCAGG	65.6	105	149%	0.772
		Panos-o.r	GAGTTGGGCAGAGTGGACAG	64.9			
G13	*Oda*	PaOda.f	ATCCACGACGACAACAACC	63.6	148	92%	0.998
		PaOda.r	TGCTTATTACCGCTTCTCTCC	62.1			
G14	*aop*	Paop.f	CGAGCTTTTATGGCGGTATC	63.3	153	85%	0.977
		Paop.r	TCTCTCTCGCACCATTTCAG	63.1			
G15	*par-1*	Papar-1.f	ATAGACAAGACCCAGCTCAACC	63.2	197	89%	0.991
		Papar-1.r	TCTTTCATGCGTCCGTGTAG	63.8			
G16	*piwi*	Papiwi.f	CATCCCATCGCACAACTG	64	123	92%	0.997
		Papiwi.r	CAACCACTCCAGAATCACCTC	63.5			
G17	*Cbz*	PaCbZ.f	GACACACTGCACACACCATTC	64.3	178	71%	0.979
		PaCbZ.r	CTTGGAACGGCACTTTATCG	64.3			
G18	*stau*	Pastau.f	GGCCAACAGTAAAGAGAAGACC	62.8	151	72%	0.989
		Pastau.r	TTCCTCAGTGTCACCCAATC	62.6			
G19	*yl*	Payl.f	GCACCACCTTCAACAGAAAC	62.2	194	68%	0.971
		Payl.r	GCAGTGACCTCCAACCATC	63.3			
G20	*Vg*	PaVg.f	AAACTGCCCACCGTTTCTAC	62.8	142	69%	0.984
		PaVg.r	TGTGCTTTGTGACTGTCTTCC	62.9			
