# Supplementary material for: Unscrambling butterfly oogenesis
Source: BMC Genomics. 2013 Apr 26;14:283. doi: 10.1186/1471-2164-14-283 (PMC3654919; doi:10.1186/1471-2164-14-283)
Supplement: Additional file 4 — Data generated by the CFX96 qPCR experiments. Details the measurements from a total of 8 96-well white plates. Cq are given for each gene of interest or reference gene. [file 1471-2164-14-283-S4.pdf]

Additional file 4 - Data generated by the CFX96 qPCR experiments

Measurements from a total of 8 96-well white plates. Each plate included Negative, Positive GAPDH Controls and Reference genes in triplicates and NTC/NRT controls for every gene of interest in duplicates. Quantification cycle values (Cq; the fractional cycle number where fluorescence increases above the threshold) are given for each gene of interest or reference gene (Gx or Rx in the Target column). The initial reaction ID used by CFX manager software is also included, specifying the type and grouping of reactions. R1 NRT controls produced low Cq values (<30). R1 (*mt:COI*) data was therefore excluded from further analyses. Test samples are indicated as Unkn (i.e. Unknown).

Plate/Run	Egg Experiments: Reaction ID	Target	Replicates Cq1	Cq2	Cq3	Ovary Experiments: Reaction ID	Target	Replicates Cq1	Cq2	Cq3
	NRT-26	G1	NEG	NEG		NRT-26	G1	37.70	38.29	
	NTC-16	G1	NEG	NEG		NTC-16	G1	NEG	NEG	
	Unkn-05	G1	23.91	23.98	23.85	Unkn-05	G1	25.12	25.11	25.11
	NRT-27	G2	35.29	39.36		NRT-27	G2	30.40	30.40	
	NTC-17	G2	36.08	36.41		NTC-17	G2	35.14	35.58	
	Unkn-06	G2	25.02	24.86	24.70	Unkn-06	G2	26.15	26.38	26.41
	NRT-28	G3	35.67	36.59		NRT-28	G3	30.89	30.98	
	NTC-18	G3	NEG	NEG		NTC-18	G3	38.82	NEG	
	Unkn-07	G3	31.25	30.65	30.76	Unkn-07	G3	28.07	28.09	28.20
	NRT-29	G4	36.06	35.26		NRT-29	G4	29.85	30.06	
	NTC-19	G4	NEG	NEG		NTC-19	G4	NEG	NEG	
	Unkn-08	G4	23.43	23.31	23.23	Unkn-08	G4	23.87	23.82	23.80
	NRT-30	G5	36.80	36.91		NRT-30	G5	30.43	30.42	
	NTC-20	G5	NEG	NEG		NTC-20	G5	39.44	NEG	
	Unkn-09	G5	22.81	22.82	22.80	Unkn-09	G5	23.40	23.41	23.55
	NRT-31	G6	NEG	NEG		NRT-31	G6	NEG	39.79	
1	NTC-21	G6	38.18	NEG		NTC-21	G6	NEG	39.27	
	Unkn-10	G6	22.33	22.32	22.16	Unkn-10	G6	23.96	23.94	23.83
	Neg Ctrl-11	GAPDH	38.46	NEG	38.71	Neg Ctrl-11	GAPDH	38.12	38.12	39.78
	Pos Ctrl-32	GAPDH	14.79	14.16	14.01	Pos Ctrl-32	GAPDH	16.89	17.23	15.71
	NRT-22	R1	28.62	28.69		NRT-22	R1	25.74	26.01	
	NTC-12	R1	NEG	NEG		NTC-12	R1	NEG	39.58	
	Unkn-01	R1	17.30	17.27	17.13	Unkn-01	R1	18.02	17.81	18.00
	NRT-23	R2	36.79	35.02		NRT-23	R2	30.25	30.60	
	NTC-13	R2	39.07	39.08		NTC-13	R2	36.72	37.48	
	Unkn-02	R2	22.46	22.50	22.52	Unkn-02	R2	23.74	23.75	23.64
	NRT-24	R3	NEG	NEG		NRT-24	R3	37.24	NEG	
	NTC-14	R3	NEG	NEG		NTC-14	R3	36.03	NEG	
	Unkn-03	R3	23.31	23.25	23.25	Unkn-03	R3	24.10	24.16	24.15
	NRT-25	R4	34.92	35.51		NRT-25	R4	30.44	30.04	
	NTC-15	R4	NEG	NEG		NTC-15	R4	NEG	39.31	
	Unkn-04	R4	27.61*	22.33	22.46	Unkn-04	R4	23.48	23.40	23.60
	NRT-26	G7	36.28	35.78		NRT-26	G7	31.44	31.17	
	NTC-16	G7	37.23	36.02		NTC-16	G7	35.66	35.69	
	Unkn-05	G7	23.75	23.74	23.73	Unkn-05	G7	23.49	23.53	23.48
	NRT-27	G8	36.01	NEG		NRT-27	G8	30.72	30.71	
	NTC-17	G8	NEG	38.13		NTC-17	G8	38.00	NEG	
	Unkn-06	G8	23.47	23.12	23.25	Unkn-06	G8	24.16	24.27	24.04
	NRT-28	G9	NEG	NEG		NRT-28	G9	NEG	NEG	
	NTC-18	G9	38.01	NEG		NTC-18	G9	NEG	NEG	
	Unkn-07	G9	20.47	20.64	20.66	Unkn-07	G9	21.21	21.23	21.20
	NRT-29	G10	38.11	37.44		NRT-29	G10	38.99	NEG	
	NTC-19	G10	39.54	38.38		NTC-19	G10	38.84	37.62	
	Unkn-08	G10	22.58	22.42	22.50	Unkn-08	G10	22.22	22.14	22.24
	NRT-30	G11	36.92	37.67		NRT-30	G11	39.30	37.57	
	NTC-20	G11	36.34	37.70		NTC-20	G11	NEG	NEG	
2	Unkn-09	G11	24.33	24.34	24.46	Unkn-09	G11	25.45	25.27	25.46
	Neg Ctrl-11	GAPDH	36.48	37.80	37.01	Neg Ctrl-11	GAPDH	39.10	37.50	NEG
	Pos Ctrl-32	GAPDH	16.25	15.55	16.98	Pos Ctrl-32	GAPDH	17.17	17.59	18.93
	NRT-22	R1	29.34	30.02		NRT-22	R1	25.59	25.89	
	NTC-12	R1	NEG	37.33		NTC-12	R1	NEG	NEG	
	Unkn-01	R1	17.48	17.62	17.67	Unkn-01	R1	17.78	17.99	17.93
	NRT-23	R2	34.98	39.19		NRT-23	R2	30.39	30.44	
	NTC-13	R2	37.00	36.59		NTC-13	R2	39.07	37.71	
	Unkn-02	R2	22.96	22.84	22.90	Unkn-02	R2	23.81	23.57	23.76
	NRT-24	R3	37.84	37.03		NRT-24	R3	NEG	NEG	
	NTC-14	R3	NEG	NEG		NTC-14	R3	NEG	39.38	
	Unkn-03	R3	23.57	23.52	23.54	Unkn-03	R3	24.05	24.20	24.15
	NRT-25	R4	39.20	36.49		NRT-25	R4	30.36	29.98	
	NTC-15	R4	NEG	37.49		NTC-15	R4	NEG	37.64	
	Unkn-04	R4	22.79	22.91	23.01	Unkn-04	R4	23.46	23.60	23.55

Plate/Run	Egg Experiments: Reaction ID	Target	Replicates Cq1	Cq2	Cq3	Ovary Experiments: Reaction ID	Target	Replicates Cq1	Cq2	Cq3
	NRT-26	G13	38.62	NEG		NRT-26	G13	39.21	39.20	
	NTC-16	G13	39.29	39.98		NTC-16	G13	39.12	31.56	
	Unkn-05	G13	24.42	24.41	24.41	Unkn-05	G13	24.45	24.57	24.53
	NRT-27	G14	35.31	NEG		NRT-27	G14	30.73	30.91	
	NTC-17	G14	39.57	38.39		NTC-17	G14	37.75	35.93	
	Unkn-06	G14	28.04	27.80	27.72	Unkn-06	G14	26.47	26.64	26.68
	NRT-28	G15	35.49	35.29		NRT-28	G15	35.41	35.02	
	NTC-18	G15	36.56	35.20		NTC-18	G15	35.16	35.26	
	Unkn-07	G15	25.30	25.17	25.11	Unkn-07	G15	25.62	25.68	25.80
	NRT-29	G16	35.95	NEG		NRT-29	G16	35.96	35.49	
	NTC-19	G16	37.05	35.19		NTC-19	G16	32.35	36.65	
	Unkn-08	G16	22.38	22.37	22.30	Unkn-08	G16	22.78	22.69	22.69
	NRT-30	G17	38.00	36.60		NRT-30	G17	30.41	30.41	
	NTC-20	G17	37.01	NEG		NTC-20	G17	NEG	37.95	
	Unkn-09	G17	23.59	23.62	23.65	Unkn-09	G17	24.00	24.01	24.10
	NRT-31	G18	NEG	NEG		NRT-31	G18	NEG	37.13	
3	NTC-21	G18	NEG	37.33		NTC-21	G18	NEG	NEG	
	Unkn-10	G18	29.03	29.07	29.23	Unkn-10	G18	29.97	29.82	29.77
	Neg Ctrl-11	GAPDH	36.81	36.81	37.19	Neg Ctrl-11	GAPDH	37.98	37.44	34.94
	Pos Ctrl-32	GAPDH	16.40	15.81	16.04	Pos Ctrl-32	GAPDH	17.03	17.12	16.42
	NRT-22	R1	30.21	30.41		NRT-22	R1	25.97	26.23	
	NTC-12	R1	NEG	NEG		NTC-12	R1	NEG	NEG	
	Unkn-01	R1	18.11	17.89	18.07	Unkn-01	R1	17.94	17.78	17.95
	NRT-23	R2	36.89	35.89		NRT-23	R2	30.58	30.74	
	NTC-13	R2	37.99	38.32		NTC-13	R2	37.11	36.08	
	Unkn-02	R2	23.18	23.28	23.25	Unkn-02	R2	23.64	23.76	23.70
	NRT-24	R3	37.26	38.43		NRT-24	R3	NEG	NEG	
	NTC-14	R3	36.90	NEG		NTC-14	R3	NEG	37.86	
	Unkn-03	R3	24.03	24.06	24.08	Unkn-03	R3	24.16	24.18	24.14
	NRT-25	R4	NEG	NEG		NRT-25	R4	30.38	30.56	
	NTC-15	R4	NEG	NEG		NTC-15	R4	37.99	39.73	
	Unkn-04	R4	23.16	23.31	23.30	Unkn-04	R4	23.44	23.38	23.63
	NRT-26	G19	NEG	NEG		NRT-26	G19	NEG	39.28	
	NTC-16	G19	NEG	NEG		NTC-16	G19	38.67	39.32	
	Unkn-05	G19	24.06	24.05	24.13	Unkn-05	G19	24.50	24.47	24.53
	NRT-27	G20	39.92	37.22		NRT-27	G20	30.42	30.77	
	NTC-17	G20	39.10	37.58		NTC-17	G20	37.99	NEG	
	Unkn-06	G20	32.58	32.70	32.66	Unkn-06	G20	19.01	19.17	19.25
	Neg Ctrl-11	GAPDH	36.71	36.61	36.85	Neg Ctrl-11	GAPDH	38.10	38.09	39.11
	Pos Ctrl-21	GAPDH	15.77	15.39	16.02	Pos Ctrl-32	GAPDH	17.01	16.83	15.68
	NRT-22	R1	30.03	30.42		NRT-22	R1	25.80	25.91	
4	NTC-12	R1	NEG	NEG		NTC-12	R1	NEG	NEG	
	Unkn-01	R1	17.65	17.61	17.59	Unkn-01	R1	17.56	17.69	17.50
	NRT-23	R2	35.62	36.41		NRT-23	R2	30.51	30.51	
	NTC-13	R2	38.09	39.25		NTC-13	R2	39.34	NEG	
	Unkn-02	R2	22.93	22.96	22.72	Unkn-02	R2	23.69	23.58	23.53
	NRT-24	R3	NEG	38.42		NRT-24	R3	NEG	NEG	
	NTC-14	R3	38.78	NEG		NTC-14	R3	NEG	36.71	
	Unkn-03	R3	23.61	23.52	23.48	Unkn-03	R3	24.01	24.03	24.05
	NRT-25	R4	39.91	36.83		NRT-25	R4	30.43	30.10	
	NTC-15	R4	NEG	NEG		NTC-15	R4	NEG	NEG	
	Unkn-04	R4	22.89	22.85	22.81	Unkn-04	R4	23.36	23.36	23.25

*Excluded from further analysis.
