# Supplementary material for: Unscrambling butterfly oogenesis
Source: BMC Genomics. 2013 Apr 26;14:283. doi: 10.1186/1471-2164-14-283 (PMC3654919; doi:10.1186/1471-2164-14-283)
Supplement: Additional file 5 — Relative Abundance Data generated by REST. Gives the results from using REST v2.0.13.0 to process Cq measurements and efficiencies in order to estimate relative transcript abundance, and thus compare relative transcript abundance between ovaries and eggs. [file 1471-2164-14-283-S5.pdf]

Additional file 5 - Relative Abundance Data generated by REST

Cq measurements and efficiencies were processed in the Relative Expression Software Tool REST v2.0.13.0 in order to estimate relative transcript abundance, and thus compare transcript abundance between ovaries and eggs. Randomisation (2000 iterations)-based statistics to determine normalised (based on 3 reference genes) and non-normalised relative expression or abundance. Normalised and non-normalised REST statistics are presented, including Standard Error (Std. Error), 95% Confidence Interval (C.I.) and P-value.

Gene	Type	Reaction Efficiency	Normalised Results					Non-Normalised Results				
			Abundance	Std. Error	95% C.I.	P(H1)	Result	Abundance	Std. Error	95% C.I.	P(H1)	Result
RPII215	REF	1	1.158					2.324	2.221 - 2.415	2.180 - 2.442	0.033	UP
Tbp	REF	0.8518	0.85					1.706	1.681 - 1.749	1.637 - 1.752	0.05	UP
Zw	REF	0.9149	1.016					2.039	1.922 - 2.162	1.856 - 2.262	0.035	UP
AGO2	TRG	1	1.145	1.080 - 1.215	1.065 - 1.224	0.08		2.297	2.193 - 2.395	2.189 - 2.408	0.068	
cad	TRG	1	1.364	1.226 - 1.575	1.117 - 1.639	0.021	UP	2.738	2.478 - 3.129	2.240 - 3.258	0	UP
dpp	TRG	1	0.083	0.080 - 0.086	0.079 - 0.088	0.049	DOWN	0.166	0.158 - 0.174	0.155 - 0.182	0	DOWN
egl	TRG	1	0.708	0.664 - 0.761	0.642 - 0.787	0.025	DOWN	1.421	1.323 - 1.500	1.296 - 1.548	0.071	
elav	TRG	0.9624	0.769	0.737 - 0.808	0.728 - 0.828	0.072		1.543	1.488 - 1.644	1.480 - 1.656	0	UP
exu	TRG	1	1.553	1.429 - 1.707	1.385 - 1.765	0	UP	3.117	2.904 - 3.366	2.832 - 3.473	0.035	UP
RPII215	REF	1	1.125					1.757	1.609 - 1.889	1.539 - 1.945	0.035	UP
Tbp	REF	0.8518	0.921					1.438	1.374 - 1.494	1.349 - 1.517	0	UP
Zw	REF	0.9149	0.966					1.509	1.423 - 1.619	1.356 - 1.682	0.075	
Fmr1	TRG	0.9802	0.543	0.525 - 0.559	0.518 - 0.572	0.037	DOWN	0.849	0.837 - 0.865	0.833 - 0.871	0.047	DOWN
mnb	TRG	1	1.175	1.059 - 1.328	0.951 - 1.390	0.122		1.836	1.645 - 2.049	1.510 - 2.187	0	UP
nos-like	TRG	1	0.986	0.938 - 1.065	0.917 - 1.083	0.76		1.54	1.467 - 1.667	1.456 - 1.689	0	UP
nos-M	TRG	0.84	0.533	0.511 - 0.557	0.493 - 0.562	0.045	DOWN	0.833	0.803 - 0.877	0.772 - 0.894	0	DOWN
nos-O	TRG	1	1.295	1.212 - 1.372	1.164 - 1.394	0.026	UP	2.023	1.909 - 2.173	1.784 - 2.186	0	UP
RPII215	REF	1	1.155					1.379	1.318 - 1.431	1.289 - 1.483	0.029	UP
Tbp	REF	0.8518	0.893					1.066	1.051 - 1.082	1.040 - 1.094	0.031	UP
Zw	REF	0.9149	0.97					1.159	1.063 - 1.237	1.048 - 1.333	0	UP
Oda	TRG	0.9181	0.896	0.855 - 0.936	0.840 - 0.954	0.054		1.07	1.026 - 1.108	1.021 - 1.110	0.017	UP
aop	TRG	0.796	0.401	0.354 - 0.447	0.333 - 0.454	0	DOWN	0.479	0.443 - 0.528	0.407 - 0.541	0	DOWN
par-1	TRG	0.894	1.157	1.043 - 1.242	1.015 - 1.279	0.095		1.382	1.291 - 1.480	1.236 - 1.542	0.058	
piwi	TRG	0.9162	1.065	0.999 - 1.133	0.964 - 1.182	0.271		1.272	1.226 - 1.303	1.223 - 1.354	0	UP
CbZ	TRG	0.7049	1.046	1.026 - 1.070	1.014 - 1.076	0.034	UP	1.249	1.215 - 1.286	1.207 - 1.309	0.017	UP
stau	TRG	0.7173	1.252	1.171 - 1.351	1.122 - 1.415	0.042	UP	1.495	1.399 - 1.601	1.346 - 1.655	0	UP
RPII215	REF	1	1.143					1.659	1.521 - 1.798	1.491 - 1.930	0	UP
Tbp	REF	0.8518	0.934					1.355	1.300 - 1.399	1.283 - 1.417	0	UP
Zw	REF	0.9149	0.937					1.36	1.306 - 1.420	1.270 - 1.429	0	UP
yl	TRG	0.6805	0.857	0.789 - 0.913	0.772 - 0.936	0	DOWN	1.244	1.217 - 1.273	1.197 - 1.282	0.045	UP
Vg	TRG	0.6918	0.001	0.001 - 0.001	0.000 - 0.001	0	DOWN	0.001	0.001 - 0.001	0.001 - 0.001	0	DOWN
