# Supplementary material for: Unscrambling butterfly oogenesis
Source: BMC Genomics. 2013 Apr 26;14:283. doi: 10.1186/1471-2164-14-283 (PMC3654919; doi:10.1186/1471-2164-14-283)
Supplement: Additional file 6 — Transcript abundance: Cq - FPKM correlation. Provides the results of the correlation analyses between two measures of transcript abundance: Cq and FPKM-values. [file 1471-2164-14-283-S6.pdf]

Additional file 6 - Cq - FPKM correlation

Linear relationship in the combined egg and ovary transcriptome between the LOG2 transformed values of transcript abundance based upon qPCR results, $(1+E)^{Cq}$, on the x-axis and and the corresponding FPKM values on the Y-axis. Data used is from 19 oogenesis genes and the 3 housekeeping genes. Vitellogenin transcripts were not present in the oocyte and thus not used in the calculation. Pearson regression results (with null hypothesis that correlation is >0): $t_{41} = 2.37$, $P = 0.011$. Analyses were performed in R 2.15.2 for Mac OS X. As can be observed from the graph, the scatter around the regression line is high for the oocyte transcripts.

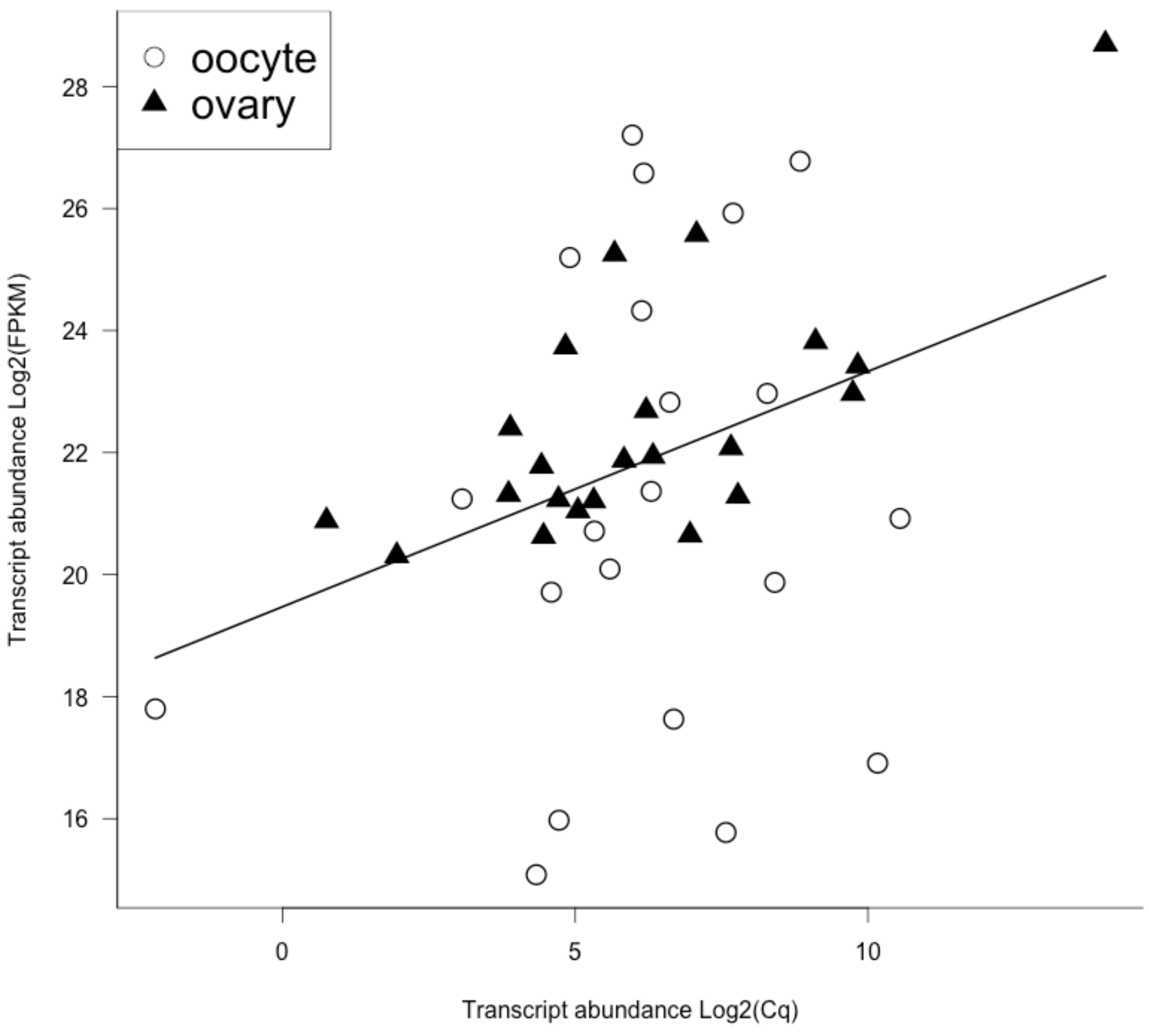
oocyte
ovary
Transcript abundance Log2(FPKM)
28
26
24
22
20
18
16
0
5
10
Transcript abundance Log2(Cq)
