# Supplementary material for: Unscrambling butterfly oogenesis
Source: BMC Genomics. 2013 Apr 26;14:283. doi: 10.1186/1471-2164-14-283 (PMC3654919; doi:10.1186/1471-2164-14-283)
Supplement: Additional file 7 — Mapping of raw RNA-seq reads against egfr and wingless coding sequences as predicted from the draft Pararge aegeria genome. Provides the complete egfr and wingless (wg) CDS fasta information from our unpublished P. aegeria genome. Furthermore, raw RNA-seq reads were mapped against these sequences and coverage determined. [file 1471-2164-14-283-S7.pdf]

Additional file 7 - Mapping of raw RNA-seq reads against *egfr* and *wingless* coding sequences as predicted from the draft *Pararge aegeria* genome

egfr

The 38bp RNA-Seq reads from both ovary and oocyte were mapped against the *egfr* CDS obtained from the draft *P. aegeria* genome using TopHat. However, only reads from the ovary derived RNA successfully mapped, resulting in approximately 7.1x coverage for *egfr* (total number of mapped reads, N=780). Two versions of the *egfr* CDS are provided, which we have labelled as *egfr*-A and *egfr*-B (fasta sequences provided at the end of this Additional file).

The difference between both sequences is that in *egfr*-A the start codon is located further downstream than in *egfr*-B. The shorter *egfr*-A sequence (4194bp) resembles *Bombyx mori egfr* (SilkDB: BGIBMGA000602-TA), whilst *egfr*-B is longer (4344bp) and is based on the available *Danaus plexippus* annotated sequence (GenBank: EHJ79031.1.) The main difference is thus the absence in *egfr*-A of what is labeled exon 1 in *egfr*-B. None of the *P. aegeria* ovary reads mapped to this exon or the associated exon junction (Figure 1).

wingless

The 38bp RNA-Seq reads from both ovary and oocyte were mapped against the *wingless* (*wg*) CDS obtained from the draft *P. aegeria* genome using TopHat. Reads from both the ovary and oocyte derived RNA were successfully mapped resulting in 6.5x and 3.2x coverage respectively (N_{ovary}=201 and N_{egg}=100) (Figure 2). The *P. aegeria* 1176bp *wg* CDS (fasta sequence at the end of this Additional file) had been located in the draft genome by means of *D. plexippus wg* (GenBank: EHJ69660.1).

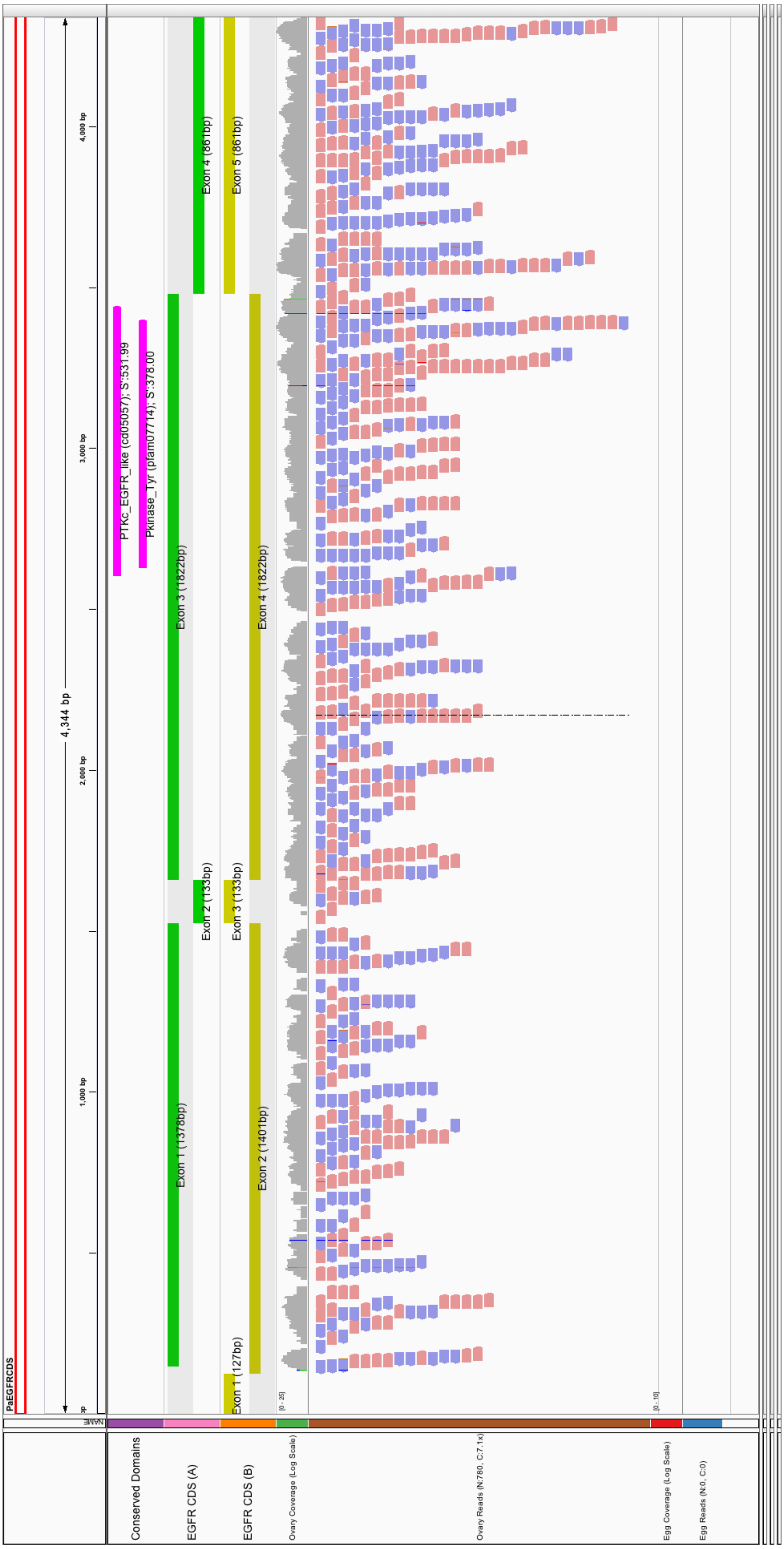
PaEGFRCDS
4,344 bp
1,000 bp
2,000 bp
3,000 bp
4,000 bp
PTKc_EGFR_like (cd05057); S:531.99
Pkinase_Tyr (pfam07714); S:378.00
Exon 1 (1378bp)
Exon 2 (133bp)
Exon 3 (1822bp)
Exon 4 (861bp)
Exon 1 (127bp)
Exon 2 (1401bp)
Exon 3 (133bp)
Exon 4 (1822bp)
Exon 5 (861bp)
Conserved Domains
EGFR CDS (A)
EGFR CDS (B)
Ovary Coverage (Log Scale)
Ovary Reads (N:780, C:7.1x)
Egg Coverage (Log Scale)
Egg Reads (N:0, C:0)

Figure 1 - Overview of mapping raw RNA-seq reads against *egfr* CDS.

The *egfr* coding sequence is displayed in 5’-3’ with exons annotated in green for *egfr*-A and yellow for *egfr*-B (rows 2 and 3 respectively). The conserved domains are annotated in purple with the relevant bitscore (S’) obtained (row 1). Ovary local read coverage is illustrated in row 4 as a grey bar chart with single nucleotide variations (A, T, C, G) highlighted in the relevant nucleotide colour (Green, Red, Blue, Orange respectively). Ovary read mapping is illustrated in row 5, where red or blue indicate a sense or antisense orientated read respectively. Oocyte local read coverage and mapping are also included in rows 6 and 7 but are empty as none of the reads were successfully mapped.

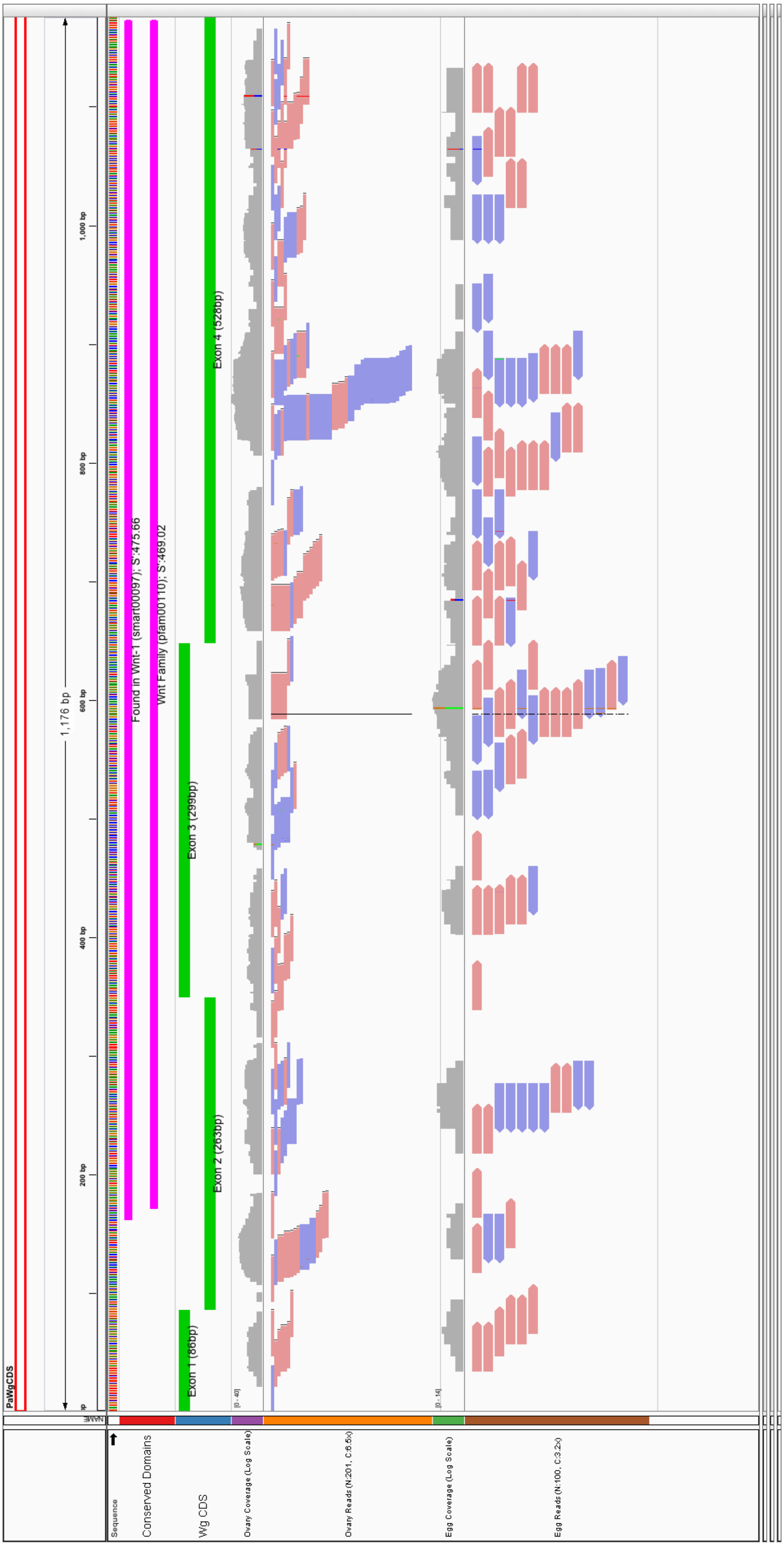
PaWgCDS
1,176 bp
Found in Wnt-1 (smart00097); S:475.66
Wnt Family (pfam00110); S:469.02
Exon 1 (86bp)
Exon 2 (263bp)
Exon 3 (299bp)
Exon 4 (528bp)
Sequence
Conserved Domains
Wg CDS
Ovary Coverage (Log Scale)
Ovary Reads (N:201, C:6.5x)
Egg Coverage (Log Scale)
Egg Reads (N:100, C:3.2x)

Figure 2 - Overview of mapping raw RNA-seq reads against *wg* CDS.

The *wg* coding sequence is displayed in 5'-3' with exons annotated in green on row 2. The conserved domains are annotated in purple with the relevant bitscore (S') obtained (row 1). Ovary and oocyte local coverage is illustrated (rows 3 and 5 respectively) as grey bar charts with single nucleotide variations (A, T, C, G) highlighted in the relevant nucleotide colour (Green, Red, Blue, Orange respectively). Ovary and oocyte read mappings are illustrated in row 4 and 6 respectively, where red indicates a sense orientation and blue antisense orientation.

>*Pararge aegeria epidermal growth factor receptor* A (*egfr* A), predicted CDS from draft genome

ATGTCGGTGCCATCGAACCGCGACACTCACTACAGAAACCTTCGCGATCGCTTCACGAACTGCACCTACG
TCGATGGAAACCTCGAGCTAACATGGCTGCAGAACAAGTCCATGGACCTCACCTTCCTCCAACACATCAG
GGAGGTCACGGGCTATGTTCTAATCGCCTATGTTCAAGTAGAGAACATCAGGCTCCCCCAACTGCAAATA
ATTCGCGGAAGGACTCTGTTCAAGCTTAAAGTTAGAGAGGAAGAGTTCGCGCTACTGGTGACTATGTCTT
CTGCTTTTACGCTGGAACTACCAGCCTTACGTGACGTGCTACGCGGAAGTGTCGGCATATACAACAATTA
CAATCTGTGCCACGTTAAAACAATAAATTGGGACGAAATAATTACGGGTGTGAATGCAACGTACGTTTTC
GTATACACGTTTAACGTACCCGAGAGAGAGTGTCCCGCGTGCCACCCAAACTGCGAAGCTGGTTGCTGGG
GCGATGGCGCGCACAACTGTCAAAAATTTTCCAAAACGAACTGCAGTCCGCAATGTGATCAAGGAAGATG
TTTCGGCCCTAACCCCAGGGATTGTTGTAACACATTTTGCGCTGGTGGATGCACAGGGCCCTTGCCCAGT
CAATGCCTCGCCTGTCGAAACTTCTACGACGAAGGAACGTGCTCGCAAGAATGTCCTCCAATGCAGAAAT
ATAACCCGACGACTTACTCTTGGGAGCCAAATCCTAACGGAAAGTACGCATATGGAGCGACCTGCGTCCG
CAACTGCCCGGAACATCTGTTGAAAGACAGCGGAGCGTGCGTTAGGAGCTGTCCTCCTAACAAAACCGAT
GTGAACGGCGAATGTATCCCTTGCAATGTTACTTGCCCAAAGAAATGCCGCGTCGAAAAACCGATTCACT
CCGGTAATATAGAAAGCTTTAAGGATTGCACAATCATTGACGGTTCGATCGAGATTCTGGAAATGACCTT
CACTGGATTTCAACATGTTAATCCAGACTACTCATTTGGTGAACGTTATTTGAAAATGGAGCCCGATGCG
CTGGAAGTATTCAGCACTGTGACGGAGGTGACTGGGTACCTGAATGTGCAGGCTCACCATCCCAACTTCA
CTAGTCTTTCTTATTTCGAAATCTAGAGGTGATAGGAGGACGCCAAGTGGTGGAGAACTTATTTGCCTC
TCTTTACATTGTCAAAACGTCGCTCAGATCGCTCGGATTAAAATCTCTGAAGAGAGTGAAGTCTGGTGCG
ATTGCGATAATGGAAAACCGCAATCTTTGTTTTGCCGAGAATATTGCGTGGAACAAACTGGTGAAGTCAA
AAGACCACAAGCAAATAATCCAAAAGAATGGGGACCAAAGAACTTGTGAGAAACAGAATCTGGTGTGTGA
TCCTGAATGTTCAGCGGATGGGTGCTGGGGCCCTGGGCCGGATCAATGCCTCTCATGCGAAAACTTTAAA
CTCGAAGGGAACTGCATACAGAATTGTACCTTAGAGGAAGGGTTGTATAAGGTGGGTCCTAAATTATGTA
AAAAATGCCATTCTGAGTGCGCTGACGGATGCACTGGCCCAAGTAGAGCCAACTGTACACGCTGCAAGCA
TGTGCGCGACGGACCTAACTGCATGGCGCATTGTCCTGCCTCCAGATATGTCTCAGAGAACGGAACCTGT
CTACCCTGCCACCCCAACTGTTTCAACGGGTGCACCGGACCGGCTAATACCGTCGGCAAGGGTGCCTGTA
ACTCATGCCAGAAAGCCATCATGAGTATCGAAGCGACAGTTGAAAGCTGTCTCAATGAAGACGAGGCTTG
CCCAGATGGTTATTACAACGAGTGGGTCGGCAATGTCAAGATTTTAGAGGGAAAAGTAAATGTGGTCTGC
AGGAAATGCCACCCATTGTGTATGCAATGCGTTGGATTTGGCATACACGAGCAAGTTTGTAAAGTGTGCA
ATGGTTTTAAAAAAGGCGACCAATGTCAAGATGAATGCCCGGCGGATCACTTCACAGACACAGTCAACCG
TCTTTGTACACCATGTCACCACGAATGTAAAGGATGCACGGGTCCCTCTTCAGTACATTGCATCAAATGC
CAGAATATAAAGGTATTCCTTACCGAAACAACTTTCAATTGTTCTACGAGTTGTCCTAACGACTTGCCCT
ATAAAATTTACTTCGACGAGCAGCTTCAAAATACCATAGATGAACCGTACTGTTCAGCGTCAGCAAAAGG
AGGAATTCCGAACATGGCAACAGCTAAAATTCCCATCGTGCTAGTAGTTATACTCGTGTTTGCATTTATA
CTTTTGGTTATCCTGGCCATCATTGGATACACATGCAGACAAAAAGCCAAAGCGAAAAAGGAAGCGGTGA
AAATGACAAGGGTGCTAACTGGTTGTGAGGACAACGAACCACTCCGACCTACCAATGTTAAACCAAATCT
GGCAAAATTGAGGATCATAAAAGAAGCTGAGCTTCGAAGAGGGGGTATGCTTGGTTTCGGTGCATTCGGA
AAAGTTTACAAAGGTGTCTGGGTTCCCGAAGGAGAAAATGTCAAGATTCCTGTTGCAATTAAAGTTTTGA
AAGAGGGAAGCGGTGCTAGCACTAGCAAAGAATTTCTAGAGGAAGCATATATAATGGCCAGTGTTGAACA
TCCGAATTTACTGCAACTTCTTGCTGTCTGTCTCACTAACCAGATGATGCTGATTACGCAACTCATGCCT
CTCGGATGTCTGTTGGACTATGTCAGGACTCATAAGGAAAAGATCGGGTCGAAGGCATTTTTGAACTGGT
GTACCCAAATAGCGCGCGGAATGGCCTATTTGGAAGACAAAAGATTGGTTCACAGAGACTTGGCAGCTCG
GAATGTTCTAGTGCAAACTCCTAACTGTGTGAAGATAACCGATTTCGGCTTAGCGAAGCTATTGGACATA
AACGAGGATGAATACAAGGCAGCTGGAGGCAAAATGCCCATTAAGTGGCTAGCGTTGGAGTGCGTGCAGC
ACAGAATATTTACACACAAAAGTGATGTCTGGGCTTTCGGCGTAACAATTTGGGAGATATTGAGCTATGG
TGCGCGGCCGTATGCTAACATATCTGCGAGAAATGTACCTGAACTAATTGAGAACGGATTGAAACTGCCC
CAGCCTACTATTTGTACGTTGGACATTTACTGCGTAATGGTTTCATGTTGGATGCTCGACGCAGATAGCC
GACCGACATTCAAGCAGCTCGCCGAAAGATTTGCTGAAATGGCTCGTGACCCCGGACGATACCTGGTCAT
CCCCGGTGACAAGTTTATGCGACTCCCATCCTATTCAACTCAGGATGAAAGGGAAATGATAAGGAGTTTG
TCTTCAGCCATGGATGGCCCCGAGCCAATAGTAGAAGCGGATGAATATCTCCACCCAAAAGCCGATATCA
CGCCCGGCTCTATCTCGCCACTCACCTCCGTCTCGCCTGGATCGCCCGAGAGCAGTGCAATCAAGCCTAT
TGCCTCTCCCTCCTGGGTGAACAACATCAATGGCCAACAAGGTGCGATAGTCGATATCTCGAGGCCAAGT
TGGGAAACAGATCTCATGAAGTACCCGCTCTCTCGCTACACCGTCAGCCCGAACGGCACGGAAATGCGGC
ACTACTATAACAATGGCGCATGCGCTTCCGATTGCTCCAGTAGCTCCCGATATTGTAGTGGCCCCATGAG
GGTCCGAGCTGACGTCACGGAGAGCAGGTTCGACAGTATGTCCAGAAACAAAGAAGCGCAAGTGGGCAAC
CTGAAACTGAACTTGCCCTTGGACGAGGACGATTACCTCATGCCATCGCCGCAGCAAAACAAGAATGCGT
CGACGTATATGGATTTAATTGGCGATGGCGGAGAAGAGCAGGAGGCTAAGGATGATGAAGAGTACAGCGG
ATTCGTGGACTCGGAGCGATGTGTGGATAATCCGGAGTACCTGATGTCCGAACAGGGGGTTCCTCCGCAA
ACGATCGGGATACCCACCGAGCCGGTGGCGCTGGAGTCTTTGGAGACTTGCGAGAGCAGCGCCGGGGATT
CGACGCCGCAGCCGGGCCCGAGCAAATACCAGCCGCAGCGGTCGGTGGAGGAGGAATCGATGTCCGACCA
CGAGTACTACAACGACCTTCAGCGGGAACTCCAACCCCTACGACGCGACGAGACCACGGTGTAA

> *Pararge aegeria epidermal growth factor receptor* B (*egfr* B), predicted CDS from draft genome

ATGTTGGCCCGTGTCTTGTGTCTGTGGCTGTGTCTGAGCCTCGAGGGGGCAGCCGCCGCGCGGGGACAGC
GCGTGCACCACATAGTGCCCGCCCGGCATAAGCACTCCGAGTTCGTCAAGGGAAAAATTTGCATCGGCAC
CAATGGGCGGATGTCGGTGCCATCGAACCGCGACACTCACTACAGAAACCTTCGCGATCGCTTCACGAAC
TGCACCTACGTCGATGGAAACCTCGAGCTAACATGGCTGCAGAACAAGTCCATGGACCTCACCTTCCTCC
AACACATCAGGGAGGTCACGGGCTATGTTCTAATCGCCTATGTTCAAGTAGAGAACATCAGGCTCCCCCA
ACTGCAAATAATTCGCGGAAGGACTCTGTTCAAGCTTAAAGTTAGAGAGGAAGAGTTCGCGCTACTGGTG
ACTATGTCTTCTGCTTTTACGCTGGAACTACCAGCCTTACGTGACGTGCTACGCGGAAGTGTCGGCATAT
ACAACAATTACAATCTGTGCCACGTTAAAACAATAAATTGGGACGAAATAATTACGGGTGTGAATGCAAC
GTACGTTTTCGTATACACGTTTAACGTACCCGAGAGAGAGTGTCCCGCGTGCCACCCAAACTGCGAAGCT
GGTTGCTGGGGCGATGGCGCGCACAACTGTCAAAAATTTTCCAAAACGAACTGCAGTCCGCAATGTGATC
AAGGAAGATGTTTCGGCCCTAACCCCAGGGATTGTTGTAACACATTTTGCGCTGGTGGATGCACAGGGCC
CTTGCCCAGTCAATGCCTCGCCTGTCGAAACTTCTACGACGAAGGAACGTGCTCGCAAGAATGTCCTCCA
ATGCAGAAATATAACCCGACGACTTACTCTTGGGAGCCAAATCCTAACGGAAAGTACGCATATGGAGCGA
CCTGCGTCCGCAACTGCCCGGAACATCTGTTGAAAGACAGCGGAGCGTGCGTTAGGAGCTGTCCTCCTAA
CAAAACCGATGTGAACGGCGAATGTATCCCTTGCAATGTTACTTGCCCAAAGAAATGCCGCGTCGAAAAA
CCGATTCACTCCGGTAATATAGAAAGCTTTAAGGATTGCACAATCATTGACGGTTCGATCGAGATTCTGG
AAATGACCTTCACTGGATTTCAACATGTTAATCCAGACTACTCATTTGGTGAACGTTATTTGAAAATGGA
GCCCGATGCGCTGGAAGTATTCAGCACTGTGACGGAGGTGACTGGGTACCTGAATGTGCAGGCTCACCAT
CCCAACTTCACTAGTCTTTCTTATTTTCGAAATCTAGAGGTGATAGGAGGACGCCAAGTGGTGGAGAACT
TATTTGCCTCTCTTTACATTGTCAAAACGTCGCTCAGATCGCTCGGATTAAAATCTCTGAAGAGAGTGAA
GTCTGGTGCGATTGCGATAATGGAAAACCGCAATCTTTGTTTTGCCGAGAATATTGCGTGGAACAAACTG
GTGAAGTCAAAAGACCACAAGCAAATAATCCAAAAGAATGGGGACCAAAGAACTTGTGAGAAACAGAATC
TGGTGTGTGATCCTGAATGTTCAGCGGATGGGTGCTGGGGCCCTGGGCCGGATCAATGCCTCTCATGCGA
AAACTTTAAACTCGAAGGGAACTGCATACAGAATTGTACCTTAGAGGAAGGGTTGTATAAGGTGGGTCCT
AAATTATGTAAAAAATGCCATTCTGAGTGCGCTGACGGATGCACTGGCCCAAGTAGAGCCAACTGTACAC
GCTGCAAGCATGTGCGCGACGGACCTAACTGCATGGCGCATTGTCCTGCCTCCAGATATGTCTCAGAGAA
CGGAACCTGTCTACCCTGCCACCCCAACTGTTTCAACGGGTGCACCGGACCGGCTAATACCGTCGGCAAG
GGTGCCTGTAACTCATGCCAGAAAGCCATCATGAGTATCGAAGCGACAGTTGAAAGCTGTCTCAATGAAG
ACGAGGCTTGCCCAGATGGTTATTACAACGAGTGGGTCGGCAATGTCAAGATTTTAGAGGGAAAAGTAAA
TGTGGTCTGCAGGAAATGCCACCCATTGTGTATGCAATGCGTTGGATTTGGCATACACGAGCAAGTTTGT
AAAGTGTGCAATGGTTTTAAAAAAGGCGACCAATGTCAAGATGAATGCCCGGCGGATCACTTCACAGACA
CAGTCAACCGTCTTTGTACACCATGTCACCACGAATGTAAAGGATGCACGGGTCCCTCTTCAGTACATTG
CATCAAATGCCAGAATATAAAGGTATTCCTTACCGAAACAACTTTCAATTGTTCTACGAGTTGTCCTAAC
GACTTGCCCTATAAAATTTACTTCGACGAGCAGCTTCAAAATACCATAGATGAACCGTACTGTTCAGCGT
CAGCAAAAGGAGGAATTCCGAACATGGCAACAGCTAAAATTCCCATCGTGCTAGTAGTTATACTCGTGTT
TGCATTTATACTTTTGGTTATCCTGGCCATCATTGGATACACATGCAGACAAAAAGCCAAAGCGAAAAAG
GAAGCGGTGAAAATGACAAGGGTGCTAACTGGTTGTGAGGACAACGAACCACTCCGACCTACCAATGTTA
AACCAAATCTGGCAAAATTGAGGATCATAAAAGAAGCTGAGCTTCGAAGAGGGGGTATGCTTGGTTTCGG
TGCATTCGGAAAAGTTTACAAAGGTGTCTGGGTTCCCGAAGGAGAAAATGTCAAGATTCCTGTTGCAATT
AAAGTTTTGAAAGAGGGAAGCGGTGCTAGCACTAGCAAAGAATTTCTAGAGGAAGCATATATAATGGCCA
GTGTTGAACATCCGAATTTACTGCAACTTCTTGCTGTCTGTCTCACTAACCAGATGATGCTGATTACGCA
ACTCATGCCTCTCGGATGTCTGTTGGACTATGTCAGGACTCATAAGGAAAAGATCGGGTCGAAGGCATTT
TTGAACTGGTGTACCCAAATAGCGCGCGGAATGGCCTATTTGGAAGACAAAAGATTGGTTCACAGAGACT
TGGCAGCTCGGAATGTTCTAGTGCAAACTCCTAACTGTGTGAAGATAACCGATTTCGGCTTAGCGAAGCT
ATTGGACATAAACGAGGATGAATACAAGGCAGCTGGAGGCAAAATGCCCATTAAGTGGCTAGCGTTGGAG
TGCGTGCAGCACAGAATATTTACACACAAAAGTGATGTCTGGGCTTTCGGCGTAACAATTTGGGAGATAT
TGAGCTATGGTGCGCGGCCGTATGCTAACATATCTGCGAGAAATGTACCTGAACTAATTGAGAACGGATT
GAAACTGCCCCAGCCTACTATTTGTACGTTGGACATTTACTGCGTAATGGTTTCATGTTGGATGCTCGAC
GCAGATAGCCGACCGACATTCAAGCAGCTCGCCGAAAGATTTGCTGAAATGGCTCGTGACCCCGGACGAT
ACCTGGTCATCCCCGGTGACAAGTTTATGCGACTCCCATCCTATTCAACTCAGGATGAAAGGGAAATGAT
AAGGAGTTTGTCTTCAGCCATGGATGGCCCCGAGCCAATAGTAGAAGCGGATGAATATCTCCACCCAAAA
GCCGATATCACGCCCGGCTCTATCTCGCCACTCACCTCCGTCTCGCCTGGATCGCCCGAGAGCAGTGCAA
TCAAGCCTATTGCCTCTCCCTCCTGGGTGAACAACATCAATGGCCAACAAGGTGCGATAGTCGATATCTC
GAGGCCAAGTTGGGAAACAGATCTCATGAAGTACCCGCTCTCTCGCTACACCGTCAGCCCGAACGGCACG
GAAATGCGGCACTACTATAACAATGGCGCATGCGCTTCCGATTGCTCCAGTAGCTCCCGATATTGTAGTG
GCCCCATGAGGGTCCGAGCTGACGTCACGGAGAGCAGGTTCGACAGTATGTCCAGAAACAAAGAAGCGCA
AGTGGGCAACCTGAAACTGAACTTGCCCTTGGACGAGGACGATTACCTCATGCCATCGCCGCAGCAAAAC
AAGAATGCGTCGACGTATATGGATTTAATTGGCGATGGCGGAGAAGAGCAGGAGGCTAAGGATGATGAAG
AGTACAGCGGATTCGTGGACTCGGAGCGATGTGTGGATAATCCGGAGTACCTGATGTCCGAACAGGGGGT
TCCTCCGCAAACGATCGGGATACCCACCGAGCCGGTGGCGCTGGAGTCTTTGGAGACTTGCGAGAGCAGC
GCCGGGGATTCGACGCCGCAGCCGGGCCCGAGCAAATACCAGCCGCAGCGGTCGGTGGAGGAGGAATCGA
TGTCCGACCACGAGTACTACAACGACCTTCAGCGGGAACTCCAACCCCTACGACGCGACGAGACCACGGT
GTAA

>*Pararge aegeria wingless* (*wg*), predicted CDS from draft genome

ATGAAGTGGCTGTGCTTGTTTGTGCTGTTTCTGTGTTTGAGGTGCGAGGCGAATAAGCCGAGGCGAGGAC
GAGGCAGCATGTGGTGGGGCATTGCAAAAGCAGGCGAACCAAACAATCTTTCACCCATATCTCCAGGAGT
CCTTTACATGGATCCAGCTGTCCACGCAACTCTCAGGAGGAAGCAGAGAAGGTTAGCGAGAGAGAACCCT
GGGGTTCTTGCAGCCGTATCTAAGGGAGCAAGCATGGCTGTAGCTGAATGCCAGCACCAGTTCAAATATA
GAAGATGGAACTGTTCCACAAGAAATTTTTTAAGAGGGAAAAATCTCTTCGGCAAAATTGTTGACAGAGG
TTGCCGTGAAACAGCGTTTATATACGCGATTACGAGTGCCGGTGTGACGCATGCGGTGTCACGCGCATGC
GCCGAGGGCTCCATCGAGTCCTGCACCTGCGACTACTCGCACGTAGACCGCTCCCCGCACCGCTCCCGCG
CCGCCGCCGCCGCCAACGTGCGTGTCTGGAAGTGGGGGGGCTGCAGCGACAACATCGGCTTCGGCTTCAA
GTTCAGCAGGGAATTCGTCGACACCGGAGAGAGAGGCAAAACCCTCAGAGAGAAAATGAACTTGCACAAC
AACGAAGCCGGCAGGATGCACGTGCAAACGGAGATGCGCCAGGAGTGCAAATGCCACGGTATGTCTGGGT
CCTGCACGGTTAAGACCTGCTGGATGAGGCTGCCGACTTTTCGGTCTGTAGGCGATGCCTTGAAAGATGG
CTTCGACGGGGCGTCGCGGGTAATGATGCCCAATACAGACGTGGAAGCACCGGCTCAGCGGAACGATGCC
GCTCCTCACAGAGTCCCGCGACGAGACCGATACAGATTTCAACTTCGGCCGCATAACCCTGACCACAAAA
CACCCGGGGTCAAGGACCTAGTGTACTTAGAATCATCGCCGGGTTTCTGCGAAAAGAATCCCAGGCTGGG
CATTCCCGGTACGCACGGGCGTGCCTGCAACGATACGAGTATCGGCGTCGACGGCTGCGACCTAATGTGC
TGCGGCCGCGGTTATCGGACCGAGACAATGTTCGTTGTGGAACGATGCAATTGCACGTTCCACTGGTGCT
GCGAAGTTAAATGCAAACTGTGTCGCACGGAAAAAGTAGTTCACACGTGTTTATAG
