# Supplementary material for: Unscrambling butterfly oogenesis
Source: BMC Genomics. 2013 Apr 26;14:283. doi: 10.1186/1471-2164-14-283 (PMC3654919; doi:10.1186/1471-2164-14-283)
Supplement: Additional file 8 — Phylogenetic analysis of Nanos. Provides a phylogenetic analysis of insect Nanos protein sequences. [file 1471-2164-14-283-S8.pdf]

Additional file 8 – Phylogenetic analysis of Nanos protein sequences

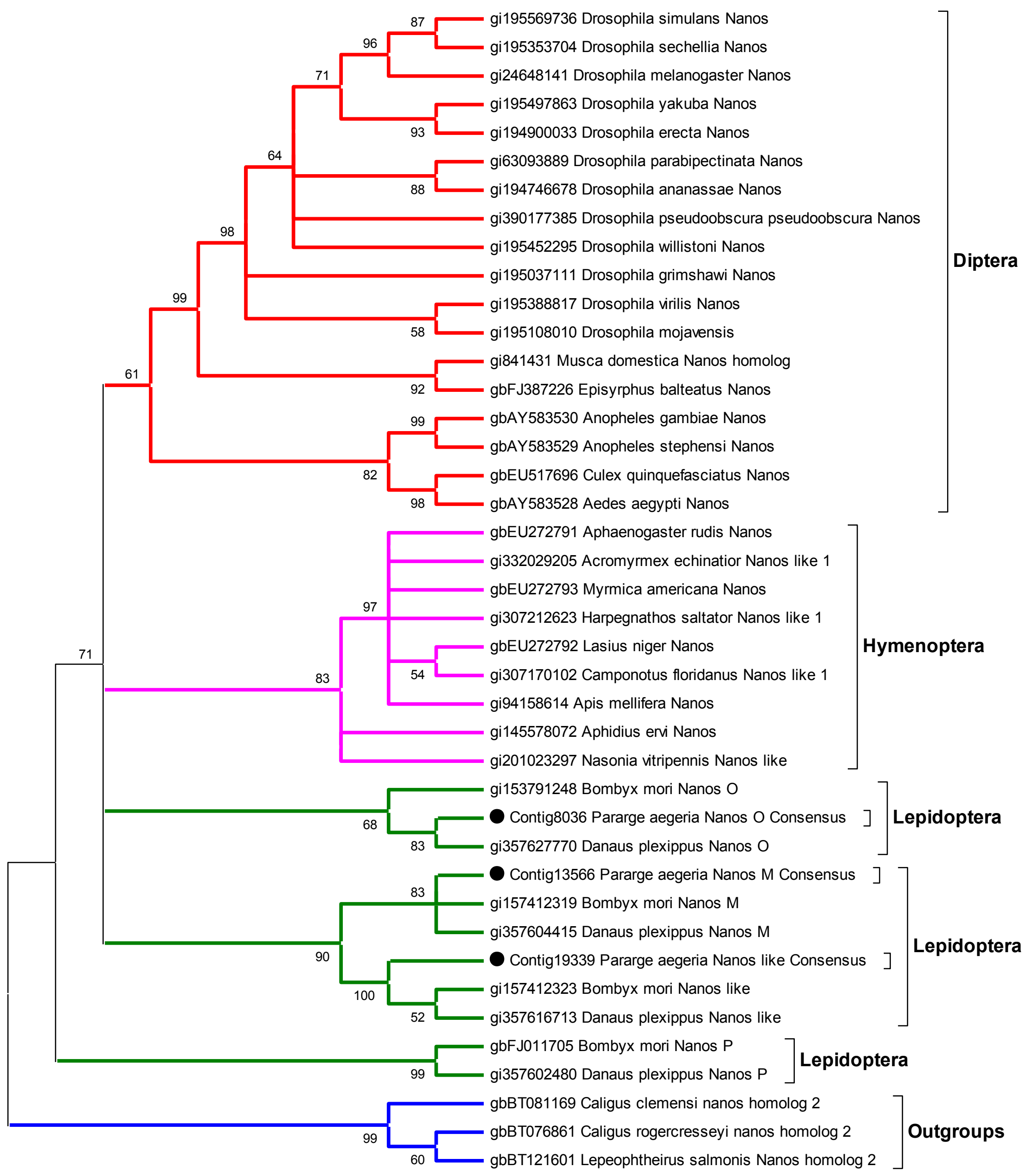

The evolutionary history of (predicted) Nanos protein sequences was inferred using the Neighbor-Joining method. The bootstrap consensus tree inferred from 2000 replicates is taken to represent the evolutionary history of the sequences analysed. Branches corresponding to partitions reproduced in less than 50% bootstrap replicates are collapsed. The percentage of replicate trees in which the associated taxa clustered together in the bootstrap test (2000 replicates) is shown next to the branches. The evolutionary distances were computed using the Poisson correction method and are in the units of the number of amino acid substitutions per site. The analysis involved 41 amino acid sequences. All positions with less than 95% site coverage were eliminated. That is, fewer than 5% alignment gaps, missing data, and ambiguous bases were allowed at any position. There were a total of 76 positions in the final dataset. Evolutionary analyses were conducted in MEGA5. Accession numbers are given in the tree. *Pararge aegeria* sequences are indicated by a dot. All sequences are from Diptera, Hymenoptera and Lepidoptera. The salmon louse *Lepeophtheirus salmonis* and the sea lice *Caligus clemensi* and *Caligus rogercresseyi* were chosen as outgroups.
