# Supplementary material for: Unscrambling butterfly oogenesis
Source: BMC Genomics. 2013 Apr 26;14:283. doi: 10.1186/1471-2164-14-283 (PMC3654919; doi:10.1186/1471-2164-14-283)
Supplement: Additional file 9 — Phylogenetic analyses of both chorion and minor yolk proteins in Lepidoptera. Provides the phylogenetic analyses of both chorion and minor yolk proteins in Lepidoptera. [file 1471-2164-14-283-S9.pdf]

Additional file 9 – Phylogenetic analysis of chorion proteins in Lepidoptera and lipase-like yolk proteins in insects

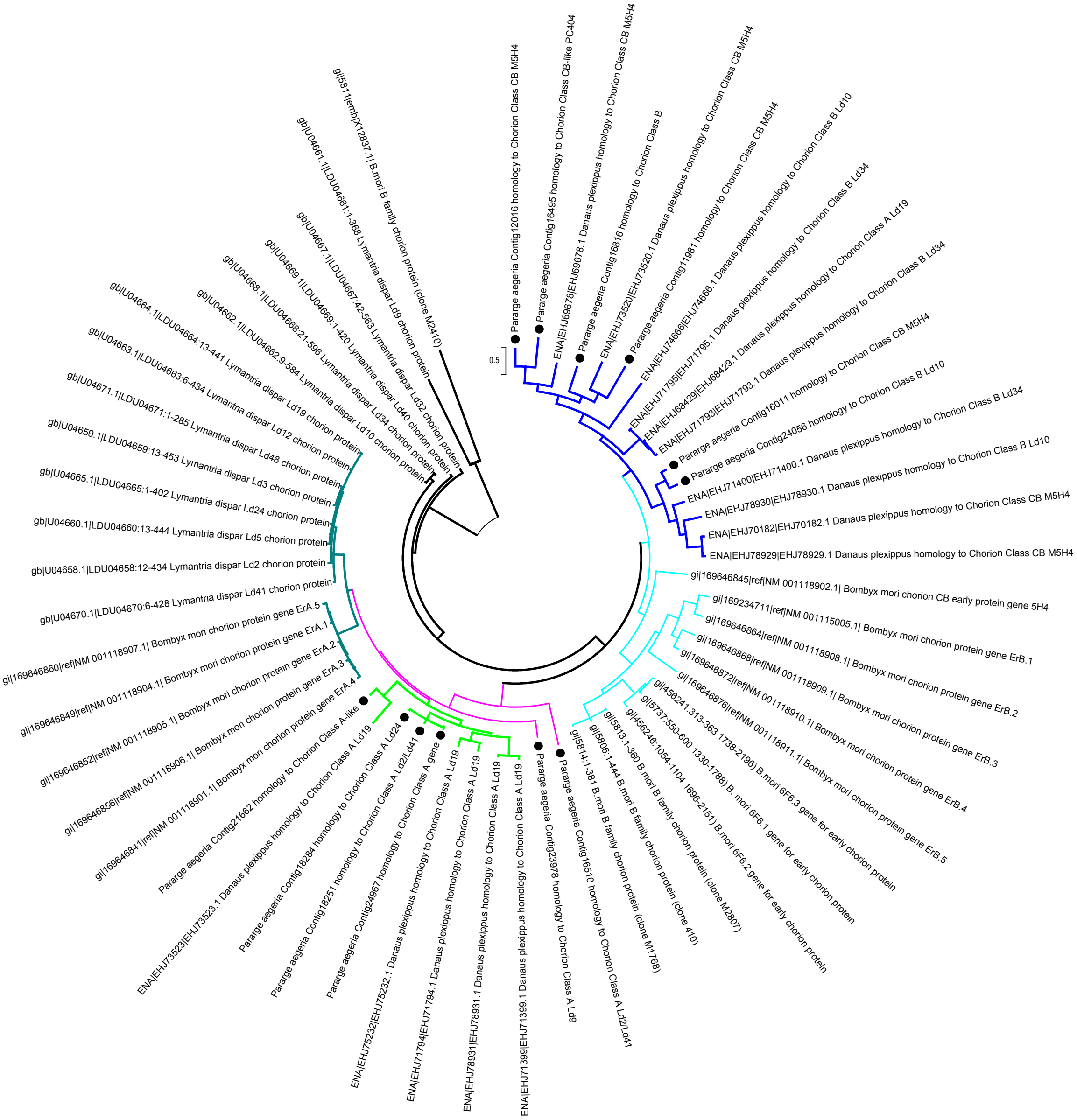

Figure 1 - Phylogenetic analysis of chorion proteins in Lepidoptera

The evolutionary history of the Lepidopteran chorion genes was inferred using the Maximum Likelihood method based on the JTT matrix-based model. *Pararge aegeria* sequences are indicated with a dot. The tree with the highest log likelihood (-11550.3992) is shown. Initial tree(s) for the heuristic search were obtained automatically as follows. When the number of common sites was less than 100 or less than one fourth of the total number of sites, the maximum parsimony method was used; otherwise BIONJ method with MCL distance matrix was used. The tree is drawn to scale, with branch lengths measured in the number of substitutions per site. The analysis involved 58 amino acid sequences. All ambiguous positions were removed for each sequence pair. There were a total of 628 positions in the final dataset. Phylogenetic analyses were conducted in MEGA5. Accession numbers are given in the tree.

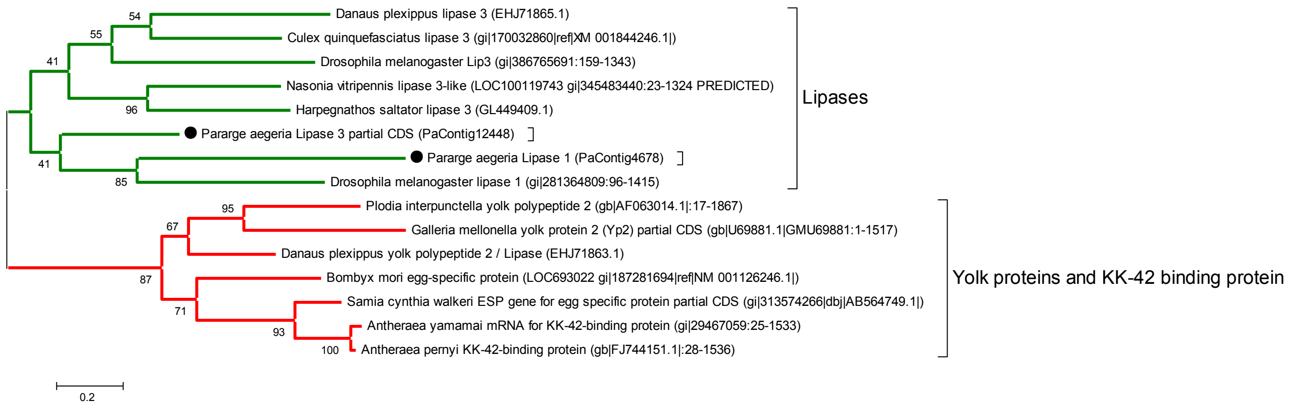

Figure 2 - Phylogenetic analysis of lipase-like proteins in insects

The evolutionary history of minor yolk proteins, Lipase-1 and Lipase-3 was inferred using the Maximum Likelihood method based on the JTT matrix-based model. The tree with the highest log likelihood (-11747.4622) is shown. *Pararge aegeria* sequences are indicated with a dot. The percentage of trees in which the associated taxa clustered together is shown next to the branches. Initial tree(s) for the heuristic search were obtained automatically by applying Neighbor-Join and BioNJ algorithms to a matrix of pairwise distances estimated using a JTT model, and then selecting the topology with superior log likelihood value. The tree is drawn to scale, with branch lengths measured in the number of substitutions per site. The analysis involved 15 amino acid sequences. There were a total of 633 positions in the final dataset. Evolutionary analyses were conducted in MEGA5. Accession numbers are given in the tree.
