# Supplementary material for: Unscrambling butterfly oogenesis
Source: BMC Genomics. 2013 Apr 26;14:283. doi: 10.1186/1471-2164-14-283 (PMC3654919; doi:10.1186/1471-2164-14-283)
Supplement: Additional file 10 — Oocyte and ovarian RNA quality. Provides the Agilent BioAnalyzer Electropherograms detailing oocyte and ovarian RNA quality prior to cDNA synthesis. [file 1471-2164-14-283-S10.pdf]

Additional file 10 – Oocyte and ovarian RNA quality

Agilent BioAnalyzer Electropherograms detailing **A**) oocyte (sample 4), and **B**) ovarian (sample 3) RNA quality prior to cDNA synthesis

A)

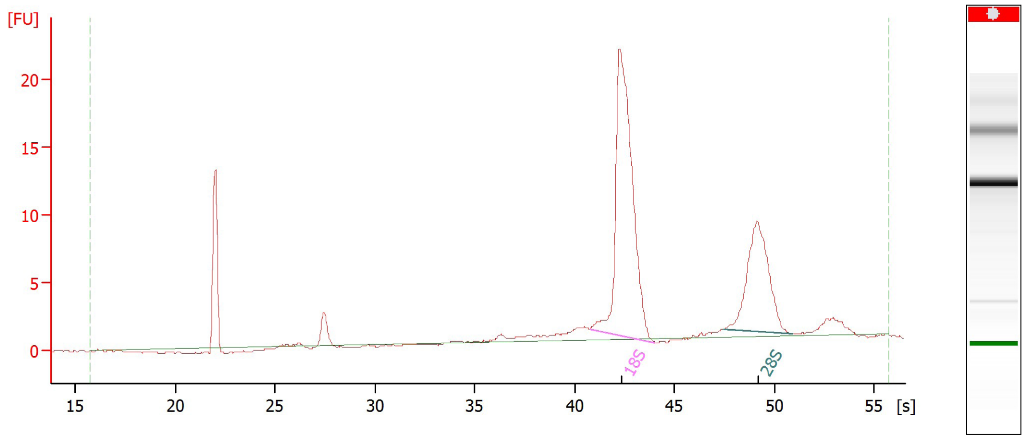

Overall Results for sample 4 : **W135 1:2000**

RNA Area:	88.9	RNA Integrity Number (RIN):	N/A (B.02.08)
RNA Concentration:	420 pg/µl	Result Flagging Color:	
rRNA Ratio [28s / 18s]:	0.4	Result Flagging Label:	RIN N/A

B)

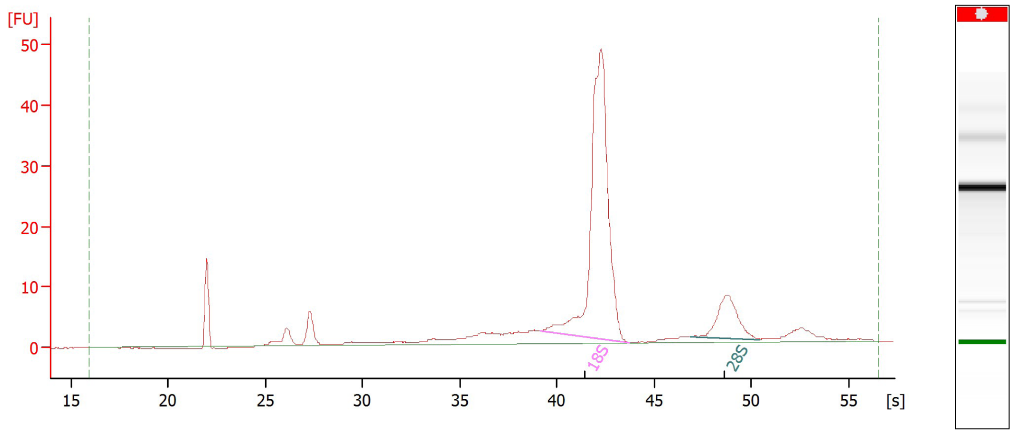

Overall Results for sample 3 : **A 1:2000**

RNA Area:	207.3	RNA Integrity Number (RIN):	N/A (B.02.08)
RNA Concentration:	979 pg/µl	Result Flagging Color:	
rRNA Ratio [28s / 18s]:	0.2	Result Flagging Label:	RIN N/A
