# Supplementary material for: Unscrambling butterfly oogenesis
Source: BMC Genomics. 2013 Apr 26;14:283. doi: 10.1186/1471-2164-14-283 (PMC3654919; doi:10.1186/1471-2164-14-283)

Additional file 11 – Filtering of BLAST hits in the automated annotation of the *Pararge aegeria* transcriptome

Similarity scores (SS) were assigned to each BLAST hit based on the bitscore (S'), number of positives in each alignment (P) and original contig length (L), calculated as SS = S'(P/L). Alpha and Beta BLAST hits are plotted with bitscores on the X-axis and the P/L ratio on the Y-axis providing a visualisation of the similarity score distribution. BLAST hits with a Similarity Score over a certain threshold are classified into 4 categories, High, Mild, Low Similarity, and Fail. Failed hits, are those hits with a bitscore (S') below 40, they were not annotated and the contigs were discarded.

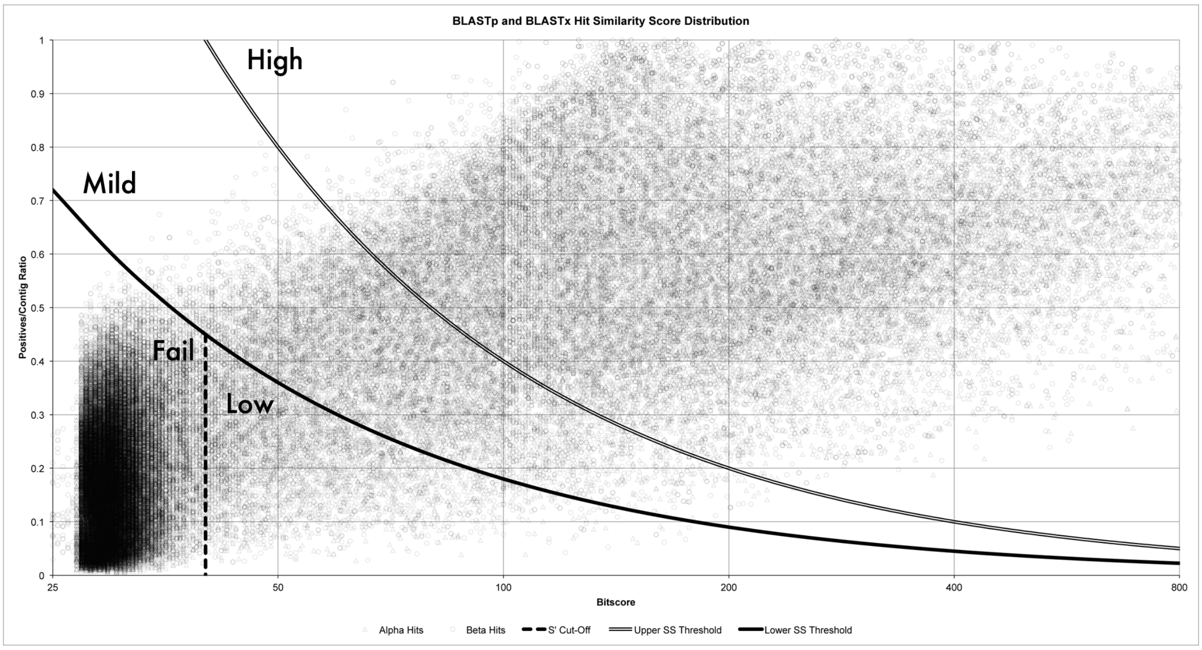

Supplement: Additional file 11 — Filtering of BLAST hits in the automated annotation. Provides a visualisation of the similarity score distribution and thresholds applied in the automated annotation of the P. aegeria transcriptome. [file 1471-2164-14-283-S11.pdf]
