# Supplementary material for: Unscrambling butterfly oogenesis
Source: BMC Genomics. 2013 Apr 26;14:283. doi: 10.1186/1471-2164-14-283 (PMC3654919; doi:10.1186/1471-2164-14-283)
Supplement: Additional file 12 — Automated annotation based on different BLAST Strategies. Provides a summary of the automated annotation method, detailing the different queries. [file 1471-2164-14-283-S12.pdf]

Additional file 12 - Automated annotation based on different BLAST strategies

Summary of the automated annotation method, detailing the different queries (e.g. *Pararge aegeria* translated Open Reading Frames (ORFs), or full nucleotide (nt) sequences), BLAST strategies employed and the publicly available sequence databases used for each of the queries/BLASTs. BLAST outputs were ranked, all hits were pooled and subsequently sorted with a minimum similarity score threshold as explained in the text.

VII* refers to the combined annotated protein databases for 6 insect species (*Drosophila melanogaster, Culex quinquefasciatus, Aedes aegypti, Pediculus humanus corporis, Bombyx mori* and *Harpegnathos saltator*) and 1 Arachnid (*Ixodes scapularis).* These databases were specifically consulted for the depth of their annotation.

DB	BLAST+	Group	Query	Taxonomy	Filter	Rank
nr	BLASTp	Alpha	ORF (aa)	*Eukaryota*	*UniProtKB/Swiss-Prot*	A
				*VII**	-	B
			Mt ORF (aa)	*Protostomia*	*Mitochondrion*	C
	BLASTx	Beta	Contig (nt)	*Eukaryota*	*UniProtKB/Swiss-Prot*	D
				*VII**	-	E
				Protostomia	-	F
nt	BLASTn	Delta	ORF (nt)	*Eukaryota*	*CDS*	G
			Contig (nt)	*Eukaryota*	*CDS*	H
		Zeta	Contig (nt)	-	-	I
