# Supplementary material for: Unscrambling butterfly oogenesis
Source: BMC Genomics. 2013 Apr 26;14:283. doi: 10.1186/1471-2164-14-283 (PMC3654919; doi:10.1186/1471-2164-14-283)
Supplement: Additional file 13 — Distribution of similarity classes across BLAST sources. Provides details regarding the number of Pararge aegeria contigs in each of the similarity classes, according to the BLAST strategy used in the automated annotation. [file 1471-2164-14-283-S13.pdf]

Additional file 13 - Distribution of similarity classes across BLAST sources.

Details regarding the number of *Pararge aegeria* contigs in each of the similarity classes, according to the BLAST strategy used in the automated annotation.

Source		High	Mild	Low	Fail	Total
BLASTp	*Alpha*	6097	1294	109	709	8209
	A	861	284	22	224	1391
	B	5214	1007	86	213	6520
	C	22	3	1	272	298
BLASTx	*Beta*	2226	1579	783	11376	15964
	D	187	198	88	4146	4619
	E	1016	606	259	3690	5571
	F	1023	775	436	3540	5774
Total		8323	2873	892	12085	24173
