# Supplementary material for: Unscrambling butterfly oogenesis
Source: BMC Genomics. 2013 Apr 26;14:283. doi: 10.1186/1471-2164-14-283 (PMC3654919; doi:10.1186/1471-2164-14-283)
Supplement: Additional file 14 — Thermocycler and qPCR reaction setup. Provides details regarding the reaction conditions and thermocycler programming parameters for successful qPCR amplification for each qPCR measurement reported in this study. [file 1471-2164-14-283-S14.pdf]

Additional file 14 - Thermocycler and qPCR reaction setup

Reaction conditions and thermocycler programming parameters for successful qPCR amplification for every qPCR measurement reported in this study. A melt curve was performed at the end of every plate run confirming the correct amplicon was amplified

Reaction Conditions		Thermocycler Parameters		
Absolute Blue qPCR Mix	1x	95°C	15 min	x1
Primer Mix	1.5 pmol/µl	95°C	15 s	x40
Template	20 ng/µl	60°C	1 min	
Reaction Volume	12.5 µl	65°C	5 s / 0.5°C	Melt Curve
ROX Reference Dye	100 nM	95°C		
